# Dynamic blood single-cell immune responses in patients with COVID-19

**DOI:** 10.1038/s41392-021-00526-2

**Published:** 2021-03-06

**Authors:** Lulin Huang, Yi Shi, Bo Gong, Li Jiang, Zhixin Zhang, Xiaoqi Liu, Jialiang Yang, Yongquan He, Zhilin Jiang, Ling Zhong, Juan Tang, Chunfang You, Qi Jiang, Bo Long, Tao Zeng, Mei Luo, Fanwei Zeng, Fanxin Zeng, Shuqiang Wang, Xingxiang Yang, Zhenglin Yang

**Affiliations:** 1 The Key Laboratory for Human Disease Gene Study of Sichuan Province and the Department of Laboratory Medicine, Sichuan Provincial People’s Hospital, University of Electronic Science and Technology of China, Chengdu, Sichuan China; 2 Research Unit for Blindness Prevention of Chinese Academy of Medical Sciences (2019RU026), Sichuan Academy of Medical Sciences, Chengdu, Sichuan China; 3 Natural Products Research Center, Institute of Chengdu Biology, Sichuan Translational Medicine Hospital, Chinese Academy of Sciences, Chengdu, Sichuan China; 4 Institute of Health Management, Health Management Center, Sichuan Provincial People’s Hospital, University of Electronic Science and Technology of China, Chengdu, China; 5 Department of Infection Disease, Zigong First People’s Hospital, Zigong, Sichuan China; 6 Department of Infection Disease, Mianyang No. 404 Hospital, Mianyang, Sichuan China; 7 Infectious diseases laboratory, Public Health and Clinical Center of Chengdu, Chengdu, Sichuan China; 8 Sichuan Dazhou Central Hosptical, Dazhou, Sichuan China; 9 Department of Infection Disease, Sichuan Provincial People’s Hospital, University of Electronic Science and Technology of China, Chengdu, Sichuan China

**Keywords:** Cell biology, Infectious diseases

## Abstract

The 2019 coronavirus disease (COVID-19) outbreak caused by the SARS-CoV-2 virus is an ongoing global health emergency. However, the virus’ pathogenesis remains unclear, and there is no cure for the disease. We investigated the dynamic changes of blood immune response in patients with COVID-19 at different stages by using 5’ gene expression, T cell receptor (TCR), and B cell receptors (BCR) V(D)J transcriptome analysis at a single-cell resolution. We obtained single-cell mRNA sequencing (scRNA-seq) data of 341,420 peripheral blood mononuclear cells (PBMCs) and 185,430 clonotypic T cells and 28,802 clonotypic B cells from 25 samples of 16 patients with COVID-19 for dynamic studies. In addition, we used three control samples. We found expansion of dendritic cells (DCs), CD14+ monocytes, and megakaryocytes progenitor cells (MP)/platelets and a reduction of naïve CD4+ T lymphocytes in patients with COVID-19, along with a significant decrease of CD8+ T lymphocytes, and natural killer cells (NKs) in patients in critical condition. The type I interferon (IFN-I), mitogen-activated protein kinase (MAPK), and ferroptosis pathways were activated while the disease was active, and recovered gradually after patient conditions improved. Consistent with this finding, the mRNA level of IFN-I signal-induced gene *IFI27* was significantly increased in patients with COVID-19 compared with that of the controls in a validation cohort that included 38 patients and 35 controls. The concentration of interferon-α (IFN-α) in the serum of patients with COVID-19 increased significantly compared with that of the controls in an additional cohort of 215 patients with COVID-19 and 106 controls, further suggesting the important role of the IFN-I pathway in the immune response of COVID-19. TCR and BCR sequences analyses indicated that patients with COVID-19 developed specific immune responses against SARS-CoV-2 antigens. Our study reveals a dynamic landscape of human blood immune responses to SARS-CoV-2 infection, providing clues for therapeutic potentials in treating COVID-19.

## Introduction

The war between humans and pathogenic viruses is never ending. Epidemic diseases, including Zika,^1^ severe acute respiratory syndrome (SARS),^2^ and Ebola,^3^ strikes quickly, often killing thousands of people during a single outbreak. In late December 2019, a novel coronavirus (SARS-CoV-2) belonging to the *Orthocoronavirinae* subfamily and distinct from the Middle East respiratory syndrome (MERS)-CoV and SARS-CoV, emerged.^4^ The rapid person-to-person spread of SARS-CoV-2, which causes the disease known as COVID-19, caused a global health emergency.^5^ Symptoms of COVID-19 include fever, myalgia, and fatigue, as well as dry cough, shortness of breath, sputum production, headache, hemoptysis, sore throat, and diarrhea. Lymphopenia, prolonged prothrombin time, and elevated lactate dehydrogenase levels have also been observed in patients with COVID-19.^6^ A computed tomography (CT) scan can identify bilateral patchy shadows or ground-glass opacity in the lungs in patients with COVID-19.^7^ Despite the high infection and mortality rates, there is no specific cure for the disease because its pathogenesis remains unclear.^8^

The human blood immune system plays a critical role in defending against viral infections. Many immune cells (such as leukocytes) and immune molecules (such as specific plasma proteins) are intrinsic components of blood. T cell receptor (TCR) mediates the recognition of pathogen-associated epitopes through interactions with peptide and major histocompatibility complexes (pMHCs). TCRs and B cell receptors (BCRs) are generated by genomic rearrangement at the germline level, a process termed the variable (V), diversity (D), and joining (J) segments of *CDR3* gene (V(D)J) recombination, a process that can generate marked diversity among TCRs and BCRs. Parameterizing the elements of antigen-specific immune repertoires across a diverse set of epitopes has the potential to create powerful applications in a variety of research fields for the diagnosis and treatment of infectious diseases.^9–11^ Recent studies have highlighted the importance of lymphocyte counts in the severe cases of COVID-19,^12,13^ suggesting the blood immune system is involved in the SARS-CoV-2 defense. Most recent studies on immune cell profiling of COVID-19 revealed the important role of an inflammatory immune signature in the blood^14^ and bronchoalveolar.^15^ Based on this knowledge, we set out to explore the atlas of blood immune cells in patients with COVID-19 by using Chromium Single-Cell immune profiling technology,^16–18^ to investigate the systematic mechanisms of the blood immune defense system against SARS-CoV-2.

## Results

### Sampling information for scRNA-seq

In total, 28 samples were included in the current study, including 25 samples from 16 patients with COVID-19 (different stages’ data for seven of the 16 patients were comparatively analyzed for a dynamic study) and three controls. The basic information and clinical features of the patients are listed in Supplementary Table S1. There were two critical cases (patient 1, male, 34 years old; patient 2, male, 33 years old), one severe case (patient 3, male, 43 years old), six moderate cases (patients 4–9, comprising two males and four females aged 25–62 years old), and three mild cases (patient 10, female, 19 years old; patient 11, male, 30 years old and patient 12, female, 32 years old). The four cured patients (patient 13–16, comprising two males and two females aged 20–40 years old) were enrolled on their discharge day from the hospital after they tested negative for SARS-CoV-2 and after the disease signs had disappeared. Three healthy people were included as normal controls (NC 1–3, two males and one female, aged 28–62).

Considering the kinetics of the body’s immune response in the blood involved in different stages of COVID-19, we performed a comparison study with seven of the 16 patients (patients 1, 2, 3, 6, 7, 9, and 10) who were comparatively analyzed during their battle with the disease at different stages.

### The single-cell transcriptome of PBMC

**Fig. 1:**
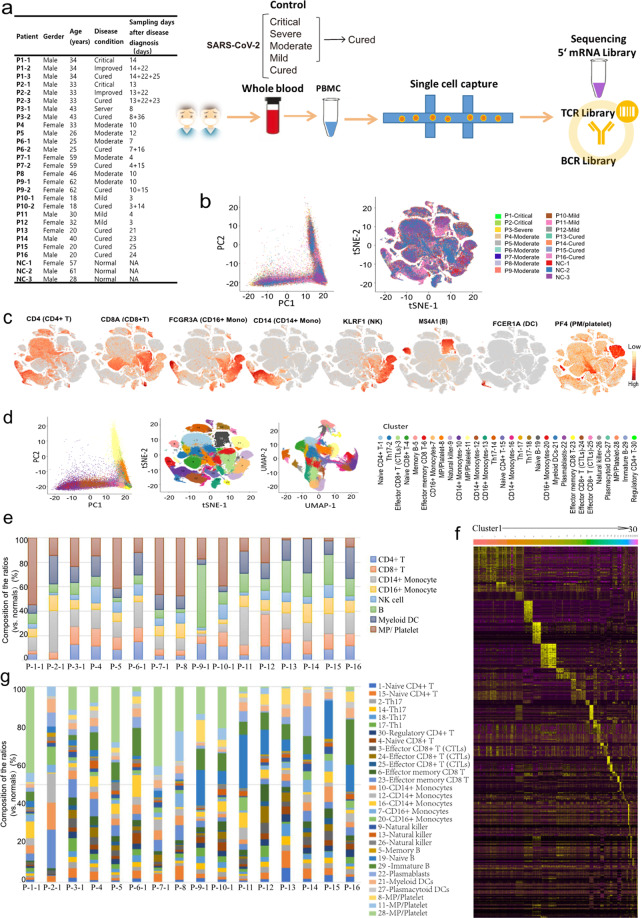
Cellular composition of the PBMCs in patients with COVID-19 and controls. **a** Brief summary of the samples for scRNA sequencing and the study design. The detailed information of the samples is shown in Table S1. **b** Integration analysis results of patients with COVID-19 and normal controls showing principal component (PC) and TSNE algorithm visualization. No beach effect was observed between the samples. **c** Eight main PBMC cell types identified by known cell markers. **d** Integration analysis results of patients with COVID-19 and normal controls showing principal component (PC), TSNE algorithm, and UMAP algorithm visualization. In total, 30 cell subtypes were identified in PBMC. **e** Composition of the ratios of the main cell type compositions of PBMC comparing each of the patients to normal controls. The proportion of each cell type in each sample was calculated and then compare the proportion of each cell type between cases and controls (for control samples, the mean proportion of three control samples was used for comparing). **f** The gene expression heatmap of the top 30 genes for each cell subtype. **g** Composition of the ratios of all the 30 cell subtype compositions of PBMC comparing each patient to normal controls. The proportion of each cell subtype in each sample was calculated and then compare the proportion of each cell subtype between cases and controls (for control samples, the mean proportion of three control samples was used for comparing)

The peripheral blood mononuclear cells (PBMCs) of each individual were isolated from the whole blood, and a scRNA-seq analysis of the PBMCs was performed on the 10X genomics platform with Chromium Next GEM Single-Cell V(D)J Reagent Kits v1.1(Fig. 1a).^18^ After quality control, in the first-round analysis, we obtained 241,292 PBMC cells, 131,391 TCR clones, and 23,674 BCR clones from the 19 samples of 19 individuals, including 16 patients with COVID-19 and three controls. Principal component analysis (PCA) and t-distributed Stochastic Neighbor Embedding (t-SNE) plots of single-cell gene expression suggested no batch effect among the 19 samples after normalization (Fig. 1b). In these samples, we identified eight completely different PBMC cell types, which could be further divided into 30 cell subtypes (clusters) based on the clustering results with Seurat V3 R package at a 0.9 resolution (Fig. 1c, d). The eight PBMC cell types include CD4+ T lymphocytes identified by *CD4*, CD8+ T lymphocytes identified by *CD8A*, CD16+ monocytes identified by *FCGR3A*, CD14+ monocytes identified by *CD14*, natural killer cells (NKs) identified by *KLRF1*, B cells identified by *MS4A1*, dendritic cells (DCs) identified by *FCER1A* and megakaryocyte progenitor cells/platelets (MP/platelets) identified by *PF4* (Fig. 1c). The t-SNE expression plot of the marker gene in part of the samples is shown in Supplementary Fig. S1. Detailed information for the 30 cell subtypes is shown in Fig.1d, including seven subtypes of CD4+ T lymphocytes, six subtypes of CD8+ T lymphocytes, four subtypes of B lymphocytes, and other cell subtypes. The dot plots of classical T cell marker genes and B cell markers were presented in Supplementary Fig. S2a, b, respectively.

### Comparison of PBMC cell subtype proportions between patients with COVID-19 and the controls

To evaluate the blood immune system states of patients with COVID-19, we first compared the proportion of the main eight cell types of each of the COVID-19 patients with the normal controls. We found the expansion of myeloid DCs, CD14+ monocytes, and MP/platelets in most of the patients with COVID-19, and decreased CD16+ monocytes and NKs in more than half of the patients with COVID-19 (Fig. 1e). To further investigate the changes of the 30 cell subtypes in different conditions of COVID-19, we compared the cell subtype proportions in each of the patients with COVID-19 to the three normal controls (Fig. 1e). A subtype of naïve CD4+ T lymphocytes (cluster 1), which are marked by highly expressed ribosome coding genes, such as *RPL32, RPL30, RPS12*, and *RPS3A*, was decreased in all hospitalized patients. Given that ribosome coding genes play critical roles in viral RNA transcription and replication (CL: 14985, in the STRING database), high expression of the ribosome coding genes in the naïve CD4+ T lymphocytes might be associated with the replication of the virus.^19^ At the same time, naïve CD8+ T (clusters 4, highly expressed noncoding RNA *LINC02446*, elongation factors *EEF1A1* and *EEF1B2*), and NKs (clusters 13, highly expressed a cystatin superfamily gene *CST7*, killer cell lectin-like receptors *KLRD1* and *KLRG1*) also were found to be decreased in more than half of the patients. On the other hand, some cell subtypes including effector memory CD8 T (cluster 23, highly expressed *STMN1*, *HMGB2*, *TUBA1B*, *HIST1H4C*), CD14+ monocytes (clusters 10, highly expressed *FCN1*, *LYZ*, *S100A8*, cluster 12, highly expressed *S100A12*, *S100A6*, and *TYROBP*, and cluster 16, highly expressed *CAMP*, *LCN2*, *CCL3*, and *CCL3L1*), naive B cells (cluster 19, which highly express *CD74*, *CD79A*, and *HLA-DRA*), myeloid DCs (cluster 21, highly expressed *HLA-DRB5, HLA-DPB1*, and *HLA-DQA1*), and MP/Platelets (cluster 11, highly expressed *CAVIN2*, *HIST1H2AC*, and *TUBB1*, and cluster 28, highly expressed *HIST1H4H*, *HIST1H3H*, *H3F3A*, and *NT5C3A*) were found to be increased in more than half of the patients. The gene expression heatmap of the top 30 genes in each cluster was shown in Fig. 1f.

The proportion of 15 out of the 30 cell subtypes, such as CD4+ T, and CD8+ T cells, dramatically decreased in both of the critical patients (patient 1 and patient 2). This is consistent with the much lower number of lymphocytes in these two patients (0.75 × 10^9^/L for patient 1 and 0.27 × 10^9^/L for patient 2) compared with the normal range (1.1–3.2 × 10^9^/L, Table S1). In the severe condition (patient 3), the proportions of some cell subtypes such as terminal effector CD8 T and effector memory CD8 T cells were increased. For the moderate patients (patients 4–9), they also presented a medium degree of changes in cell proportions (Fig. 1e, g), except for patient 9. In patient 9, who had moderate symptoms, her cell proportions of naive B cells (clusters 19) and subtypes of CD4+ and CD8+ T cells (clusters 4, 14 17, 18, and 23) were greatly expanded (Fig. 1e, g). For the mild patients (patients 10–12), they also presented a medium degree of changes in cell proportions (Fig. 1e, g), except for patient 11. In patient 11, the cell proportion of a subtype of naive B cell (clusters 19) is the highest among all patients, 12.52 times increase compared with controls (Fig. 1e, g). In the cured condition, the proportion of MP/platelets (clusters 11 and 28) decreased significantly. These results suggest that human blood immune responses are highly regulated individually according to the disease conditions during SARS-CoV-2 infection, and immune cell imbalance is associated with COVID-19.

**Fig. 2:**
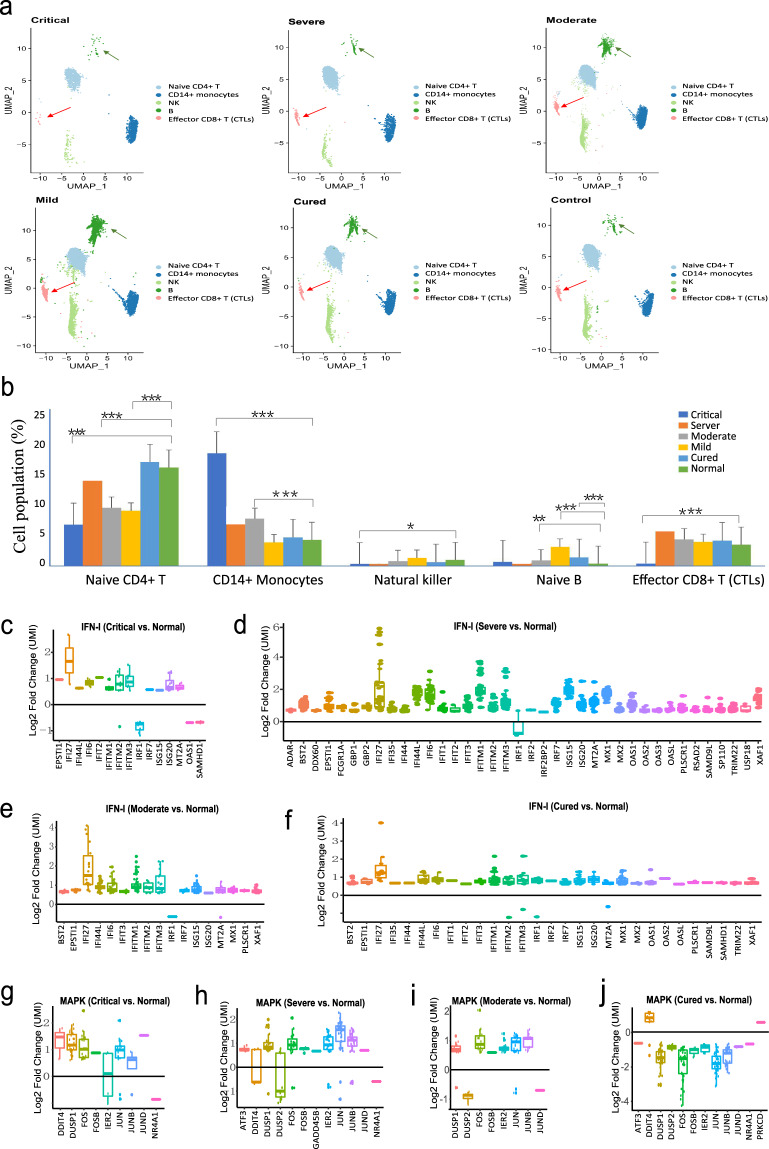
UMAP projection of five cell clusters and Gene expression changes of IFN-I and MAPK pathway in response to SARS-CoV-2 infection. **a** UMAP projection of five cell clusters among critical (*n* = 2), severe (*n* = 1), moderate (*n* = 6), mild (*n* = 3), cured (*n* = 4), and controls (*n* = 3). naive CD4 + T: cluster 1 in Fig. 1d; CD14+ monocytes: cluster 10 in Fig. 1d; NKs: cluster 13 in Fig. 1d; B cells: cluster 19 in Fig. 1d; terminal CD8+ effector T: cluster 24 in Fig. 1d. The red arrow in **a** indicated the effector CD8 + T (CTLs), the green arrow in **a** indicated the B cells. They are significantly changed comparing to COVID-19 patients and controls. The related section is explained in the bottom of Page 7 of the main text. **b** Statistical significance (each of the other conditions vs. controls) for the cell population mentioned in **a**. *p < 0.05; **p < 0.01; ***p < 0.001. **c**–**f** Log2 fold changes of the unique molecular identifiers (UMI) counts of IFN-I pathway genes comparing patients to normal controls. **c** The critical patients (patient 1 and patient 2) vs. normal controls. **d** The severe patient (patient 3) vs. normal controls. **e** The moderate patients (patients 4–9) vs. normal controls, and **f** The cured patients (patients 11 and 10) vs. normal controls. **g**–**j** Log2 fold changes of the UMI counts of MAPK targets in critical (**g**), severe (**h**), moderate (**i**), and cured patients with COVID-19 vs. normal controls (**j**), respectively. Each point represents a different cell subtype

To give detailed information about the cell proportion changes in the different disease conditions, we further analyzed cell subtypes in the five clusters with dramatic cell proportion changes in the studied disease conditions: cluster 1 (naïve CD4+ T), cluster 10 (CD14+ monocytes), cluster 13 (NK), cluster 19 (naive B), and cluster 24 (terminal effector CD8 T) (Fig. 2a, b). Findings show that the terminal CD8+ effector cell depletion is associated with disease critical condition (red arrow in Fig. 2a, b). Compared with the other conditions, the mild group presents a much higher naive B cell proportion (green arrow in Fig. 2a, b). These results suggest that terminal CD8+ effector T cell depletion might be associated with patients in critical condition, and a large part of naive B cell expansion for antibody production may prevent COVID-19 from worsening.

### Differentially expressed pathways between patients with COVID-19 and controls

Then, we analyzed the gene expression profiles in each cell subtype of patients with different COVID-19 conditions and compared these profiles to the controls. We identified many differentially expressed (DE, FDR < 0.05) genes between patients with COVID-19 and normal controls in each cell cluster. To further explore the enriched signal pathways of the DE genes in each cell subtype, we performed a local STRING network analysis,^20–22^ using DE genes and their log2 changed folds between the patients and controls. In patients with critical, severe, moderate, mild, and cured conditions, we found that 16 (in 9 cell subtypes), 22 (in 26 cell subtypes), 22 (in 20 cell subtypes), 28 (in 16 cell subtypes), and 19 (in 9 cell subtypes) signal pathways were significantly changed for each disease condition, respectively (FDR < 0.05) (Supplementary Figs. S3–S7, Table 2–6).

By STRING network analysis, we found that besides basic metabolic signaling pathways, such as respiratory and oxidative phosphorylation, the significantly enriched signal pathways were mainly involved in the overactivation of five pathways (Fig. 2c–j, Supplementary Figs. S3–S7): (1) viral genome replication and infection (viral mRNA translation, peptide chain elongation, and SRP-dependent co-translational protein targeting the membrane, which was highly expressed in CD14+ monocytes, naïve CD4+ T cells, and other cells); (2) type I interferon signaling (IFN-I) (highly expressed in naive B cells and CD14 + monocytes); (3) mitogen-activated protein kinase (MAPK) (also including AP-1, Jun and bZIP Maf transcription factor) (highly expressed in naive B cells, CD8+ T cells, and NKs); (4) immunology interactions between lymphoid and nonlymphoid cells (highly expressed in terminal CD8+ effector T and naive B cells); and (5) major histocompatibility complex (MHC) class II protein complex (highly expressed in B cells). Most genes involved in these pathways were upregulated in hospitalized patients with COVID-19 compared with the normal controls (Fig. 2c–j, Supplementary Fig. S8–S9). However, the expression of MAPK pathway genes was downregulated in the cured patients, suggesting that decreased expression of MAPK pathway genes is a good sign for a patient’s recovery (Fig. 2j).

To study the signaling alterations associated with disease severity, which might lead to new intervention strategies, we have further compared the critical/severe condition with the moderate condition (Supplementary Fig. S10) and mild condition (Supplementary Fig. S11). Respiratory and G alpha (i) signaling events enriched in the critical/severe condition compared with the moderate ones in PM/Platelets (cluster 8). Many signaling pathways were altered for the comparison of critical/severe conditions with the mild condition, and most of the enriched signaling pathways are included or related to the five pathways mentioned above besides basic metabolic signaling pathways (Supplementary Fig. S11).

### Paired comparison of patients with COVID-19 before and after disease improvement

**Fig. 3:**
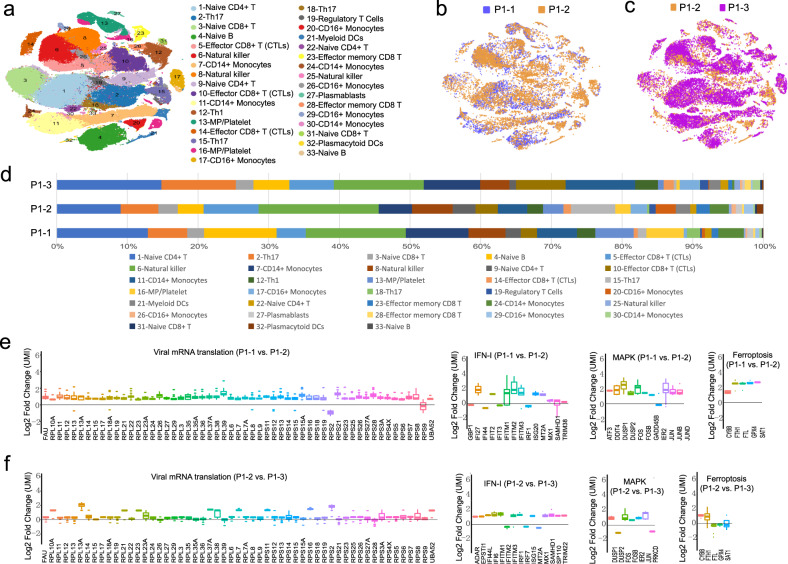
The dynamic study for critical patient 1 before and after improvement. **a** TSNE plots of cell types identified in patients with dynamic study (P1-1, P1-2, P1-3, P2-1, P2-2,m P2-3, P3-1, P3-2,P6-1, P6-2, P7-1, P7-2, P9-1, P9-2, P10-1, and P10-2). **b** TSNE plots of P1-1 (in critical condition) and P1-2 (in improvement condition), showing the cell type distribution in the two samples. **c** TSNE plots of P1-2 and P1-3 (in cured condition), showing the cell type distribution in the two samples. **d** Comparisons of all the cell subtypes of P1-1, P1-2, and P1-3. **e** Log2 fold changes of the UMI counts of the genes involved in viral mRNA translation, IFN-I, MAPK targets, and ferroptosis between P1-1 and P1-2. Each point represents a different cell subtype. **f** Log2 fold changes of the UMI counts of the genes involved in viral mRNA translation, IFN-I, MAPK targets, and ferroptosis between P1-2 and P1-3. Each point represents a different cell subtype

To further explore the immune response to SARS-CoV-2 before and after the patients recovered from the disease, we collected a second round of nine additional samples from seven patients who successfully recovered; which produced 100,128 PBMC cells, 54,039 TCR clones, and 5128 BCR clones (Supplementary Table S1). For the two critical patients—patient 1 and patient 2—we collected blood samples and performed scRNA-seq again when their disease improved (22 days after the first round of sampling for both patients) and after they were cured (25 and 23 days after the improved sampling for patient 1 and patient 2, respectively). In the other five patients (one severe patient, patient 3, three moderate patients, patients 6, 7, and 9 and one mild patient, patient 10), we collected blood samples and performed scRNA-seq analyses again right after the patients recovered from the disease. Therefore, we could compare the blood immune scRNA-seq changes in disease, and after the patients had recovered. Combined with these samples and the first-round data of these patients, cell subtypes identified based on the clustering results with Seurat V3 R package at a 0.9 resolution were shown in Fig. 3a.

We observed that the reduction or expansion of cell types and subtypes significantly improved after recovering from the disease. Pathways including viral mRNA translation, IFN-I, MAPK, immunology interactions, and MHC class II protein complex were significantly enriched in most of the paired comparisons of the before and after improvement states for the seven patients, further suggesting that these five pathways are involved in the pathogenesis of COVID-19 (Supplementary Figs. S12–S19). The gene expression profiles of most of these pathways are also consistent with our above results when comparing the patients with the controls. That is, most of these genes are upregulated in disease conditions of COVID-19.

In critical patient 1, by comparing his disease conditions in the critical with improved stages (P1-1 vs. P1-2), we observed his terminal CD8+ effector T (clusters 10) and NKs (clusters 6) reduction, etc. were recovered in the improved stage (Fig. 3b, c, and d). To give more detailed pathway information related to his recovery process, we did both a local STRING network analysis and KEGG analysis (using the DE gene list). For the STRING network analysis, we found the changes of pathways and gene expression patterns between the critical stage and improved stage were similar to our findings from the first-round comparison between critical patients and controls (Fig. 3d, Supplementary Fig. S12). Pathways including viral mRNA translation, IFN-I, and MAPK were significantly upregulated in the critical stage when comparing to the improved stage (Fig. 3e). The KEGG analysis revealed that a form of regulated cell death—ferroptosis—was significantly upregulated in the disease critical stage comparing to that of the improved stage (Fig. 3e, Supplementary Fig. S13). And then, by further comparing this patient’s disease conditions between the improved and cured stages (P1-2 vs P1-3, we found that the proportions of Th1 cell (cluster 19) were recovered in the cured stage (Fig. 3c, d). Pathways including viral mRNA translation, IFN-I, and MAPK were also upregulated in the improved stage when comparing to the cured stage (Fig. 3f, Supplementary Figs. S14-S15). The gene expression profiles involved in ferroptosis were not significantly changed between the improved and cured stages, indicating that ferroptosis mainly happened in the critical stage and gradually returned to normal at the improved stage (Fig. 3f). Similar changes of cell types and subtypes, pathways, and gene expression patterns were found in another critical patient—patient 2—when comparing the critical, improved, and cured stages (Supplementary Figs. S16–S20).

**Fig. 4:**
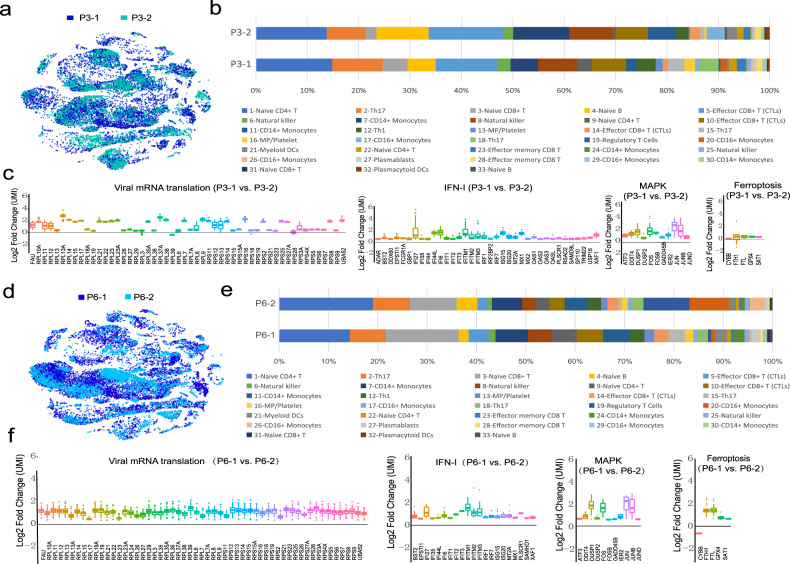
The dynamic study of severe patient 3 and moderate patient 6 in their disease and cured conditions. **a** TSNE plots of cell types identified in patients with dynamic study of P3-1 (severe condition) and P3-2 (cured condition). **b** Comparisons of all the cell subtypes of P3-1 and P3-2. **c** Log2 fold changes of the UMI counts of the genes involved in viral mRNA translation, IFN-I, and MAPK targets between P3-1 and P3-2. Each point represents a different cell subtype. Only DE genes (FDR < 0.05) were plotted for the comparison group. The cell subtype is shown in Fig. 3a. **d** TSNE plots of cell types identified in patients with dynamic study of P6-1 and P6-2 showing the cell type distribution in the two states. **e** Comparisons of all the cell subtypes of P6-1 and P6-2. **f** Log2 fold changes of the UMI counts of the genes involved in viral mRNA translation, IFN-I, MAPK targets, and ferroptosis between P6-1 and P6-2. Each point represents a different cell subtype. Only DE genes (FDR < 0.05) were plotted for the comparison group. The cell subtype is shown in Fig. 3a

Patient 3 with severe symptoms showed lymphocyte expansion when compared to the normal controls in the first-round analysis; his lymphocyte expansion reduced at the cured stage (Fig. 4a, b). Pathways including viral mRNA translation, IFN-I, and MAPK were significantly upregulated in the severe stage when comparing to the cured stage (Fig. 4c). However, the ferroptosis signal pathway was just slightly upregulated in the disease severe stage (Supplementary Figs. S21–S22), it is likely because of his lymphocyte cells’ expansion. For the three moderate patients with COVID-19—patients 6, 7, and 9—both IFN-I and MAPK pathways were significantly upregulated in the disease stage when comparing to the cured stage (Fig. 4d–f, Supplementary Figs. S23–S30). The viral mRNA translation and ferroptosis pathways were significantly upregulated in the disease stage when comparing to the cured stage for patients 6 and 7. However, the virus mRNA translation singling and ferroptosis were not significantly upregulated in the illness stage when comparing to the cured stage for patients 9, which might be explained by her relatively higher T and B cell expansion^23^ (Supplementary Figs. S27–S29). The mild condition patient—patient 10—showed a mild degree of the immune response.

### TCR and BCR expansion in patients with COVID-19

**Fig. 5:**
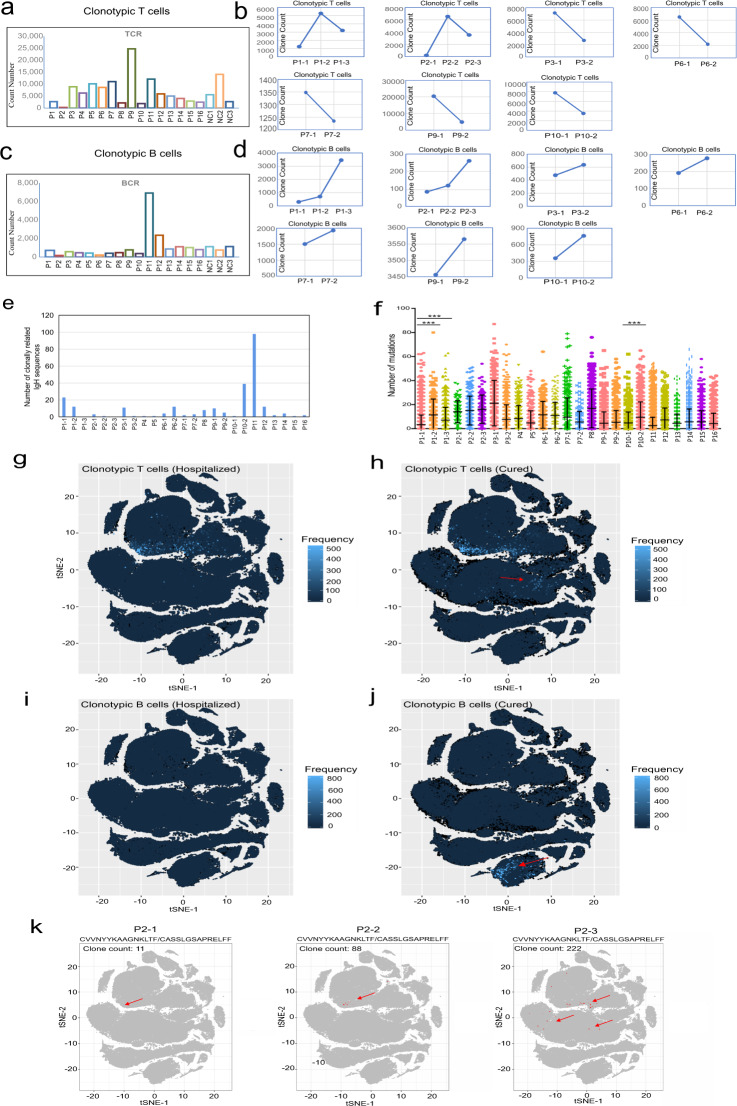
T lymphocytes and B lymphocytes V(D)J clone expansion and TSNE plots of integrated analysis of 5′mRNA, T cell receptor (TCR), and B cell receptors (BCR) of patients with COVID-19. **a** The number of T cell clones detected by V(D)J in each patient. **b** The number of T cell clones detected by disease condition and the recovery condition of patients 1, 2, 3, 6, 7, 9, and 10. **c** The number of B cell clones detected by V(D)J in each patient. **d** The number of B cell clones detected at disease and recovery conditions of patients 1, 2, 3, 6, 7, 9, and 10. **e** Clonally related IgH sequences in each patient. **f** Number of mutations in IgH gene variable regions in different COVID-19 patients. The statistical significance refers to the statistics of different courses of the same patient who had dynamic analysis data. *P < 0.05, **P < 0.01, ***P < 0.001; *t-*test. **g** Clonotypic T cells of hospitalized patients. **h** Clonotypic T cells of cured patients. **i** Clonotypic B cells of hospitalized patients. **j** Clonotypic B cells of cured patients. Patients in hospitalized condition: P1-1, P1-2, P2-1, P2-2, P3-1, P6-1, P7-1, P9-1, and P10-1. Patients in cured condition: P1-3, P2-3, P3-2, P6-2, P7-2, P9-2, and P10-2. The detailed cell types of these patients are shown in Fig. 3a. **k** TSNE plot of a clonotypic T cell (terminal effector CD8 T cells): CVVNYYKAAGNKLTF_CASSLGSAPRELFF (TRAV12-1*01, TRAJ17*01, and TRAC*010 in P2-1, P2-2, and P2-3)

To explore the clonal proliferation of TCR and BCR in COVID-19, we conducted TCR and BCR V(D)J^16,18,24^ single-cell transcriptome analysis. Using an integration analysis, we detected 185,430 clonotypic T cells and 28,802 clonotypic B cells in the 28 samples obtained from 19 participants. The repertoire in patients with COVID-19 and controls showed large diversities of the clonotypic T cells and B cells in each individual. The distributions of clonotypic T cells for each patient are shown in Fig. 5a (Supplementary Table S7). By comparing the clonotypic T cells of the seven patients before and after the improvement of disease, we found that the relative quantity of clonotypic T cells of patients decreased from the disease to the cured conditions (Fig. 5b). However, lower clonotypic cells were detected in critical conditions (patient 1–1 and patient 2–1), which might be due to lymphocyte exhaustion during this disease stage. The highest relative quantity of clonotypic T cells was detected in the moderate patient 9 (Fig. 5a, b), which might be due to the expansion of her lymphocytes.

Compared to the clonotypic T cells, the total number of clonotypic B cells detected was much lower (Fig. 5c, d, Supplementary Table S8). The relative quantity of total clonotypic B cells of patients increased with the disease condition improvement (Fig. 5d). The V(D)J segments of the immunoglobulin (Ig) genes are rearranged in an ordered fashion to generate the primary Ig repertoire during the development of B cells before the encounter with antigen; during the immune response, activated B cells are clonally expanded within the germinal center and the variable region of Ig genes can be further mutated to produce high-affinity antibodies.^25^ Detailed analyses showed that almost all these COVID-19 patients had developed clonally related IgH genes (Fig. 5e). The variable regions of IgH genes from all the patients are highly mutated, with an average of 9.39 nucleotides in each gene (Fig. 5f).

We further performed an integrated analysis of 5′ gene expression libraries, T cell receptor (TCR), and B cell receptor (BCR) data by mapping the clonotypic T cells and clonotypic B cells in the TSNE plot of the 5′ gene expression libraries scRNA data (Fig. 3a) to compare the TCR and BCR expansion of the same patients in hospitalized condition (P1-1, P1-2, P2-1, P2-2, P3-1, P6-1, P7-1, P9-1, and P10-1) and cured condition (P1-3, P2-3, P3-2, P6-2, P7-2, P9-2, and P10-2). We found that the clonotypic T cells in hospitalized conditions are mainly terminal CD8 + effector T cells (Fig. 5g); in cured condition, high-frequency clonotypic T cells in Th1/Th17/Tregs were detected besides terminal CD8+ effector T cells (Fig. 5h, red arrow). For the clonotypic B cells, no excessively high-frequency expansion is found in the hospitalized condition of these patients (Fig. 5i). However, in the cured condition of these patients, high-frequency clonotypic B cells can be detected in the naive B cells (Fig. 5j, red arrow). These results present the cell expansion dynamic landscape of T and B lymphocytes.

The patients with COVID-19 showed different degrees of specific memory T cell expansion or preservation with the disease improvement. Using the scRNA-seq data, we could identify antigen-receptor sequences in the memory clonotypic T cells during different disease conditions. The clonotypic effector memory CD8 T cells with the following TCR variable sequences significantly increased in critical patient 2 at the different stages of the disease. (1) CVVNYYKAAGNKLTF_CASSLGSAPRELFF (TRAV12-1*01, TRAJ17*01 and TRAC*01) had 11 clones in P2-1 (critical), 88 clones in P2-2 (improved) (FDR = 5.99 × 10^−4^, P2-1 vs. P2-2, adjusted binomial probabilities, the same below), and 222 clones in P2-3 (cured) (FDR = 9.48 × 10^−39^, P2-2 vs. P2-3) (Fig. 5k). These clones changed from terminal effector CD8 T cells to effector memory CD8 T cells from disease condition to cured condition (Fig. 5k). (2) CAMKTSYDKVIF_CASTPGDTIYF (TRAV12-3*01, TRAJ50*01, TRAC*01) had 6 clones in P2-1, 23 clones in P2-2 (FDR = 3.92 × 10^−5^, P2-1 vs. P2-2), and 50 clones in P2-3 (FDR = 8.32 × 10^−6^, P2-2 vs. P2-3). (3) CAFRKDTGRRALTF_CASSPQGGGGYTF (TRAV24*01, TRAJ5*01, TRAC*01) had 4 clones in P2-1, 12 clones in P2-2 (FDR = 9.75 × 10^−4^, P2-1 vs. P2-2), and 24 clones in P2-3 (FDR = 0.01, P2-2 vs. P2-3). These clonotypic cells expansions and preservations may be related to the memory of SARS-CoV-2 antigens in patients.

By analyzing Ig coding genes that changed during the course of the disease, we found that the highest changed genes belonged to the IGHV3 family (*IGHV3-21*, *IGHV3-22*, *IGHV3-30*, *IGHV3-48*, *IGHV3-49*, *IGHV3-72*, etc.). Other families, such as *IGKV1*, *IGKV3*, *IGLV2*, *IGLV3*, *IGLV1*, and *IGHV4*, were also highly changed in different disease conditions (Fig. S31a). The values of IgG and IgM of these samples were shown in Supplementary Fig. S31b. In the recently cured condition, patients had more Ig genes than normal controls (Supplementary Fig. S32). Together, the adaptive immunity involved in B cell antibody production may play a crucial role in the fight against SARS-COV-2.

### Experimental detection of IFN-I and MAPK signals

**Fig. 6:**
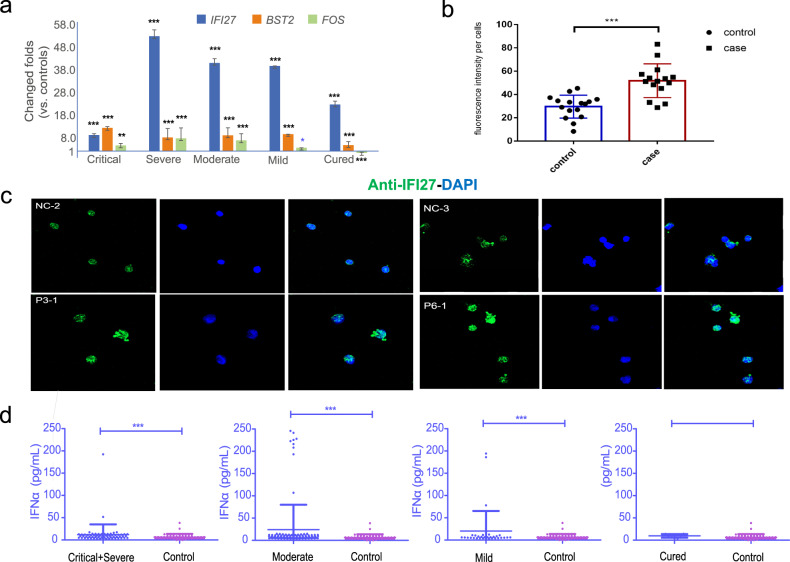
Experimental validation of IFN-I signaling. **a** Real-time PCR validation of *IFI27* and *BST2* in the interferon pathway and *FOS* in the MAPK pathway. *IFI27* and *BST2* are upregulated in patients with COVID-19. *FOS* is upregulated in hospitalized patients but downregulated in cured patients (*N* = 3 for critical, *N* = 3 for severe, *N* = 19 for moderate, *N* = 3 for mild, and *N* = 10 for cured patients with COVID-19 and 35 normal controls). *P < 0.05, **P < 0.01, ***P < 0.001; Student’s *t*-test. **b** Statistical significance of IFI27 immunofluorescence signal comparing COVID-19 and normal controls. *p < 0.05; **p < 0.01; ***p < 0.001. **c** Immunofluorescence staining of *IFI27* in PBMCs of patients with COVID-19 and normal controls. **d** IFN-α concentration in serum of 257 patients with COVID-19 and 106 matched tested by ELISA, including of the severe and critical group (12.02 ug/mL ± 2.80, *N* = 68), the moderate group (23.99 g/mL ± 4.840, *N* = 133 patients), the mild group (20.29 ug/mL ± 7.732, *N* = 34 patients), and the cured group (9.637 ug/mL ± 1.184, *N* = 22 patients). *P < 0.05, **P < 0.01, ***P < 0.001; one-way ANOVA

From the scRNA-seq analysis, we found that a set of genes involved in the IFN-I, MAPK pathways were stably upregulated in patients with COVID-19. To further validate this finding, we performed quantitative real-time reverse transcriptase-polymerase chain reaction (real-time RT-PCR) testing to detect the expressions of related genes in another cohort of 38 patients with COVID-19, including three critical, three severe, 19 moderate, three mild, and 10 cured patients with COVID-19 and 35 normal controls (Fig. 6a). *IFI27*, a reported biomarker of influenza distinct from bacterial infection,^26^ was the strongest upregulated gene in patients with COVID-19 in the scRNA-seq data. It was upregulated 8.1 times in critical patients, 51.7 times in severe patients, 39.9 times in moderate patients, 38.6 times in mild patients, and 29.5 times in cured patients compared with the normal controls (Fig. 6a). We performed PBMC immunofluorescence testing to compare the protein expressions of *IFI27* between patients with COVID-19 and the controls. Immunofluorescence staining showed that *IFI27* was highly expressed in the lymphocytes of patients with COVID-19 but only partially expressed in the lymphocytes of the controls (Fig. 6b, c). Another gene in the IFN-I pathway, *BST2*, also showed upregulation in patients with COVID-19 compared with controls (Fig. 6a). These results suggest that *IFI27* and *BST2* are candidate marker genes for SARS-CoV-2 infection. Consistent with our scRNA-seq data, the expression profile of *FOS*, which is a transcription factor mediating the MAPK pathway, showed significant upregulation in patients with COVID-19 but obvious downregulation in recovered patients by real-time RT-PCR analysis (Fig. 6a). This suggests that *FOS* is a candidate marker for COVID-19 patient recovery.

We further measured total IFN-α concentration in the serum of 257 patients with COVID-19 and 106 matched controls by using enzyme-linked immunosorbent assay (ELISA). The patients were placed into four groups: the critical and severe group, the moderate group, the mild group, and the cured group. We found that the total IFN-α level was significantly increased in patients than in controls (*p* < 0.001, 17.79 ug/mL ± 2.55 vs. 6.42 ug/mL ± 0.703). IFN-α was increased dramatically in the moderate group (*p* < 0.001, 23.99 ug/mL ± 4.840, *N* = 133 patients) and mild (*p* < 0.001, 20.29 ug/mL ± 7.732, *N* = 34 patients) patients (Fig. 6d). The IFN-α concentration in the serum of the critical and severe group is 12.02 ug/mL ± 2.80 (*N* = 68) and the cured group is 9.637 ug/mL ±1.184 (*N* = 22, *p* < 0.05,) (Fig. 6d). This result suggests that patients in moderate condition produced the highest level of IFN-α.

## Discussion

In the current study, we revealed the host immune process in the blood of patients with COVID-19. During disease progression, the host immune cells are imbalanced, and some T lymphocyte subtypes are reduced, while MP/platelets are increased. The enriched five pathways involved in the pathogenesis of COVID-19 and the fight against SARS-CoV-2 may work together. First, the host immune response may be activated through immunology interactions between lymphoid and nonlymphoid cells in certain types of CD8+ effector T and B cells to kill and remove virus, when the virus enters the human body. Then, the cytokine immune response mainly based on the IFN-I and MAPK pathways in certain types of CD14+ monocytes, B cells, and NKs may kill the virus. At the same time, the virus replication signals may be activated mainly in CD14+ monocytes, naïve CD4+ T cells, and other immune cells. Finally, the major histocompatibility complex (MHC) class II protein complex in the types of B cells that are involved in specific antigen presentation may be activated accordingly to kill the virus or inhibit virus replication to combat SARS-CoV-2. Our research provides a clue for understanding the pathogenesis of COVID-19, disease prevention, and control of the disease.

Previous studies have suggested decreased lymphocyte counts in severe COVID-19 cases.^6,27–30^ We observed naïve CD4+ T cells, Th1/Th17, naïve CD8+ T cells, and terminal effector CD8 T cells were lower in patients in critical condition. Lower levels of T lymphocytes suggest a role for dysregulated immune responses in COVID-19 pathogenesis. It has been shown that T cells, especially CD4+ T cells and CD8+ T cells, play an important role in virus infection and immune homeostasis.^31^ The balance between the naïve CD4+ T lymphocytes is crucial for maintaining an efficient immune response. As in the SARS-CoV disease,^32^ the decrease of CD4+ T cells and CD8+ T cells might be caused by fast replication and quick spread of SARS-CoV-2 and its resulting immunopathology damage.^33^ Our study suggests that the activation of the IFN-I and the MAPK signaling pathways play a critical role in blood immune system to combat SARS-CoV-2 infection. The genes of these two pathways are widely expressed in PBMC (Fig. S32).IFN-I is crucial for promoting antiviral defenses through the induction of antiviral effectors.^33^ The production of the multifunctional cytokines IFN-I (IFN-α and IFN-β) is one of the earliest innate responses induced by viral infections.^33–37^ MAPK pathways have roles in IFN-I production, viral replication, mucus production, and T cell responses, all of which are important processes in infection airway disease.^38^ We found the serum IFN-α levels of patients with COVID-19 are higher than that of normal controls, and the highest elevation was found in moderate and mild patients, which may suggest that an increase of IFN-α in the blood is beneficial for killing SARS-CoV-2. On the other hand, a recent report suggested that SARS-CoV-2 could exploit IFN-driven gene *TMPRSS2* for the upregulation of *ACE2* to enhance infection during lung injury.^39^ Therefore, the side effects of IFN-I should be noted when it is used for the treatment of COVID-19. It is important to explore when and how much IFN-α should be used to treat COVID-19 in the future. We anticipate our assay to be a starting point for investigation of potential IFN-I usage for a COVID-19 treatment.

The clinical features of COVID-19 vary from diseases caused by SARS-CoV and MERS-CoV, such as the speed of transmission speed, treatment scheme, and mortality rate, which vary from those of diseases caused by SARS-CoV and MERS-CoV.^40,41^ However, as for COVID-19, hematological abnormalities, such as thrombocytopenia and lymphopenia, are common between patients of SARS-CoV,^42,43^ and MERS-CoV.^40,44^ In this study, similar to those with SARS-CoV and MERS-CoV, critical patients with COVID-19 had lymphopenia. The IFN family of cytokines provides the first line of defense against viral pathogens. Patients initiate transcription of hundreds of IFN-stimulated genes that have antiviral, immunomodulatory, and cell regulatory functions.^41^ In SARS-CoV and MERS-CoV, the host fails to induce IFNs until 12 h after infection, which correlates with disease severity and clinical course.^41,45^ How quickly the IFN family of cytokines is induced and how the time course of IFN induction could impact the severity of COVID-19 remains unclear. We also identified some molecules such as S100A8 and S100A9, which may have promising to be potent biomarkers to predict severe COVID-19 patients, with CETP and CRP (Supplementary Table S2–S5).^46^

We observed that the ferroptosis-related genes, including *GPX4*, *FTH1*, *FTL*, and *SAT1*, were upregulated in disease conditions but downregulated during recovery. Ferroptosis is an iron-dependent cell-death modality triggered by dyshomeostasis of three metabolic pillars: iron, thiols, and polyunsaturated phospholipids.^47^ This might suggest that iron and lipid peroxidation are involved in the pathogenesis of COVID-19. However, the mechanism of cell death caused by ferroptosis for patients with COVID-19 remains unclear. Given that *IFI27* was significantly increased in all patients with COVID-19 and *FOS* was dramatically decreased in recovered patients, these two markers might be used for disease condition prediction in the future.

TCR and BCR profiling holds great potential not only for understanding the development mechanisms of the normal immune response but also for providing insight into disease mechanisms and the development of new therapies for infectious diseases.^48^ In this study, clonotypic T cell expansion was reduced during disease recovery, while clonotypic B cell expansion rose simultaneously, suggesting the acute effective immune response goes down after virus removal and antibodies will likely stay for quite a long time to protect the host from reinfection. Virus-specific memory T cell have been shown to persist for many years after infection.^49^ SARS-CoV-2-specific memory T cell responses can directed against the internal (nucleocapsid) and surface proteins (membrane and/or spike) in case of lacking antibodies.^50^ It is also found that mild cases have the higher proportions of SARS-CoV-2-specific CD8+ memory T cells than severe cases.^51^ Patients with COVID-19 showed different degrees of specific memory T clonotypic cell expansion or preservation, which may play a role in the case of another elimination of SARS-COV-2. However, because the observation time after SARS-CoV-2 infection in our study is not long enough, whether memory T cells have a protective effect for SARS-CoV-2 reinfection still needs more exploration. Antibodies produced by B cells are considered to be effective against SARS-CoV-2 because convalescent serum samples have been applied with supposedly good clinical results in COVID-19 cases ^48^ and were also previously used successfully in the treatment of SARS.^52^ We found that COVID-19 patients all have a group of plasmablasts in their peripheral blood, as characterized by a high level of *CD27*, *IgM*, *IgG*, and *IgA* transcripts. Moreover, their memory B cells also express *IgM*, *IgG*, and *IgA* transcripts, suggesting that these patients had developed antiviral immune memory. All these patients have highly mutated and clonally related IgH genes, indicating that a strong antibody response occurred during SARS-CoV-2 infection. However, we should note the unwanted side effects that can occur when an antibody is used as a therapy.^32^

Our study has some limitations, including having limited and heterogeneous patients; in particular, only one patient with severe COVID-19 was included. The effects of age and immunoregulatory therapies during the patients’ recovery could not be fully assessed. Although there is a certain correlation between the IFN and MAPK pathways and the antiviral function of immune cells in the literature, their relationship in SARS-CoV-2-infected patients is needed to be confirmed further. Besides, the B cell antibody sequences could not be fully captured due to the limited sample size and sequencing depth in this study.

## Supplementary information

Supplementary file

## Data Availability

All sequencing data are available. Please contact the corresponding author Zhenglin Yang for the raw data (zliny@yahoo.com).

## References

[1] Duffy MR (2009). Zika virus outbreak on Yap Island, Federated States of Micronesia. N. Engl. J. Med..

[2] Wu YP, Wei R, de Groot PG (2003). SARS in Hong Kong. N. Engl. J. Med..

[3] Baize S (2005). A single shot against Ebola and Marburg virus. Nat. Med..

[4] Zhu N (2020). A novel coronavirus from patients with pneumonia in China, 2019. N. Engl. J. Med..

[5] Li Q (2020). Early transmission dynamics in Wuhan, China, of novel coronavirus-infected pneumonia. N. Engl. J. Med..

[6] Wang D (2020). Clinical characteristics of 138 hospitalized patients with 2019 novel coronavirus-infected pneumonia in Wuhan, China. JAMA.

[7] Phelan AL, Katz R, Gostin LO (2020). The novel coronavirus originating in Wuhan, China: challenges for Global Health Governance. JAMA.

[8] Cao W, Li T (2020). COVID-19: towards understanding of pathogenesis. Cell Res..

[9] Thomas PG, Handel A, Doherty PC, La Gruta NL (2013). Ecological analysis of antigen-specific CTL repertoires defines the relationship between naive and immune T-cell populations. Proc. Natl Acad. Sci. USA.

[10] Godthelp BC, van Tol MJ, Vossen JM, van Den Elsen PJ (1999). T-Cell immune reconstitution in pediatric leukemia patients after allogeneic bone marrow transplantation with T-cell-depleted or unmanipulated grafts: evaluation of overall and antigen-specific T-cell repertoires. Blood.

[11] Zhong L (2020). Detection of serum IgM and IgG for COVID-19 diagnosis. Sci. China Life Sci..

[12] Ding Q (2020). The clinical characteristics of pneumonia patients coinfected with 2019 novel coronavirus and influenza virus in Wuhan, China. J. Med. Virol..

[13] Wang F (2020). Characteristics of peripheral lymphocyte subset alteration in COVID-19 pneumonia. J. Infect. Dis..

[14] Wen W (2020). Immune cell profiling of COVID-19 patients in the recovery stage by single-cell sequencing. Cell Discov..

[15] Liao M (2020). Single-cell landscape of bronchoalveolar immune cells in patients with COVID-19. Nat. Med..

[16] Gu Y (1992). The (4;11)(q21;q23) chromosome translocations in acute leukemias involve the VDJ recombinase. Proc. Natl Acad. Sci. USA.

[17] Kamel OW (1995). Clonal VDJ recombination of the immunoglobulin heavy chain gene by PCR in classical Hodgkin’s disease. Am. J. Clin. Pathol..

[18] Ferguson A, Chen K (2020). Analysis of transcriptional profiling of immune cells at the single-cell level. Methods Mol. Biol..

[19] Okoye AA, Picker LJ (2013). CD4(+) T-cell depletion in HIV infection: mechanisms of immunological failure. Immunol. Rev..

[20] Szklarczyk D (2019). STRING v11: protein-protein association networks with increased coverage, supporting functional discovery in genome-wide experimental datasets. Nucleic Acids Res..

[21] von Mering C (2007). STRING 7–recent developments in the integration and prediction of protein interactions. Nucleic Acids Res..

[22] von Mering C (2003). STRING: a database of predicted functional associations between proteins. Nucleic Acids Res..

[23] Gordon J (1986). Control of human B-lymphocyte replication. II. Transforming Epstein-Barr virus exploits three distinct viral signals to undermine three separate control points in B-cell growth. Immunology.

[24] Golub R, Fellah JS, Charlemagne J (1997). Structure and diversity of the heavy chain VDJ junctions in the developing Mexican axolotl. Immunogenetics.

[25] Gonzalez D (2007). Immunoglobulin gene rearrangements and the pathogenesis of multiple myeloma. Blood.

[26] Tang, B. M. et al. A novel immune biomarker IFI27 discriminates between influenza and bacteria in patients with suspected respiratory infection. *Eur. Respir. J*. **49**, 1–12 (2017). 10.1183/13993003.02098-2016 28619954

[27] Zhang G (2020). Clinical features and short-term outcomes of 221 patients with COVID-19 in Wuhan, China. J. Clin. Virol..

[28] Xia XY (2020). Epidemiological and initial clinical characteristics of patients with family aggregation of COVID-19. J. Clin. Virol..

[29] Wei, X. S. et al. Diarrhea is associated with prolonged symptoms and viral carriage in corona virus disease 2019. *Clin. Gastroenterol. Hepatol*. **18**, 1753–1759 e1752 (2020). 10.1016/j.cgh.2020.04.030 PMC7165091 32311512

[30] Deng Y (2020). Clinical characteristics of fatal and recovered cases of coronavirus disease 2019 in Wuhan, China: a retrospective study. China Med. J. (Engl.).

[31] Falchetti R (1998). Determination of cytokine co-expression in individual splenic CD4+ and CD8+ T cells from influenza virus-immune mice. Immunology.

[32] Tay MZ (2020). The trinity of COVID-19: immunity, inflammation and intervention. Nat. Rev. Immunol..

[33] Hakeem L (2010). Prevalence and immunization status of Hepatitis B virus in the HIV cohort in Fife, Scotland. J. Clin. Med. Res..

[34] Au WC, Su Y, Raj NB, Pitha PM (1993). Virus-mediated induction of interferon A gene requires cooperation between multiple binding factors in the interferon alpha promoter region. J. Biol. Chem..

[35] Rang A, Gunther S, Will H (1999). Effect of interferon alpha on hepatitis B virus replication and gene expression in transiently transfected human hepatoma cells. J. Hepatol..

[36] de Lang A, Osterhaus AD, Haagmans BL (2006). Interferon-gamma and interleukin-4 downregulate expression of the SARS coronavirus receptor ACE2 in Vero E6 cells. Virology.

[37] Leela SL (2016). Drug repurposing of minocycline against dengue virus infection. Biochem. Biophys. Res. Commun..

[38] Manley, G. C. A., Parker, L. C. & Zhang, Y. Emerging regulatory roles of dual-specificity phosphatases in inflammatory airway disease. *Int. J. Mol. Sci*. **20**, 1–23 (2019). 10.3390/ijms20030678 PMC6387402 30764493

[39] Ziegler CGK (2020). SARS-CoV-2 receptor ACE2 is an interferon-stimulated gene in human airway epithelial cells and is detected in specific cell subsets across tissues. Cell.

[40] Assiri A (2013). Epidemiological, demographic, and clinical characteristics of 47 cases of Middle East respiratory syndrome coronavirus disease from Saudi Arabia: a descriptive study. Lancet Infect. Dis..

[41] Yin Y, Wunderink RG (2018). MERS, SARS and other coronaviruses as causes of pneumonia. Respirology.

[42] Peiris JS (2003). Clinical progression and viral load in a community outbreak of coronavirus-associated SARS pneumonia: a prospective study. Lancet.

[43] Booth CM (2003). Clinical features and short-term outcomes of 144 patients with SARS in the greater Toronto area. JAMA.

[44] Arabi YM (2014). Clinical course and outcomes of critically ill patients with Middle East respiratory syndrome coronavirus infection. Ann. Intern. Med..

[45] Lau SKP (2013). Delayed induction of proinflammatory cytokines and suppression of innate antiviral response by the novel Middle East respiratory syndrome coronavirus: implications for pathogenesis and treatment. J. Gen. Virol..

[46] Shu T (2020). Plasma proteomics identify biomarkers and pathogenesis of COVID-19. Immunity.

[47] Bayir H (2020). Achieving life through death: redox biology of lipid peroxidation in ferroptosis. Cell Chem. Biol..

[48] Calis JJ, Rosenberg BR (2014). Characterizing immune repertoires by high throughput sequencing: strategies and applications. Trends Immunol..

[49] Tang F (2011). Lack of peripheral memory B cell responses in recovered patients with severe acute respiratory syndrome: a six-year follow-up study. J. Immunol..

[50] Sekine T (2020). Robust T cell immunity in convalescent individuals with asymptomatic or mild COVID-19. Cell.

[51] Peng Y (2020). Broad and strong memory CD4(+) and CD8(+) T cells induced by SARS-CoV-2 in UK convalescent individuals following COVID-19. Nat. Immunol..

[52] Cheng Y (2005). Use of convalescent plasma therapy in SARS patients in Hong Kong. Eur. J. Clin. Microbiol. Infect. Dis..

