# Supplementary material for: Dynamic blood single-cell immune responses in patients with COVID-19
Source: Signal Transduct Target Ther. 2021 Mar 6;6:110. doi: 10.1038/s41392-021-00526-2 (PMC7936231; doi:10.1038/s41392-021-00526-2)
Supplement: Supplementary file 1 — Supplementary file [file 41392_2021_526_MOESM1_ESM.docx]

Supplementary Materials for

**Dynamic blood single cell immune responses in patients with COVID-19**

Lulin Huang^1,2,3*^, Yi Shi^1,2,3*^, Bo Gong^1,2,3*^, Li Jiang^1,2*^, Zhixin Zhang^4^, Xiaoqi Liu^1,2,3^, Jialiang Yang^1,2^, Yongquan He^1^, Zhilin Jiang^1^, Ling Zhong^1^, Juan Tang^5^, Chunfang You^5^, Qi Jiang^6^, Bo Long^6^, Tao Zeng^7^, Mei Luo^7^, Fanwei Zeng^8^, Fanxin Zeng^8^, Shuqiang Wang^9^, Xingxiang Yang^9^, Zhenglin Yang^1,2,3, #^

Correspondence to: zliny@yahoo.com

**This PDF file includes:**

Materials and Methods

Figures S1 to S32

Tables S1 to S9

Materials and Methods

Sample collection

For single-cell V(D)J analysis, 16 patients with COVID-19 were included in the current study, and the basic information of the included samples is shown in Table S1. All 16 patients were recruited from the Sichuan Province. Among the COVID-19 patients, their onset time was January 2020, and their age was between 19 and 62 years old, including six males and six females. The samples for the first-round analysis included two critical cases (patient 1 and patient 2), one severe case (patient 3), six moderate cases (patients 4–9), three mild case (patient 10, patient 11 and patient 12), and four cured patients (patient 13 to patient 16). CT images of the lungs of these patients are shown in Fig. S1. The study also includes three normal healthy people (NC-1, NC-2, and NC-3). For the longitudinal study, specifically for V(D)J analysis, nine samples from seven patients have collected again when their disease state improved or was cured, including patient 1, patient 2, patient 3, patient 6, patient 7, patient 9, and patient 10.

Human blood sample PBMCs isolation

All experiments using human samples were approved by the Ethics Committee of Sichuan Provincial People’s Hospital (approval no. 2020 (95)). Informed consent was obtained from all donors. Fresh whole blood from COVID-19 patients and unrelated control donors was collected in 2 mL tubes containing ethylene diamine tetraacetic acid (EDTA). PBMCs were isolated from whole blood within 8 h of sample collection by using 15 ml tubes with a lymphocyte cell separation media (CEDARLANE) according to the manufacturer’s instructions. CEDARLANE’s Lympholyte® Cell Separation density gradient centrifugation media (Lot#25-127) has been specifically designed for the isolation of viable lymphocytes from human peripheral blood. Briefly, each blood sample was diluted with an equal volume of 1X PBS (pH7.4) added into a 15 ml tube. Gently layer the cell suspension or whole blood over top of the Lympholyte® making sure not to mix the two layers and centrifuged at 800 × g for 20 min at room temperature. After centrifugation, carefully remove the cells at the interface using a pasteur pipette and transfer into a new centrifuge tube for washing. Enriched cells were washed with PBS and centrifuged at 800 × g for 10 min twice. Cell numbers and viability were measured using a Countess II Automated Cell Counter (Thermo Fisher Scientific).

Chromium Single-Cell V(D)J Libraries preparation and sequencing

Chromium Single-Cell V(D)J libraries were prepared according to the instructions. Briefly, GEMs were generated by combining barcoded Single-Cell VDJ 5' Gel Beads v1.1, a master mix with cells, and partitioning oil on Chromium Next GEM Chip G. Immediately following GEM generation, the gel bead was dissolved, and any partitioned cell was lysed. Incubation of the GEMs produced 10x barcoded, full-length cDNA from polyadenylated mRNA. GEMs were broken and pooled after GEM-RT reaction mixtures were recovered. Silane magnetic beads were used to purify the 10x barcoded first-strand cDNA from the post-GEM-RT reaction mixture, which includes leftover biochemical reagents and primers. After cleanup, a user can pursue target enrichment directly from the first-strand cDNA, in which case we consulted the demonstrated protocol of the Chromium Single-Cell V(D)J Reagent Kits-Direct Target Enrichment. Amplification generates sufficient material to construct multiple libraries from the same cells, for example, both T cell– and/or B cell-enriched libraries and 5' gene expression libraries. Qubit was used for library quantification before pooling. The final library pool was sequenced on the Illumina novaseq instrument using 150 base-pair paired-end reads. The technical features of each sample are listed in Table S9.

Single-cell data processing

Analysis pipelines in Cell Ranger version 3.0.2 were used for sequencing data processing. TCR/BCR data were processed by running Cell Ranger vdj with reference = refdata-cellranger-vdj-GRCh38-alts-ensembl-2.0.0 to assemble TCR/BCR chains and determine clonotypes. Transcriptome data were processed by running Cell Ranger count with transcriptome = refdata-cellranger-GRCh38-1.2.0.

Comprehensive integration of single-cell data

The output of Cell Ranger aggr, was loaded into R by using Seurat v3 for a modified integration analysis to remove batch effects and clustering. Recent approaches have established the use of canonical correlation analysis (CCA),^53^ alongside independent pioneering work leveraging the identification of mutual nearest neighbors (MNNs),^54^ to identify shared subpopulations across datasets. Based on CCA and MNNs, a computational strategy to “anchor” diverse datasets together was developed, enabling us to integrate and compare single-cell measurements across scRNA-seq technologies to remove batch effects.^55^ The Seurat v3 anchoring procedure is designed to integrate diverse single-cell datasets across technologies and modalities. To facilitate the assembly of datasets into an integrated reference, Seurat returns a corrected data matrix for all datasets, enabling them to be analyzed jointly in a single workflow. To transfer information from a reference to a query dataset, Seurat does not modify the underlying expression data, instead of projecting either discrete labels or continuous data across experiments. Although each sampling experiment has different capture cells, the underlying methods are conserved across samples. The Seurat v3 anchoring procedure consists of four broad steps: (1) data preprocessing and feature selection, (2) dimension reduction and identification of “anchor” correspondences between datasets, (3) filtering, scoring, and weighting of anchor correspondences, and (4) data matrix correction or data transfer across experiments. A reciprocal principal component analysis (PCA) is used in place of a CCA for the dimension reduction used in anchor finding. Most other parameters used to refer to the Seurat v3 integration analysis workflow. About 2,000 genes with an average expression of more than 0.01 and a dispersion greater than 0.5 were used as inputs for the initial PCA, and the number of principal components (PC) used for the nonlinear dimensional reduction (t-SNE) analysis was chosen based on the PCElbowPlot function and JackStrawPlot function. We set a 0.9 or 1.5 resolution parameter in the FindAllCluster function. The bimod likelihood ratio statistical test of Seurat V3 was used to screen the specific differential expression genes of different cell groups, which met the requirements of p-value < = 0.05 and more than two times differential expression range to screen the DEs between the designated cell group and other cell groups. The screening basis of the cell group for specific expression genes is significantly up-regulated expression genes belonging to this cell group, not the up-regulated expression genes belonging to other cell groups. The thermogram only shows how the top 50 genes rank in terms of the average expression amount of this cell group compared with the difference amplitude of other cell groups. The TSNE coordinate information of projection_TSNE_SC.csv—or the UMAP coordinate information of projection_UMAP_SC.csv—and the clustering category information of clusters_SC.csv can be imported into cloupe viewer to use the Loupe Cell Browser for visualization.

Next, DE genes in each comparison group of samples (e.g., between the cells from patients with COVID-19 and the cells from all healthy controls) were calculated by FindMarkers(). The average log (fold change) of each DE gene (FDR<0.05) that was calculated by FindMarkers() was supplied to further the STRING network analysis. For cell subpopulation identification, besides basic markers for PBMC, SingleR package (https://github.com/dviraran/SingleR) is also used.

For the cell proportion comparison analysis in Fig. 1f and 1g, the cell proportion of each cell type in each sample were calculated. Then, we compared the proportion of each cell type between the cases and controls (for the control samples, the mean proportion of three control samples was used for comparing).

Analysis of interaction network pathways of DE genes

The protein interaction network of this species was queried and downloaded from the STRING protein interaction network database using proteins with values for the functional enrichment analysis. Because of the large number of DE genes between each comparison group, the differential expressed genes with FDR<0.05 were selected to draw the protein interaction network map. Local STRING network cluster results were used as the STRING network pathway. The DE gene lists were used for the KEGG pathway analysis.

Integration analysis with TCR/BCR clones

Full-length TCR/BCR V(D)J segments were enriched from amplified cDNA from 5' libraries via PCR amplification using a Chromium Single-Cell V(D)J Enrichment kit according to the manufacturer’s protocol (10x Genomics). The TCR/BCR sequences for every single T/B cell were assembled by a Cell Ranger vdj pipeline (v3.1.0), leading to the identification of a CDR3 sequence and the rearranged TCR/BCR gene. Integration analysis with T/B clonotypic cells clones was done using the Chromium Single-Cell Immune Profiling Software Suite, which is a complete package for assembling, analyzing, and visualizing V(D)J sequences and clonotypes, gene expression profiles, and feature barcoding technology that enables cell surface protein expression. The package includes Cell Ranger Analysis Pipelines, Loupe V(D)J Browser, and Loupe Cell Browser visualization tools. The FASTQs were generated with Cell Ranger mkfastq and by running Cell Ranger vdj in V(D)J T Cell and B Cell Analysis. According to the clone type obtained by TCR or BCR sequencing, TCR/BCR sequencing corresponds to the cell marker sequence sequenced by 5-terminal transcripts one by one, and the cells without clone type were filtered. Based on the binomial distribution test, the clone types satisfying a p-value < = 0.05 were screened for significant differences in cell types. The different clone types are shown in a UMAP map.

Real-time PCR analysis

RNA was extracted from patients’ PBMC. The following primers were designed for amplification genes: GAPDH was used as the internal reference. Applied Biosystems SYBR Green was used for the real-time PCR analysis in a 7500 Fast Instrument (Applied Biosystems) according to the instructions.

Immunofluorescent staining

PBMC smears were used for immunofluorescent staining. We used 1× PBS to wash the smears three times. The tissue sections were then subjected to blocking using a blocking buffer (5% normal donkey serum, 0.3% TritonTM X-100, and 1× PBS) for 1 h at room temperature. The sections were then incubated with primary antibody rabbit polyclonal anti-IFI27 (Cat#bs-15549R, BIOSS, USA; diluted 1:300) in a blocking buffer at 4°C overnight. Alexa 488-conjugated anti-rabbit secondary antibody (Molecular Probes, USA; diluted 1:500) was incubated for 2 h at room temperature. Nuclei were visualized by counterstaining with DAPI in a secondary antibody buffer. Finally, the tissues were washed with PBS, mounted, and allowed to cure overnight at 4°C. Images were visualized using Zeiss LSM 710 confocal microscopes.

IFN-α ELISA assay

The levels of IFN-α in serum/plasma were examined using commercially available ELISA kits according to the instruction (Cat#: MB0464A; Jiangsu enzymatic Biotechnology Co., Ltd, Jiangsu, China). This assay was able to detect interferon alfa 1, interferon alfa 2, interferon alfa 10, and interferon alfa-2b together. For sample collection and storages for serum, we used a serum separator tube and allowed the samples to clot for 30 minutes before centrifugation for 10 min at approximately 3000×g. Then, we removed the serum and stored the samples at -80°C. For the plasma, we collected plasma using EDTA or heparin as an anticoagulant. The samples were centrifuged for 30 min at 3000×g at 2-8°C within 30 min of collection and store samples at -80°C. We avoided repeated freeze-thaw cycles. The following is the procedure. 1) Add standard: set Standard wells, testing sample wells. Add standard 50μl to standard well. 2) Add Sample: Add testing sample 10μl then add Sample Diluent 40μl to testing sample well; Blank well doesn’t add anything. 3) Add 100μl of HRP-conjugate reagent to each well, cover with an adhesive strip and incubate for 60 minutes at 37°C. 4) Aspirate each well and wash, repeating the process four times for a total of five washes. Wash by filling each well with Wash Solution (400μl) using a squirt bottle, manifold dispenser or autowasher. Complete removal of liquid at each step is essential to good performance. After the last wash, remove any remaining Wash Solution by aspirating or decanting. Invert the plate and blot it against clean paper towels. 5) Add chromogen solution A 50μl and chromogen solution B 50μl to each well. Gently mix and incubate for 15 minutes at 37°C. Protect from light. 6) Add 50μl Stop Solution to each well. The color in the wells should change from blue to yellow. If the color in the wells is green or the color change does not appear uniform, gently tap the plate to ensure thorough mixing. 7) Read the Optical Density (O.D.) at 450 nm using a microtiter plate reader within 15 minutes. The calibration standards are assayed at the same time as the samples and allow the operator to produce a standard curve of O.D. versus IFN-α concentration. The concentration of IFN-α in the samples is then determined by comparing the O.D. of the samples to the standard curve.

Chemiluminescence IgG and IgM Method

Biotin was labelled with N and /or S protein, coating with streptavidin magnetic particles, to bind with the antibodies of IgG and IgM in patients’ serum. The sample is mixed with the magnetic particles coated with the recombinant antigen of the new coronavirus. Specific IgG and IgM antibodies in the sample are combined with the recombinant antigen to form an immune complex. After washing, acridine-labeled murine anti-human IgG and IgM antibodies were added, followed by adding the substrate solution, then a chemiluminescence reaction occurred. The value of the luminescence signal was measured by automatic chemiluminescent immunoassay (Maccura, i3000). The signal was positively correlated with the content of specific IgG and IgM antibodies in the sample.^11^

Statistical analysis

IFN-α concentration data were analyzed using GraphPad prism 5 (GraphPad Software, Inc., La Jolla, CA, USA) and expressed as the mean ± standard deviation (SD) from all independent samples. All statistical analyses between different comparison groups were performed through one way ANOVA test and Bonferroni’s post hoc test. P-values less than 0.05 were considered to be significantly different.

**References:**

53 Butler, A. *et al.* Integrating single-cell transcriptomic data across different conditions, technologies, and species. *Nat Biotechnol*. **36**, 411-420, (2018).

54 Haghverdi, L., Lun, A. T. L., Morgan, M. D. & Marioni, J. C. Batch effects in single-cell RNA-sequencing data are corrected by matching mutual nearest neighbors. *Nat Biotechnol*. **36**, 421-427, (2018).

55 Stuart, T. *et al.* Comprehensive Integration of Single-Cell Data. *Cell*. **177**, 1888-1902 e1821, (2019).


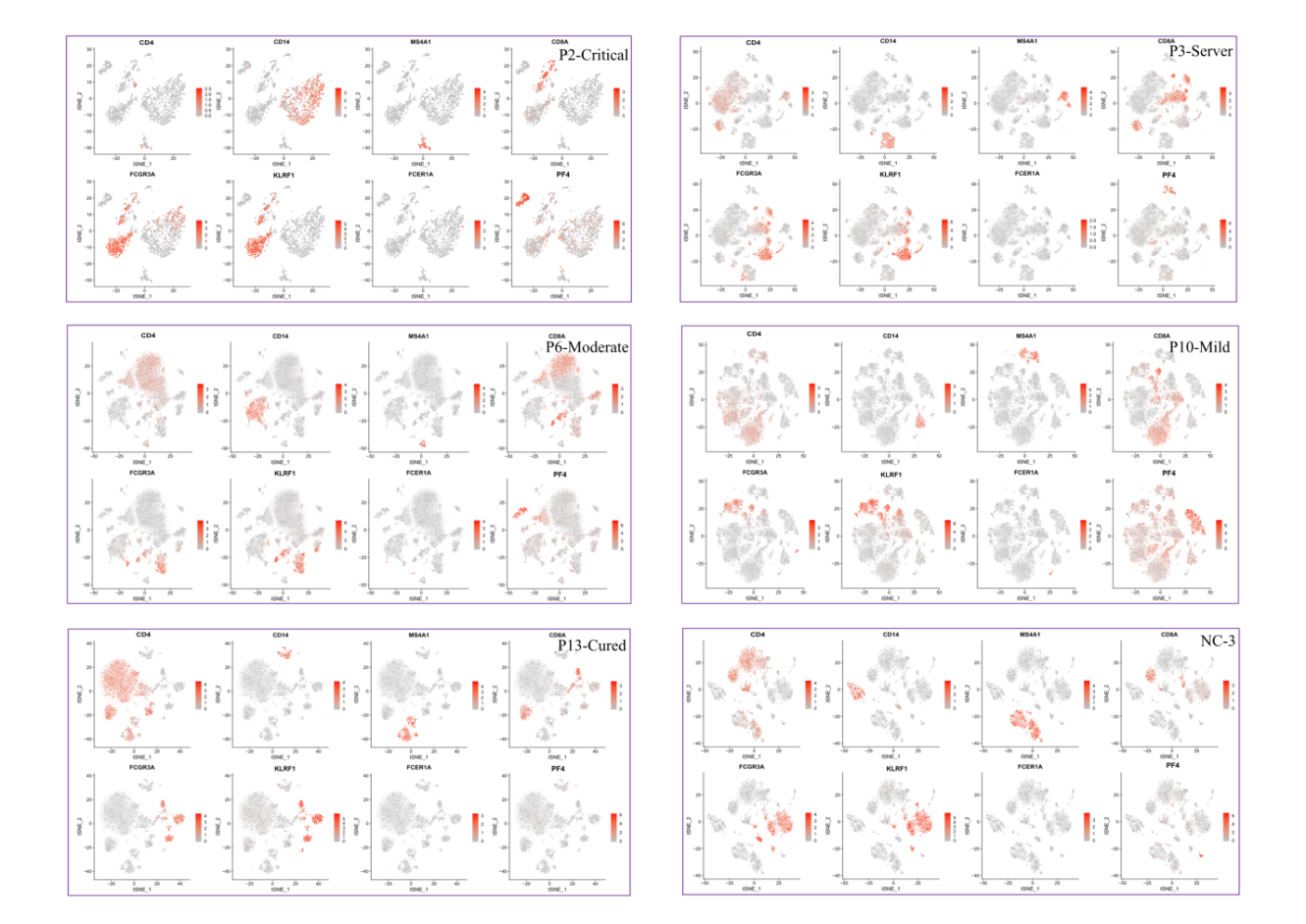


**Fig. S1 TSNE plots of the main cell marker genes in PBMC shown in six representative samples.** *CD4* (naïve CD4+ T), *CD14+* (CD14+ Monocyte), *MS4A1* (B cells), *CD8A* (CD8+ T), *FCGR3A* (FCGR3A+ Monocyte), *KLRF1* (NK), *FCER1A* (DC), and *PF4* (MP/platelets).


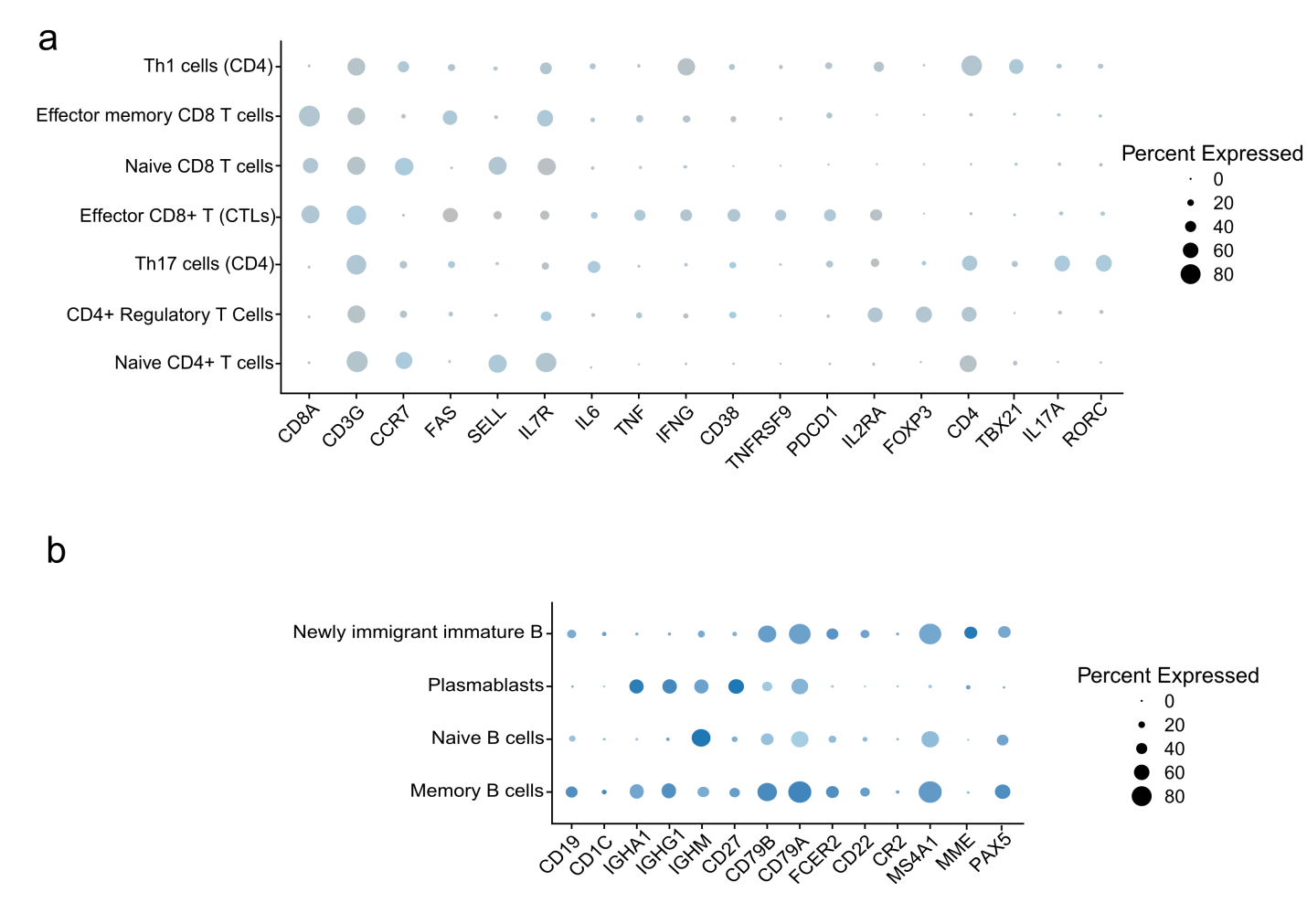


**Fig. S2 Dot plots of classic markers of T lymphocytes (a) and B lymphocytes (b)**.


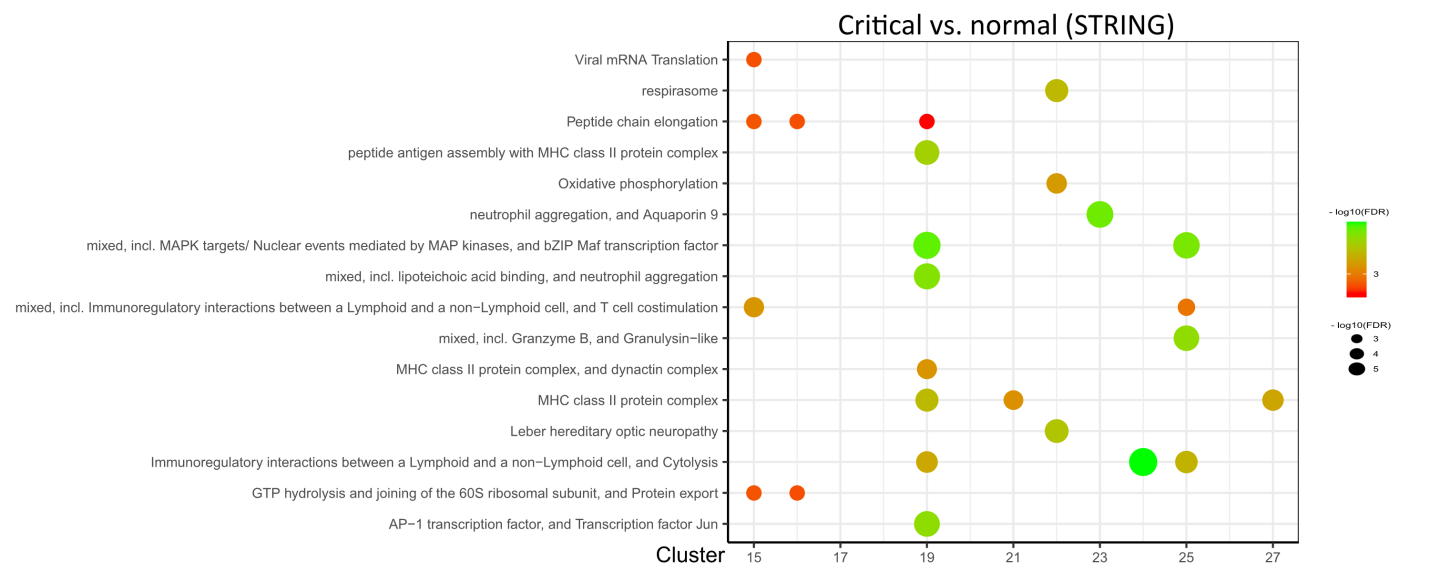


**Fig. S3 Enriched STRING pathways in critical condition patients with COVID-19 (P1 and P2) vs. normal controls.** STRING network pathways were analyzed by using DE genes and their corresponding log2 fold changes of the UMI counts between cases and controls.


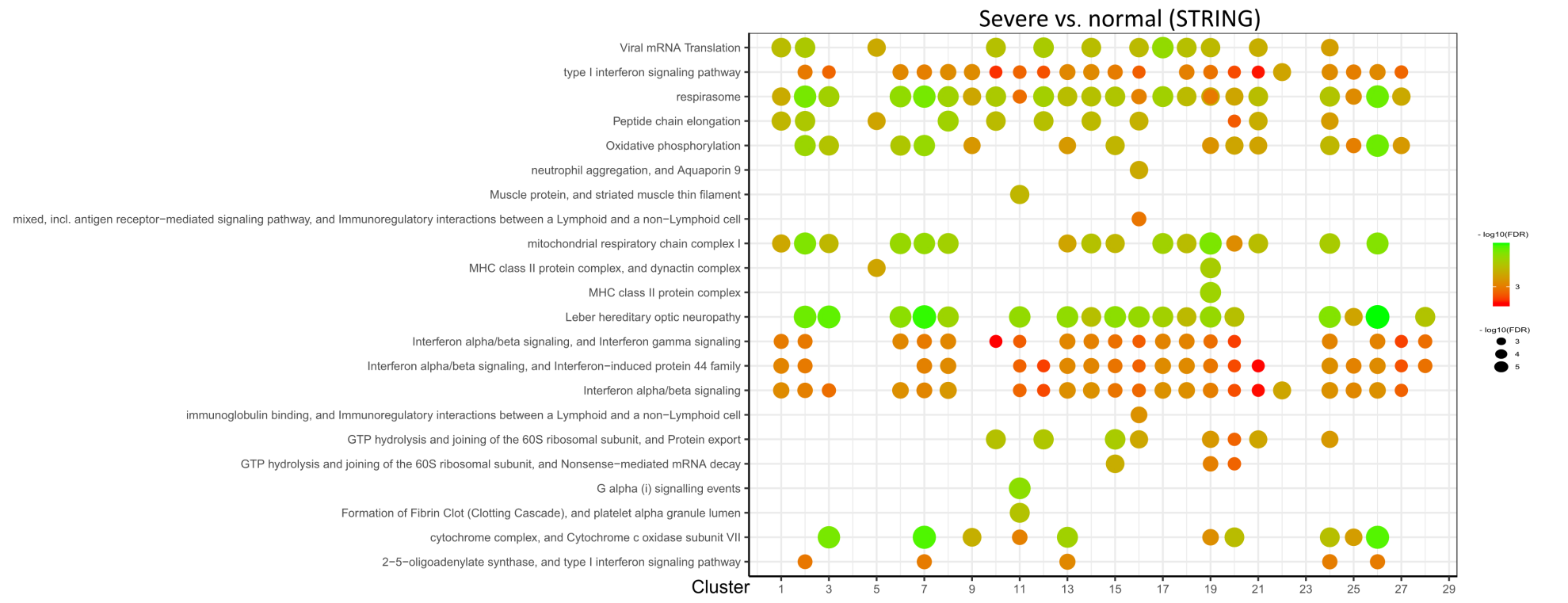


**Fig. S4 Enriched STRING pathways in server condition patient (P3) with COVID-19 vs. normal controls.** STRING network pathways were analyzed by using DE genes and their corresponding log2 fold changes of the UMI counts between cases and controls.

**
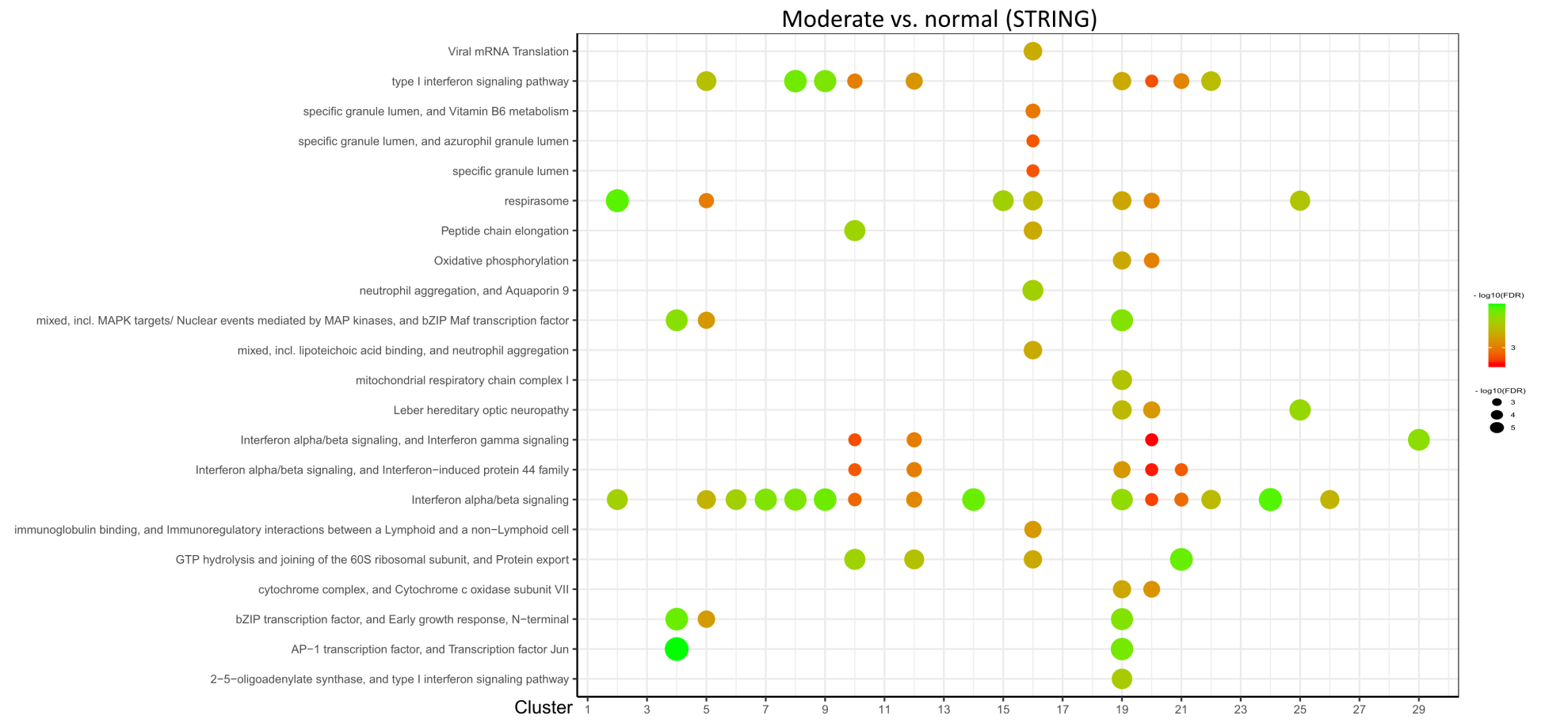
**

**Fig. S5 Enriched STRING pathways in moderate condition patients (P4-P9) with COVID-19 vs. normal controls.** STRING network pathways were analyzed by using DE genes and their corresponding log2 fold changes of the UMI counts between cases and controls.

**
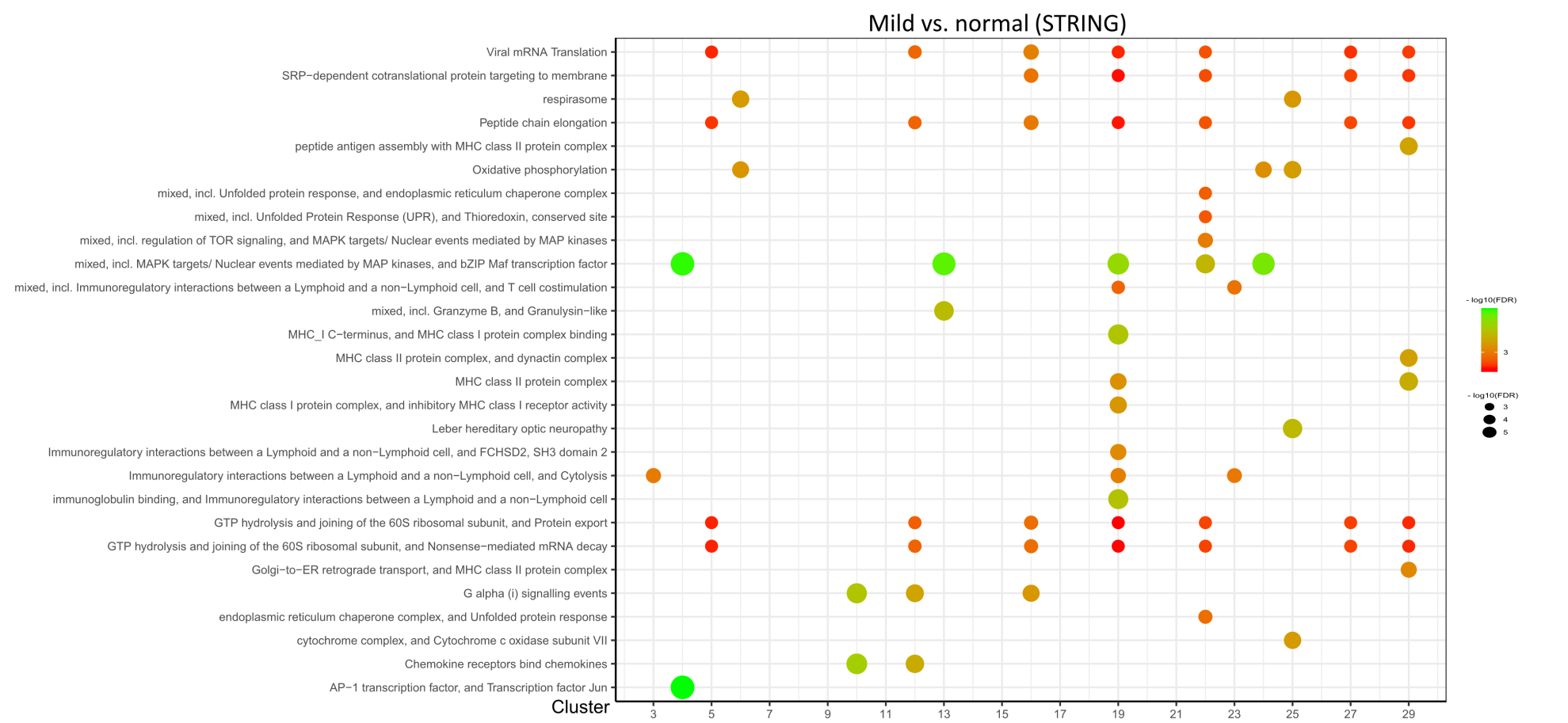
**

**Fig. S6 Enriched STRING pathways in mild condition patients (P10-P12) with COVID-19 vs. normal controls.** STRING network pathways were analyzed by using DE genes and their corresponding log2 fold changes of the UMI counts between cases and controls.


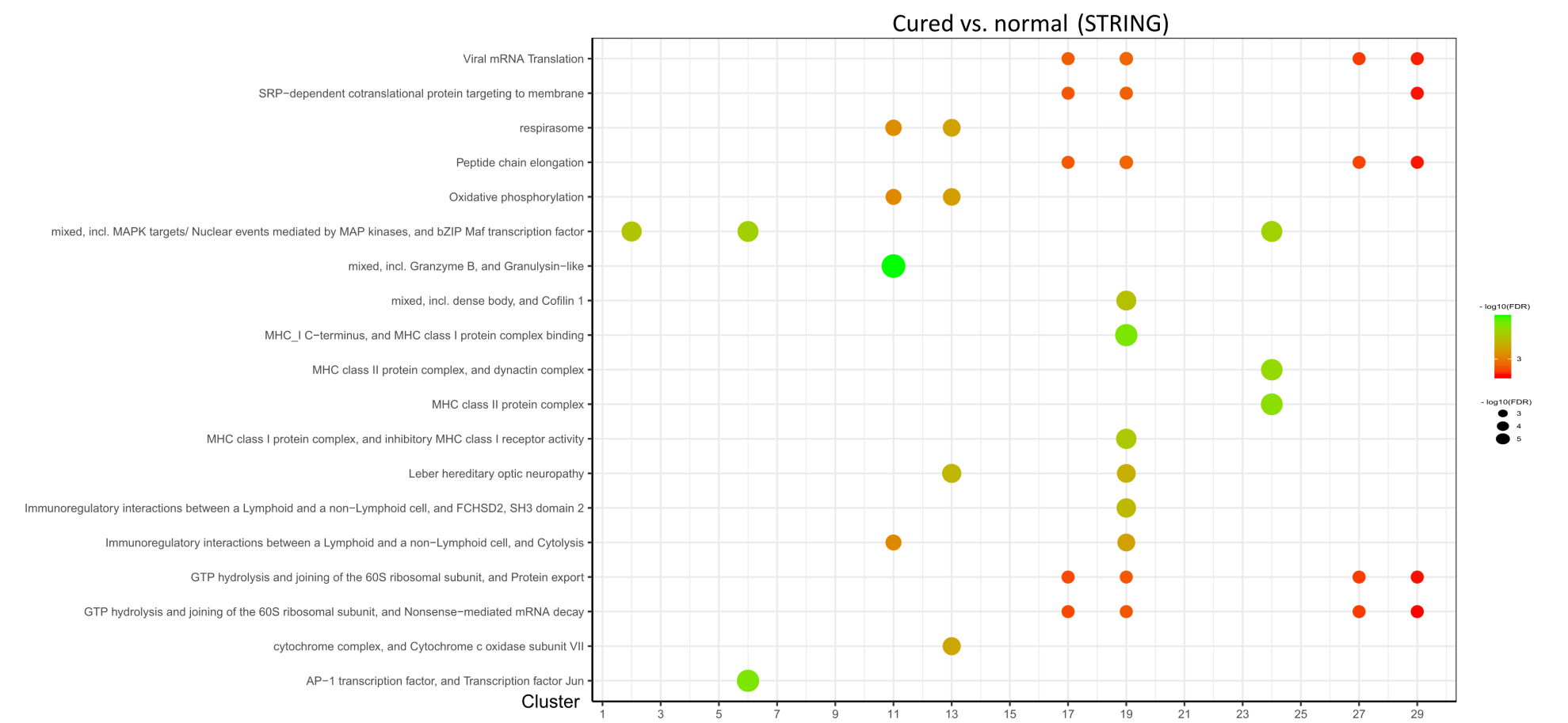


**Fig. S7 Enriched STRING pathways in cured condition patients with COVID-19 (P13-P16) vs. normal controls.** STRING network pathways were analyzed by using DE genes and their corresponding log2 fold changes of the UMI counts between cases and controls.


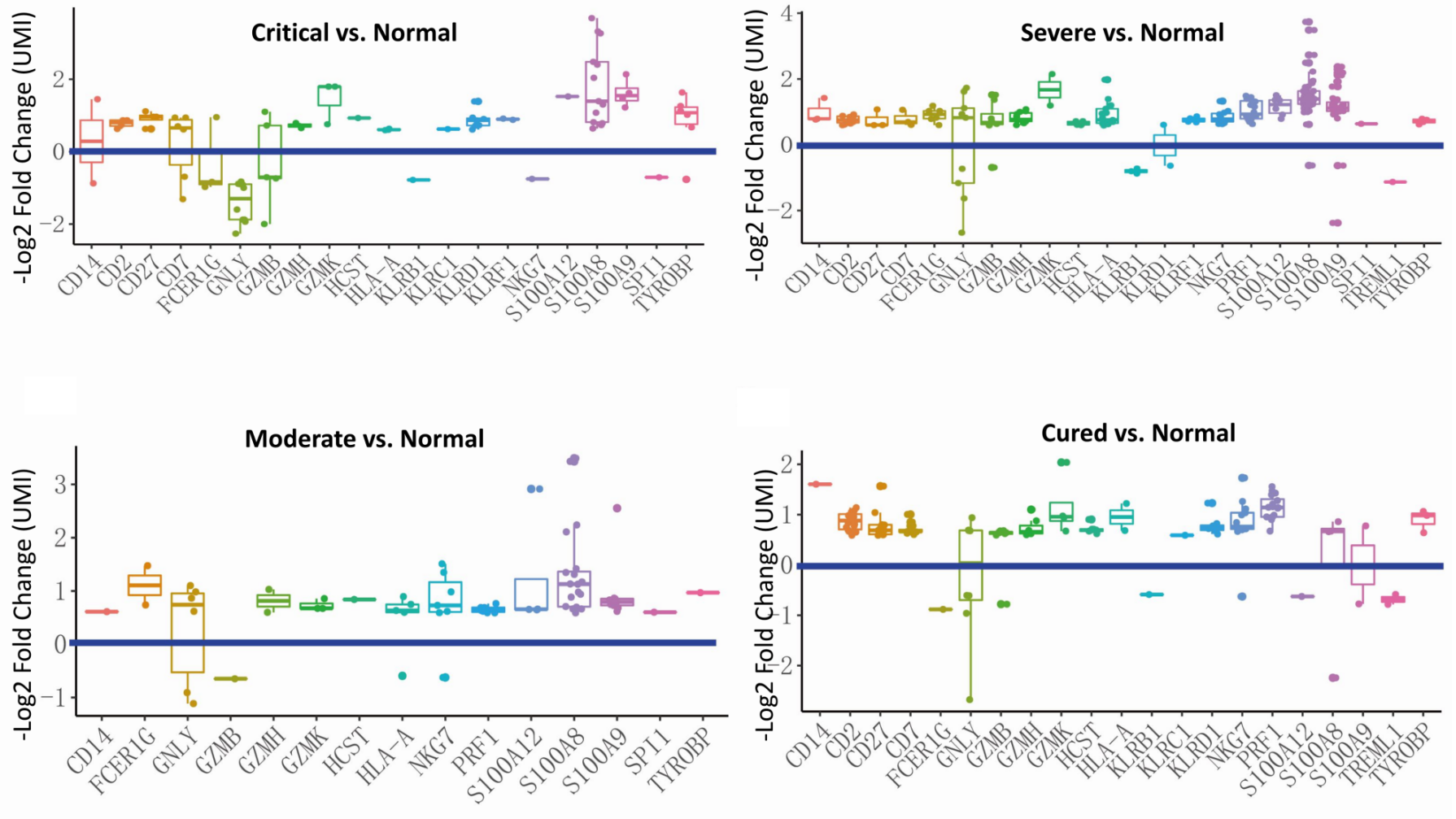


**Fig. 8 Gene expression changes of immunology interactions between lymphoid and nonlymphoid cells in response to SARS-CoV-2 infection.** Log2 fold changes of the unique molecular identifiers (UMI) counts of genes comparing patients in different conditions to normal controls. Each point represents a different cell subtype.

**
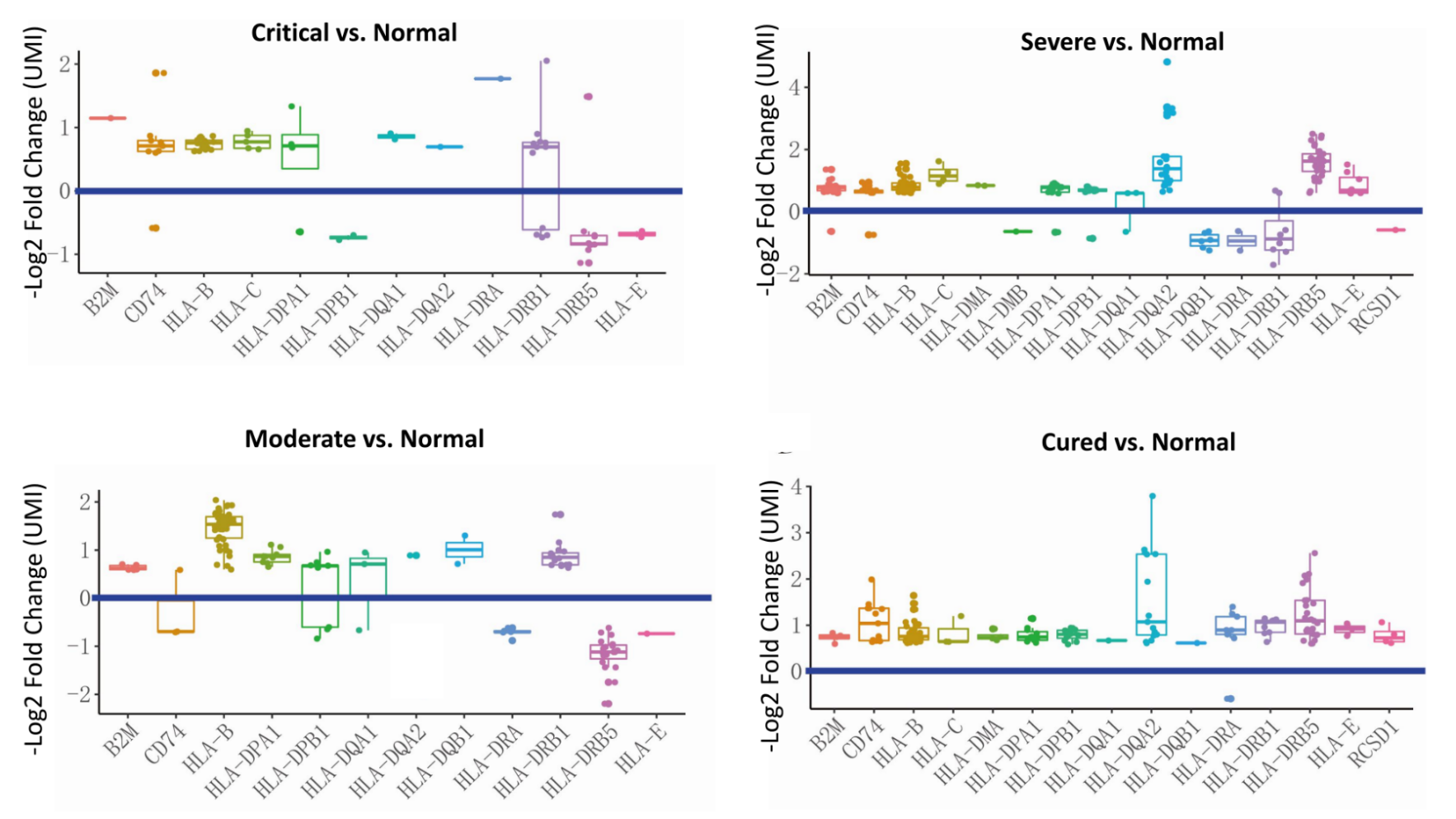
**

**Fig. 9 Gene expression changes of major histocompatibility complex (MHC) class II protein complex pathway in response to SARS-CoV-2 infection.** Log2 fold changes of the unique molecular identifiers (UMI) counts of genes comparing patients in different conditions to normal controls. Each point represents a different cell subtype.


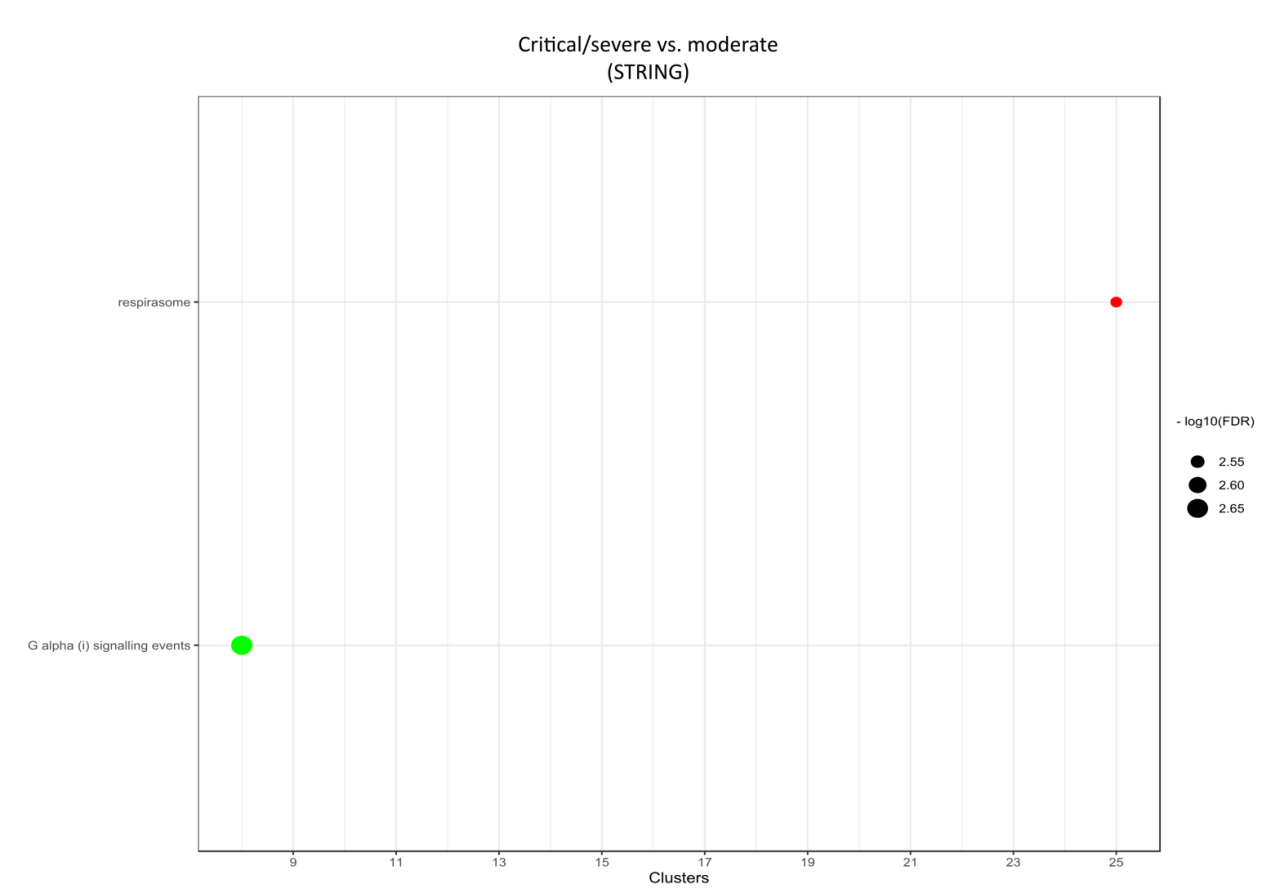


**Fig. S10 Enriched STRING pathways in critical/severe condition patients with COVID-19 vs. moderate condition patients with COVID-19.** STRING network pathways were analyzed by using DE genes and their corresponding log2 fold changes of the UMI counts between two comparing groups.

**
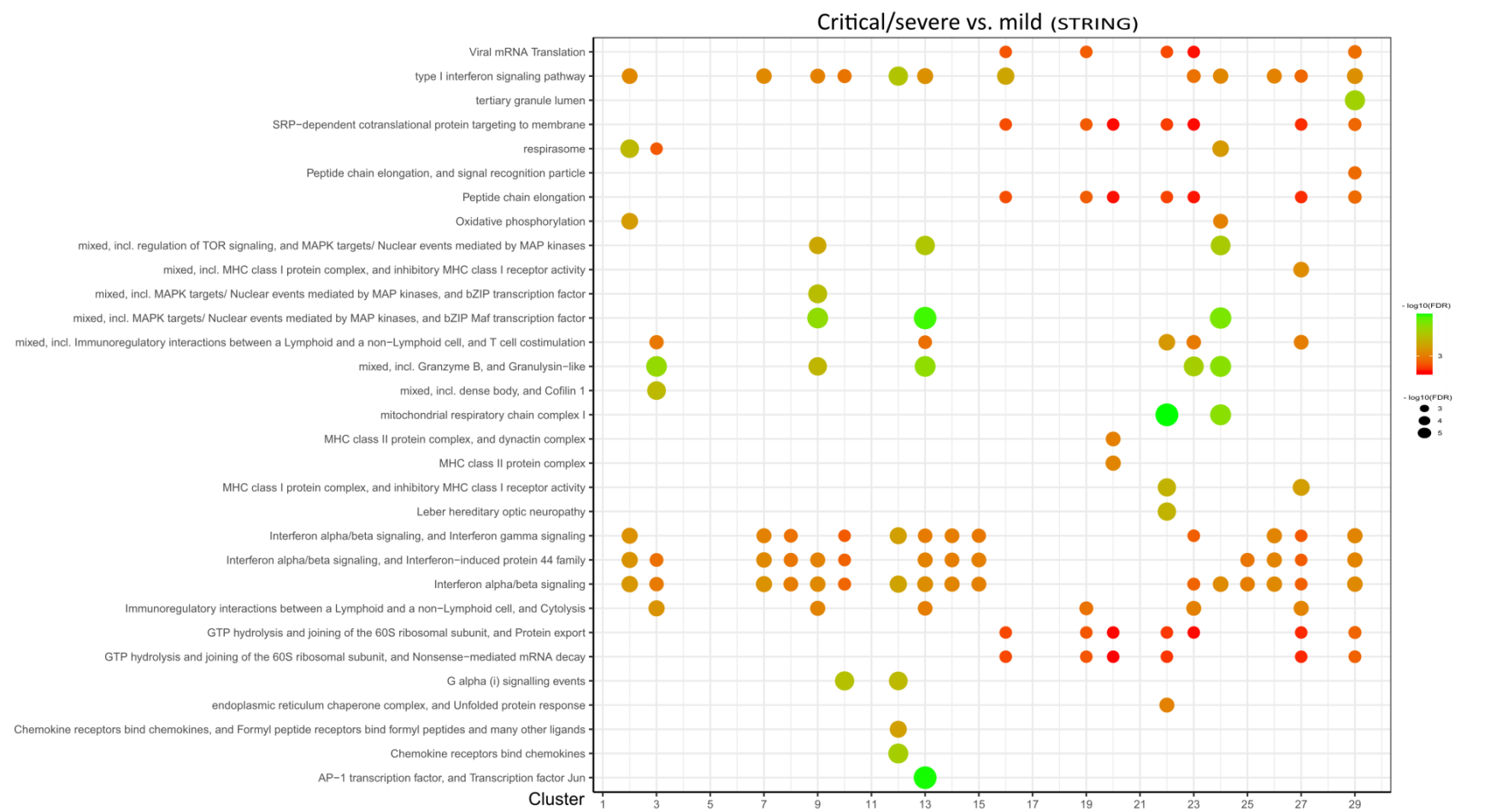
**

**Fig. S11 Enriched STRING pathways in critical/severe condition patients with COVID-19 vs. mild condition patients with COVID-19.** STRING network pathways were analyzed by using DE genes and their corresponding log2 fold changes of the UMI counts between two comparing groups.


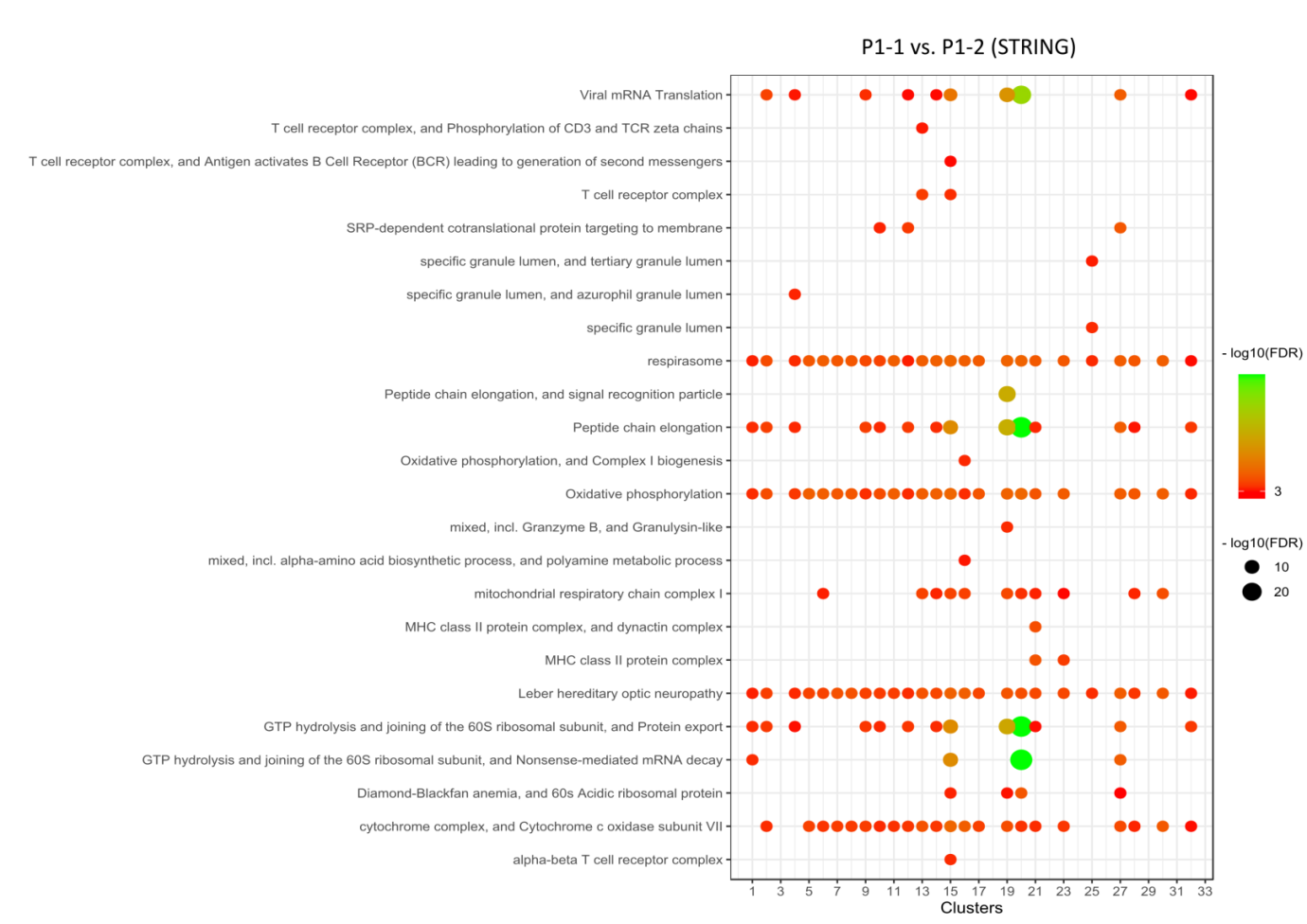


**Fig. S12 The enrichment of STRING network pathways of differential expressed (DE) genes in each cell subtype of P1-1 (in critical condition) vs. P1-2 (disease improvement).** The cell subtype is shown in Fig. 3a. STRING network pathways were analyzed by using DE genes and their corresponding log2 fold changes of the UMI counts between cases and controls.


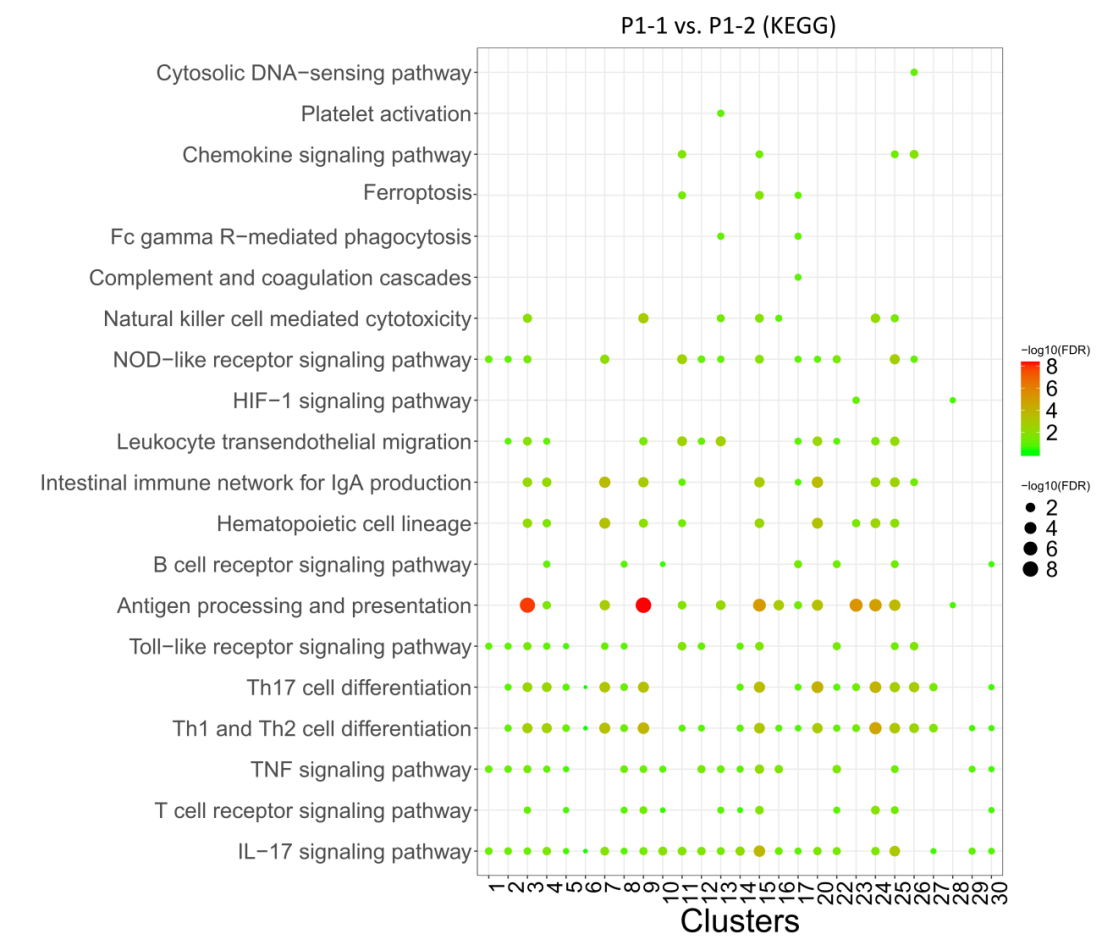


**Fig. S13 The enrichment of KEGG pathways of differential expressed (DE) genes in each cell subtype of P1-1 (in critical condition) vs. P1-2 (disease improvement).** KEGG pathways were analyzed by using DE genes lists between cases and controls. The cell subtype is shown in Fig. 3a.


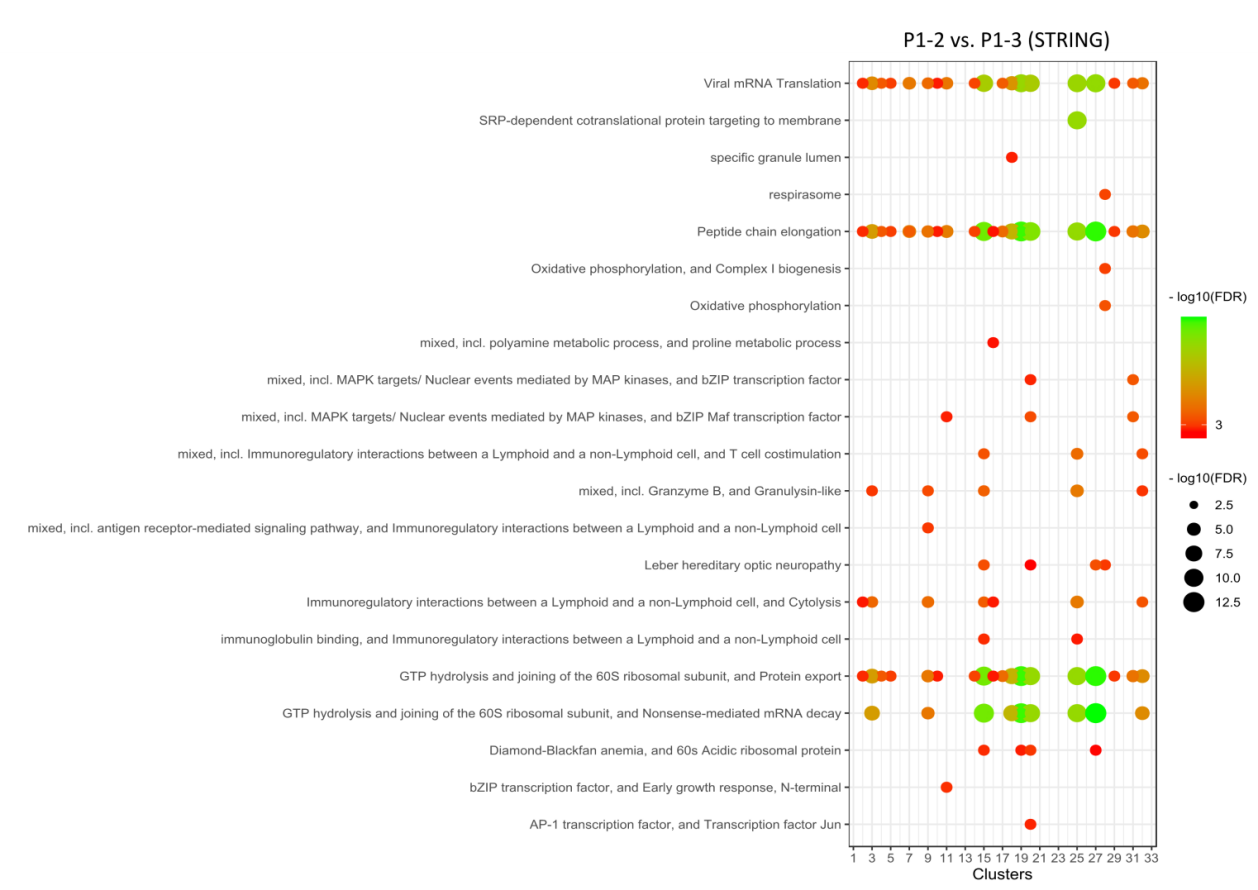


**Fig. S14 The enrichment of STRING network pathways of differential expressed (DE) genes in each cell subtype of P1-2 (disease improvement) vs. P1-3 (cured).**  The cell subtype is shown in Fig. 5a. STRING network pathways were analyzed by using DE genes and their corresponding log2 fold changes of the UMI counts between cases and controls.

**
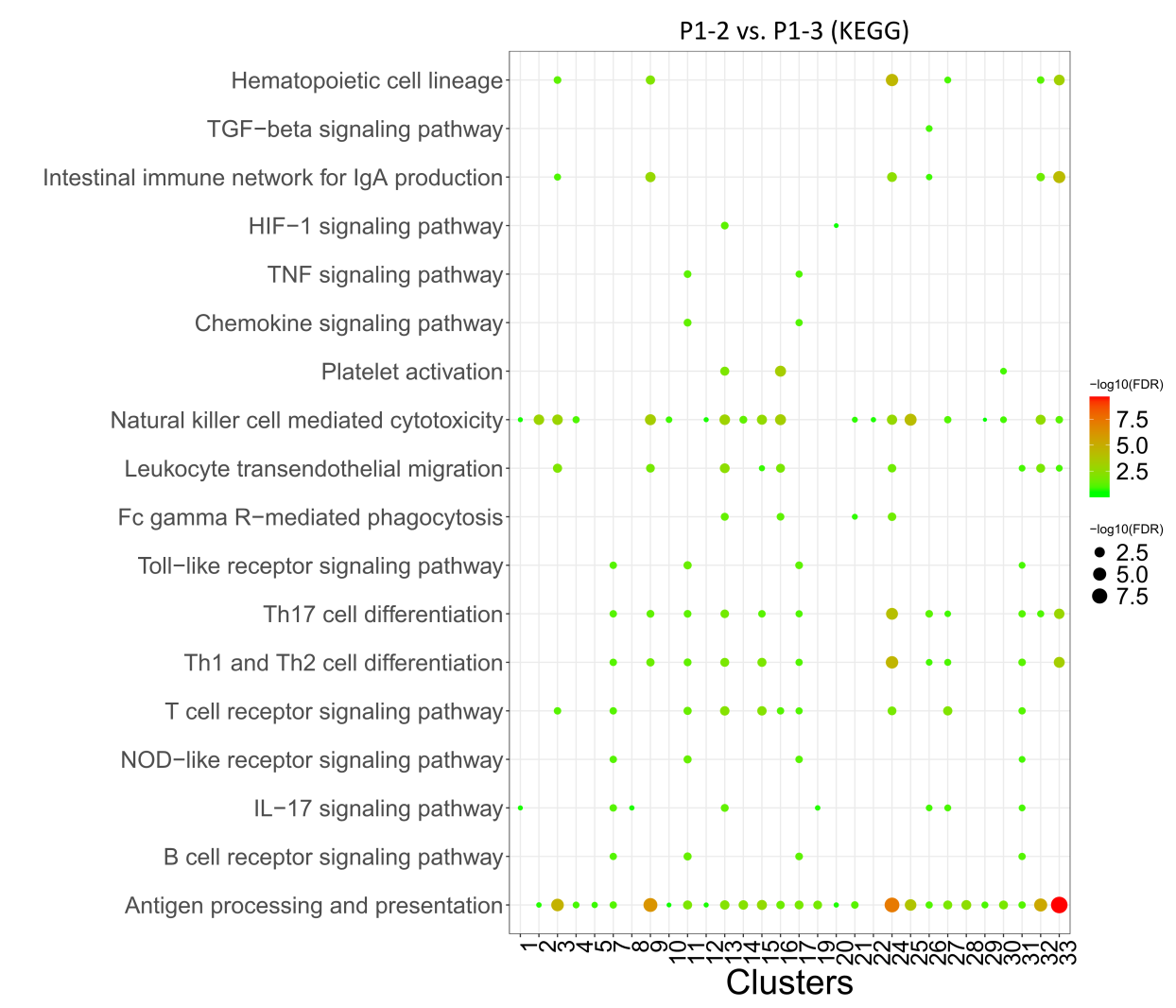
**

**Fig. S15 The enrichment of KEGG pathways of differential expressed (DE) genes in each cell subtype of P1-2 (disease improvement) vs. P1-3 (cured).** KEGG pathways were analyzed by using DE genes lists between cases and controls. The cell subtype is shown in Fig. 3a.


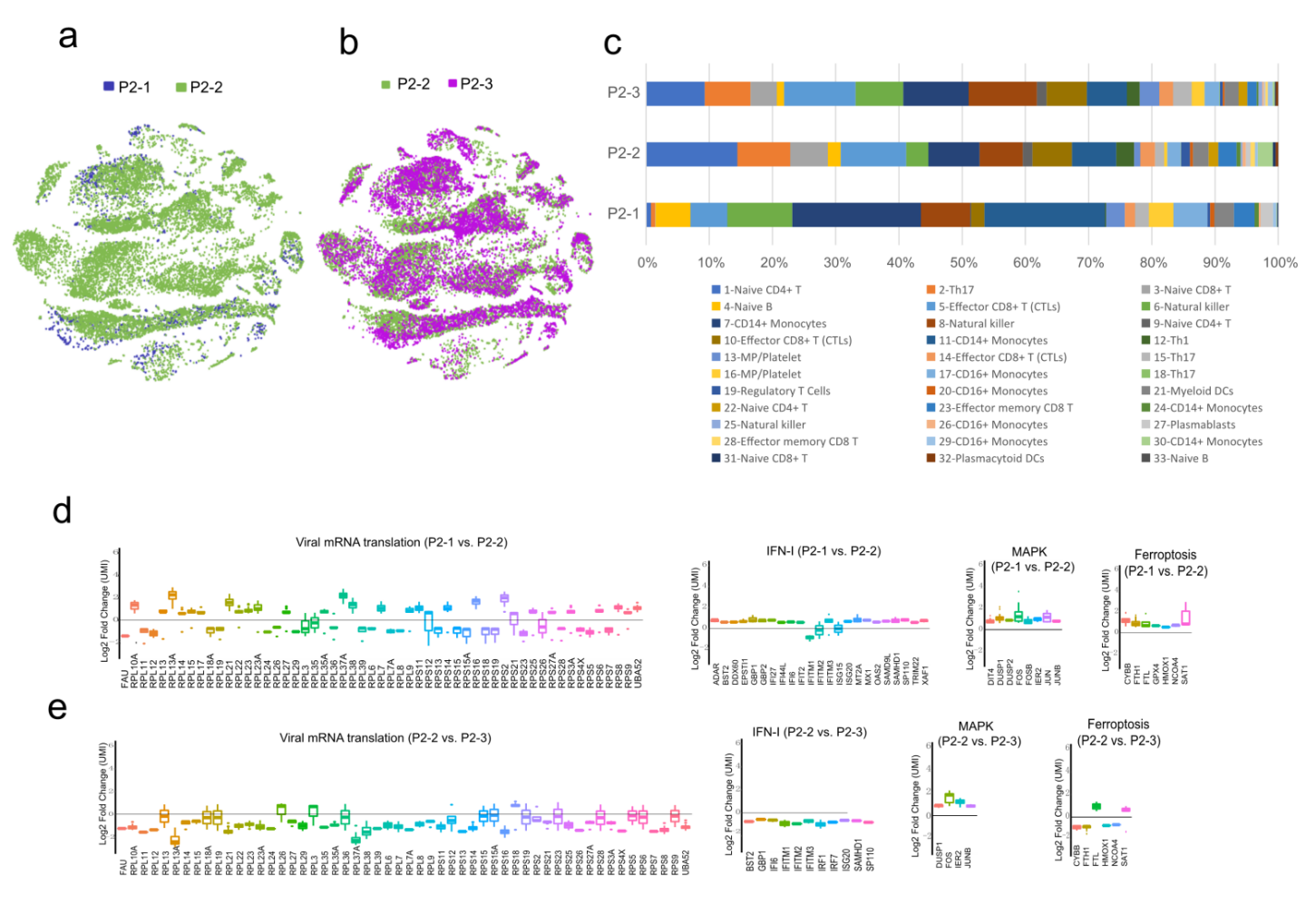


**Fig. S16 The dynamic changing picture of critical patient 2 during his recovery.**  **a** TSNE plots of P2-1(critical) and P2-2 (improved), suggesting the cell type distribution in the two samples. **b** TSNE plots of P2-2 and P2-3, suggesting the cell type distribution in the two samples. **c** Comparisons of all the cell subtypes of P2-1, P2-2, and P2-3. **d** Log2 fold changes of the UMI counts of the genes involved in viral mRNA translation, IFN-I, MAPK targets, and ferroptosis between P2-1 and P2-2. Each point represents a different cell subtype. **e** Log2 fold changes of the UMI counts of the genes involved in viral mRNA translation, IFN-I, MAPK targets, and ferroptosis between P2-2 and P2-3. Each point represents a different cell subtype. Only DE genes (FDR<0.05) were plotted for the comparison group.


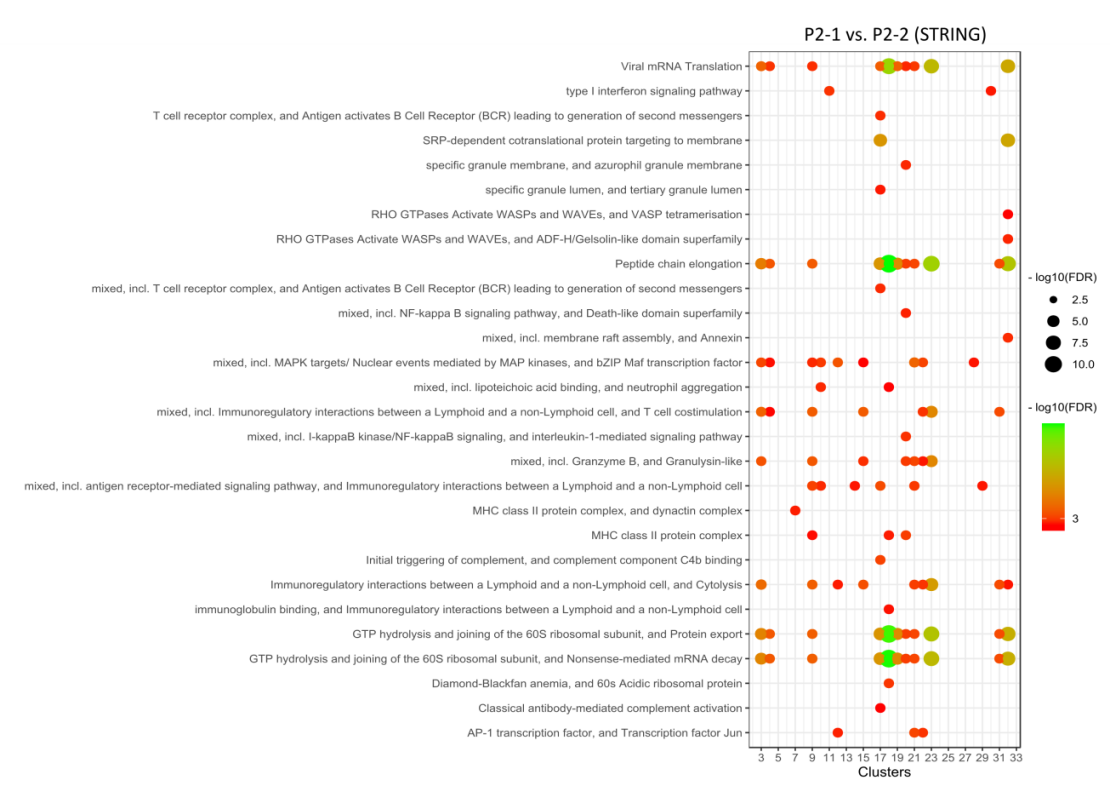


**Fig. S17 The enrichment of STRING network pathways of differential expressed (DE) genes in each cell subtype of P2-1 (in critical condition) vs. P2-2 (disease improvement).**  The cell subtype is shown in Fig. 3a. STRING network pathways were analyzed by using DE genes and their corresponding log2 fold changes of the UMI counts between cases and controls. The cell subtype is shown in Fig. 3a.


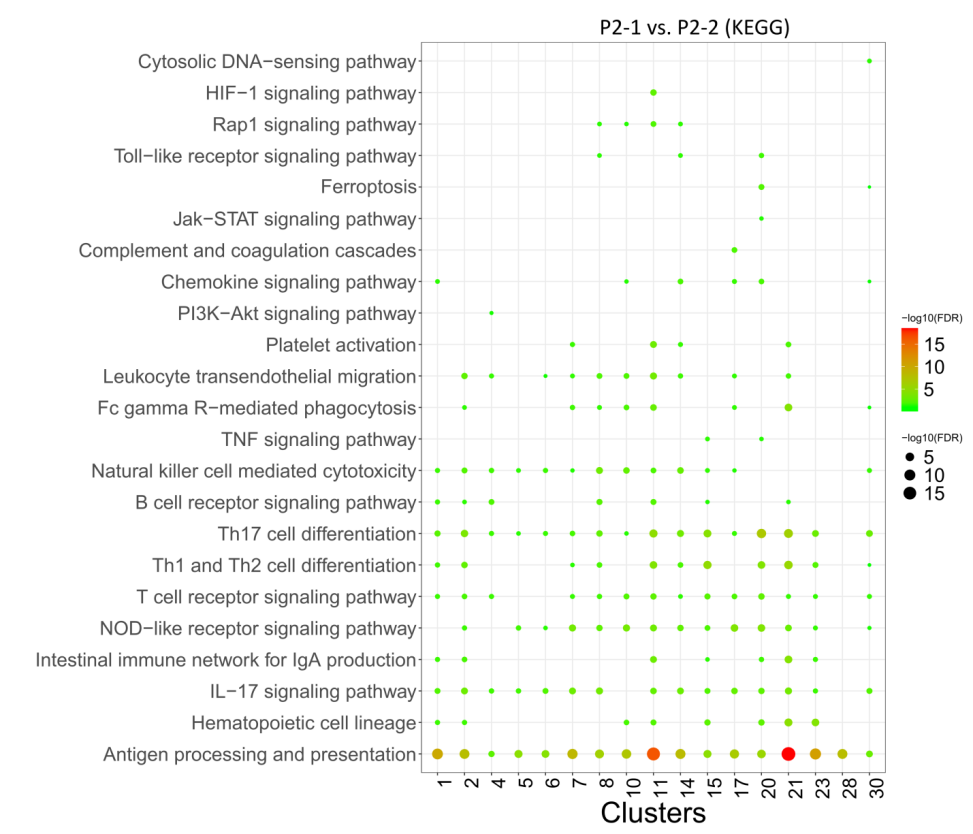


**Fig. S18 The enrichment of KEGG pathways of differential expressed (DE) genes in each cell subtype of P2-1 (in critical condition) vs. P2-2 (disease improvement).** KEGG pathways were analyzed by using DE genes lists between cases and controls. The cell subtype is shown in Fig. 3a.


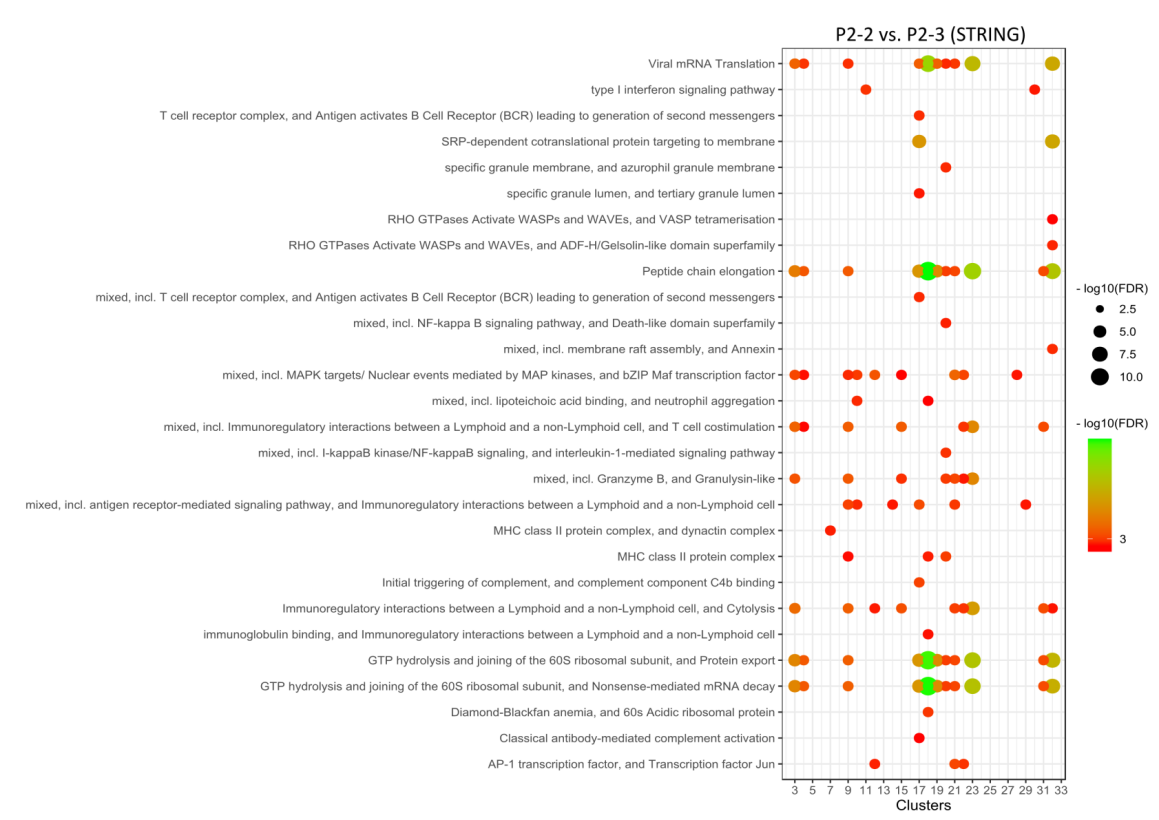


**Fig. S19 The enrichment of STRING network pathways of differential expressed (DE) genes in each cell subtype of P2-2 (disease improvement) vs. P2-3 (cured).**  The cell subtype is shown in Fig. 3a. STRING network pathways were analyzed by using DE genes and their corresponding log2 fold changes of the UMI counts between cases and controls.


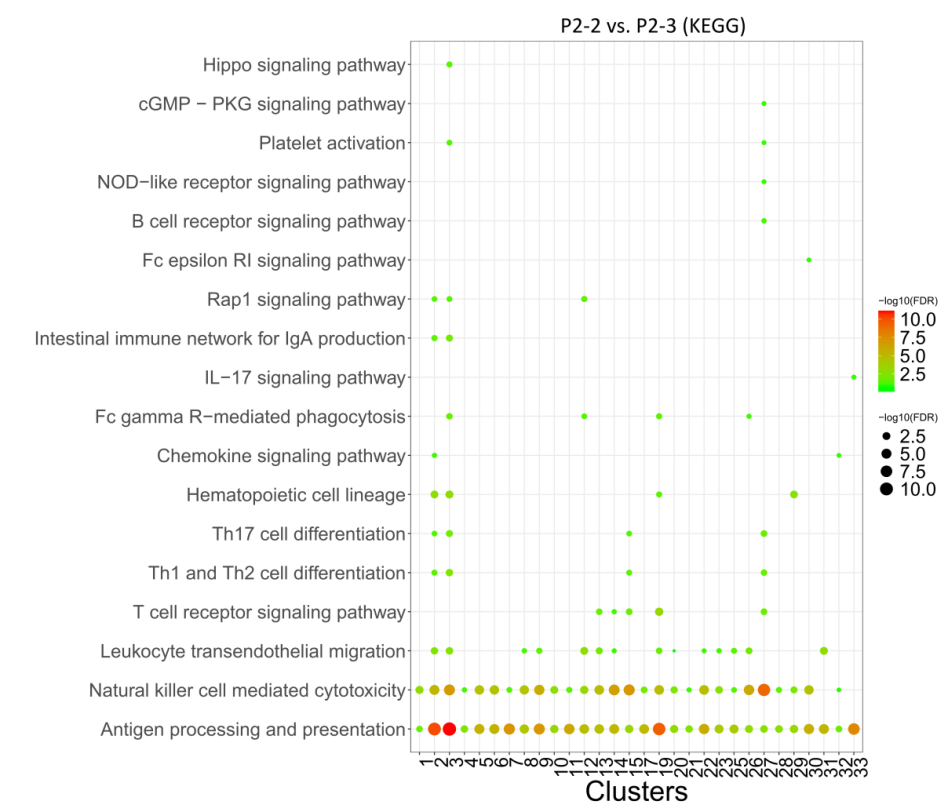


**Fig. S20 The enrichment of KEGG pathways of differential expressed (DE) genes in each cell subtype of P2-2 (disease improvement) vs. P2-3 (cured).** KEGG pathways were analyzed by using DE genes lists between cases and controls. The cell subtype is shown in Fig. 3a.


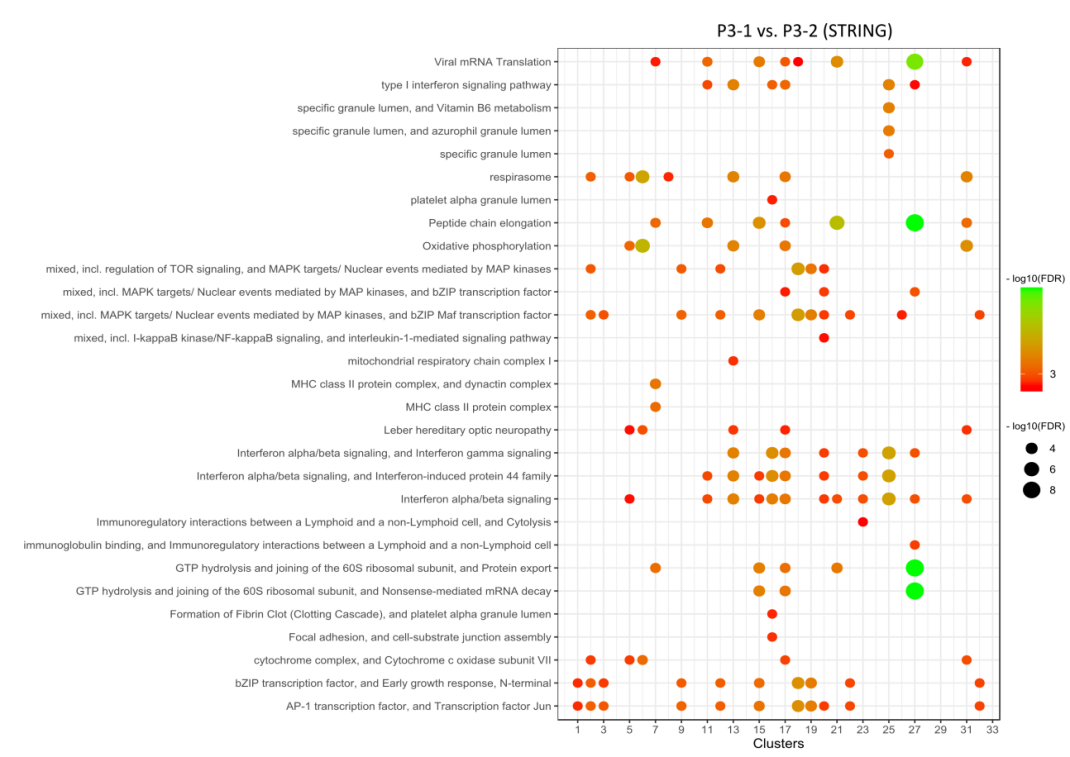


**Fig. S21 The enrichment of STRING network pathways of differential expressed (DE) genes in each cell subtype of P3-1 (disease improvement) vs. P3-2 (cured).** The cell subtype is shown in Fig. 3a. STRING network pathways were analyzed by using DE genes and their corresponding log2 fold changes of the UMI counts between cases and controls.


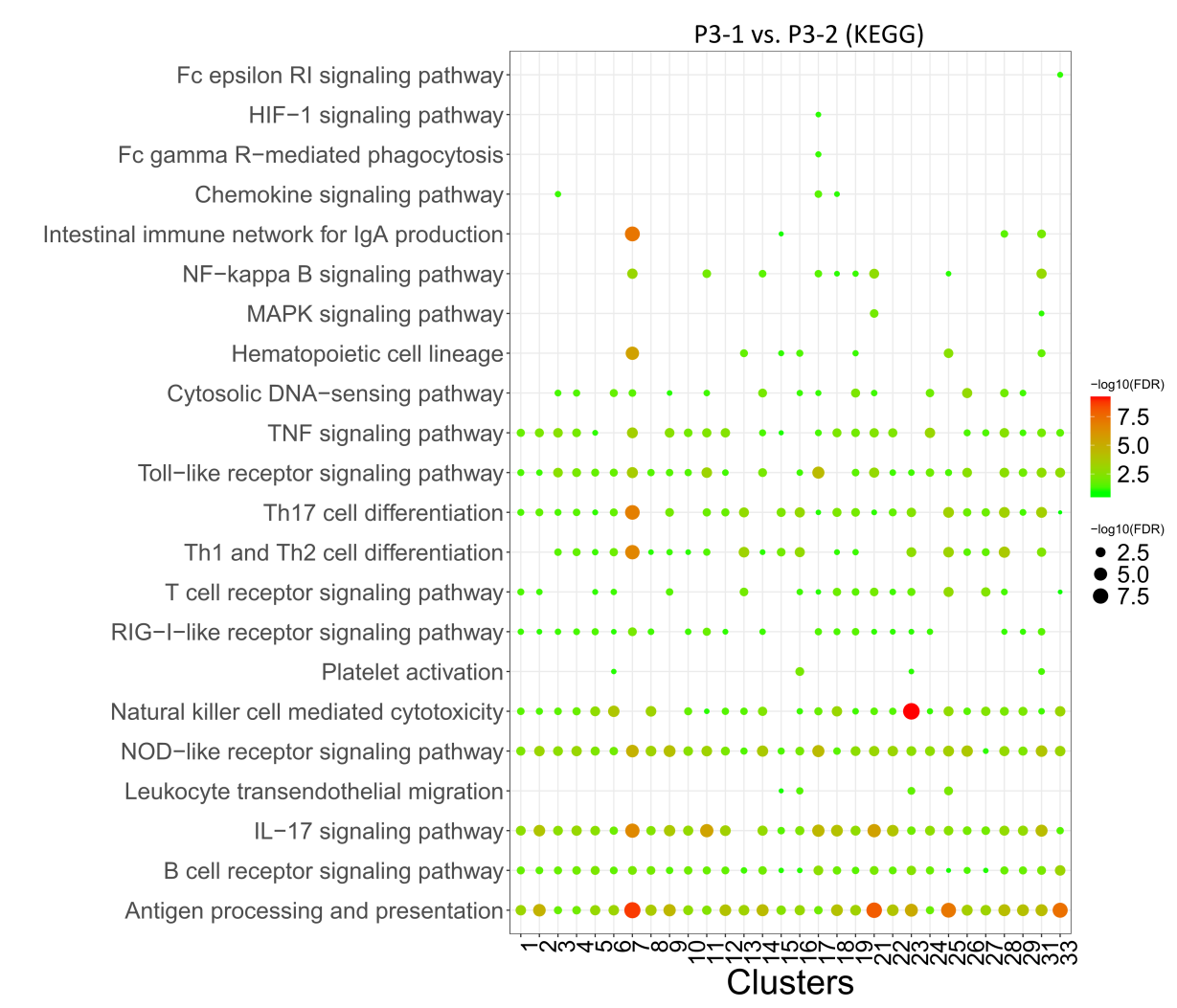


**Fig. S22 The enrichment of KEGG pathways of differential expressed (DE) genes in each cell subtype of P3-1 (severe) vs. P3-2 (cured).** KEGG pathways were analyzed by using DE genes lists between cases and controls. The cell subtype is shown in Fig. 3a.


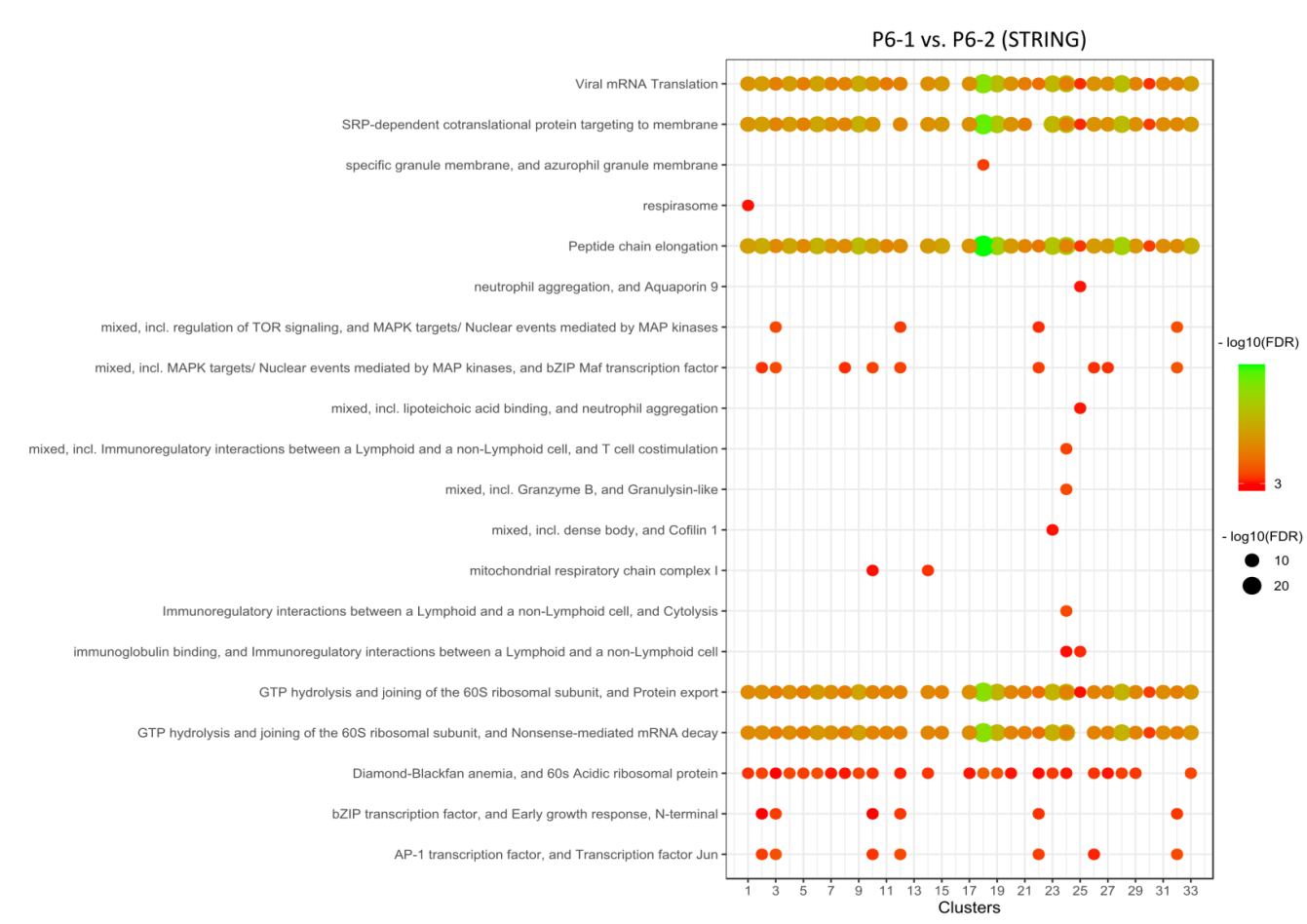


**Fig. S23 The enrichment of STRING network pathways of differential expressed (DE) genes in each cell subtype of P6-1 (moderate) vs. P6-2 (cured).**  The cell subtype is shown in Fig. 3a. STRING network pathways were analyzed by using DE genes and their corresponding log2 fold changes of the UMI counts between cases and controls.


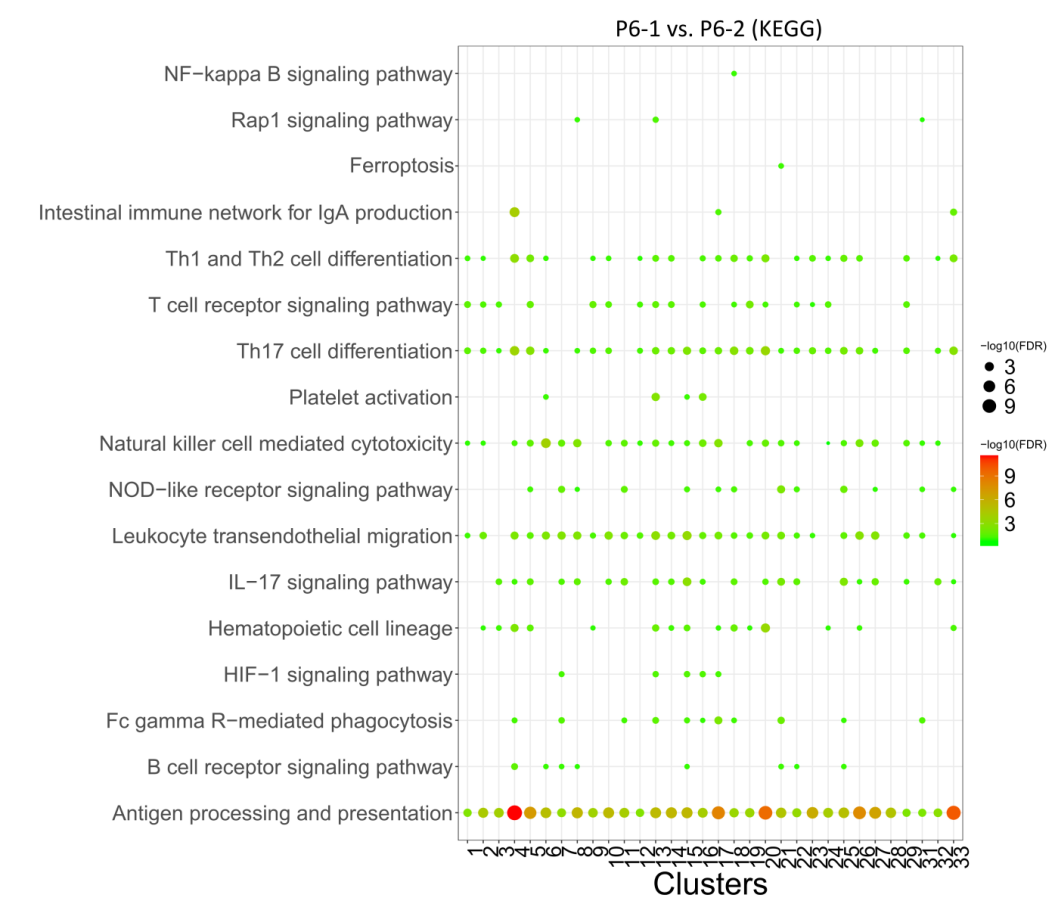


**Fig. S24 The enrichment of KEGG pathways of differential expressed (DE) genes in each cell subtype of P6-1 (moderate) vs. P6-2 (cured).** KEGG pathways were analyzed by using DE genes lists between cases and controls. The cell subtype is shown in Fig. 3a.


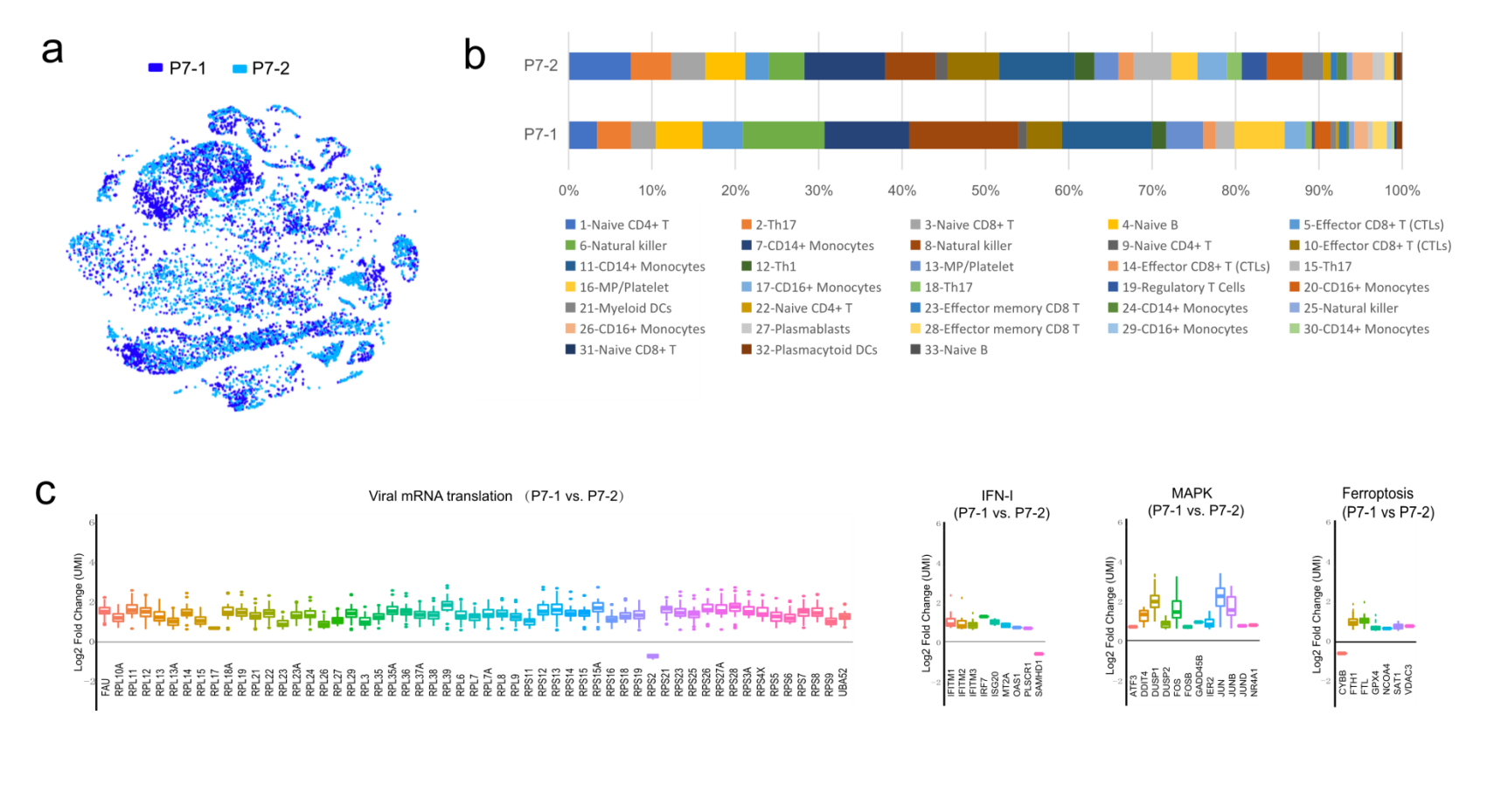


**Fig. S25 The dynamic study for moderate patient 7 in his disease and cured conditions. a** TSNE plots of P7-1 (moderate) vs. P7-2 (cured), showing the cell type distribution in the two states. **b** Comparisons of all the cell subtypes of P7-1 and P7-2. **c** Log2 fold changes of the UMI counts of the genes involved in viral mRNA translation, IFN-I, MAPK targets, and ferroptosis between P7-1 and P7-2. Each point represents a different cell subtype. Only DE genes (FDR<0.05) were plotted for the comparison group.


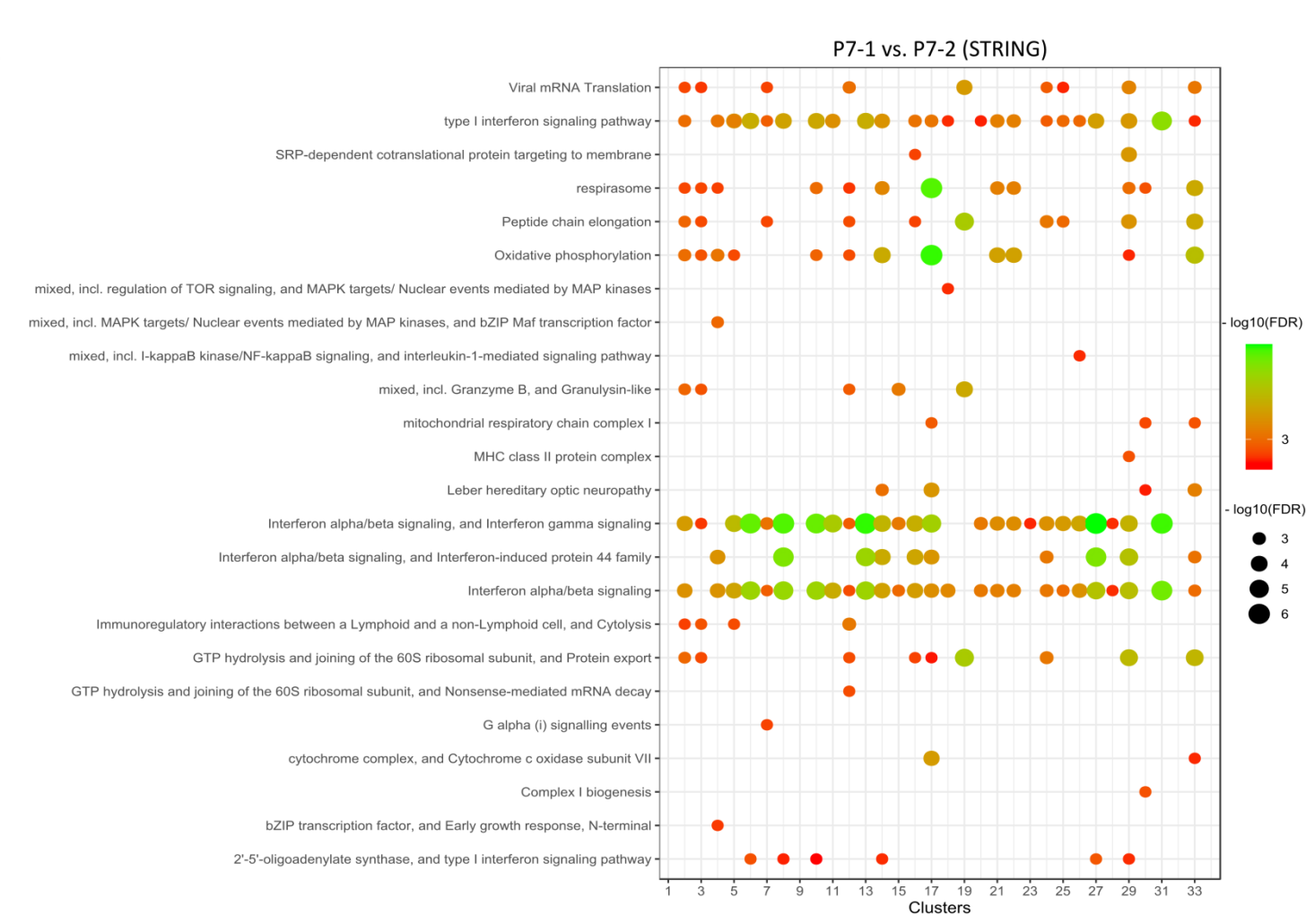


**Fig. S26 The enrichment of STRING network pathways of differential expressed (DE) genes in each cell subtype of P7-1 (moderate) vs. P7-2 (cured).**  The cell subtype is shown in Fig. 3a. STRING network pathways were analyzed by using DE genes and their corresponding log2 fold changes of the UMI counts between cases and controls.


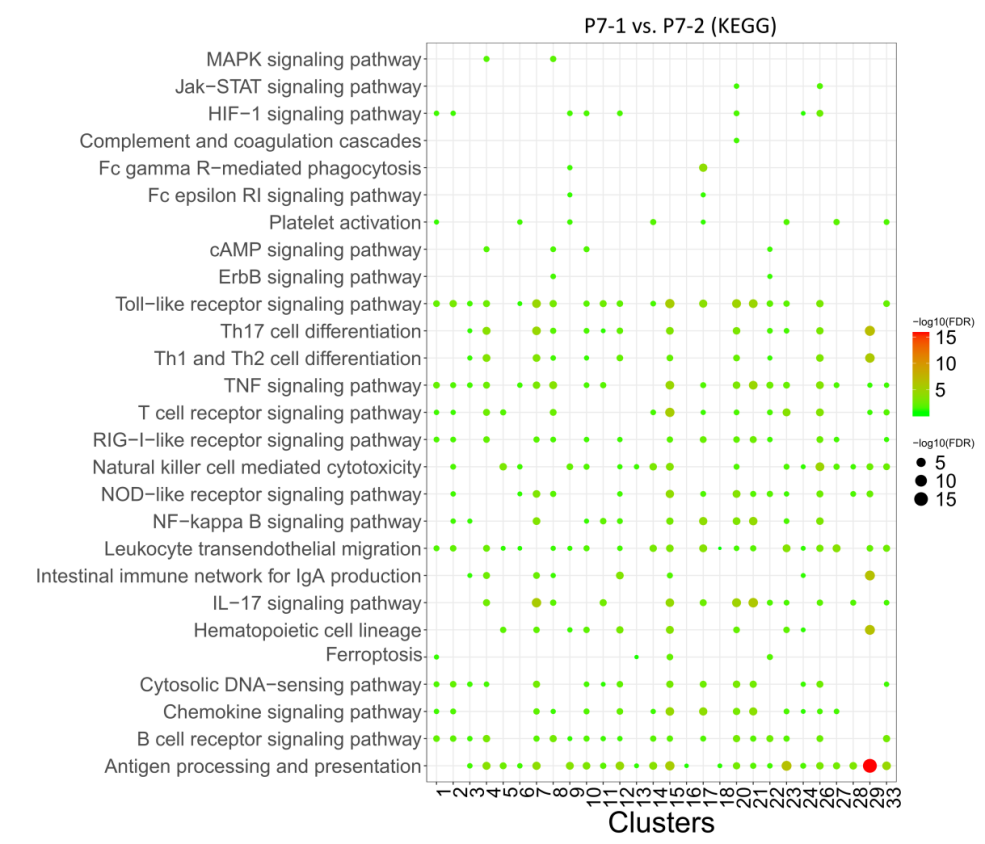


**Fig. S27 The enrichment of KEGG pathways of differential expressed (DE) genes in each cell subtype of P7-1 (moderate) vs. P7-2 (cured).** KEGG pathways were analyzed by using DE genes lists between cases and controls. The cell subtype is shown in Fig. 3a.


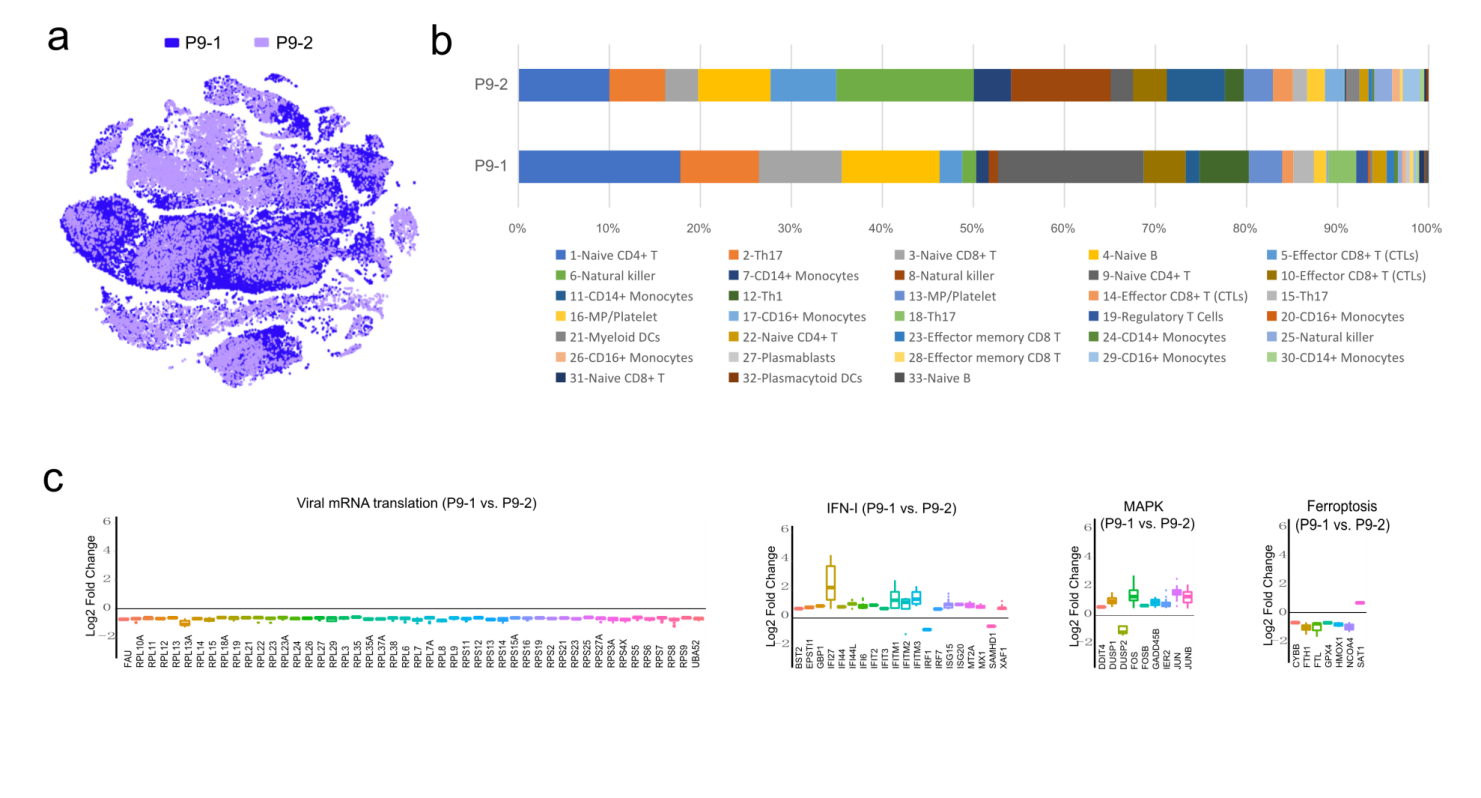


**Fig. S28 The dynamic study for moderate patient 9 in his disease and cured conditions. a** SNE plots of P9-1 (moderate) vs. P9-2 (cured), showing the cell type distribution in the two states. **b** Comparisons of all the cell subtypes of P9-1 and P9-2. **c** Log2 fold changes of the UMI counts of the genes involved in viral mRNA translation, IFN-I, MAPK targets, and ferroptosis between P9-1 and P9-2. Each point represents a different cell subtype. Only DE genes (FDR<0.05) were plotted for the comparison group.


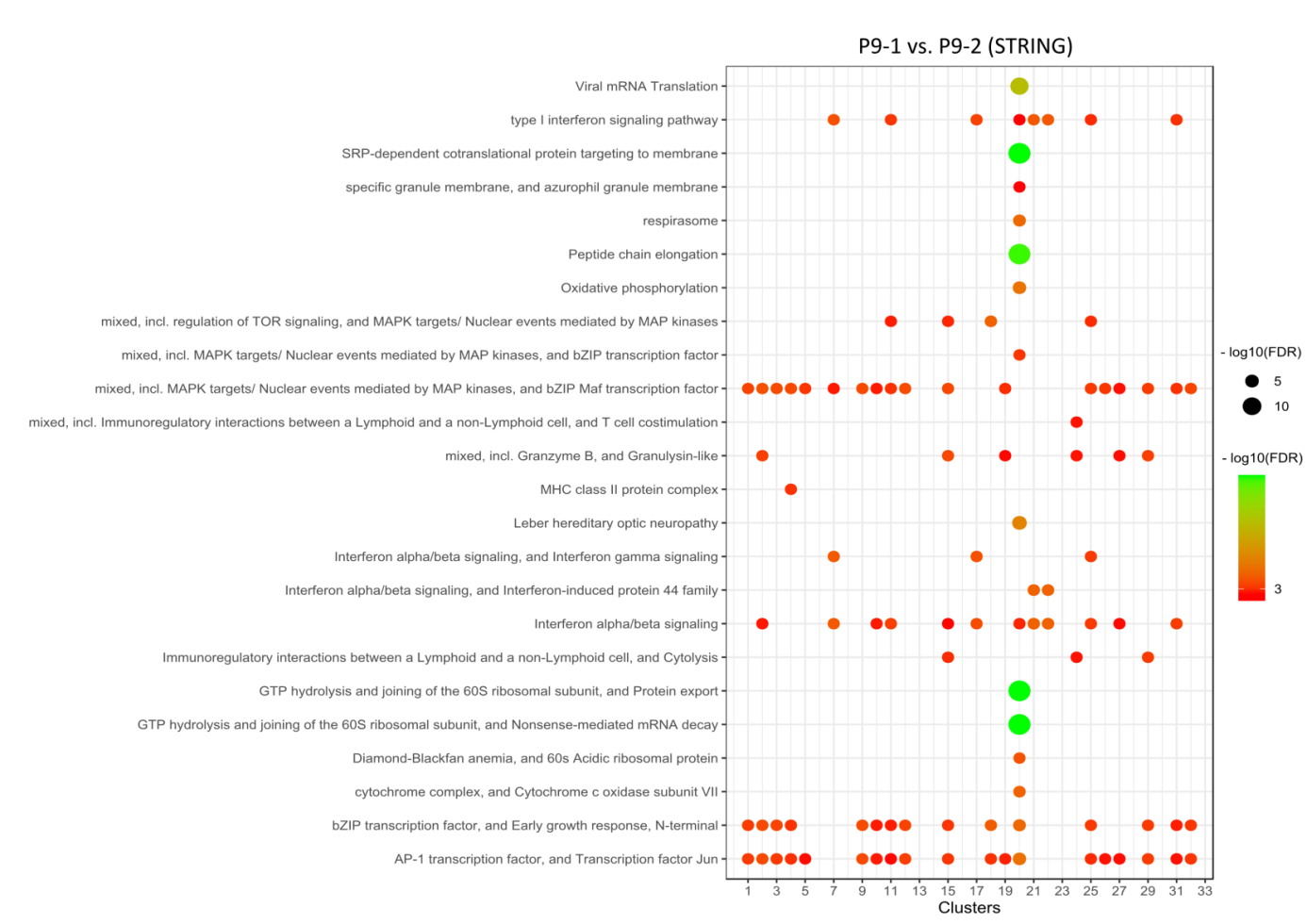


**Fig. S29 The enrichment of STRING network pathways of differential expressed (DE) genes in each cell subtype of P9-1 (moderate) vs. P9-2 (cured).**  The cell subtype is shown in Fig. 3a. STRING network pathways were analyzed by using DE genes and their corresponding log2 fold changes of the UMI counts between cases and controls.


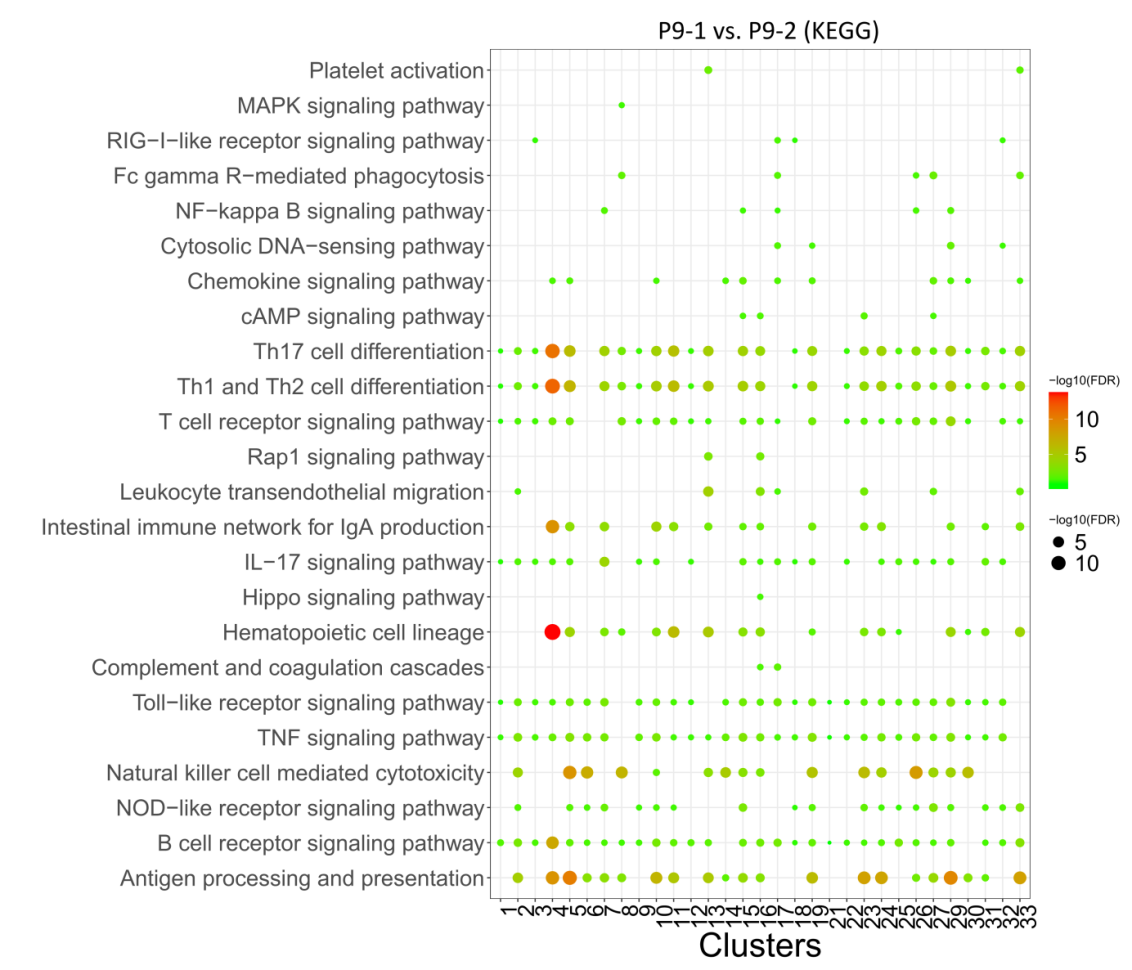


**Fig. S30 The enrichment of KEGG pathways of differential expressed (DE) genes in each cell subtype of P9-1 (moderate) vs. P9-2 (cured).** KEGG pathways were analyzed by using DE genes lists between cases and controls. The cell subtype is shown in Fig. 3a.


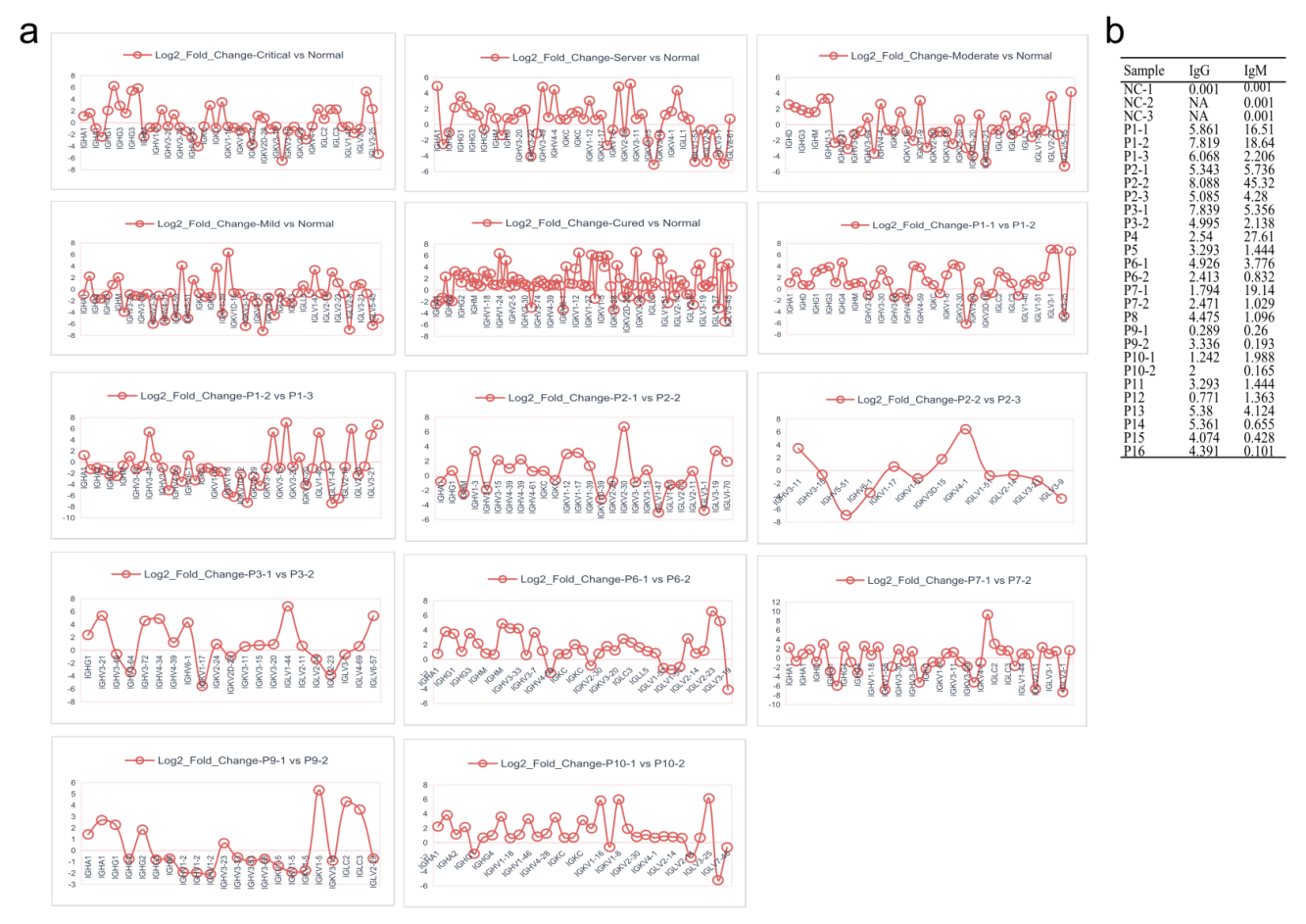


**Fig. S31 The dynamic study for Ig coding genes changes in 14 comparison group. a** Only DE genes (FDR<0.05) were plotted for each comparison group. **b** IgG and IgM values of for the scRNA samples detected by chemiluminescence.

**
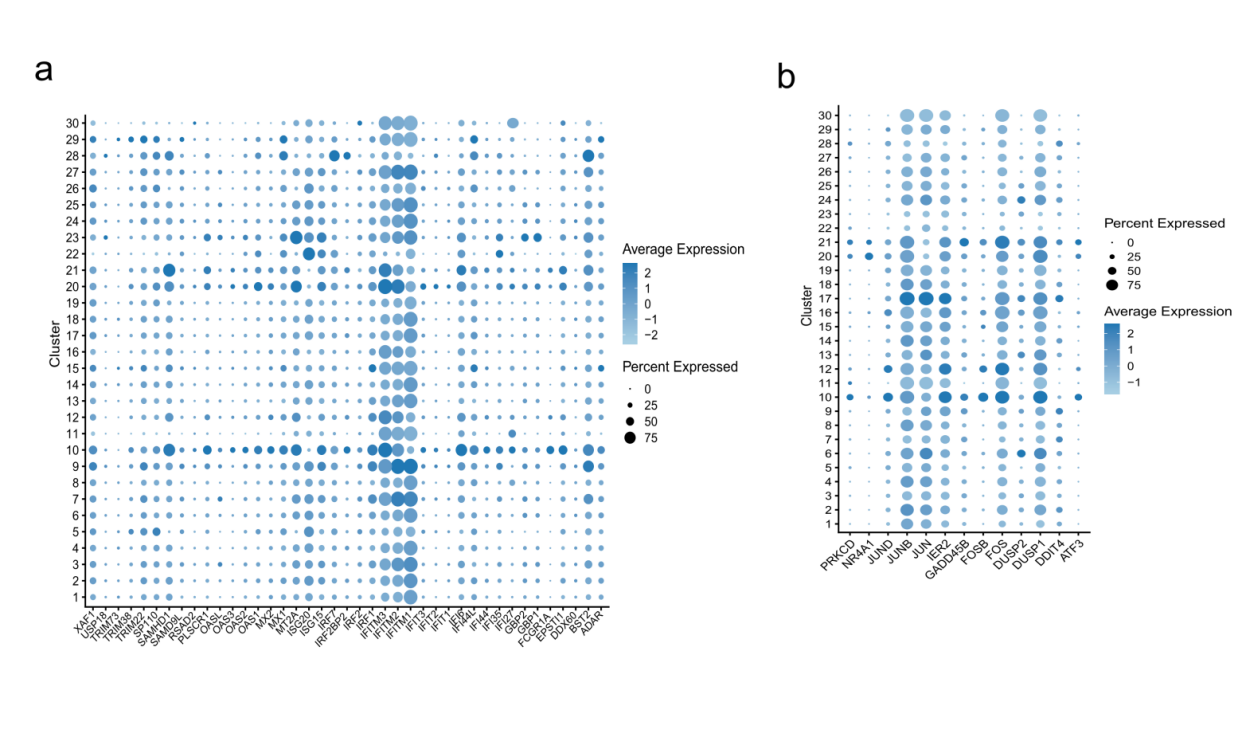
**

**Fig. S32 Expression profiles of IFN-I and MAPK pathway genes. a** Expression of IFN-I pathway genes across cell clusters of the 19 samples enrolled in the first round V(DJ) experiment. Dot size represents the fraction of cells expressing. The cluster name corresponds to Fig.1d. **b** Expression of MAPK targets pathway genes across cell clusters of the 19 samples enrolled in the first round V(DJ) experiment. Dot size represents the fraction of cells expressing. The cluster name corresponds to Fig.1d. The cell subtype is shown in Fig. 1d.

**Table S1 Clinical information 16 patients with COVID-19 patients and three controls.**

| **Patient** | **Gerder** | **Age (years)** | **Disease condition** | **Sampling days after disease diagnosis**（days） | **Hematocrit (normal range 0.40-0.50)** | **Mean corpuscular volume (fl) (normal range 82-100)** | **Mean corpuscular hemoglobin (pg) (normal range 27-34)** | **RBC distribution width (%); (normal range 10-16)** | **Platelet count (× 10**⁹ **cells / L);(normal range 100–300)** | **Lymphocyte (%); (normal range 20-50)** | **Monocyte (%); (normal range 3-10)** | **Neutrophils(%); (normal range40-75)** | **Eosinophils (%); (normal range 0.4-7)** | **Basophils (%); (normal range 0-2)** | **Lymphocyte count (× 10**⁹ **cells / L); (normal range 1.1-3.2)** | **Monocytes (× 10**⁹ **cells / L); (normal range 0.1 - 0.6)** | **Neutrophils (× 10**⁹ **cells / L); (normal range 1.8 - 6.3)** | **Eosinophils (× 10**⁹ **cells / L); (normal range 0.02-0.52)** | **Basophils (× 10**⁹ **cells / L); (normal range 0 - 0.2)** | **Leukocytes (× 10**⁹ **cells / L); (normal range 3.5-9.5)** | **Erythrocytes (× 10**⁹ **cells / L); (normal range 4.3-5.8)** |
| --- | --- | --- | --- | --- | --- | --- | --- | --- | --- | --- | --- | --- | --- | --- | --- | --- | --- | --- | --- | --- | --- |
| **Patient 1-1** | Male | 34 | Critical | 14 | 0.398 | 94.5 | 30.9 | - | 299 | 7.3 | 3.1 | 89.5 | 0 | 0.1 | 0.75 | 0.32 | 9.25 | 0 | 0.01 | 10.34 | 4.21 |
| **Patient 1-2** | Male | 34 | Improved | 14+22 | 0.36 | 97 | 31.6 | 43.7 | 152 | 34.5 | 8.6 | 49.4 | 7.1 | 0.4 | 1.64 | 2.35 | 0.34 | 0.02 | 0.4 | 4.76 | 3.76 |
| **Patient 1-3** | Male | 34 | Cured | 14+22+25 | 0.39 | 95 | 31.8 | 44.7 | 196 | 30.8 | 10.2 | 56.4 | 2.2 | 0.4 | 2.42 | 0.8 | 4.43 | 0.17 | 0.03 | 7.86 | 4.12 |
| **Patient 2-1** | Male | 33 | Critical | 13 | NA | 93 | NA | 38.6 | 144 | 4.7 | 5.4 | 89.9 | 0 | 0 | 0.27 | 0.31 | 5.17 | 0 | 0 | 5.75 | 3.04 |
| **Patient 2-2** | Male | 33 | Improved | 13+22 | 0.42 | 96 | 30.6 | 15.1 | 242 | 28.2 | 11.9 | 52.9 | 1.7 | 0.56 | 1.33 | 0.56 | 2.5 | 0.25 | 0.08 | 4.72 | 4.22 |
| **Patient 2-3** | Male | 33 | Cured | 13+22+23 | 0.36 | 77 | 27.9 | 13.5 | 361 | 37.1 | 12.7 | 58.1 | 1.6 | 0.5 | 2.82 | 0.21 | 4.42 | 0.12 | 0.03 | 7.6 | 4.69 |
| **Patient 3-1** | Male | 43 | Server | 8 | 0.46 | 92 | 31 | 12.6 | 142 | 30.4 | 14.5 | 52.9 | 1.3 | 0.9 | 1.88 | 0.48 | 1.75 | NA | NA | 3.3 | 5.06 |
| **Patient 3-2** | Male | 43 | Cured | 8+36 | 0.36 | 89 | 31.9 | 12.8 | 215 | 22.1 | 5.2 | 66.2 | 3.6 | 2.9 | 1.67 | 0.2 | 2.58 | 0.14 | 0.11 | 3.9 | 4.07 |
| **Patient 4** | Female | 33 | Moderate | 10 | 0.31 | 72 | 23.3 | 17 | 236 | 15.7 | 9.2 | 74 | 0.7 | 0.4 | 1.52 | 0.89 | 7.18 | 0.03 | 0.04 | 9.7 | 4.3 |
| **Patient 5** | Male | 26 | Moderate | 12 | 0.43 | 92 | 31.8 | 343 | 132 | 23.1 | 11.4 | 64.1 | 0.9 | 0.5 | 0.9 | 0.44 | 2.5 | 0.04 | 0.02 | 3.9 | 4.66 |
| **Patient 6-1** | Male | 25 | Moderate | 7 | 0.38 | 90 | 30.4 | 10.9 | 227 | 18.9 | 9.1 | 70.2 | 1.3 | 0.5 | 1.46 | 0.7 | 5.41 | 0.1 | 0.04 | 7.7 | 4.24 |
| **Patient 6-2** | Male | 25 | Cured | 7+16 | 0.43 | 89 | 29.4 | 11.7 | 243 | 23.3 | 9.7 | 64.5 | 2.1 | 0.4 | 1.74 | 0.72 | 4.81 | 0.16 | 0.03 | 7.48 | 4.8 |
| **Patient 7-1** | Female | 59 | Moderate | 4 | 0.51 | 87.8 | 30 | 39.5 | 274 | 34.7 | 5.5 | 58.9 | 0.6 | 0.3 | 2.2 | 0.35 | 3.73 | 0.04 | 0.02 | 6.34 | 5.84 |
| **Patient 7-2** | Female | 59 | Cured | 4+15 | 0.39 | 98.3 | 31 | 23.1 | 188.3 | 29.4 | 8.9 | 63.7 | 0.65 | 0.24 | 1.89 | 0.47 | 3.34 | 0.03 | 0.01 | 5.99 | 465 |
| **Patient 8** | Female | 46 | Moderate | 10 | 0.37 | 93.4 | 31.9 | NA | NA | NA | 8.2 | 68.1 | 0.1 | 0.2 | 1.03 | 0.36 | 2.98 | 0 | 0.01 | 4.38 | 3.98 |
| **Patient 9-1** | Female | 62 | Moderate | 10 | 0.36 | 89.5 | 30.5 | NA | NA | 27.4 | 6.8 | 65 | 0.6 | 0.2 | 1.4 | 0.35 | 3.32 | 0.03 | 0.01 | 5.11 | 3.97 |
| **Patient 9-2** | Female | 62 | Cured | 10+15 | 0.36 | 89.5 | 30.5 | 13.8 | 198 | 28.8 | 8 | 63.3 | 0.87 | 0.3 | 1.67 | 0.4 | 2.98 | 0.04 | 0.02 | 4.45 | 4.32 |
| **Patient 10-1** | Female | 18 | Mild | 3 | 0.36 | 88 | 29 | 14 | 189 | 35.1 | 5.7 | 57.7 | 0.9 | 0.6 | 1.97 | 0.32 | 3.23 | 0.05 | 0.03 | 5.6 | 4.07 |
| **Patient 10-2** | Female | 18 | Cured | 3+14 | 0.33 | 91 | 28.6 | 13.2 | 274 | 35.7 | 6.2 | 56.4 | 1.3 | 0.4 | 1.67 | 0.29 | 2.64 | 0.06 | 0.02 | 4.68 | 3.6 |
| **Patient 11** | Male | 30 | Mild | 4 | 0.489 | 88.6 | 30.8 | 40.5 | 184 | 24.8 | 7.3 | 66.5 | 1.1 | 0.3 | 1.133 | 0.334 | 3.039 | 0.05 | 0.014 | 4.57 | 5.53 |
| **Patient 12** | Female | 32 | Mild | 3 | 0.37 | 87.6 | 30.7 | 43.3 | 157 | 42.9 | 7.6 | 46.8 | 2.4 | 0.3 | 1.261 | 0.223 | 1.376 | 0.071 | 0.009 | 2.94 | 4.23 |
| **Patient** 13 | Female | 20 | Cured | 21 | 0.38 | 70.2 | 21.9 | NA | 173 | 26 | 12.3 | 61.1 | 0.4 | 0.2 | 2.32 | 1.1 | 5.46 | 0.04 | 0.02 | 8.93 | 5.24 |
| **Patient 14** | Male | 40 | Cured | 23 | 0.4 | 84.4 | 26.8 | NA | 118 | 24 | 11.2 | 64.4 | 0 | 0.4 | 1.16 | 0.54 | 3.12 | 0 | 0.02 | 4.84 | 4.73 |
| **Patient 15** | Female | 20 | Cured | 25 | 0.363 | 77 | 27.9 | 13.5 | 270 | 37.1 | 2.7 | 58.1 | 1.6 | 0.5 | 2.82 | 0.21 | 4.42 | 0.12 | 0.04 | 7.6 | 4.69 |
| **Patient 16** | Male | 20 | Cured | 24 | 0.488 | 89.5 | 31 | 41.4 | 217 | 36.2 | 9.6 | 51.5 | 2.3 | 0.4 | 1.358 | 0.36 | 1.931 | 0.086 | 0.015 | 3.75 | 5.46 |
| **Normal-1** | Female | 57 | Normal | NA | 0.46 | 90.3 | 29.3 | 39.5 | 156 | 33 | 4.2 | 61.1 | 1.4 | 0.3 | 2.68 | 0.34 | 4.97 | 0.11 | 0.02 | 8.13 | 5.06 |
| **Normal-2** | Male | 61 | Normal | NA | 0.43 | 94.8 | 30 | 45.1 | 111 | 31.7 | 7.6 | 59.1 | 1.1 | 0.5 | 1.37 | 0.33 | 2.55 | 0.05 | 0.02 | 4.32 | 4.54 |
| **Normal-3** | Male | 28 | Normal | NA | 0.44 | 87.7 | 27.6 | 40.1 | 248 | 39.3 | 8.7 | 49.7 | 1.9 | 0.4 | 2.12 | 0.47 | 2.68 | 0.1 | 0.02 | 5.4 | 5.04 |

**Table S2 Compared with the normal controls, significantly enriched pathways of each cell subtypes in the critical condition (patient 1 and patient 2). Cell type refers to Fig. 1d.**

| **Cluster** | **#term ID** | **term description** | **genes mapped** | **enrichment score** | **direction** | **false discovery rate** | **method** | **matching proteins in your input (IDs)** | **matching proteins in your input (labels)** |
| --- | --- | --- | --- | --- | --- | --- | --- | --- | --- |
| Cluster15 | CL:14967 | GTP hydrolysis and joining of the 60S ribosomal subunit, and Protein export | 14 | 3.95523 | top of list | 0.00068 | afc | ENSP00000230050,ENSP00000272317,ENSP00000278572,ENSP00000296674,ENSP00000318646,ENSP00000339063,ENSP00000345957,ENSP00000346001,ENSP00000357555,ENSP00000363676,ENSP00000393241,ENSP00000413436,ENSP00000416429,ENSP00000428085 | EEF1A1,RPL10,RPL11,RPL3,RPL30,RPL32,RPS12,RPS15A,RPS18,RPS21,RPS23,RPS27,RPS27A,RPS3 |
| Cluster15 | CL:14976 | Peptide chain elongation | 13 | 3.95847 | top of list | 0.00073 | afc | ENSP00000230050,ENSP00000272317,ENSP00000296674,ENSP00000318646,ENSP00000339063,ENSP00000345957,ENSP00000346001,ENSP00000357555,ENSP00000363676,ENSP00000393241,ENSP00000413436,ENSP00000416429,ENSP00000428085 | EEF1A1,RPL10,RPL11,RPL3,RPL30,RPL32,RPS12,RPS15A,RPS18,RPS21,RPS23,RPS27,RPS27A |
| Cluster15 | CL:14978 | Peptide chain elongation | 12 | 3.93397 | top of list | 0.001 | afc | ENSP00000230050,ENSP00000272317,ENSP00000296674,ENSP00000318646,ENSP00000339063,ENSP00000345957,ENSP00000346001,ENSP00000357555,ENSP00000363676,ENSP00000393241,ENSP00000413436,ENSP00000416429 | EEF1A1,RPL10,RPL11,RPL3,RPL32,RPS12,RPS15A,RPS18,RPS21,RPS23,RPS27,RPS27A |
| Cluster15 | CL:14982 | Peptide chain elongation | 11 | 3.95149 | top of list | 0.0013 | afc | ENSP00000230050,ENSP00000272317,ENSP00000296674,ENSP00000318646,ENSP00000345957,ENSP00000346001,ENSP00000357555,ENSP00000363676,ENSP00000393241,ENSP00000413436,ENSP00000416429 | RPL10,RPL11,RPL3,RPL32,RPS12,RPS15A,RPS18,RPS21,RPS23,RPS27,RPS27A |
| Cluster15 | CL:14983 | Peptide chain elongation | 9 | 3.87924 | top of list | 0.0061 | afc | ENSP00000230050,ENSP00000272317,ENSP00000296674,ENSP00000318646,ENSP00000345957,ENSP00000346001,ENSP00000363676,ENSP00000393241,ENSP00000416429 | RPL11,RPL3,RPL32,RPS12,RPS15A,RPS18,RPS21,RPS23,RPS27A |
| Cluster15 | CL:14985 | Viral mRNA Translation | 8 | 3.89922 | top of list | 0.0081 | afc | ENSP00000230050,ENSP00000272317,ENSP00000296674,ENSP00000318646,ENSP00000345957,ENSP00000346001,ENSP00000363676,ENSP00000393241 | RPL11,RPL3,RPS12,RPS15A,RPS18,RPS21,RPS23,RPS27A |
| Cluster15 | CL:4209 | mixed, incl. Immunoregulatory interactions between a Lymphoid and a non-Lymphoid cell, and T cell costimulation | 4 | 5.32994 | top of list | 0.0082 | afc | ENSP00000236147,ENSP00000263863,ENSP00000399168,ENSP00000452780 | B2M,GNLY,HLA-B,SELL |
| Cluster | #term ID | term description | genes mapped | enrichment score | direction | false discovery rate | method | matching proteins in your input (IDs) | matching proteins in your input (labels) |
| Cluster16 | CL:14978 | Peptide chain elongation | 11 | 3.87038 | top of list | 0.0058 | afc | ENSP00000251453,ENSP00000307889,ENSP00000339063,ENSP00000346001,ENSP00000346027,ENSP00000357555,ENSP00000362744,ENSP00000369757,ENSP00000379506,ENSP00000389103,ENSP00000393241 | EEF1A1,RPL13,RPL14,RPL21,RPL23A,RPL3,RPS16,RPS18,RPS27,RPS4X,RPS6 |
| Cluster16 | CL:14966 | GTP hydrolysis and joining of the 60S ribosomal subunit, and Protein export | 12 | 3.87677 | top of list | 0.0058 | afc | ENSP00000251453,ENSP00000307889,ENSP00000339063,ENSP00000346001,ENSP00000346027,ENSP00000357555,ENSP00000362744,ENSP00000369757,ENSP00000379506,ENSP00000389103,ENSP00000393241,ENSP00000477781 | EEF1A1,RPL13,RPL14,RPL21,RPL23A,RPL3,RPS16,RPS18,RPS27,RPS4X,RPS6,TPT1 |
| Cluster | #term ID | term description | genes mapped | enrichment score | direction | false discovery rate | method | matching proteins in your input (IDs) | matching proteins in your input (labels) |
| Cluster19 | CL:18630 | MHC class II protein complex | 9 | 6.28757 | top of list | 7.14E-06 | afc | ENSP00000009530,ENSP00000339398,ENSP00000353099,ENSP00000363976,ENSP00000364080,ENSP00000378786,ENSP00000393566,ENSP00000398890,ENSP00000408146 | CD74,HLA-DMA,HLA-DMB,HLA-DPA1,HLA-DPB1,HLA-DQA1,HLA-DQB1,HLA-DRA,HLA-DRB1 |
| Cluster19 | CL:2403 | mixed, incl. MAPK targets/ Nuclear events mediated by MAP kinases, and bZIP Maf transcription factor | 4 | 8.00064 | bottom of list | 8.26E-05 | afc | ENSP00000239223,ENSP00000252818,ENSP00000306245,ENSP00000360266 | DUSP1,FOS,JUN,JUND |
| Cluster19 | CL:18627 | MHC class II protein complex, and dynactin complex | 10 | 5.26751 | top of list | 8.26E-05 | afc | ENSP00000009530,ENSP00000339398,ENSP00000353099,ENSP00000363976,ENSP00000364076,ENSP00000364080,ENSP00000378786,ENSP00000393566,ENSP00000398890,ENSP00000408146 | CD74,HLA-DMA,HLA-DMB,HLA-DPA1,HLA-DPB1,HLA-DQA1,HLA-DQA2,HLA-DQB1,HLA-DRA,HLA-DRB1 |
| Cluster19 | CL:18633 | peptide antigen assembly with MHC class II protein complex | 5 | 6.93524 | top of list | 0.00016 | afc | ENSP00000009530,ENSP00000353099,ENSP00000363976,ENSP00000378786,ENSP00000398890 | CD74,HLA-DMA,HLA-DMB,HLA-DRA,HLA-DRB1 |
| Cluster19 | CL:14980 | Peptide chain elongation | 20 | 3.30638 | bottom of list | 0.0019 | afc | ENSP00000251453,ENSP00000259469,ENSP00000274242,ENSP00000296674,ENSP00000309830,ENSP00000318646,ENSP00000322419,ENSP00000341885,ENSP00000346015,ENSP00000346088,ENSP00000355315,ENSP00000357555,ENSP00000375730,ENSP00000386717,ENSP00000400467,ENSP00000416429,ENSP00000418868,ENSP00000429374,ENSP00000464342,ENSP00000470972 | RPL13A,RPL22,RPL27A,RPL29,RPL31,RPL32,RPL35,RPL36,RPL37,RPL38,RPL39,RPL9,RPLP2,RPS15A,RPS16,RPS19,RPS2,RPS20,RPS23,RPS27 |
| Cluster19 | CL:4212 | Immunoregulatory interactions between a Lymphoid and a non-Lymphoid cell, and Cytolysis | 6 | 5.80259 | top of list | 0.0064 | afc | ENSP00000216338,ENSP00000221978,ENSP00000263863,ENSP00000365402,ENSP00000399168,ENSP00000452780 | B2M,GNLY,GZMH,HLA-B,HLA-C,NKG7 |
| Cluster19 | CL:4594 | mixed, incl. lipoteichoic acid binding, and neutrophil aggregation | 3 | 7.51004 | bottom of list | 0.0064 | afc | ENSP00000262629,ENSP00000357722,ENSP00000357727 | S100A8,S100A9,TYROBP |
| **Cluster** | **#term ID** | **term description** | **genes mapped** | **enrichment score** | **direction** | **false discovery rate** | **method** | **matching proteins in your input (IDs)** | **matching proteins in your input (labels)** |
| Cluster19 | CL:2482 | AP-1 transcription factor, and Transcription factor Jun | 3 | 7.33419 | bottom of list | 0.0022 | afc | ENSP00000252818,ENSP00000306245,ENSP00000360266 | FOS,JUN,JUND |
| Cluster19 | CL:14982 | Peptide chain elongation | 19 | 3.34885 | bottom of list | 0.0022 | afc | ENSP00000251453,ENSP00000259469,ENSP00000274242,ENSP00000296674,ENSP00000309830,ENSP00000318646,ENSP00000322419,ENSP00000341885,ENSP00000346088,ENSP00000355315,ENSP00000357555,ENSP00000375730,ENSP00000386717,ENSP00000400467,ENSP00000416429,ENSP00000418868,ENSP00000429374,ENSP00000464342,ENSP00000470972 | RPL13A,RPL22,RPL29,RPL31,RPL32,RPL35,RPL36,RPL37,RPL38,RPL39,RPL9,RPLP2,RPS15A,RPS16,RPS19,RPS2,RPS20,RPS23,RPS27 |
| Cluster19 | CL:14983 | Peptide chain elongation | 16 | 3.30433 | bottom of list | 0.0045 | afc | ENSP00000251453,ENSP00000259469,ENSP00000274242,ENSP00000296674,ENSP00000309830,ENSP00000318646,ENSP00000322419,ENSP00000341885,ENSP00000346088,ENSP00000355315,ENSP00000375730,ENSP00000400467,ENSP00000416429,ENSP00000418868,ENSP00000464342,ENSP00000470972 | RPL13A,RPL22,RPL29,RPL32,RPL35,RPL36,RPL37,RPL38,RPL39,RPL9,RPLP2,RPS15A,RPS16,RPS19,RPS2,RPS23 |
| Cluster | #term ID | term description | genes mapped | enrichment score | direction | false discovery rate | method | matching proteins in your input (IDs) | matching proteins in your input (labels) |
| Cluster21 | CL:18630 | MHC class II protein complex | 8 | 5.17413 | top of list | 0.003 | afc | ENSP00000009530,ENSP00000339398,ENSP00000353099,ENSP00000363976,ENSP00000364080,ENSP00000378786,ENSP00000393566,ENSP00000408146 | CD74,HLA-DMA,HLA-DPA1,HLA-DPB1,HLA-DQA1,HLA-DQB1,HLA-DRA,HLA-DRB1 |
| Cluster | #term ID | term description | genes mapped | enrichment score | direction | false discovery rate | method | matching proteins in your input (IDs) | matching proteins in your input (labels) |
| Cluster22 | CL:22327 | Oxidative phosphorylation | 9 | 5.41168 | bottom of list | 0.0002 | afc | ENSP00000300688,ENSP00000354554,ENSP00000354665,ENSP00000354687,ENSP00000354813,ENSP00000354961,ENSP00000354982,ENSP00000355046,ENSP00000355265 | ATP5L,MT-ATP8,MT-CO3,MT-CYB,MT-ND1,MT-ND2,MT-ND4,MT-ND5,MT-ND6 |
| Cluster22 | CL:22328 | respirasome | 7 | 6.28096 | bottom of list | 0.0002 | afc | ENSP00000354554,ENSP00000354665,ENSP00000354687,ENSP00000354813,ENSP00000354961,ENSP00000354982,ENSP00000355046 | MT-CO3,MT-CYB,MT-ND1,MT-ND2,MT-ND4,MT-ND5,MT-ND6 |
| Cluster22 | CL:22408 | Leber hereditary optic neuropathy | 4 | 6.57424 | bottom of list | 0.004 | afc | ENSP00000354665,ENSP00000354687,ENSP00000354813,ENSP00000355046 | MT-ND1,MT-ND2,MT-ND5,MT-ND6 |
| Cluster | #term ID | term description | genes mapped | enrichment score | direction | false discovery rate | method | matching proteins in your input (IDs) | matching proteins in your input (labels) |
| Cluster23 | CL:4615 | neutrophil aggregation, and Aquaporin 9 | 3 | 7.80683 | bottom of list | 0.0074 | afc | ENSP00000357722,ENSP00000357726,ENSP00000357727 | S100A12,S100A8,S100A9 |
| Cluster | #term ID | term description | genes mapped | enrichment score | direction | false discovery rate | method | matching proteins in your input (IDs) | matching proteins in your input (labels) |
| Cluster24 | CL:4212 | Immunoregulatory interactions between a Lymphoid and a non-Lymphoid cell, and Cytolysis | 3 | 8.44105 | top of list | 0.0047 | afc | ENSP00000263863,ENSP00000365402,ENSP00000399168 | GNLY,HLA-B,HLA-C |
| Cluster | #term ID | term description | genes mapped | enrichment score | direction | false discovery rate | method | matching proteins in your input (IDs) | matching proteins in your input (labels) |
| Cluster25 | CL:4212 | Immunoregulatory interactions between a Lymphoid and a non-Lymphoid cell, and Cytolysis | 7 | 6.08002 | both ends | 4.35E-05 | afc | ENSP00000216338,ENSP00000216341,ENSP00000221978,ENSP00000231009,ENSP00000263863,ENSP00000365402,ENSP00000399168 | GNLY,GZMB,GZMH,GZMK,HLA-B,HLA-C,NKG7 |
| Cluster25 | CL:4211 | Immunoregulatory interactions between a Lymphoid and a non-Lymphoid cell, and Cytolysis | 8 | 5.10096 | both ends | 0.00013 | afc | ENSP00000216338,ENSP00000216341,ENSP00000221978,ENSP00000231009,ENSP00000263863,ENSP00000357036,ENSP00000365402,ENSP00000399168 | GNLY,GZMB,GZMH,GZMK,HLA-B,HLA-C,NKG7,SLAMF6 |
| Cluster25 | CL:4209 | mixed, incl. Immunoregulatory interactions between a Lymphoid and a non-Lymphoid cell, and T cell costimulation | 9 | 4.54485 | both ends | 0.00019 | afc | ENSP00000216338,ENSP00000216341,ENSP00000221978,ENSP00000231009,ENSP00000263863,ENSP00000266557,ENSP00000357036,ENSP00000365402,ENSP00000399168 | CD27,GNLY,GZMB,GZMH,GZMK,HLA-B,HLA-C,NKG7,SLAMF6 |
| Cluster25 | CL:4300 | mixed, incl. Granzyme B, and Granulysin-like | 4 | 7.30882 | top of list | 0.00052 | afc | ENSP00000216338,ENSP00000216341,ENSP00000221978,ENSP00000263863 | GNLY,GZMB,GZMH,NKG7 |
| Cluster25 | CL:2403 | mixed, incl. MAPK targets/ Nuclear events mediated by MAP kinases, and bZIP Maf transcription factor | 3 | 7.67142 | bottom of list | 0.00084 | afc | ENSP00000239223,ENSP00000303315,ENSP00000306245 | DUSP1,FOS,JUNB |
| Cluster | #term ID | term description | genes mapped | enrichment score | direction | false discovery rate | method | matching proteins in your input (IDs) | matching proteins in your input (labels) |
| Cluster27 | CL:18630 | MHC class II protein complex | 7 | 5.71147 | top of list | 0.0026 | afc | ENSP00000009530,ENSP00000339398,ENSP00000353099,ENSP00000363976,ENSP00000378786,ENSP00000393566,ENSP00000408146 | CD74,HLA-DMA,HLA-DPA1,HLA-DPB1,HLA-DQA1,HLA-DRA,HLA-DRB1 |

**Table S3 Compared with the normal controls, significantly enriched pathways of each cell subtypes in the severe condition (patient 3). Cell type refers to Fig. 1d.**

| **Cluster** | **#term ID** | **term description** | **genes mapped** | **enrichment score** | **direction** | **false discovery rate** | **method** | **matching proteins in your input (IDs)** | **matching proteins in your input (labels)** |
| --- | --- | --- | --- | --- | --- | --- | --- | --- | --- |
| Cluster1 | CL:14978 | Peptide chain elongation | 5 | 6.22703 | top of list | 0.0022 | afc | ENSP00000311430,ENSP00000341885,ENSP00000346001,ENSP00000348849,ENSP00000362744 | RPL3,RPL4,RPS2,RPS26,RPS4X |
| Cluster1 | CL:22328 | respirasome | 6 | 5.75464 | top of list | 0.0022 | afc | ENSP00000354728,ENSP00000354813,ENSP00000354961,ENSP00000354982,ENSP00000355046,ENSP00000355206 | MT-CO3,MT-ND2,MT-ND3,MT-ND4,MT-ND4L,MT-ND5 |
| Cluster1 | CL:4665 | Interferon alpha/beta signaling | 14 | 4.54494 | bottom of list | 0.0032 | afc | ENSP00000252593,ENSP00000306565,ENSP00000333657,ENSP00000342513,ENSP00000354822,ENSP00000359787,ENSP00000360883,ENSP00000368699,ENSP00000380697,ENSP00000381601,ENSP00000382707,ENSP00000386187,ENSP00000388001,ENSP00000484689 | BST2,IFI44L,IFI6,IFIT3,IFITM1,IFITM2,IFITM3,IRF7,ISG15,ISG20,MX1,MX2,OAS1,XAF1 |
| Cluster1 | CL:14985 | Viral mRNA Translation | 4 | 6.49385 | top of list | 0.0032 | afc | ENSP00000341885,ENSP00000346001,ENSP00000348849,ENSP00000362744 | RPL3,RPS2,RPS26,RPS4X |
| Cluster1 | CL:4662 | Interferon alpha/beta signaling, and Interferon-induced protein 44 family | 15 | 4.27788 | bottom of list | 0.0039 | afc | ENSP00000252593,ENSP00000306565,ENSP00000318982,ENSP00000333657,ENSP00000342513,ENSP00000354822,ENSP00000359787,ENSP00000360883,ENSP00000368699,ENSP00000380697,ENSP00000381601,ENSP00000382707,ENSP00000386187,ENSP00000388001,ENSP00000484689 | BST2,EPSTI1,IFI44L,IFI6,IFIT3,IFITM1,IFITM2,IFITM3,IRF7,ISG15,ISG20,MX1,MX2,OAS1,XAF1 |
| Cluster1 | CL:4661 | Interferon alpha/beta signaling, and Interferon gamma signaling | 17 | 3.97711 | bottom of list | 0.0041 | afc | ENSP00000245185,ENSP00000252593,ENSP00000306565,ENSP00000318982,ENSP00000333657,ENSP00000342513,ENSP00000354822,ENSP00000359787,ENSP00000360883,ENSP00000368699,ENSP00000369299,ENSP00000380697,ENSP00000381601,ENSP00000382707,ENSP00000386187,ENSP00000388001,ENSP00000484689 | BST2,EPSTI1,IFI44L,IFI6,IFIT3,IFITM1,IFITM2,IFITM3,IRF7,ISG15,ISG20,MT2A,MX1,MX2,OAS1,TRIM22,XAF1 |
| Cluster1 | CL:22332 | mitochondrial respiratory chain complex I | 4 | 5.59437 | top of list | 0.0053 | afc | ENSP00000354728,ENSP00000354813,ENSP00000355046,ENSP00000355206 | MT-ND2,MT-ND3,MT-ND4L,MT-ND5 |
| Cluster | #term ID | term description | genes mapped | enrichment score | direction | false discovery rate | method | matching proteins in your input (IDs) | matching proteins in your input (labels) |
| Cluster2 | CL:22327 | Oxidative phosphorylation | 12 | 7.62107 | top of list | 5.20E-07 | afc | ENSP00000354499,ENSP00000354554,ENSP00000354632,ENSP00000354687,ENSP00000354728,ENSP00000354813,ENSP00000354961,ENSP00000354982,ENSP00000355046,ENSP00000355206,ENSP00000355265,ENSP00000429690 | ATP6V0E1,MT-ATP6,MT-ATP8,MT-CO1,MT-CO3,MT-CYB,MT-ND1,MT-ND2,MT-ND3,MT-ND4,MT-ND4L,MT-ND5 |
| Cluster2 | CL:22328 | respirasome | 10 | 8.51013 | top of list | 5.20E-07 | afc | ENSP00000354499,ENSP00000354554,ENSP00000354632,ENSP00000354687,ENSP00000354728,ENSP00000354813,ENSP00000354961,ENSP00000354982,ENSP00000355046,ENSP00000355206 | MT-ATP6,MT-CO1,MT-CO3,MT-CYB,MT-ND1,MT-ND2,MT-ND3,MT-ND4,MT-ND4L,MT-ND5 |
| Cluster2 | CL:14978 | Peptide chain elongation | 10 | 6.97159 | top of list | 2.20E-05 | afc | ENSP00000272317,ENSP00000307889,ENSP00000311430,ENSP00000341885,ENSP00000346001,ENSP00000346027,ENSP00000348849,ENSP00000362744,ENSP00000375730,ENSP00000470972 | RPL13,RPL13A,RPL21,RPL3,RPL4,RPS19,RPS2,RPS26,RPS27A,RPS4X |
| Cluster2 | CL:4665 | Interferon alpha/beta signaling | 20 | 4.15235 | bottom of list | 3.74E-05 | afc | ENSP00000252593,ENSP00000257570,ENSP00000306565,ENSP00000326247,ENSP00000333657,ENSP00000342513,ENSP00000354822,ENSP00000359783,ENSP00000359787,ENSP00000360869,ENSP00000360883,ENSP00000368699,ENSP00000380697,ENSP00000381601,ENSP00000382707,ENSP00000386187,ENSP00000388001,ENSP00000395590,ENSP00000483430,ENSP00000484689 | BST2,IFI27,IFI35,IFI44,IFI44L,IFI6,IFIT1,IFIT3,IFITM1,IFITM2,IFITM3,IRF7,ISG15,ISG20,MX1,MX2,OAS1,OASL,SAMD9L,XAF1 |
| Cluster2 | CL:14985 | Viral mRNA Translation | 9 | 7.03886 | top of list | 4.16E-05 | afc | ENSP00000272317,ENSP00000307889,ENSP00000341885,ENSP00000346001,ENSP00000346027,ENSP00000348849,ENSP00000362744,ENSP00000375730,ENSP00000470972 | RPL13,RPL13A,RPL21,RPL3,RPS19,RPS2,RPS26,RPS27A,RPS4X |
| Cluster2 | CL:22502 | Leber hereditary optic neuropathy | 5 | 8.70835 | top of list | 0.0001 | afc | ENSP00000354499,ENSP00000354554,ENSP00000354632,ENSP00000354961,ENSP00000354982 | MT-ATP6,MT-CO1,MT-CO3,MT-CYB,MT-ND4 |
| Cluster2 | CL:22332 | mitochondrial respiratory chain complex I | 5 | 8.31191 | top of list | 0.00033 | afc | ENSP00000354687,ENSP00000354728,ENSP00000354813,ENSP00000355046,ENSP00000355206 | MT-ND1,MT-ND2,MT-ND3,MT-ND4L,MT-ND5 |
| Cluster2 | CL:4668 | type I interferon signaling pathway | 16 | 3.91787 | bottom of list | 0.00047 | afc | ENSP00000252593,ENSP00000257570,ENSP00000306565,ENSP00000333657,ENSP00000342513,ENSP00000354822,ENSP00000360869,ENSP00000360883,ENSP00000380697,ENSP00000381601,ENSP00000382707,ENSP00000386187,ENSP00000388001,ENSP00000395590,ENSP00000483430,ENSP00000484689 | BST2,IFI27,IFI35,IFI6,IFIT1,IFIT3,IFITM1,IFITM2,IFITM3,IRF7,ISG20,MX1,MX2,OAS1,OASL,XAF1 |
| Cluster2 | CL:4670 | type I interferon signaling pathway | 15 | 3.98531 | bottom of list | 0.00069 | afc | ENSP00000257570,ENSP00000306565,ENSP00000333657,ENSP00000342513,ENSP00000354822,ENSP00000360869,ENSP00000360883,ENSP00000380697,ENSP00000381601,ENSP00000382707,ENSP00000386187,ENSP00000388001,ENSP00000395590,ENSP00000483430,ENSP00000484689 | IFI27,IFI35,IFI6,IFIT1,IFIT3,IFITM1,IFITM2,IFITM3,IRF7,ISG20,MX1,MX2,OAS1,OASL,XAF1 |
| Cluster2 | CL:22408 | Leber hereditary optic neuropathy | 4 | 8.17935 | top of list | 0.0016 | afc | ENSP00000354687,ENSP00000354728,ENSP00000354813,ENSP00000355046 | MT-ND1,MT-ND2,MT-ND4L,MT-ND5 |
| Cluster2 | CL:4661 | Interferon alpha/beta signaling, and Interferon gamma signaling | 24 | 3.90813 | bottom of list | 0.0041 | ks | ENSP00000245185,ENSP00000252593,ENSP00000257570,ENSP00000306565,ENSP00000318982,ENSP00000326247,ENSP00000333657,ENSP00000342513,ENSP00000345494,ENSP00000354822,ENSP00000359783,ENSP00000359787,ENSP00000360869,ENSP00000360883,ENSP00000368699,ENSP00000369299,ENSP00000380697,ENSP00000381601,ENSP00000382707,ENSP00000386187,ENSP00000388001,ENSP00000395590,ENSP00000483430,ENSP00000484689 | BST2,EPSTI1,IFI27,IFI35,IFI44,IFI44L,IFI6,IFIT1,IFIT3,IFITM1,IFITM2,IFITM3,IRF7,ISG15,ISG20,MT2A,MX1,MX2,OAS1,OASL,PLSCR1,SAMD9L,TRIM22,XAF1 |
| **Cluster** | **#term ID** | **term description** | **genes mapped** | **enrichment score** | **direction** | **false discovery rate** | **method** | **matching proteins in your input (IDs)** | **matching proteins in your input (labels)** |
| Cluster2 | CL:4662 | Interferon alpha/beta signaling, and Interferon-induced protein 44 family | 22 | 3.94292 | bottom of list | 0.0044 | ks | ENSP00000252593,ENSP00000257570,ENSP00000306565,ENSP00000318982,ENSP00000326247,ENSP00000333657,ENSP00000342513,ENSP00000345494,ENSP00000354822,ENSP00000359783,ENSP00000359787,ENSP00000360869,ENSP00000360883,ENSP00000368699,ENSP00000380697,ENSP00000381601,ENSP00000382707,ENSP00000386187,ENSP00000388001,ENSP00000395590,ENSP00000483430,ENSP00000484689 | BST2,EPSTI1,IFI27,IFI35,IFI44,IFI44L,IFI6,IFIT1,IFIT3,IFITM1,IFITM2,IFITM3,IRF7,ISG15,ISG20,MX1,MX2,OAS1,OASL,PLSCR1,SAMD9L,XAF1 |
| Cluster2 | CL:4659 | Interferon alpha/beta signaling, and Interferon gamma signaling | 25 | 3.77404 | bottom of list | 0.0064 | ks | ENSP00000245185,ENSP00000252593,ENSP00000257570,ENSP00000258381,ENSP00000306565,ENSP00000318982,ENSP00000326247,ENSP00000333657,ENSP00000342513,ENSP00000345494,ENSP00000354822,ENSP00000359783,ENSP00000359787,ENSP00000360869,ENSP00000360883,ENSP00000368699,ENSP00000369299,ENSP00000380697,ENSP00000381601,ENSP00000382707,ENSP00000386187,ENSP00000388001,ENSP00000395590,ENSP00000483430,ENSP00000484689 | BST2,EPSTI1,IFI27,IFI35,IFI44,IFI44L,IFI6,IFIT1,IFIT3,IFITM1,IFITM2,IFITM3,IRF7,ISG15,ISG20,MT2A,MX1,MX2,OAS1,OASL,PLSCR1,SAMD9L,SP110,TRIM22,XAF1 |
| Cluster2 | CL:4673 | 2'-5'-oligoadenylate synthase, and type I interferon signaling pathway | 10 | 3.84624 | bottom of list | 0.0071 | afc | ENSP00000257570,ENSP00000333657,ENSP00000342513,ENSP00000354822,ENSP00000360869,ENSP00000360883,ENSP00000380697,ENSP00000381601,ENSP00000388001,ENSP00000395590 | IFI35,IFI6,IFIT1,IFIT3,IRF7,MX1,MX2,OAS1,OASL,XAF1 |
| Cluster | #term ID | term description | genes mapped | enrichment score | direction | false discovery rate | method | matching proteins in your input (IDs) | matching proteins in your input (labels) |
| Cluster3 | CL:22328 | respirasome | 12 | 7.39261 | top of list | 3.20E-07 | afc | ENSP00000354499,ENSP00000354554,ENSP00000354632,ENSP00000354687,ENSP00000354728,ENSP00000354813,ENSP00000354876,ENSP00000354961,ENSP00000354982,ENSP00000355046,ENSP00000355206,ENSP00000418438 | MT-ATP6,MT-CO1,MT-CO2,MT-CO3,MT-CYB,MT-ND1,MT-ND2,MT-ND3,MT-ND4,MT-ND4L,MT-ND5,NDUFA3 |
| Cluster3 | CL:22327 | Oxidative phosphorylation | 14 | 6.77967 | top of list | 3.20E-07 | afc | ENSP00000354499,ENSP00000354554,ENSP00000354632,ENSP00000354687,ENSP00000354728,ENSP00000354813,ENSP00000354876,ENSP00000354961,ENSP00000354982,ENSP00000355046,ENSP00000355206,ENSP00000355265,ENSP00000418438,ENSP00000429690 | ATP6V0E1,MT-ATP6,MT-ATP8,MT-CO1,MT-CO2,MT-CO3,MT-CYB,MT-ND1,MT-ND2,MT-ND3,MT-ND4,MT-ND4L,MT-ND5,NDUFA3 |
| Cluster3 | CL:22446 | cytochrome complex, and Cytochrome c oxidase subunit VII | 6 | 8.49832 | top of list | 4.00E-06 | afc | ENSP00000354499,ENSP00000354554,ENSP00000354632,ENSP00000354876,ENSP00000354961,ENSP00000354982 | MT-ATP6,MT-CO1,MT-CO2,MT-CO3,MT-CYB,MT-ND4 |
| Cluster3 | CL:22502 | Leber hereditary optic neuropathy | 5 | 8.94176 | top of list | 4.00E-06 | afc | ENSP00000354499,ENSP00000354554,ENSP00000354632,ENSP00000354961,ENSP00000354982 | MT-ATP6,MT-CO1,MT-CO3,MT-CYB,MT-ND4 |
| Cluster3 | CL:4665 | Interferon alpha/beta signaling | 20 | 3.59162 | bottom of list | 0.00024 | afc | ENSP00000252593,ENSP00000257570,ENSP00000306565,ENSP00000326247,ENSP00000333657,ENSP00000342513,ENSP00000354822,ENSP00000359787,ENSP00000360869,ENSP00000360883,ENSP00000368699,ENSP00000371471,ENSP00000380697,ENSP00000381601,ENSP00000382707,ENSP00000386187,ENSP00000388001,ENSP00000395590,ENSP00000483430,ENSP00000484689 | BST2,IFI27,IFI35,IFI44L,IFI6,IFIT1,IFIT3,IFITM1,IFITM2,IFITM3,IRF7,ISG15,ISG20,MX1,MX2,OAS1,OASL,RSAD2,SAMD9L,XAF1 |
| Cluster3 | CL:22332 | mitochondrial respiratory chain complex I | 6 | 6.2869 | top of list | 0.00098 | afc | ENSP00000354687,ENSP00000354728,ENSP00000354813,ENSP00000355046,ENSP00000355206,ENSP00000418438 | MT-ND1,MT-ND2,MT-ND3,MT-ND4L,MT-ND5,NDUFA3 |
| Cluster3 | CL:4668 | type I interferon signaling pathway | 17 | 3.32974 | bottom of list | 0.0018 | afc | ENSP00000252593,ENSP00000257570,ENSP00000306565,ENSP00000333657,ENSP00000342513,ENSP00000354822,ENSP00000360869,ENSP00000360883,ENSP00000371471,ENSP00000380697,ENSP00000381601,ENSP00000382707,ENSP00000386187,ENSP00000388001,ENSP00000395590,ENSP00000483430,ENSP00000484689 | BST2,IFI27,IFI35,IFI6,IFIT1,IFIT3,IFITM1,IFITM2,IFITM3,IRF7,ISG20,MX1,MX2,OAS1,OASL,RSAD2,XAF1 |
| Cluster3 | CL:22408 | Leber hereditary optic neuropathy | 4 | 7.59808 | top of list | 0.0018 | afc | ENSP00000354687,ENSP00000354728,ENSP00000354813,ENSP00000355046 | MT-ND1,MT-ND2,MT-ND4L,MT-ND5 |
| Cluster3 | CL:4670 | type I interferon signaling pathway | 16 | 3.29154 | bottom of list | 0.0023 | afc | ENSP00000257570,ENSP00000306565,ENSP00000333657,ENSP00000342513,ENSP00000354822,ENSP00000360869,ENSP00000360883,ENSP00000371471,ENSP00000380697,ENSP00000381601,ENSP00000382707,ENSP00000386187,ENSP00000388001,ENSP00000395590,ENSP00000483430,ENSP00000484689 | IFI27,IFI35,IFI6,IFIT1,IFIT3,IFITM1,IFITM2,IFITM3,IRF7,ISG20,MX1,MX2,OAS1,OASL,RSAD2,XAF1 |
| Cluster | #term ID | term description | genes mapped | enrichment score | direction | false discovery rate | method | matching proteins in your input (IDs) | matching proteins in your input (labels) |
| Cluster5 | CL:14983 | Peptide chain elongation | 7 | 5.48774 | top of list | 0.0015 | afc | ENSP00000307940,ENSP00000346001,ENSP00000348849,ENSP00000397798,ENSP00000403172,ENSP00000404375,ENSP00000463784 | EEF2,RPL17,RPL26,RPL3,RPL36A,RPL6,RPS26 |
| Cluster5 | CL:14978 | Peptide chain elongation | 8 | 5.35349 | top of list | 0.0015 | afc | ENSP00000307940,ENSP00000311430,ENSP00000346001,ENSP00000348849,ENSP00000397798,ENSP00000403172,ENSP00000404375,ENSP00000463784 | EEF2,RPL17,RPL26,RPL3,RPL36A,RPL4,RPL6,RPS26 |
| Cluster5 | CL:14985 | Viral mRNA Translation | 5 | 5.69969 | top of list | 0.0037 | afc | ENSP00000346001,ENSP00000348849,ENSP00000397798,ENSP00000403172,ENSP00000463784 | RPL17,RPL26,RPL3,RPL6,RPS26 |
| Cluster5 | CL:18627 | MHC class II protein complex, and dynactin complex | 4 | 5.49373 | both ends | 0.0064 | afc | ENSP00000353099,ENSP00000364076,ENSP00000364080,ENSP00000364114 | HLA-DQA2,HLA-DQB1,HLA-DRB1,HLA-DRB5 |
| Cluster | #term ID | term description | genes mapped | enrichment score | direction | false discovery rate | method | matching proteins in your input (IDs) | matching proteins in your input (labels) |
| Cluster6 | CL:4665 | Interferon alpha/beta signaling | 19 | 4.68938 | bottom of list | 5.29E-05 | afc | ENSP00000252593,ENSP00000306565,ENSP00000326247,ENSP00000333657,ENSP00000342513,ENSP00000354822,ENSP00000359787,ENSP00000360869,ENSP00000360883,ENSP00000368699,ENSP00000371471,ENSP00000380697,ENSP00000381601,ENSP00000382707,ENSP00000386187,ENSP00000388001,ENSP00000395590,ENSP00000483430,ENSP00000484689 | BST2,IFI27,IFI35,IFI44L,IFI6,IFIT1,IFIT3,IFITM1,IFITM2,IFITM3,IRF7,ISG15,ISG20,MX1,MX2,OAS1,RSAD2,SAMD9L,XAF1 |
| Cluster6 | CL:22328 | respirasome | 9 | 7.88692 | top of list | 5.29E-05 | afc | ENSP00000354499,ENSP00000354554,ENSP00000354632,ENSP00000354728,ENSP00000354813,ENSP00000354961,ENSP00000354982,ENSP00000355046,ENSP00000355206 | MT-ATP6,MT-CO1,MT-CO3,MT-CYB,MT-ND2,MT-ND3,MT-ND4,MT-ND4L,MT-ND5 |
| Cluster6 | CL:22327 | Oxidative phosphorylation | 10 | 6.93916 | top of list | 0.0001 | afc | ENSP00000354499,ENSP00000354554,ENSP00000354632,ENSP00000354728,ENSP00000354813,ENSP00000354961,ENSP00000354982,ENSP00000355046,ENSP00000355206,ENSP00000429690 | ATP6V0E1,MT-ATP6,MT-CO1,MT-CO3,MT-CYB,MT-ND2,MT-ND3,MT-ND4,MT-ND4L,MT-ND5 |
| Cluster6 | CL:4668 | type I interferon signaling pathway | 16 | 4.38358 | bottom of list | 0.00063 | afc | ENSP00000252593,ENSP00000306565,ENSP00000333657,ENSP00000342513,ENSP00000354822,ENSP00000360869,ENSP00000360883,ENSP00000371471,ENSP00000380697,ENSP00000381601,ENSP00000382707,ENSP00000386187,ENSP00000388001,ENSP00000395590,ENSP00000483430,ENSP00000484689 | BST2,IFI27,IFI35,IFI6,IFIT1,IFIT3,IFITM1,IFITM2,IFITM3,IRF7,ISG20,MX1,MX2,OAS1,RSAD2,XAF1 |
| Cluster6 | CL:4670 | type I interferon signaling pathway | 15 | 4.38906 | bottom of list | 0.00065 | afc | ENSP00000306565,ENSP00000333657,ENSP00000342513,ENSP00000354822,ENSP00000360869,ENSP00000360883,ENSP00000371471,ENSP00000380697,ENSP00000381601,ENSP00000382707,ENSP00000386187,ENSP00000388001,ENSP00000395590,ENSP00000483430,ENSP00000484689 | IFI27,IFI35,IFI6,IFIT1,IFIT3,IFITM1,IFITM2,IFITM3,IRF7,ISG20,MX1,MX2,OAS1,RSAD2,XAF1 |
| Cluster6 | CL:22502 | Leber hereditary optic neuropathy | 5 | 8.01823 | top of list | 0.0025 | afc | ENSP00000354499,ENSP00000354554,ENSP00000354632,ENSP00000354961,ENSP00000354982 | MT-ATP6,MT-CO1,MT-CO3,MT-CYB,MT-ND4 |
| Cluster6 | CL:4661 | Interferon alpha/beta signaling, and Interferon gamma signaling | 23 | 4.39925 | bottom of list | 0.007 | ks | ENSP00000245185,ENSP00000252593,ENSP00000306565,ENSP00000318982,ENSP00000326247,ENSP00000333657,ENSP00000342513,ENSP00000345494,ENSP00000354822,ENSP00000359787,ENSP00000360869,ENSP00000360883,ENSP00000368699,ENSP00000369299,ENSP00000371471,ENSP00000380697,ENSP00000381601,ENSP00000382707,ENSP00000386187,ENSP00000388001,ENSP00000395590,ENSP00000483430,ENSP00000484689 | BST2,EPSTI1,IFI27,IFI35,IFI44L,IFI6,IFIT1,IFIT3,IFITM1,IFITM2,IFITM3,IRF7,ISG15,ISG20,MT2A,MX1,MX2,OAS1,PLSCR1,RSAD2,SAMD9L,TRIM22,XAF1 |
| Cluster6 | CL:22332 | mitochondrial respiratory chain complex I | 4 | 7.72278 | top of list | 0.0086 | afc | ENSP00000354728,ENSP00000354813,ENSP00000355046,ENSP00000355206 | MT-ND2,MT-ND3,MT-ND4L,MT-ND5 |
| Cluster | #term ID | term description | genes mapped | enrichment score | direction | false discovery rate | method | matching proteins in your input (IDs) | matching proteins in your input (labels) |
| Cluster7 | CL:22328 | respirasome | 11 | 8.54514 | top of list | 3.60E-07 | afc | ENSP00000354499,ENSP00000354554,ENSP00000354632,ENSP00000354687,ENSP00000354728,ENSP00000354813,ENSP00000354876,ENSP00000354961,ENSP00000354982,ENSP00000355046,ENSP00000355206 | MT-ATP6,MT-CO1,MT-CO2,MT-CO3,MT-CYB,MT-ND1,MT-ND2,MT-ND3,MT-ND4,MT-ND4L,MT-ND5 |
| Cluster7 | CL:22327 | Oxidative phosphorylation | 13 | 7.72921 | top of list | 3.60E-07 | afc | ENSP00000354499,ENSP00000354554,ENSP00000354632,ENSP00000354687,ENSP00000354728,ENSP00000354813,ENSP00000354876,ENSP00000354961,ENSP00000354982,ENSP00000355046,ENSP00000355206,ENSP00000355265,ENSP00000429690 | ATP6V0E1,MT-ATP6,MT-ATP8,MT-CO1,MT-CO2,MT-CO3,MT-CYB,MT-ND1,MT-ND2,MT-ND3,MT-ND4,MT-ND4L,MT-ND5 |
| Cluster7 | CL:22446 | cytochrome complex, and Cytochrome c oxidase subunit VII | 6 | 9.16274 | top of list | 8.64E-06 | afc | ENSP00000354499,ENSP00000354554,ENSP00000354632,ENSP00000354876,ENSP00000354961,ENSP00000354982 | MT-ATP6,MT-CO1,MT-CO2,MT-CO3,MT-CYB,MT-ND4 |
| Cluster7 | CL:22502 | Leber hereditary optic neuropathy | 5 | 9.443 | top of list | 1.55E-05 | afc | ENSP00000354499,ENSP00000354554,ENSP00000354632,ENSP00000354961,ENSP00000354982 | MT-ATP6,MT-CO1,MT-CO3,MT-CYB,MT-ND4 |
| Cluster7 | CL:4665 | Interferon alpha/beta signaling | 19 | 4.3293 | bottom of list | 2.39E-05 | afc | ENSP00000252593,ENSP00000257570,ENSP00000306565,ENSP00000326247,ENSP00000333657,ENSP00000342513,ENSP00000354822,ENSP00000359787,ENSP00000360869,ENSP00000360883,ENSP00000368699,ENSP00000371471,ENSP00000380697,ENSP00000381601,ENSP00000382707,ENSP00000386187,ENSP00000388001,ENSP00000395590,ENSP00000483430 | BST2,IFI27,IFI35,IFI44L,IFI6,IFIT1,IFIT3,IFITM1,IFITM3,IRF7,ISG15,ISG20,MX1,MX2,OAS1,OASL,RSAD2,SAMD9L,XAF1 |
| Cluster7 | CL:4668 | type I interferon signaling pathway | 16 | 4.1009 | bottom of list | 0.00038 | afc | ENSP00000252593,ENSP00000257570,ENSP00000306565,ENSP00000333657,ENSP00000342513,ENSP00000354822,ENSP00000360869,ENSP00000360883,ENSP00000371471,ENSP00000380697,ENSP00000381601,ENSP00000382707,ENSP00000386187,ENSP00000388001,ENSP00000395590,ENSP00000483430 | BST2,IFI27,IFI35,IFI6,IFIT1,IFIT3,IFITM1,IFITM3,IRF7,ISG20,MX1,MX2,OAS1,OASL,RSAD2,XAF1 |
| Cluster7 | CL:4670 | type I interferon signaling pathway | 15 | 4.07699 | bottom of list | 0.00072 | afc | ENSP00000257570,ENSP00000306565,ENSP00000333657,ENSP00000342513,ENSP00000354822,ENSP00000360869,ENSP00000360883,ENSP00000371471,ENSP00000380697,ENSP00000381601,ENSP00000382707,ENSP00000386187,ENSP00000388001,ENSP00000395590,ENSP00000483430 | IFI27,IFI35,IFI6,IFIT1,IFIT3,IFITM1,IFITM3,IRF7,ISG20,MX1,MX2,OAS1,OASL,RSAD2,XAF1 |
| Cluster7 | CL:4662 | Interferon alpha/beta signaling, and Interferon-induced protein 44 family | 21 | 4.18294 | bottom of list | 0.0011 | ks | ENSP00000252593,ENSP00000257570,ENSP00000306565,ENSP00000318982,ENSP00000326247,ENSP00000333657,ENSP00000342513,ENSP00000345494,ENSP00000354822,ENSP00000359787,ENSP00000360869,ENSP00000360883,ENSP00000368699,ENSP00000371471,ENSP00000380697,ENSP00000381601,ENSP00000382707,ENSP00000386187,ENSP00000388001,ENSP00000395590,ENSP00000483430 | BST2,EPSTI1,IFI27,IFI35,IFI44L,IFI6,IFIT1,IFIT3,IFITM1,IFITM3,IRF7,ISG15,ISG20,MX1,MX2,OAS1,OASL,PLSCR1,RSAD2,SAMD9L,XAF1 |
| Cluster7 | CL:22332 | mitochondrial respiratory chain complex I | 5 | 7.80401 | top of list | 0.0011 | afc | ENSP00000354687,ENSP00000354728,ENSP00000354813,ENSP00000355046,ENSP00000355206 | MT-ND1,MT-ND2,MT-ND3,MT-ND4L,MT-ND5 |
| Cluster7 | CL:4661 | Interferon alpha/beta signaling, and Interferon gamma signaling | 24 | 3.96209 | bottom of list | 0.0026 | ks | ENSP00000245185,ENSP00000252593,ENSP00000257570,ENSP00000306565,ENSP00000318982,ENSP00000326247,ENSP00000333657,ENSP00000342513,ENSP00000345494,ENSP00000354822,ENSP00000359504,ENSP00000359787,ENSP00000360869,ENSP00000360883,ENSP00000368699,ENSP00000369299,ENSP00000371471,ENSP00000380697,ENSP00000381601,ENSP00000382707,ENSP00000386187,ENSP00000388001,ENSP00000395590,ENSP00000483430 | BST2,EPSTI1,GBP1,IFI27,IFI35,IFI44L,IFI6,IFIT1,IFIT3,IFITM1,IFITM3,IRF7,ISG15,ISG20,MT2A,MX1,MX2,OAS1,OASL,PLSCR1,RSAD2,SAMD9L,TRIM22,XAF1 |
| **Cluster** | **#term ID** | **term description** | **genes mapped** | **enrichment score** | **direction** | **false discovery rate** | **method** | **matching proteins in your input (IDs)** | **matching proteins in your input (labels)** |
| Cluster7 | CL:4659 | Interferon alpha/beta signaling, and Interferon gamma signaling | 25 | 3.88407 | bottom of list | 0.003 | ks | ENSP00000245185,ENSP00000252593,ENSP00000257570,ENSP00000258381,ENSP00000306565,ENSP00000318982,ENSP00000326247,ENSP00000333657,ENSP00000342513,ENSP00000345494,ENSP00000354822,ENSP00000359504,ENSP00000359787,ENSP00000360869,ENSP00000360883,ENSP00000368699,ENSP00000369299,ENSP00000371471,ENSP00000380697,ENSP00000381601,ENSP00000382707,ENSP00000386187,ENSP00000388001,ENSP00000395590,ENSP00000483430 | BST2,EPSTI1,GBP1,IFI27,IFI35,IFI44L,IFI6,IFIT1,IFIT3,IFITM1,IFITM3,IRF7,ISG15,ISG20,MT2A,MX1,MX2,OAS1,OASL,PLSCR1,RSAD2,SAMD9L,SP110,TRIM22,XAF1 |
| Cluster7 | CL:22408 | Leber hereditary optic neuropathy | 4 | 7.50923 | top of list | 0.0046 | afc | ENSP00000354687,ENSP00000354728,ENSP00000354813,ENSP00000355046 | MT-ND1,MT-ND2,MT-ND4L,MT-ND5 |
| Cluster7 | CL:4673 | 2'-5'-oligoadenylate synthase, and type I interferon signaling pathway | 11 | 3.98162 | bottom of list | 0.0047 | afc | ENSP00000257570,ENSP00000333657,ENSP00000342513,ENSP00000354822,ENSP00000360869,ENSP00000360883,ENSP00000371471,ENSP00000380697,ENSP00000381601,ENSP00000388001,ENSP00000395590 | IFI35,IFI6,IFIT1,IFIT3,IRF7,MX1,MX2,OAS1,OASL,RSAD2,XAF1 |
| Cluster | #term ID | term description | genes mapped | enrichment score | direction | false discovery rate | method | matching proteins in your input (IDs) | matching proteins in your input (labels) |
| Cluster8 | CL:22328 | respirasome | 8 | 7.46334 | top of list | 7.44E-05 | afc | ENSP00000354554,ENSP00000354632,ENSP00000354728,ENSP00000354813,ENSP00000354961,ENSP00000354982,ENSP00000355046,ENSP00000355206 | MT-ATP6,MT-CO3,MT-CYB,MT-ND2,MT-ND3,MT-ND4,MT-ND4L,MT-ND5 |
| Cluster8 | CL:4665 | Interferon alpha/beta signaling | 15 | 4.98605 | bottom of list | 0.00015 | afc | ENSP00000252593,ENSP00000306565,ENSP00000333657,ENSP00000342513,ENSP00000354822,ENSP00000359787,ENSP00000360883,ENSP00000368699,ENSP00000380697,ENSP00000381601,ENSP00000382707,ENSP00000386187,ENSP00000388001,ENSP00000483430,ENSP00000484689 | BST2,IFI27,IFI44L,IFI6,IFIT3,IFITM1,IFITM2,IFITM3,IRF7,ISG15,ISG20,MX1,MX2,OAS1,XAF1 |
| Cluster8 | CL:4661 | Interferon alpha/beta signaling, and Interferon gamma signaling | 19 | 4.34545 | bottom of list | 0.00022 | afc | ENSP00000245185,ENSP00000252593,ENSP00000306565,ENSP00000318982,ENSP00000333657,ENSP00000342513,ENSP00000345494,ENSP00000354822,ENSP00000359787,ENSP00000360883,ENSP00000368699,ENSP00000369299,ENSP00000380697,ENSP00000381601,ENSP00000382707,ENSP00000386187,ENSP00000388001,ENSP00000483430,ENSP00000484689 | BST2,EPSTI1,IFI27,IFI44L,IFI6,IFIT3,IFITM1,IFITM2,IFITM3,IRF7,ISG15,ISG20,MT2A,MX1,MX2,OAS1,PLSCR1,TRIM22,XAF1 |
| Cluster8 | CL:4662 | Interferon alpha/beta signaling, and Interferon-induced protein 44 family | 17 | 4.49694 | bottom of list | 0.00022 | afc | ENSP00000252593,ENSP00000306565,ENSP00000318982,ENSP00000333657,ENSP00000342513,ENSP00000345494,ENSP00000354822,ENSP00000359787,ENSP00000360883,ENSP00000368699,ENSP00000380697,ENSP00000381601,ENSP00000382707,ENSP00000386187,ENSP00000388001,ENSP00000483430,ENSP00000484689 | BST2,EPSTI1,IFI27,IFI44L,IFI6,IFIT3,IFITM1,IFITM2,IFITM3,IRF7,ISG15,ISG20,MX1,MX2,OAS1,PLSCR1,XAF1 |
| Cluster8 | CL:4670 | type I interferon signaling pathway | 12 | 4.54201 | bottom of list | 0.002 | afc | ENSP00000306565,ENSP00000333657,ENSP00000342513,ENSP00000354822,ENSP00000360883,ENSP00000380697,ENSP00000381601,ENSP00000382707,ENSP00000386187,ENSP00000388001,ENSP00000483430,ENSP00000484689 | IFI27,IFI6,IFIT3,IFITM1,IFITM2,IFITM3,IRF7,ISG20,MX1,MX2,OAS1,XAF1 |
| Cluster8 | CL:4668 | type I interferon signaling pathway | 13 | 4.3361 | bottom of list | 0.0027 | afc | ENSP00000252593,ENSP00000306565,ENSP00000333657,ENSP00000342513,ENSP00000354822,ENSP00000360883,ENSP00000380697,ENSP00000381601,ENSP00000382707,ENSP00000386187,ENSP00000388001,ENSP00000483430,ENSP00000484689 | BST2,IFI27,IFI6,IFIT3,IFITM1,IFITM2,IFITM3,IRF7,ISG20,MX1,MX2,OAS1,XAF1 |
| Cluster8 | CL:22332 | mitochondrial respiratory chain complex I | 4 | 7.37424 | top of list | 0.0038 | afc | ENSP00000354728,ENSP00000354813,ENSP00000355046,ENSP00000355206 | MT-ND2,MT-ND3,MT-ND4L,MT-ND5 |
| Cluster8 | CL:14982 | Peptide chain elongation | 4 | 7.44144 | top of list | 0.0038 | afc | ENSP00000341885,ENSP00000348849,ENSP00000375730,ENSP00000429374 | RPL13A,RPS2,RPS20,RPS26 |
| Cluster8 | CL:22502 | Leber hereditary optic neuropathy | 4 | 7.55244 | top of list | 0.0038 | afc | ENSP00000354554,ENSP00000354632,ENSP00000354961,ENSP00000354982 | MT-ATP6,MT-CO3,MT-CYB,MT-ND4 |
| Cluster | #term ID | term description | genes mapped | enrichment score | direction | false discovery rate | method | matching proteins in your input (IDs) | matching proteins in your input (labels) |
| Cluster9 | CL:4668 | type I interferon signaling pathway | 17 | 4.51439 | bottom of list | 0.00022 | afc | ENSP00000252593,ENSP00000257570,ENSP00000306565,ENSP00000333657,ENSP00000342278,ENSP00000342513,ENSP00000354822,ENSP00000360869,ENSP00000360883,ENSP00000371471,ENSP00000380697,ENSP00000381601,ENSP00000382707,ENSP00000386187,ENSP00000388001,ENSP00000395590,ENSP00000483430 | BST2,IFI27,IFI35,IFI6,IFIT1,IFIT3,IFITM1,IFITM3,IRF7,ISG20,MX1,MX2,OAS1,OAS2,OASL,RSAD2,XAF1 |
| Cluster9 | CL:22327 | Oxidative phosphorylation | 13 | 4.98071 | top of list | 0.00022 | afc | ENSP00000354499,ENSP00000354554,ENSP00000354632,ENSP00000354687,ENSP00000354728,ENSP00000354813,ENSP00000354876,ENSP00000354961,ENSP00000354982,ENSP00000355046,ENSP00000355206,ENSP00000355265,ENSP00000429690 | ATP6V0E1,MT-ATP6,MT-ATP8,MT-CO1,MT-CO2,MT-CO3,MT-CYB,MT-ND1,MT-ND2,MT-ND3,MT-ND4,MT-ND4L,MT-ND5 |
| Cluster9 | CL:22328 | respirasome | 11 | 5.5775 | top of list | 0.00022 | afc | ENSP00000354499,ENSP00000354554,ENSP00000354632,ENSP00000354687,ENSP00000354728,ENSP00000354813,ENSP00000354876,ENSP00000354961,ENSP00000354982,ENSP00000355046,ENSP00000355206 | MT-ATP6,MT-CO1,MT-CO2,MT-CO3,MT-CYB,MT-ND1,MT-ND2,MT-ND3,MT-ND4,MT-ND4L,MT-ND5 |
| Cluster9 | CL:4670 | type I interferon signaling pathway | 16 | 4.44251 | bottom of list | 0.00032 | afc | ENSP00000257570,ENSP00000306565,ENSP00000333657,ENSP00000342278,ENSP00000342513,ENSP00000354822,ENSP00000360869,ENSP00000360883,ENSP00000371471,ENSP00000380697,ENSP00000381601,ENSP00000382707,ENSP00000386187,ENSP00000388001,ENSP00000395590,ENSP00000483430 | IFI27,IFI35,IFI6,IFIT1,IFIT3,IFITM1,IFITM3,IRF7,ISG20,MX1,MX2,OAS1,OAS2,OASL,RSAD2,XAF1 |
| **Cluster** | **#term ID** | **term description** | **genes mapped** | **enrichment score** | **direction** | **false discovery rate** | **method** | **matching proteins in your input (IDs)** | **matching proteins in your input (labels)** |
| Cluster9 | CL:22446 | cytochrome complex, and Cytochrome c oxidase subunit VII | 6 | 5.93476 | top of list | 0.005 | afc | ENSP00000354499,ENSP00000354554,ENSP00000354632,ENSP00000354876,ENSP00000354961,ENSP00000354982 | MT-ATP6,MT-CO1,MT-CO2,MT-CO3,MT-CYB,MT-ND4 |
| Cluster | #term ID | term description | genes mapped | enrichment score | direction | false discovery rate | method | matching proteins in your input (IDs) | matching proteins in your input (labels) |
| Cluster10 | CL:14978 | Peptide chain elongation | 18 | 6.46072 | top of list | 7.80E-06 | afc | ENSP00000272317,ENSP00000307940,ENSP00000309334,ENSP00000311430,ENSP00000339063,ENSP00000341885,ENSP00000346001,ENSP00000346027,ENSP00000346050,ENSP00000348849,ENSP00000359345,ENSP00000362744,ENSP00000369757,ENSP00000403172,ENSP00000404375,ENSP00000449328,ENSP00000452909,ENSP00000463784 | EEF1A1,EEF2,RPL15,RPL21,RPL26,RPL28,RPL3,RPL36A,RPL4,RPL5,RPL6,RPLP0,RPS2,RPS26,RPS27A,RPS3A,RPS4X,RPS6 |
| Cluster10 | CL:14966 | GTP hydrolysis and joining of the 60S ribosomal subunit, and Protein export | 24 | 6.68253 | top of list | 7.90E-06 | ks | ENSP00000272317,ENSP00000307940,ENSP00000309334,ENSP00000311430,ENSP00000313007,ENSP00000339063,ENSP00000341885,ENSP00000346001,ENSP00000346027,ENSP00000346050,ENSP00000348849,ENSP00000359345,ENSP00000362744,ENSP00000369757,ENSP00000376056,ENSP00000388806,ENSP00000403172,ENSP00000404375,ENSP00000419449,ENSP00000449328,ENSP00000452909,ENSP00000463784,ENSP00000477781,ENSP00000485663 | EEF1A1,EEF1B2,EEF2,EIF1,EIF3L,EIF4B,PABPC1,RPL15,RPL21,RPL26,RPL28,RPL3,RPL36A,RPL4,RPL5,RPL6,RPLP0,RPS2,RPS26,RPS27A,RPS3A,RPS4X,RPS6,TPT1 |
| Cluster10 | CL:14982 | Peptide chain elongation | 16 | 6.3552 | top of list | 1.95E-05 | afc | ENSP00000272317,ENSP00000307940,ENSP00000309334,ENSP00000341885,ENSP00000346001,ENSP00000346027,ENSP00000346050,ENSP00000348849,ENSP00000359345,ENSP00000362744,ENSP00000369757,ENSP00000403172,ENSP00000404375,ENSP00000449328,ENSP00000452909,ENSP00000463784 | EEF2,RPL15,RPL21,RPL26,RPL28,RPL3,RPL36A,RPL5,RPL6,RPLP0,RPS2,RPS26,RPS27A,RPS3A,RPS4X,RPS6 |
| Cluster10 | CL:14967 | GTP hydrolysis and joining of the 60S ribosomal subunit, and Protein export | 22 | 6.58499 | top of list | 1.95E-05 | ks | ENSP00000272317,ENSP00000307940,ENSP00000309334,ENSP00000311430,ENSP00000313007,ENSP00000339063,ENSP00000341885,ENSP00000346001,ENSP00000346027,ENSP00000346050,ENSP00000348849,ENSP00000359345,ENSP00000362744,ENSP00000369757,ENSP00000388806,ENSP00000403172,ENSP00000404375,ENSP00000419449,ENSP00000449328,ENSP00000452909,ENSP00000463784,ENSP00000485663 | EEF1A1,EEF2,EIF1,EIF3L,EIF4B,PABPC1,RPL15,RPL21,RPL26,RPL28,RPL3,RPL36A,RPL4,RPL5,RPL6,RPLP0,RPS2,RPS26,RPS27A,RPS3A,RPS4X,RPS6 |
| Cluster10 | CL:14983 | Peptide chain elongation | 13 | 6.7301 | top of list | 3.90E-05 | afc | ENSP00000272317,ENSP00000307940,ENSP00000309334,ENSP00000341885,ENSP00000346001,ENSP00000346027,ENSP00000346050,ENSP00000348849,ENSP00000362744,ENSP00000369757,ENSP00000403172,ENSP00000404375,ENSP00000463784 | EEF2,RPL15,RPL21,RPL26,RPL3,RPL36A,RPL6,RPS2,RPS26,RPS27A,RPS3A,RPS4X,RPS6 |
| Cluster10 | CL:14985 | Viral mRNA Translation | 11 | 6.65566 | top of list | 0.00013 | afc | ENSP00000272317,ENSP00000309334,ENSP00000341885,ENSP00000346001,ENSP00000346027,ENSP00000346050,ENSP00000348849,ENSP00000362744,ENSP00000369757,ENSP00000403172,ENSP00000463784 | RPL15,RPL21,RPL26,RPL3,RPL6,RPS2,RPS26,RPS27A,RPS3A,RPS4X,RPS6 |
| Cluster10 | CL:22328 | respirasome | 10 | 7.05017 | top of list | 0.00013 | afc | ENSP00000354499,ENSP00000354554,ENSP00000354632,ENSP00000354687,ENSP00000354728,ENSP00000354813,ENSP00000354961,ENSP00000354982,ENSP00000355046,ENSP00000355206 | MT-ATP6,MT-CO1,MT-CO3,MT-CYB,MT-ND1,MT-ND2,MT-ND3,MT-ND4,MT-ND4L,MT-ND5 |
| Cluster10 | CL:4670 | type I interferon signaling pathway | 17 | 2.25084 | bottom of list | 0.00051 | afc | ENSP00000228928,ENSP00000257570,ENSP00000306565,ENSP00000333657,ENSP00000342278,ENSP00000342513,ENSP00000354822,ENSP00000360883,ENSP00000360891,ENSP00000380697,ENSP00000381601,ENSP00000382707,ENSP00000386187,ENSP00000388001,ENSP00000395590,ENSP00000483430,ENSP00000484689 | IFI27,IFI35,IFI6,IFIT2,IFIT3,IFITM1,IFITM2,IFITM3,IRF7,ISG20,MX1,MX2,OAS1,OAS2,OAS3,OASL,XAF1 |
| Cluster10 | CL:4668 | type I interferon signaling pathway | 18 | 2.16483 | bottom of list | 0.00055 | afc | ENSP00000228928,ENSP00000252593,ENSP00000257570,ENSP00000306565,ENSP00000333657,ENSP00000342278,ENSP00000342513,ENSP00000354822,ENSP00000360883,ENSP00000360891,ENSP00000380697,ENSP00000381601,ENSP00000382707,ENSP00000386187,ENSP00000388001,ENSP00000395590,ENSP00000483430,ENSP00000484689 | BST2,IFI27,IFI35,IFI6,IFIT2,IFIT3,IFITM1,IFITM2,IFITM3,IRF7,ISG20,MX1,MX2,OAS1,OAS2,OAS3,OASL,XAF1 |
| Cluster10 | CL:4661 | Interferon alpha/beta signaling, and Interferon gamma signaling | 26 | 1.91314 | bottom of list | 0.0074 | ks | ENSP00000228928,ENSP00000245185,ENSP00000252593,ENSP00000257570,ENSP00000306565,ENSP00000318982,ENSP00000333657,ENSP00000342278,ENSP00000342513,ENSP00000345494,ENSP00000354822,ENSP00000358165,ENSP00000359504,ENSP00000359783,ENSP00000359787,ENSP00000360883,ENSP00000360891,ENSP00000368699,ENSP00000380697,ENSP00000381601,ENSP00000382707,ENSP00000386187,ENSP00000388001,ENSP00000395590,ENSP00000483430,ENSP00000484689 | BST2,EPSTI1,FCGR1A,GBP1,IFI27,IFI35,IFI44,IFI44L,IFI6,IFIT2,IFIT3,IFITM1,IFITM2,IFITM3,IRF7,ISG15,ISG20,MT2A,MX1,MX2,OAS1,OAS2,OAS3,OASL,PLSCR1,XAF1 |
| Cluster | #term ID | term description | genes mapped | enrichment score | direction | false discovery rate | method | matching proteins in your input (IDs) | matching proteins in your input (labels) |
| Cluster11 | CL:4662 | Interferon alpha/beta signaling, and Interferon-induced protein 44 family | 17 | 3.25498 | bottom of list | 1.04E-05 | afc | ENSP00000252593,ENSP00000306565,ENSP00000318982,ENSP00000342513,ENSP00000345494,ENSP00000354822,ENSP00000359787,ENSP00000360883,ENSP00000368699,ENSP00000371471,ENSP00000380697,ENSP00000381601,ENSP00000382707,ENSP00000386187,ENSP00000388001,ENSP00000483430,ENSP00000484689 | BST2,EPSTI1,IFI27,IFI44L,IFI6,IFIT3,IFITM1,IFITM2,IFITM3,IRF7,ISG15,ISG20,MX1,OAS1,PLSCR1,RSAD2,XAF1 |
| Cluster11 | CL:4661 | Interferon alpha/beta signaling, and Interferon gamma signaling | 19 | 3.12695 | bottom of list | 1.04E-05 | afc | ENSP00000245185,ENSP00000252593,ENSP00000306565,ENSP00000318982,ENSP00000342513,ENSP00000345494,ENSP00000354822,ENSP00000359787,ENSP00000360883,ENSP00000368699,ENSP00000369299,ENSP00000371471,ENSP00000380697,ENSP00000381601,ENSP00000382707,ENSP00000386187,ENSP00000388001,ENSP00000483430,ENSP00000484689 | BST2,EPSTI1,IFI27,IFI44L,IFI6,IFIT3,IFITM1,IFITM2,IFITM3,IRF7,ISG15,ISG20,MT2A,MX1,OAS1,PLSCR1,RSAD2,TRIM22,XAF1 |
| Cluster11 | CL:4659 | Interferon alpha/beta signaling, and Interferon gamma signaling | 20 | 3.04412 | bottom of list | 1.04E-05 | afc | ENSP00000245185,ENSP00000252593,ENSP00000258381,ENSP00000306565,ENSP00000318982,ENSP00000342513,ENSP00000345494,ENSP00000354822,ENSP00000359787,ENSP00000360883,ENSP00000368699,ENSP00000369299,ENSP00000371471,ENSP00000380697,ENSP00000381601,ENSP00000382707,ENSP00000386187,ENSP00000388001,ENSP00000483430,ENSP00000484689 | BST2,EPSTI1,IFI27,IFI44L,IFI6,IFIT3,IFITM1,IFITM2,IFITM3,IRF7,ISG15,ISG20,MT2A,MX1,OAS1,PLSCR1,RSAD2,SP110,TRIM22,XAF1 |
| **Cluster** | **#term ID** | **term description** | **genes mapped** | **enrichment score** | **direction** | **false discovery rate** | **method** | **matching proteins in your input (IDs)** | **matching proteins in your input (labels)** |
| Cluster11 | CL:4665 | Interferon alpha/beta signaling | 15 | 3.3598 | bottom of list | 1.83E-05 | afc | ENSP00000252593,ENSP00000306565,ENSP00000342513,ENSP00000354822,ENSP00000359787,ENSP00000360883,ENSP00000368699,ENSP00000371471,ENSP00000380697,ENSP00000381601,ENSP00000382707,ENSP00000386187,ENSP00000388001,ENSP00000483430,ENSP00000484689 | BST2,IFI27,IFI44L,IFI6,IFIT3,IFITM1,IFITM2,IFITM3,IRF7,ISG15,ISG20,MX1,OAS1,RSAD2,XAF1 |
| Cluster11 | CL:4668 | type I interferon signaling pathway | 13 | 3.33626 | bottom of list | 6.26E-05 | afc | ENSP00000252593,ENSP00000306565,ENSP00000342513,ENSP00000354822,ENSP00000360883,ENSP00000371471,ENSP00000380697,ENSP00000381601,ENSP00000382707,ENSP00000386187,ENSP00000388001,ENSP00000483430,ENSP00000484689 | BST2,IFI27,IFI6,IFIT3,IFITM1,IFITM2,IFITM3,IRF7,ISG20,MX1,OAS1,RSAD2,XAF1 |
| Cluster11 | CL:4670 | type I interferon signaling pathway | 12 | 3.46532 | bottom of list | 7.54E-05 | afc | ENSP00000306565,ENSP00000342513,ENSP00000354822,ENSP00000360883,ENSP00000371471,ENSP00000380697,ENSP00000381601,ENSP00000382707,ENSP00000386187,ENSP00000388001,ENSP00000483430,ENSP00000484689 | IFI27,IFI6,IFIT3,IFITM1,IFITM2,IFITM3,IRF7,ISG20,MX1,OAS1,RSAD2,XAF1 |
| Cluster11 | CL:22502 | Leber hereditary optic neuropathy | 5 | 7.82738 | top of list | 0.00011 | afc | ENSP00000354499,ENSP00000354554,ENSP00000354632,ENSP00000354961,ENSP00000354982 | MT-ATP6,MT-CO1,MT-CO3,MT-CYB,MT-ND4 |
| Cluster11 | CL:22328 | respirasome | 19 | 3.55411 | top of list | 0.00043 | afc | ENSP00000184266,ENSP00000317780,ENSP00000332887,ENSP00000354499,ENSP00000354554,ENSP00000354632,ENSP00000354687,ENSP00000354728,ENSP00000354813,ENSP00000354876,ENSP00000354961,ENSP00000354982,ENSP00000355046,ENSP00000355206,ENSP00000367939,ENSP00000417656,ENSP00000418438,ENSP00000418842,ENSP00000419087 | COX5A,COX7B,MT-ATP6,MT-CO1,MT-CO2,MT-CO3,MT-CYB,MT-ND1,MT-ND2,MT-ND3,MT-ND4,MT-ND4L,MT-ND5,NDUFA3,NDUFA6,NDUFB2,NDUFB4,UQCR10,UQCRQ |
| Cluster11 | CL:7134 | G alpha (i) signalling events | 3 | 8.0257 | top of list | 0.0042 | afc | ENSP00000226524,ENSP00000296028,ENSP00000474412 | CCL5,PF4V1,PPBP |
| Cluster11 | CL:22446 | cytochrome complex, and Cytochrome c oxidase subunit VII | 10 | 4.16435 | top of list | 0.0042 | afc | ENSP00000317780,ENSP00000332887,ENSP00000354499,ENSP00000354554,ENSP00000354632,ENSP00000354876,ENSP00000354961,ENSP00000354982,ENSP00000367939,ENSP00000417656 | COX5A,COX7B,MT-ATP6,MT-CO1,MT-CO2,MT-CO3,MT-CYB,MT-ND4,UQCR10,UQCRQ |
| Cluster11 | CL:9922 | Formation of Fibrin Clot (Clotting Cascade), and platelet alpha granule lumen | 4 | 6.74301 | top of list | 0.0051 | afc | ENSP00000260356,ENSP00000264870,ENSP00000370010,ENSP00000377783 | F13A1,PROS1,THBS1,TMSB4X |
| Cluster11 | CL:22408 | Leber hereditary optic neuropathy | 4 | 6.54435 | top of list | 0.0061 | afc | ENSP00000354687,ENSP00000354728,ENSP00000354813,ENSP00000355046 | MT-ND1,MT-ND2,MT-ND4L,MT-ND5 |
| Cluster11 | CL:1331 | Muscle protein, and striated muscle thin filament | 4 | 6.2124 | top of list | 0.0099 | afc | ENSP00000279022,ENSP00000345230,ENSP00000351022,ENSP00000446955 | MYL6,MYL9,TPM1,TPM4 |
| Cluster | #term ID | term description | genes mapped | enrichment score | direction | false discovery rate | method | matching proteins in your input (IDs) | matching proteins in your input (labels) |
| Cluster12 | CL:14966 | GTP hydrolysis and joining of the 60S ribosomal subunit, and Protein export | 20 | 6.90626 | top of list | 5.00E-07 | afc | ENSP00000272317,ENSP00000307940,ENSP00000311430,ENSP00000313007,ENSP00000339063,ENSP00000346001,ENSP00000346027,ENSP00000348849,ENSP00000359345,ENSP00000362744,ENSP00000369757,ENSP00000376056,ENSP00000388806,ENSP00000403172,ENSP00000413436,ENSP00000419449,ENSP00000449328,ENSP00000452909,ENSP00000463784,ENSP00000477781 | EEF1A1,EEF1B2,EEF2,EIF1,EIF4B,PABPC1,RPL10,RPL21,RPL26,RPL28,RPL3,RPL4,RPL5,RPL6,RPLP0,RPS26,RPS27A,RPS4X,RPS6,TPT1 |
| Cluster12 | CL:14967 | GTP hydrolysis and joining of the 60S ribosomal subunit, and Protein export | 18 | 6.78078 | top of list | 2.00E-06 | afc | ENSP00000272317,ENSP00000307940,ENSP00000311430,ENSP00000313007,ENSP00000339063,ENSP00000346001,ENSP00000346027,ENSP00000348849,ENSP00000359345,ENSP00000362744,ENSP00000369757,ENSP00000388806,ENSP00000403172,ENSP00000413436,ENSP00000419449,ENSP00000449328,ENSP00000452909,ENSP00000463784 | EEF1A1,EEF2,EIF1,EIF4B,PABPC1,RPL10,RPL21,RPL26,RPL28,RPL3,RPL4,RPL5,RPL6,RPLP0,RPS26,RPS27A,RPS4X,RPS6 |
| Cluster12 | CL:14978 | Peptide chain elongation | 15 | 6.6209 | top of list | 3.50E-05 | afc | ENSP00000272317,ENSP00000307940,ENSP00000311430,ENSP00000339063,ENSP00000346001,ENSP00000346027,ENSP00000348849,ENSP00000359345,ENSP00000362744,ENSP00000369757,ENSP00000403172,ENSP00000413436,ENSP00000449328,ENSP00000452909,ENSP00000463784 | EEF1A1,EEF2,RPL10,RPL21,RPL26,RPL28,RPL3,RPL4,RPL5,RPL6,RPLP0,RPS26,RPS27A,RPS4X,RPS6 |
| Cluster12 | CL:14982 | Peptide chain elongation | 13 | 6.46104 | top of list | 0.0002 | afc | ENSP00000272317,ENSP00000307940,ENSP00000346001,ENSP00000346027,ENSP00000348849,ENSP00000359345,ENSP00000362744,ENSP00000369757,ENSP00000403172,ENSP00000413436,ENSP00000449328,ENSP00000452909,ENSP00000463784 | EEF2,RPL10,RPL21,RPL26,RPL28,RPL3,RPL5,RPL6,RPLP0,RPS26,RPS27A,RPS4X,RPS6 |
| Cluster12 | CL:4662 | Interferon alpha/beta signaling, and Interferon-induced protein 44 family | 20 | 2.50418 | bottom of list | 0.00041 | afc | ENSP00000252593,ENSP00000257570,ENSP00000306565,ENSP00000318982,ENSP00000333657,ENSP00000342513,ENSP00000345494,ENSP00000354822,ENSP00000359783,ENSP00000359787,ENSP00000360883,ENSP00000368699,ENSP00000380697,ENSP00000381601,ENSP00000382707,ENSP00000386187,ENSP00000388001,ENSP00000395590,ENSP00000483430,ENSP00000484689 | BST2,EPSTI1,IFI27,IFI35,IFI44,IFI44L,IFI6,IFIT3,IFITM1,IFITM2,IFITM3,IRF7,ISG15,ISG20,MX1,MX2,OAS1,OASL,PLSCR1,XAF1 |
| Cluster12 | CL:4665 | Interferon alpha/beta signaling | 18 | 2.65274 | bottom of list | 0.00041 | afc | ENSP00000252593,ENSP00000257570,ENSP00000306565,ENSP00000333657,ENSP00000342513,ENSP00000354822,ENSP00000359783,ENSP00000359787,ENSP00000360883,ENSP00000368699,ENSP00000380697,ENSP00000381601,ENSP00000382707,ENSP00000386187,ENSP00000388001,ENSP00000395590,ENSP00000483430,ENSP00000484689 | BST2,IFI27,IFI35,IFI44,IFI44L,IFI6,IFIT3,IFITM1,IFITM2,IFITM3,IRF7,ISG15,ISG20,MX1,MX2,OAS1,OASL,XAF1 |
| **Cluster** | **#term ID** | **term description** | **genes mapped** | **enrichment score** | **direction** | **false discovery rate** | **method** | **matching proteins in your input (IDs)** | **matching proteins in your input (labels)** |
| Cluster12 | CL:14983 | Peptide chain elongation | 9 | 7.10355 | top of list | 0.00043 | afc | ENSP00000272317,ENSP00000307940,ENSP00000346001,ENSP00000346027,ENSP00000348849,ENSP00000362744,ENSP00000369757,ENSP00000403172,ENSP00000463784 | EEF2,RPL21,RPL26,RPL3,RPL6,RPS26,RPS27A,RPS4X,RPS6 |
| Cluster12 | CL:22328 | respirasome | 8 | 7.3363 | top of list | 0.00049 | afc | ENSP00000354554,ENSP00000354632,ENSP00000354728,ENSP00000354813,ENSP00000354961,ENSP00000354982,ENSP00000355046,ENSP00000355206 | MT-ATP6,MT-CO3,MT-CYB,MT-ND2,MT-ND3,MT-ND4,MT-ND4L,MT-ND5 |
| Cluster12 | CL:4670 | type I interferon signaling pathway | 14 | 2.82588 | bottom of list | 0.00075 | afc | ENSP00000257570,ENSP00000306565,ENSP00000333657,ENSP00000342513,ENSP00000354822,ENSP00000360883,ENSP00000380697,ENSP00000381601,ENSP00000382707,ENSP00000386187,ENSP00000388001,ENSP00000395590,ENSP00000483430,ENSP00000484689 | IFI27,IFI35,IFI6,IFIT3,IFITM1,IFITM2,IFITM3,IRF7,ISG20,MX1,MX2,OAS1,OASL,XAF1 |
| Cluster12 | CL:14985 | Viral mRNA Translation | 8 | 7.11003 | top of list | 0.00075 | afc | ENSP00000272317,ENSP00000346001,ENSP00000346027,ENSP00000348849,ENSP00000362744,ENSP00000369757,ENSP00000403172,ENSP00000463784 | RPL21,RPL26,RPL3,RPL6,RPS26,RPS27A,RPS4X,RPS6 |
| Cluster12 | CL:4668 | type I interferon signaling pathway | 15 | 2.73265 | bottom of list | 0.00082 | afc | ENSP00000252593,ENSP00000257570,ENSP00000306565,ENSP00000333657,ENSP00000342513,ENSP00000354822,ENSP00000360883,ENSP00000380697,ENSP00000381601,ENSP00000382707,ENSP00000386187,ENSP00000388001,ENSP00000395590,ENSP00000483430,ENSP00000484689 | BST2,IFI27,IFI35,IFI6,IFIT3,IFITM1,IFITM2,IFITM3,IRF7,ISG20,MX1,MX2,OAS1,OASL,XAF1 |
| Cluster | #term ID | term description | genes mapped | enrichment score | direction | false discovery rate | method | matching proteins in your input (IDs) | matching proteins in your input (labels) |
| Cluster13 | CL:22328 | respirasome | 11 | 6.54242 | top of list | 5.28E-06 | afc | ENSP00000354499,ENSP00000354554,ENSP00000354632,ENSP00000354728,ENSP00000354813,ENSP00000354876,ENSP00000354961,ENSP00000354982,ENSP00000355046,ENSP00000355206,ENSP00000418438 | MT-ATP6,MT-CO1,MT-CO2,MT-CO3,MT-CYB,MT-ND2,MT-ND3,MT-ND4,MT-ND4L,MT-ND5,NDUFA3 |
| Cluster13 | CL:22327 | Oxidative phosphorylation | 15 | 5.05935 | top of list | 1.50E-05 | afc | ENSP00000243997,ENSP00000292475,ENSP00000354499,ENSP00000354554,ENSP00000354632,ENSP00000354728,ENSP00000354813,ENSP00000354876,ENSP00000354961,ENSP00000354982,ENSP00000355046,ENSP00000355206,ENSP00000355265,ENSP00000418438,ENSP00000429690 | ATP5E,ATP5J2,ATP6V0E1,MT-ATP6,MT-ATP8,MT-CO1,MT-CO2,MT-CO3,MT-CYB,MT-ND2,MT-ND3,MT-ND4,MT-ND4L,MT-ND5,NDUFA3 |
| Cluster13 | CL:4662 | Interferon alpha/beta signaling, and Interferon-induced protein 44 family | 19 | 4.4406 | bottom of list | 1.50E-05 | afc | ENSP00000252593,ENSP00000306565,ENSP00000318982,ENSP00000326247,ENSP00000342513,ENSP00000345494,ENSP00000354822,ENSP00000359787,ENSP00000360869,ENSP00000360883,ENSP00000368699,ENSP00000380697,ENSP00000381601,ENSP00000382707,ENSP00000386187,ENSP00000388001,ENSP00000395590,ENSP00000483430,ENSP00000484689 | BST2,EPSTI1,IFI27,IFI35,IFI44L,IFI6,IFIT1,IFIT3,IFITM1,IFITM2,IFITM3,IRF7,ISG15,ISG20,MX1,OAS1,PLSCR1,SAMD9L,XAF1 |
| Cluster13 | CL:4665 | Interferon alpha/beta signaling | 17 | 4.69124 | bottom of list | 1.50E-05 | afc | ENSP00000252593,ENSP00000306565,ENSP00000326247,ENSP00000342513,ENSP00000354822,ENSP00000359787,ENSP00000360869,ENSP00000360883,ENSP00000368699,ENSP00000380697,ENSP00000381601,ENSP00000382707,ENSP00000386187,ENSP00000388001,ENSP00000395590,ENSP00000483430,ENSP00000484689 | BST2,IFI27,IFI35,IFI44L,IFI6,IFIT1,IFIT3,IFITM1,IFITM2,IFITM3,IRF7,ISG15,ISG20,MX1,OAS1,SAMD9L,XAF1 |
| Cluster13 | CL:22446 | cytochrome complex, and Cytochrome c oxidase subunit VII | 6 | 7.43513 | top of list | 8.98E-05 | afc | ENSP00000354499,ENSP00000354554,ENSP00000354632,ENSP00000354876,ENSP00000354961,ENSP00000354982 | MT-ATP6,MT-CO1,MT-CO2,MT-CO3,MT-CYB,MT-ND4 |
| Cluster13 | CL:22502 | Leber hereditary optic neuropathy | 5 | 7.89819 | top of list | 0.00011 | afc | ENSP00000354499,ENSP00000354554,ENSP00000354632,ENSP00000354961,ENSP00000354982 | MT-ATP6,MT-CO1,MT-CO3,MT-CYB,MT-ND4 |
| Cluster13 | CL:4668 | type I interferon signaling pathway | 14 | 4.3814 | bottom of list | 0.00018 | afc | ENSP00000252593,ENSP00000306565,ENSP00000342513,ENSP00000354822,ENSP00000360869,ENSP00000360883,ENSP00000380697,ENSP00000381601,ENSP00000382707,ENSP00000386187,ENSP00000388001,ENSP00000395590,ENSP00000483430,ENSP00000484689 | BST2,IFI27,IFI35,IFI6,IFIT1,IFIT3,IFITM1,IFITM2,IFITM3,IRF7,ISG20,MX1,OAS1,XAF1 |
| Cluster13 | CL:4670 | type I interferon signaling pathway | 13 | 4.3947 | bottom of list | 0.0003 | afc | ENSP00000306565,ENSP00000342513,ENSP00000354822,ENSP00000360869,ENSP00000360883,ENSP00000380697,ENSP00000381601,ENSP00000382707,ENSP00000386187,ENSP00000388001,ENSP00000395590,ENSP00000483430,ENSP00000484689 | IFI27,IFI35,IFI6,IFIT1,IFIT3,IFITM1,IFITM2,IFITM3,IRF7,ISG20,MX1,OAS1,XAF1 |
| Cluster13 | CL:4661 | Interferon alpha/beta signaling, and Interferon gamma signaling | 21 | 4.35681 | bottom of list | 0.0019 | ks | ENSP00000245185,ENSP00000252593,ENSP00000306565,ENSP00000318982,ENSP00000326247,ENSP00000342513,ENSP00000345494,ENSP00000354822,ENSP00000359787,ENSP00000360869,ENSP00000360883,ENSP00000368699,ENSP00000369299,ENSP00000380697,ENSP00000381601,ENSP00000382707,ENSP00000386187,ENSP00000388001,ENSP00000395590,ENSP00000483430,ENSP00000484689 | BST2,EPSTI1,IFI27,IFI35,IFI44L,IFI6,IFIT1,IFIT3,IFITM1,IFITM2,IFITM3,IRF7,ISG15,ISG20,MT2A,MX1,OAS1,PLSCR1,SAMD9L,TRIM22,XAF1 |
| Cluster13 | CL:4659 | Interferon alpha/beta signaling, and Interferon gamma signaling | 22 | 4.2023 | bottom of list | 0.0036 | ks | ENSP00000245185,ENSP00000252593,ENSP00000258381,ENSP00000306565,ENSP00000318982,ENSP00000326247,ENSP00000342513,ENSP00000345494,ENSP00000354822,ENSP00000359787,ENSP00000360869,ENSP00000360883,ENSP00000368699,ENSP00000369299,ENSP00000380697,ENSP00000381601,ENSP00000382707,ENSP00000386187,ENSP00000388001,ENSP00000395590,ENSP00000483430,ENSP00000484689 | BST2,EPSTI1,IFI27,IFI35,IFI44L,IFI6,IFIT1,IFIT3,IFITM1,IFITM2,IFITM3,IRF7,ISG15,ISG20,MT2A,MX1,OAS1,PLSCR1,SAMD9L,SP110,TRIM22,XAF1 |
| Cluster13 | CL:4673 | 2'-5'-oligoadenylate synthase, and type I interferon signaling pathway | 8 | 4.43153 | bottom of list | 0.0077 | afc | ENSP00000342513,ENSP00000354822,ENSP00000360869,ENSP00000360883,ENSP00000380697,ENSP00000381601,ENSP00000388001,ENSP00000395590 | IFI35,IFI6,IFIT1,IFIT3,IRF7,MX1,OAS1,XAF1 |
| **Cluster** | **#term ID** | **term description** | **genes mapped** | **enrichment score** | **direction** | **false discovery rate** | **method** | **matching proteins in your input (IDs)** | **matching proteins in your input (labels)** |
| Cluster13 | CL:22332 | mitochondrial respiratory chain complex I | 5 | 5.47117 | top of list | 0.0077 | afc | ENSP00000354728,ENSP00000354813,ENSP00000355046,ENSP00000355206,ENSP00000418438 | MT-ND2,MT-ND3,MT-ND4L,MT-ND5,NDUFA3 |
| Cluster13 | CL:22408 | Leber hereditary optic neuropathy | 3 | 7.14898 | top of list | 0.0086 | afc | ENSP00000354728,ENSP00000354813,ENSP00000355046 | MT-ND2,MT-ND4L,MT-ND5 |
| Cluster | #term ID | term description | genes mapped | enrichment score | direction | false discovery rate | method | matching proteins in your input (IDs) | matching proteins in your input (labels) |
| Cluster14 | CL:22328 | respirasome | 9 | 6.66585 | top of list | 1.87E-05 | afc | ENSP00000354499,ENSP00000354554,ENSP00000354632,ENSP00000354728,ENSP00000354813,ENSP00000354961,ENSP00000354982,ENSP00000355046,ENSP00000355206 | MT-ATP6,MT-CO1,MT-CO3,MT-CYB,MT-ND2,MT-ND3,MT-ND4,MT-ND4L,MT-ND5 |
| Cluster14 | CL:14978 | Peptide chain elongation | 6 | 6.3707 | top of list | 0.00061 | afc | ENSP00000272317,ENSP00000311430,ENSP00000341885,ENSP00000346001,ENSP00000348849,ENSP00000362744 | RPL3,RPL4,RPS2,RPS26,RPS27A,RPS4X |
| Cluster14 | CL:4661 | Interferon alpha/beta signaling, and Interferon gamma signaling | 20 | 4.13895 | bottom of list | 0.00061 | afc | ENSP00000245185,ENSP00000252593,ENSP00000306565,ENSP00000318982,ENSP00000333657,ENSP00000342513,ENSP00000345494,ENSP00000354822,ENSP00000359783,ENSP00000359787,ENSP00000360883,ENSP00000368699,ENSP00000369299,ENSP00000380697,ENSP00000381601,ENSP00000382707,ENSP00000386187,ENSP00000388001,ENSP00000483430,ENSP00000484689 | BST2,EPSTI1,IFI27,IFI44,IFI44L,IFI6,IFIT3,IFITM1,IFITM2,IFITM3,IRF7,ISG15,ISG20,MT2A,MX1,MX2,OAS1,PLSCR1,TRIM22,XAF1 |
| Cluster14 | CL:4662 | Interferon alpha/beta signaling, and Interferon-induced protein 44 family | 18 | 4.32632 | bottom of list | 0.00061 | afc | ENSP00000252593,ENSP00000306565,ENSP00000318982,ENSP00000333657,ENSP00000342513,ENSP00000345494,ENSP00000354822,ENSP00000359783,ENSP00000359787,ENSP00000360883,ENSP00000368699,ENSP00000380697,ENSP00000381601,ENSP00000382707,ENSP00000386187,ENSP00000388001,ENSP00000483430,ENSP00000484689 | BST2,EPSTI1,IFI27,IFI44,IFI44L,IFI6,IFIT3,IFITM1,IFITM2,IFITM3,IRF7,ISG15,ISG20,MX1,MX2,OAS1,PLSCR1,XAF1 |
| Cluster14 | CL:4665 | Interferon alpha/beta signaling | 16 | 4.70509 | bottom of list | 0.00061 | afc | ENSP00000252593,ENSP00000306565,ENSP00000333657,ENSP00000342513,ENSP00000354822,ENSP00000359783,ENSP00000359787,ENSP00000360883,ENSP00000368699,ENSP00000380697,ENSP00000381601,ENSP00000382707,ENSP00000386187,ENSP00000388001,ENSP00000483430,ENSP00000484689 | BST2,IFI27,IFI44,IFI44L,IFI6,IFIT3,IFITM1,IFITM2,IFITM3,IRF7,ISG15,ISG20,MX1,MX2,OAS1,XAF1 |
| Cluster14 | CL:14985 | Viral mRNA Translation | 5 | 6.54174 | top of list | 0.001 | afc | ENSP00000272317,ENSP00000341885,ENSP00000346001,ENSP00000348849,ENSP00000362744 | RPL3,RPS2,RPS26,RPS27A,RPS4X |
| Cluster14 | CL:22502 | Leber hereditary optic neuropathy | 5 | 6.62655 | top of list | 0.001 | afc | ENSP00000354499,ENSP00000354554,ENSP00000354632,ENSP00000354961,ENSP00000354982 | MT-ATP6,MT-CO1,MT-CO3,MT-CYB,MT-ND4 |
| Cluster14 | CL:22332 | mitochondrial respiratory chain complex I | 4 | 6.71496 | top of list | 0.0021 | afc | ENSP00000354728,ENSP00000354813,ENSP00000355046,ENSP00000355206 | MT-ND2,MT-ND3,MT-ND4L,MT-ND5 |
| Cluster14 | CL:4670 | type I interferon signaling pathway | 12 | 4.47548 | bottom of list | 0.0028 | afc | ENSP00000306565,ENSP00000333657,ENSP00000342513,ENSP00000354822,ENSP00000360883,ENSP00000380697,ENSP00000381601,ENSP00000382707,ENSP00000386187,ENSP00000388001,ENSP00000483430,ENSP00000484689 | IFI27,IFI6,IFIT3,IFITM1,IFITM2,IFITM3,IRF7,ISG20,MX1,MX2,OAS1,XAF1 |
| Cluster14 | CL:4668 | type I interferon signaling pathway | 13 | 4.27261 | bottom of list | 0.0032 | afc | ENSP00000252593,ENSP00000306565,ENSP00000333657,ENSP00000342513,ENSP00000354822,ENSP00000360883,ENSP00000380697,ENSP00000381601,ENSP00000382707,ENSP00000386187,ENSP00000388001,ENSP00000483430,ENSP00000484689 | BST2,IFI27,IFI6,IFIT3,IFITM1,IFITM2,IFITM3,IRF7,ISG20,MX1,MX2,OAS1,XAF1 |
| Cluster | #term ID | term description | genes mapped | enrichment score | direction | false discovery rate | method | matching proteins in your input (IDs) | matching proteins in your input (labels) |
| Cluster15 | CL:22328 | respirasome | 8 | 6.89044 | top of list | 0.00029 | afc | ENSP00000354554,ENSP00000354632,ENSP00000354728,ENSP00000354813,ENSP00000354961,ENSP00000355046,ENSP00000355206,ENSP00000362060 | MT-ATP6,MT-CYB,MT-ND2,MT-ND3,MT-ND4,MT-ND4L,MT-ND5,NDUFS5 |
| Cluster15 | CL:22327 | Oxidative phosphorylation | 9 | 6.17782 | top of list | 0.00036 | afc | ENSP00000301587,ENSP00000354554,ENSP00000354632,ENSP00000354728,ENSP00000354813,ENSP00000354961,ENSP00000355046,ENSP00000355206,ENSP00000362060 | ATP5H,MT-ATP6,MT-CYB,MT-ND2,MT-ND3,MT-ND4,MT-ND4L,MT-ND5,NDUFS5 |
| Cluster15 | CL:4665 | Interferon alpha/beta signaling | 17 | 3.77206 | bottom of list | 0.0013 | afc | ENSP00000245414,ENSP00000252593,ENSP00000306565,ENSP00000333657,ENSP00000342513,ENSP00000354822,ENSP00000357459,ENSP00000359783,ENSP00000359787,ENSP00000360883,ENSP00000368699,ENSP00000380697,ENSP00000381601,ENSP00000382707,ENSP00000386187,ENSP00000388001,ENSP00000483430 | ADAR,BST2,IFI27,IFI44,IFI44L,IFI6,IFIT3,IFITM1,IFITM3,IRF1,IRF7,ISG15,ISG20,MX1,MX2,OAS1,XAF1 |
| Cluster15 | CL:14967 | GTP hydrolysis and joining of the 60S ribosomal subunit, and Protein export | 5 | 7.13116 | top of list | 0.0013 | afc | ENSP00000272317,ENSP00000346001,ENSP00000348849,ENSP00000388806,ENSP00000485663 | EIF3L,EIF4B,RPL3,RPS26,RPS27A |
| Cluster15 | CL:4662 | Interferon alpha/beta signaling, and Interferon-induced protein 44 family | 18 | 3.75925 | bottom of list | 0.0013 | afc | ENSP00000245414,ENSP00000252593,ENSP00000306565,ENSP00000318982,ENSP00000333657,ENSP00000342513,ENSP00000354822,ENSP00000357459,ENSP00000359783,ENSP00000359787,ENSP00000360883,ENSP00000368699,ENSP00000380697,ENSP00000381601,ENSP00000382707,ENSP00000386187,ENSP00000388001,ENSP00000483430 | ADAR,BST2,EPSTI1,IFI27,IFI44,IFI44L,IFI6,IFIT3,IFITM1,IFITM3,IRF1,IRF7,ISG15,ISG20,MX1,MX2,OAS1,XAF1 |
| **Cluster** | **#term ID** | **term description** | **genes mapped** | **enrichment score** | **direction** | **false discovery rate** | **method** | **matching proteins in your input (IDs)** | **matching proteins in your input (labels)** |
| Cluster15 | CL:4661 | Interferon alpha/beta signaling, and Interferon gamma signaling | 20 | 3.63074 | bottom of list | 0.0013 | afc | ENSP00000245185,ENSP00000245414,ENSP00000252593,ENSP00000306565,ENSP00000318982,ENSP00000333657,ENSP00000342513,ENSP00000354822,ENSP00000357459,ENSP00000359783,ENSP00000359787,ENSP00000360883,ENSP00000368699,ENSP00000369299,ENSP00000380697,ENSP00000381601,ENSP00000382707,ENSP00000386187,ENSP00000388001,ENSP00000483430 | ADAR,BST2,EPSTI1,IFI27,IFI44,IFI44L,IFI6,IFIT3,IFITM1,IFITM3,IRF1,IRF7,ISG15,ISG20,MT2A,MX1,MX2,OAS1,TRIM22,XAF1 |
| Cluster15 | CL:14963 | GTP hydrolysis and joining of the 60S ribosomal subunit, and Nonsense-mediated mRNA decay | 6 | 5.84607 | top of list | 0.0045 | afc | ENSP00000272317,ENSP00000336702,ENSP00000346001,ENSP00000348849,ENSP00000388806,ENSP00000485663 | EIF3L,EIF4B,EIF5A,RPL3,RPS26,RPS27A |
| Cluster15 | CL:4670 | type I interferon signaling pathway | 11 | 4.00869 | bottom of list | 0.0053 | afc | ENSP00000306565,ENSP00000333657,ENSP00000342513,ENSP00000354822,ENSP00000360883,ENSP00000380697,ENSP00000381601,ENSP00000382707,ENSP00000386187,ENSP00000388001,ENSP00000483430 | IFI27,IFI6,IFIT3,IFITM1,IFITM3,IRF7,ISG20,MX1,MX2,OAS1,XAF1 |
| Cluster15 | CL:22332 | mitochondrial respiratory chain complex I | 5 | 6.2078 | top of list | 0.0059 | afc | ENSP00000354728,ENSP00000354813,ENSP00000355046,ENSP00000355206,ENSP00000362060 | MT-ND2,MT-ND3,MT-ND4L,MT-ND5,NDUFS5 |
| Cluster15 | CL:22502 | Leber hereditary optic neuropathy | 3 | 8.02815 | top of list | 0.0061 | afc | ENSP00000354554,ENSP00000354632,ENSP00000354961 | MT-ATP6,MT-CYB,MT-ND4 |
| Cluster | #term ID | term description | genes mapped | enrichment score | direction | false discovery rate | method | matching proteins in your input (IDs) | matching proteins in your input (labels) |
| Cluster16 | CL:14980 | Peptide chain elongation | 27 | 6.01124 | top of list | 3.53E-09 | ks | ENSP00000225430,ENSP00000230050,ENSP00000272317,ENSP00000296674,ENSP00000307889,ENSP00000318646,ENSP00000322419,ENSP00000331019,ENSP00000339095,ENSP00000341885,ENSP00000346015,ENSP00000346027,ENSP00000346050,ENSP00000346088,ENSP00000348849,ENSP00000362744,ENSP00000363018,ENSP00000363676,ENSP00000369757,ENSP00000375730,ENSP00000379888,ENSP00000385958,ENSP00000403172,ENSP00000413436,ENSP00000435777,ENSP00000447001,ENSP00000472469 | RPL10,RPL10A,RPL11,RPL13,RPL13A,RPL18,RPL19,RPL21,RPL22,RPL27A,RPL6,RPLP2,RPS12,RPS13,RPS14,RPS15A,RPS2,RPS23,RPS26,RPS27A,RPS27L,RPS28,RPS3A,RPS4X,RPS6,RPS7,RPS8 |
| Cluster16 | CL:14976 | Peptide chain elongation | 30 | 5.80695 | top of list | 3.53E-09 | ks | ENSP00000225430,ENSP00000230050,ENSP00000272317,ENSP00000296674,ENSP00000307889,ENSP00000318646,ENSP00000322419,ENSP00000331019,ENSP00000339095,ENSP00000341885,ENSP00000346015,ENSP00000346027,ENSP00000346050,ENSP00000346088,ENSP00000348849,ENSP00000362744,ENSP00000363018,ENSP00000363676,ENSP00000369757,ENSP00000375730,ENSP00000378163,ENSP00000379888,ENSP00000385958,ENSP00000388337,ENSP00000403172,ENSP00000413436,ENSP00000428085,ENSP00000435777,ENSP00000447001,ENSP00000472469 | RPL10,RPL10A,RPL11,RPL13,RPL13A,RPL18,RPL19,RPL21,RPL22,RPL27A,RPL30,RPL34,RPL6,RPLP2,RPS12,RPS13,RPS14,RPS15A,RPS2,RPS23,RPS26,RPS27A,RPS27L,RPS28,RPS3A,RPS4X,RPS6,RPS7,RPS8,SEC61G |
| Cluster16 | CL:14982 | Peptide chain elongation | 24 | 6.32409 | top of list | 3.53E-09 | ks | ENSP00000225430,ENSP00000230050,ENSP00000272317,ENSP00000296674,ENSP00000307889,ENSP00000318646,ENSP00000322419,ENSP00000339095,ENSP00000341885,ENSP00000346027,ENSP00000346050,ENSP00000346088,ENSP00000348849,ENSP00000362744,ENSP00000363018,ENSP00000363676,ENSP00000369757,ENSP00000375730,ENSP00000379888,ENSP00000385958,ENSP00000403172,ENSP00000413436,ENSP00000435777,ENSP00000472469 | RPL10,RPL10A,RPL11,RPL13,RPL13A,RPL19,RPL21,RPL22,RPL6,RPLP2,RPS12,RPS13,RPS14,RPS15A,RPS2,RPS23,RPS26,RPS27A,RPS28,RPS3A,RPS4X,RPS6,RPS7,RPS8 |
| Cluster16 | CL:14967 | GTP hydrolysis and joining of the 60S ribosomal subunit, and Protein export | 33 | 5.60627 | top of list | 3.53E-09 | ks | ENSP00000225430,ENSP00000230050,ENSP00000272317,ENSP00000278572,ENSP00000296674,ENSP00000307889,ENSP00000318646,ENSP00000322419,ENSP00000331019,ENSP00000339095,ENSP00000341885,ENSP00000346015,ENSP00000346027,ENSP00000346050,ENSP00000346088,ENSP00000348849,ENSP00000362744,ENSP00000363018,ENSP00000363676,ENSP00000364119,ENSP00000369757,ENSP00000375730,ENSP00000378163,ENSP00000379888,ENSP00000385958,ENSP00000388337,ENSP00000388806,ENSP00000403172,ENSP00000413436,ENSP00000428085,ENSP00000435777,ENSP00000447001,ENSP00000472469 | EIF2S2,EIF4B,RPL10,RPL10A,RPL11,RPL13,RPL13A,RPL18,RPL19,RPL21,RPL22,RPL27A,RPL30,RPL34,RPL6,RPLP2,RPS12,RPS13,RPS14,RPS15A,RPS2,RPS23,RPS26,RPS27A,RPS27L,RPS28,RPS3,RPS3A,RPS4X,RPS6,RPS7,RPS8,SEC61G |
| Cluster16 | CL:14978 | Peptide chain elongation | 28 | 6.05574 | top of list | 3.53E-09 | ks | ENSP00000225430,ENSP00000230050,ENSP00000272317,ENSP00000296674,ENSP00000307889,ENSP00000318646,ENSP00000322419,ENSP00000331019,ENSP00000339095,ENSP00000341885,ENSP00000346015,ENSP00000346027,ENSP00000346050,ENSP00000346088,ENSP00000348849,ENSP00000362744,ENSP00000363018,ENSP00000363676,ENSP00000369757,ENSP00000375730,ENSP00000378163,ENSP00000379888,ENSP00000385958,ENSP00000403172,ENSP00000413436,ENSP00000435777,ENSP00000447001,ENSP00000472469 | RPL10,RPL10A,RPL11,RPL13,RPL13A,RPL18,RPL19,RPL21,RPL22,RPL27A,RPL34,RPL6,RPLP2,RPS12,RPS13,RPS14,RPS15A,RPS2,RPS23,RPS26,RPS27A,RPS27L,RPS28,RPS3A,RPS4X,RPS6,RPS7,RPS8 |
| Cluster16 | CL:14983 | Peptide chain elongation | 23 | 6.35665 | top of list | 5.25E-09 | ks | ENSP00000225430,ENSP00000230050,ENSP00000272317,ENSP00000296674,ENSP00000307889,ENSP00000318646,ENSP00000322419,ENSP00000339095,ENSP00000341885,ENSP00000346027,ENSP00000346050,ENSP00000346088,ENSP00000348849,ENSP00000362744,ENSP00000363018,ENSP00000363676,ENSP00000369757,ENSP00000375730,ENSP00000379888,ENSP00000385958,ENSP00000403172,ENSP00000435777,ENSP00000472469 | RPL10A,RPL11,RPL13,RPL13A,RPL19,RPL21,RPL22,RPL6,RPLP2,RPS12,RPS13,RPS14,RPS15A,RPS2,RPS23,RPS26,RPS27A,RPS28,RPS3A,RPS4X,RPS6,RPS7,RPS8 |
| Cluster16 | CL:14985 | Viral mRNA Translation | 22 | 6.38777 | top of list | 1.09E-08 | ks | ENSP00000225430,ENSP00000230050,ENSP00000272317,ENSP00000296674,ENSP00000307889,ENSP00000318646,ENSP00000339095,ENSP00000341885,ENSP00000346027,ENSP00000346050,ENSP00000346088,ENSP00000348849,ENSP00000362744,ENSP00000363018,ENSP00000363676,ENSP00000369757,ENSP00000375730,ENSP00000379888,ENSP00000385958,ENSP00000403172,ENSP00000435777,ENSP00000472469 | RPL10A,RPL11,RPL13,RPL13A,RPL19,RPL21,RPL22,RPL6,RPS12,RPS13,RPS14,RPS15A,RPS2,RPS23,RPS26,RPS27A,RPS28,RPS3A,RPS4X,RPS6,RPS7,RPS8 |
| Cluster16 | CL:4661 | Interferon alpha/beta signaling, and Interferon gamma signaling | 18 | 3.12053 | bottom of list | 3.87E-05 | afc | ENSP00000245185,ENSP00000252593,ENSP00000306565,ENSP00000318982,ENSP00000342513,ENSP00000345494,ENSP00000354822,ENSP00000359787,ENSP00000360869,ENSP00000360883,ENSP00000368699,ENSP00000369299,ENSP00000380697,ENSP00000381601,ENSP00000382707,ENSP00000386187,ENSP00000388001,ENSP00000483430 | BST2,EPSTI1,IFI27,IFI44L,IFI6,IFIT1,IFIT3,IFITM1,IFITM3,IRF7,ISG15,ISG20,MT2A,MX1,OAS1,PLSCR1,TRIM22,XAF1 |
| **Cluster** | **#term ID** | **term description** | **genes mapped** | **enrichment score** | **direction** | **false discovery rate** | **method** | **matching proteins in your input (IDs)** | **matching proteins in your input (labels)** |
| Cluster16 | CL:4659 | Interferon alpha/beta signaling, and Interferon gamma signaling | 19 | 3.00524 | bottom of list | 4.13E-05 | afc | ENSP00000245185,ENSP00000252593,ENSP00000258381,ENSP00000306565,ENSP00000318982,ENSP00000342513,ENSP00000345494,ENSP00000354822,ENSP00000359787,ENSP00000360869,ENSP00000360883,ENSP00000368699,ENSP00000369299,ENSP00000380697,ENSP00000381601,ENSP00000382707,ENSP00000386187,ENSP00000388001,ENSP00000483430 | BST2,EPSTI1,IFI27,IFI44L,IFI6,IFIT1,IFIT3,IFITM1,IFITM3,IRF7,ISG15,ISG20,MT2A,MX1,OAS1,PLSCR1,SP110,TRIM22,XAF1 |
| Cluster16 | CL:4662 | Interferon alpha/beta signaling, and Interferon-induced protein 44 family | 16 | 3.20108 | bottom of list | 5.58E-05 | afc | ENSP00000252593,ENSP00000306565,ENSP00000318982,ENSP00000342513,ENSP00000345494,ENSP00000354822,ENSP00000359787,ENSP00000360869,ENSP00000360883,ENSP00000368699,ENSP00000380697,ENSP00000381601,ENSP00000382707,ENSP00000386187,ENSP00000388001,ENSP00000483430 | BST2,EPSTI1,IFI27,IFI44L,IFI6,IFIT1,IFIT3,IFITM1,IFITM3,IRF7,ISG15,ISG20,MX1,OAS1,PLSCR1,XAF1 |
| Cluster16 | CL:4665 | Interferon alpha/beta signaling | 14 | 3.41236 | bottom of list | 5.64E-05 | afc | ENSP00000252593,ENSP00000306565,ENSP00000342513,ENSP00000354822,ENSP00000359787,ENSP00000360869,ENSP00000360883,ENSP00000368699,ENSP00000380697,ENSP00000381601,ENSP00000382707,ENSP00000386187,ENSP00000388001,ENSP00000483430 | BST2,IFI27,IFI44L,IFI6,IFIT1,IFIT3,IFITM1,IFITM3,IRF7,ISG15,ISG20,MX1,OAS1,XAF1 |
| Cluster16 | CL:22502 | Leber hereditary optic neuropathy | 5 | 7.76753 | top of list | 0.00024 | afc | ENSP00000354499,ENSP00000354554,ENSP00000354632,ENSP00000354961,ENSP00000354982 | MT-ATP6,MT-CO1,MT-CO3,MT-CYB,MT-ND4 |
| Cluster16 | CL:4668 | type I interferon signaling pathway | 12 | 3.15017 | bottom of list | 0.00062 | afc | ENSP00000252593,ENSP00000306565,ENSP00000342513,ENSP00000354822,ENSP00000360869,ENSP00000360883,ENSP00000380697,ENSP00000381601,ENSP00000382707,ENSP00000386187,ENSP00000388001,ENSP00000483430 | BST2,IFI27,IFI6,IFIT1,IFIT3,IFITM1,IFITM3,IRF7,ISG20,MX1,OAS1,XAF1 |
| Cluster16 | CL:22328 | respirasome | 16 | 4.13953 | top of list | 0.001 | afc | ENSP00000258424,ENSP00000321260,ENSP00000332887,ENSP00000354499,ENSP00000354554,ENSP00000354632,ENSP00000354687,ENSP00000354728,ENSP00000354813,ENSP00000354961,ENSP00000354982,ENSP00000355046,ENSP00000355206,ENSP00000367939,ENSP00000418438,ENSP00000419087 | COX5B,COX8A,MT-ATP6,MT-CO1,MT-CO3,MT-CYB,MT-ND1,MT-ND2,MT-ND3,MT-ND4,MT-ND4L,MT-ND5,NDUFA3,NDUFB2,UQCR10,UQCRQ |
| Cluster16 | CL:4670 | type I interferon signaling pathway | 11 | 3.17456 | bottom of list | 0.001 | afc | ENSP00000306565,ENSP00000342513,ENSP00000354822,ENSP00000360869,ENSP00000360883,ENSP00000380697,ENSP00000381601,ENSP00000382707,ENSP00000386187,ENSP00000388001,ENSP00000483430 | IFI27,IFI6,IFIT1,IFIT3,IFITM1,IFITM3,IRF7,ISG20,MX1,OAS1,XAF1 |
| Cluster16 | CL:22408 | Leber hereditary optic neuropathy | 4 | 7.49432 | top of list | 0.0028 | afc | ENSP00000354687,ENSP00000354728,ENSP00000354813,ENSP00000355046 | MT-ND1,MT-ND2,MT-ND4L,MT-ND5 |
| Cluster16 | CL:4615 | neutrophil aggregation, and Aquaporin 9 | 3 | 5.6895 | bottom of list | 0.0035 | afc | ENSP00000357722,ENSP00000357726,ENSP00000357727 | S100A12,S100A8,S100A9 |
| Cluster16 | CL:4560 | immunoglobulin binding, and Immunoregulatory interactions between a Lymphoid and a non-Lymphoid cell | 4 | 4.73505 | bottom of list | 0.0038 | afc | ENSP00000289902,ENSP00000357722,ENSP00000357726,ENSP00000357727 | FCER1G,S100A12,S100A8,S100A9 |
| Cluster16 | CL:4442 | mixed, incl. antigen receptor-mediated signaling pathway, and Immunoregulatory interactions between a Lymphoid and a non-Lymphoid cell | 6 | 3.80822 | bottom of list | 0.0038 | afc | ENSP00000234313,ENSP00000289902,ENSP00000337825,ENSP00000357722,ENSP00000357726,ENSP00000357727 | FCER1G,LCK,PLEK,S100A12,S100A8,S100A9 |
| Cluster | #term ID | term description | genes mapped | enrichment score | direction | false discovery rate | method | matching proteins in your input (IDs) | matching proteins in your input (labels) |
| Cluster17 | CL:22328 | respirasome | 9 | 7.40196 | top of list | 3.28E-05 | afc | ENSP00000354499,ENSP00000354554,ENSP00000354632,ENSP00000354728,ENSP00000354813,ENSP00000354961,ENSP00000354982,ENSP00000355046,ENSP00000355206 | MT-ATP6,MT-CO1,MT-CO3,MT-CYB,MT-ND2,MT-ND3,MT-ND4,MT-ND4L,MT-ND5 |
| Cluster17 | CL:22502 | Leber hereditary optic neuropathy | 5 | 7.36816 | top of list | 0.0015 | afc | ENSP00000354499,ENSP00000354554,ENSP00000354632,ENSP00000354961,ENSP00000354982 | MT-ATP6,MT-CO1,MT-CO3,MT-CYB,MT-ND4 |
| Cluster17 | CL:4662 | Interferon alpha/beta signaling, and Interferon-induced protein 44 family | 16 | 4.51683 | bottom of list | 0.0015 | afc | ENSP00000252593,ENSP00000306565,ENSP00000318982,ENSP00000333657,ENSP00000342513,ENSP00000354822,ENSP00000359787,ENSP00000360883,ENSP00000368699,ENSP00000380697,ENSP00000381601,ENSP00000382707,ENSP00000386187,ENSP00000388001,ENSP00000483430,ENSP00000484689 | BST2,EPSTI1,IFI27,IFI44L,IFI6,IFIT3,IFITM1,IFITM2,IFITM3,IRF7,ISG15,ISG20,MX1,MX2,OAS1,XAF1 |
| **Cluster** | **#term ID** | **term description** | **genes mapped** | **enrichment score** | **direction** | **false discovery rate** | **method** | **matching proteins in your input (IDs)** | **matching proteins in your input (labels)** |
| Cluster17 | CL:4665 | Interferon alpha/beta signaling | 15 | 4.74189 | bottom of list | 0.0015 | afc | ENSP00000252593,ENSP00000306565,ENSP00000333657,ENSP00000342513,ENSP00000354822,ENSP00000359787,ENSP00000360883,ENSP00000368699,ENSP00000380697,ENSP00000381601,ENSP00000382707,ENSP00000386187,ENSP00000388001,ENSP00000483430,ENSP00000484689 | BST2,IFI27,IFI44L,IFI6,IFIT3,IFITM1,IFITM2,IFITM3,IRF7,ISG15,ISG20,MX1,MX2,OAS1,XAF1 |
| Cluster17 | CL:4661 | Interferon alpha/beta signaling, and Interferon gamma signaling | 18 | 4.2548 | bottom of list | 0.0018 | afc | ENSP00000245185,ENSP00000252593,ENSP00000306565,ENSP00000318982,ENSP00000333657,ENSP00000342513,ENSP00000354822,ENSP00000359787,ENSP00000360883,ENSP00000368699,ENSP00000369299,ENSP00000380697,ENSP00000381601,ENSP00000382707,ENSP00000386187,ENSP00000388001,ENSP00000483430,ENSP00000484689 | BST2,EPSTI1,IFI27,IFI44L,IFI6,IFIT3,IFITM1,IFITM2,IFITM3,IRF7,ISG15,ISG20,MT2A,MX1,MX2,OAS1,TRIM22,XAF1 |
| Cluster17 | CL:22332 | mitochondrial respiratory chain complex I | 4 | 7.4442 | top of list | 0.006 | afc | ENSP00000354728,ENSP00000354813,ENSP00000355046,ENSP00000355206 | MT-ND2,MT-ND3,MT-ND4L,MT-ND5 |
| Cluster17 | CL:14985 | Viral mRNA Translation | 3 | 7.85084 | top of list | 0.0092 | afc | ENSP00000341885,ENSP00000348849,ENSP00000375730 | RPL13A,RPS2,RPS26 |
| Cluster | #term ID | term description | genes mapped | enrichment score | direction | false discovery rate | method | matching proteins in your input (IDs) | matching proteins in your input (labels) |
| Cluster18 | CL:22328 | respirasome | 9 | 6.41594 | top of list | 1.75E-05 | afc | ENSP00000354499,ENSP00000354554,ENSP00000354632,ENSP00000354728,ENSP00000354813,ENSP00000354961,ENSP00000354982,ENSP00000355046,ENSP00000355206 | MT-ATP6,MT-CO1,MT-CO3,MT-CYB,MT-ND2,MT-ND3,MT-ND4,MT-ND4L,MT-ND5 |
| Cluster18 | CL:22502 | Leber hereditary optic neuropathy | 5 | 6.35828 | top of list | 0.0011 | afc | ENSP00000354499,ENSP00000354554,ENSP00000354632,ENSP00000354961,ENSP00000354982 | MT-ATP6,MT-CO1,MT-CO3,MT-CYB,MT-ND4 |
| Cluster18 | CL:4661 | Interferon alpha/beta signaling, and Interferon gamma signaling | 18 | 4.24537 | bottom of list | 0.0011 | afc | ENSP00000245185,ENSP00000252593,ENSP00000306565,ENSP00000318982,ENSP00000333657,ENSP00000342513,ENSP00000354822,ENSP00000359787,ENSP00000360883,ENSP00000368699,ENSP00000369299,ENSP00000380697,ENSP00000381601,ENSP00000382707,ENSP00000386187,ENSP00000388001,ENSP00000483430,ENSP00000484689 | BST2,EPSTI1,IFI27,IFI44L,IFI6,IFIT3,IFITM1,IFITM2,IFITM3,IRF7,ISG15,ISG20,MT2A,MX1,MX2,OAS1,TRIM22,XAF1 |
| Cluster18 | CL:4662 | Interferon alpha/beta signaling, and Interferon-induced protein 44 family | 16 | 4.49368 | bottom of list | 0.0011 | afc | ENSP00000252593,ENSP00000306565,ENSP00000318982,ENSP00000333657,ENSP00000342513,ENSP00000354822,ENSP00000359787,ENSP00000360883,ENSP00000368699,ENSP00000380697,ENSP00000381601,ENSP00000382707,ENSP00000386187,ENSP00000388001,ENSP00000483430,ENSP00000484689 | BST2,EPSTI1,IFI27,IFI44L,IFI6,IFIT3,IFITM1,IFITM2,IFITM3,IRF7,ISG15,ISG20,MX1,MX2,OAS1,XAF1 |
| Cluster18 | CL:14985 | Viral mRNA Translation | 5 | 6.61075 | top of list | 0.0011 | afc | ENSP00000341885,ENSP00000346001,ENSP00000346027,ENSP00000348849,ENSP00000362744 | RPL21,RPL3,RPS2,RPS26,RPS4X |
| Cluster18 | CL:4665 | Interferon alpha/beta signaling | 15 | 4.68275 | bottom of list | 0.0011 | afc | ENSP00000252593,ENSP00000306565,ENSP00000333657,ENSP00000342513,ENSP00000354822,ENSP00000359787,ENSP00000360883,ENSP00000368699,ENSP00000380697,ENSP00000381601,ENSP00000382707,ENSP00000386187,ENSP00000388001,ENSP00000483430,ENSP00000484689 | BST2,IFI27,IFI44L,IFI6,IFIT3,IFITM1,IFITM2,IFITM3,IRF7,ISG15,ISG20,MX1,MX2,OAS1,XAF1 |
| Cluster18 | CL:4659 | Interferon alpha/beta signaling, and Interferon gamma signaling | 19 | 4.0439 | bottom of list | 0.0012 | afc | ENSP00000245185,ENSP00000252593,ENSP00000258381,ENSP00000306565,ENSP00000318982,ENSP00000333657,ENSP00000342513,ENSP00000354822,ENSP00000359787,ENSP00000360883,ENSP00000368699,ENSP00000369299,ENSP00000380697,ENSP00000381601,ENSP00000382707,ENSP00000386187,ENSP00000388001,ENSP00000483430,ENSP00000484689 | BST2,EPSTI1,IFI27,IFI44L,IFI6,IFIT3,IFITM1,IFITM2,IFITM3,IRF7,ISG15,ISG20,MT2A,MX1,MX2,OAS1,SP110,TRIM22,XAF1 |
| Cluster18 | CL:22332 | mitochondrial respiratory chain complex I | 4 | 6.48801 | top of list | 0.0024 | afc | ENSP00000354728,ENSP00000354813,ENSP00000355046,ENSP00000355206 | MT-ND2,MT-ND3,MT-ND4L,MT-ND5 |
| Cluster18 | CL:4670 | type I interferon signaling pathway | 12 | 4.15184 | bottom of list | 0.0076 | afc | ENSP00000306565,ENSP00000333657,ENSP00000342513,ENSP00000354822,ENSP00000360883,ENSP00000380697,ENSP00000381601,ENSP00000382707,ENSP00000386187,ENSP00000388001,ENSP00000483430,ENSP00000484689 | IFI27,IFI6,IFIT3,IFITM1,IFITM2,IFITM3,IRF7,ISG20,MX1,MX2,OAS1,XAF1 |
| Cluster18 | CL:22408 | Leber hereditary optic neuropathy | 3 | 6.37393 | top of list | 0.0086 | afc | ENSP00000354728,ENSP00000354813,ENSP00000355046 | MT-ND2,MT-ND4L,MT-ND5 |
| Cluster18 | CL:4668 | type I interferon signaling pathway | 13 | 3.93089 | bottom of list | 0.0086 | afc | ENSP00000252593,ENSP00000306565,ENSP00000333657,ENSP00000342513,ENSP00000354822,ENSP00000360883,ENSP00000380697,ENSP00000381601,ENSP00000382707,ENSP00000386187,ENSP00000388001,ENSP00000483430,ENSP00000484689 | BST2,IFI27,IFI6,IFIT3,IFITM1,IFITM2,IFITM3,IRF7,ISG20,MX1,MX2,OAS1,XAF1 |
| Cluster | #term ID | term description | genes mapped | enrichment score | direction | false discovery rate | method | matching proteins in your input (IDs) | matching proteins in your input (labels) |
| Cluster19 | CL:22328 | respirasome | 10 | 5.78301 | top of list | 6.84E-05 | afc | ENSP00000229379,ENSP00000246554,ENSP00000309565,ENSP00000354554,ENSP00000354632,ENSP00000354728,ENSP00000354813,ENSP00000354961,ENSP00000354982,ENSP00000355206 | COX6A1,COX6B1,MT-ATP6,MT-CO3,MT-CYB,MT-ND3,MT-ND4,MT-ND4L,MT-ND5,UQCRH |
| Cluster19 | CL:22327 | Oxidative phosphorylation | 14 | 4.81869 | top of list | 6.84E-05 | afc | ENSP00000229379,ENSP00000246554,ENSP00000292475,ENSP00000309565,ENSP00000354554,ENSP00000354632,ENSP00000354728,ENSP00000354813,ENSP00000354961,ENSP00000354982,ENSP00000355206,ENSP00000355265,ENSP00000363162,ENSP00000377878 | ATP5G2,ATP5J2,ATP6V1G1,COX6A1,COX6B1,MT-ATP6,MT-ATP8,MT-CO3,MT-CYB,MT-ND3,MT-ND4,MT-ND4L,MT-ND5,UQCRH |
| **Cluster** | **#term ID** | **term description** | **genes mapped** | **enrichment score** | **direction** | **false discovery rate** | **method** | **matching proteins in your input (IDs)** | **matching proteins in your input (labels)** |
| Cluster19 | CL:4665 | Interferon alpha/beta signaling | 14 | 4.09109 | bottom of list | 0.00074 | afc | ENSP00000252593,ENSP00000306565,ENSP00000342513,ENSP00000354822,ENSP00000359787,ENSP00000360883,ENSP00000368699,ENSP00000380697,ENSP00000381601,ENSP00000382707,ENSP00000386187,ENSP00000388001,ENSP00000483430,ENSP00000484689 | BST2,IFI27,IFI44L,IFI6,IFIT3,IFITM1,IFITM2,IFITM3,IRF7,ISG15,ISG20,MX1,OAS1,XAF1 |
| Cluster19 | CL:4662 | Interferon alpha/beta signaling, and Interferon-induced protein 44 family | 15 | 3.82833 | bottom of list | 0.00095 | afc | ENSP00000252593,ENSP00000306565,ENSP00000342513,ENSP00000345494,ENSP00000354822,ENSP00000359787,ENSP00000360883,ENSP00000368699,ENSP00000380697,ENSP00000381601,ENSP00000382707,ENSP00000386187,ENSP00000388001,ENSP00000483430,ENSP00000484689 | BST2,IFI27,IFI44L,IFI6,IFIT3,IFITM1,IFITM2,IFITM3,IRF7,ISG15,ISG20,MX1,OAS1,PLSCR1,XAF1 |
| Cluster19 | CL:22502 | Leber hereditary optic neuropathy | 4 | 7.75608 | top of list | 0.001 | afc | ENSP00000354554,ENSP00000354632,ENSP00000354961,ENSP00000354982 | MT-ATP6,MT-CO3,MT-CYB,MT-ND4 |
| Cluster19 | CL:4661 | Interferon alpha/beta signaling, and Interferon gamma signaling | 16 | 3.58129 | bottom of list | 0.001 | afc | ENSP00000252593,ENSP00000306565,ENSP00000342513,ENSP00000345494,ENSP00000354822,ENSP00000359787,ENSP00000360883,ENSP00000368699,ENSP00000369299,ENSP00000380697,ENSP00000381601,ENSP00000382707,ENSP00000386187,ENSP00000388001,ENSP00000483430,ENSP00000484689 | BST2,IFI27,IFI44L,IFI6,IFIT3,IFITM1,IFITM2,IFITM3,IRF7,ISG15,ISG20,MX1,OAS1,PLSCR1,TRIM22,XAF1 |
| Cluster19 | CL:4659 | Interferon alpha/beta signaling, and Interferon gamma signaling | 17 | 3.375 | bottom of list | 0.0016 | afc | ENSP00000252593,ENSP00000258381,ENSP00000306565,ENSP00000342513,ENSP00000345494,ENSP00000354822,ENSP00000359787,ENSP00000360883,ENSP00000368699,ENSP00000369299,ENSP00000380697,ENSP00000381601,ENSP00000382707,ENSP00000386187,ENSP00000388001,ENSP00000483430,ENSP00000484689 | BST2,IFI27,IFI44L,IFI6,IFIT3,IFITM1,IFITM2,IFITM3,IRF7,ISG15,ISG20,MX1,OAS1,PLSCR1,SP110,TRIM22,XAF1 |
| Cluster19 | CL:22332 | mitochondrial respiratory chain complex I | 3 | 8.41474 | top of list | 0.0019 | afc | ENSP00000354728,ENSP00000354813,ENSP00000355206 | MT-ND3,MT-ND4L,MT-ND5 |
| Cluster19 | CL:4662 | Interferon alpha/beta signaling, and Interferon-induced protein 44 family | 15 | 3.75143 | bottom of list | 0.0003 | afc | ENSP00000252593,ENSP00000318982,ENSP00000326247,ENSP00000333657,ENSP00000342513,ENSP00000345494,ENSP00000354822,ENSP00000359787,ENSP00000360883,ENSP00000368699,ENSP00000380697,ENSP00000381601,ENSP00000382707,ENSP00000386187,ENSP00000388001 | BST2,EPSTI1,IFI44L,IFI6,IFIT3,IFITM1,IFITM3,IRF7,ISG15,MX1,MX2,OAS1,PLSCR1,SAMD9L,XAF1 |
| Cluster19 | CL:4665 | Interferon alpha/beta signaling | 13 | 3.98489 | bottom of list | 0.0003 | afc | ENSP00000252593,ENSP00000326247,ENSP00000333657,ENSP00000342513,ENSP00000354822,ENSP00000359787,ENSP00000360883,ENSP00000368699,ENSP00000380697,ENSP00000381601,ENSP00000382707,ENSP00000386187,ENSP00000388001 | BST2,IFI44L,IFI6,IFIT3,IFITM1,IFITM3,IRF7,ISG15,MX1,MX2,OAS1,SAMD9L,XAF1 |
| Cluster19 | CL:4661 | Interferon alpha/beta signaling, and Interferon gamma signaling | 17 | 3.57248 | bottom of list | 0.0003 | afc | ENSP00000252593,ENSP00000318982,ENSP00000326247,ENSP00000333657,ENSP00000342513,ENSP00000345494,ENSP00000349596,ENSP00000354822,ENSP00000359787,ENSP00000360883,ENSP00000368699,ENSP00000369299,ENSP00000380697,ENSP00000381601,ENSP00000382707,ENSP00000386187,ENSP00000388001 | BST2,EPSTI1,IFI44L,IFI6,IFIT3,IFITM1,IFITM3,IRF7,ISG15,MX1,MX2,OAS1,PLSCR1,SAMD9L,TRIM22,TRIM38,XAF1 |
| Cluster19 | CL:4670 | type I interferon signaling pathway | 9 | 4.18694 | bottom of list | 0.0027 | afc | ENSP00000333657,ENSP00000342513,ENSP00000354822,ENSP00000360883,ENSP00000380697,ENSP00000381601,ENSP00000382707,ENSP00000386187,ENSP00000388001 | IFI6,IFIT3,IFITM1,IFITM3,IRF7,MX1,MX2,OAS1,XAF1 |
| Cluster19 | CL:4668 | type I interferon signaling pathway | 10 | 3.98153 | bottom of list | 0.0027 | afc | ENSP00000252593,ENSP00000333657,ENSP00000342513,ENSP00000354822,ENSP00000360883,ENSP00000380697,ENSP00000381601,ENSP00000382707,ENSP00000386187,ENSP00000388001 | BST2,IFI6,IFIT3,IFITM1,IFITM3,IRF7,MX1,MX2,OAS1,XAF1 |
| Cluster19 | CL:14967 | GTP hydrolysis and joining of the 60S ribosomal subunit, and Protein export | 6 | 5.01673 | top of list | 0.0033 | afc | ENSP00000253039,ENSP00000348849,ENSP00000362744,ENSP00000375730,ENSP00000400467,ENSP00000403172 | EIF2S3,RPL13A,RPL6,RPL9,RPS26,RPS4X |
| Cluster19 | CL:22328 | respirasome | 9 | 3.95348 | top of list | 0.0054 | afc | ENSP00000317780,ENSP00000354554,ENSP00000354687,ENSP00000354728,ENSP00000354813,ENSP00000354961,ENSP00000354982,ENSP00000355046,ENSP00000355206 | COX5A,MT-CO3,MT-CYB,MT-ND1,MT-ND2,MT-ND3,MT-ND4,MT-ND4L,MT-ND5 |
| Cluster19 | CL:14985 | Viral mRNA Translation | 5 | 4.94112 | top of list | 0.0075 | afc | ENSP00000348849,ENSP00000362744,ENSP00000375730,ENSP00000400467,ENSP00000403172 | RPL13A,RPL6,RPL9,RPS26,RPS4X |
| Cluster19 | CL:14963 | GTP hydrolysis and joining of the 60S ribosomal subunit, and Nonsense-mediated mRNA decay | 7 | 4.1507 | top of list | 0.0089 | afc | ENSP00000253039,ENSP00000336702,ENSP00000348849,ENSP00000362744,ENSP00000375730,ENSP00000400467,ENSP00000403172 | EIF2S3,EIF5A,RPL13A,RPL6,RPL9,RPS26,RPS4X |
| Cluster19 | CL:18627 | MHC class II protein complex, and dynactin complex | 5 | 7.18329 | both ends | 0.0012 | afc | ENSP00000353099,ENSP00000364076,ENSP00000364080,ENSP00000364114,ENSP00000378786 | HLA-DQA2,HLA-DQB1,HLA-DRA,HLA-DRB1,HLA-DRB5 |
| **Cluster** | **#term ID** | **term description** | **genes mapped** | **enrichment score** | **direction** | **false discovery rate** | **method** | **matching proteins in your input (IDs)** | **matching proteins in your input (labels)** |
| Cluster19 | CL:18630 | MHC class II protein complex | 4 | 7.50974 | both ends | 0.0018 | afc | ENSP00000353099,ENSP00000364080,ENSP00000364114,ENSP00000378786 | HLA-DQB1,HLA-DRA,HLA-DRB1,HLA-DRB5 |
| Cluster19 | CL:4662 | Interferon alpha/beta signaling, and Interferon-induced protein 44 family | 20 | 2.63221 | bottom of list | 0.0093 | afc | ENSP00000245414,ENSP00000252593,ENSP00000306565,ENSP00000318982,ENSP00000326247,ENSP00000342278,ENSP00000342513,ENSP00000345494,ENSP00000354822,ENSP00000359787,ENSP00000360869,ENSP00000360883,ENSP00000368699,ENSP00000380697,ENSP00000381601,ENSP00000382707,ENSP00000386187,ENSP00000388001,ENSP00000483430,ENSP00000484689 | BST2,EPSTI1,IFI27,IFI44L,IFI6,IFIT1,IFIT3,IFITM1,IFITM2,IFITM3,IRF1,IRF7,ISG15,ISG20,MX1,OAS1,OAS2,PLSCR1,SAMD9L,XAF1 |
| Cluster19 | CL:4670 | type I interferon signaling pathway | 13 | 3.27694 | bottom of list | 0.0093 | afc | ENSP00000306565,ENSP00000342278,ENSP00000342513,ENSP00000354822,ENSP00000360869,ENSP00000360883,ENSP00000380697,ENSP00000381601,ENSP00000382707,ENSP00000386187,ENSP00000388001,ENSP00000483430,ENSP00000484689 | IFI27,IFI6,IFIT1,IFIT3,IFITM1,IFITM2,IFITM3,IRF7,ISG20,MX1,OAS1,OAS2,XAF1 |
| Cluster19 | CL:4665 | Interferon alpha/beta signaling | 18 | 2.84473 | bottom of list | 0.0093 | afc | ENSP00000245414,ENSP00000252593,ENSP00000306565,ENSP00000326247,ENSP00000342278,ENSP00000342513,ENSP00000354822,ENSP00000359787,ENSP00000360869,ENSP00000360883,ENSP00000368699,ENSP00000380697,ENSP00000381601,ENSP00000382707,ENSP00000386187,ENSP00000388001,ENSP00000483430,ENSP00000484689 | BST2,IFI27,IFI44L,IFI6,IFIT1,IFIT3,IFITM1,IFITM2,IFITM3,IRF1,IRF7,ISG15,ISG20,MX1,OAS1,OAS2,SAMD9L,XAF1 |
| Cluster19 | CL:4670 | type I interferon signaling pathway | 11 | 3.71783 | bottom of list | 0.0028 | afc | ENSP00000306565,ENSP00000342513,ENSP00000354822,ENSP00000360883,ENSP00000380697,ENSP00000381601,ENSP00000382707,ENSP00000386187,ENSP00000388001,ENSP00000483430,ENSP00000484689 | IFI27,IFI6,IFIT3,IFITM1,IFITM2,IFITM3,IRF7,ISG20,MX1,OAS1,XAF1 |
| Cluster19 | CL:4668 | type I interferon signaling pathway | 12 | 3.61643 | bottom of list | 0.0028 | afc | ENSP00000252593,ENSP00000306565,ENSP00000342513,ENSP00000354822,ENSP00000360883,ENSP00000380697,ENSP00000381601,ENSP00000382707,ENSP00000386187,ENSP00000388001,ENSP00000483430,ENSP00000484689 | BST2,IFI27,IFI6,IFIT3,IFITM1,IFITM2,IFITM3,IRF7,ISG20,MX1,OAS1,XAF1 |
| Cluster19 | CL:22446 | cytochrome complex, and Cytochrome c oxidase subunit VII | 7 | 4.65513 | top of list | 0.0056 | afc | ENSP00000229379,ENSP00000246554,ENSP00000309565,ENSP00000354554,ENSP00000354632,ENSP00000354961,ENSP00000354982 | COX6A1,COX6B1,MT-ATP6,MT-CO3,MT-CYB,MT-ND4,UQCRH |
| Cluster19 | CL:14985 | Viral mRNA Translation | 3 | 6.34485 | top of list | 0.006 | afc | ENSP00000348849,ENSP00000354739,ENSP00000375730 | RPL12,RPL13A,RPS26 |
| Cluster | #term ID | term description | genes mapped | enrichment score | direction | false discovery rate | method | matching proteins in your input (IDs) | matching proteins in your input (labels) |
| Cluster20 | CL:22328 | respirasome | 12 | 5.51956 | top of list | 5.80E-07 | afc | ENSP00000354499,ENSP00000354554,ENSP00000354632,ENSP00000354687,ENSP00000354728,ENSP00000354813,ENSP00000354876,ENSP00000354961,ENSP00000354982,ENSP00000355046,ENSP00000355206,ENSP00000418438 | MT-ATP6,MT-CO1,MT-CO2,MT-CO3,MT-CYB,MT-ND1,MT-ND2,MT-ND3,MT-ND4,MT-ND4L,MT-ND5,NDUFA3 |
| Cluster20 | CL:22327 | Oxidative phosphorylation | 13 | 5.52469 | top of list | 5.80E-07 | afc | ENSP00000354499,ENSP00000354554,ENSP00000354632,ENSP00000354687,ENSP00000354728,ENSP00000354813,ENSP00000354876,ENSP00000354961,ENSP00000354982,ENSP00000355046,ENSP00000355206,ENSP00000355265,ENSP00000418438 | MT-ATP6,MT-ATP8,MT-CO1,MT-CO2,MT-CO3,MT-CYB,MT-ND1,MT-ND2,MT-ND3,MT-ND4,MT-ND4L,MT-ND5,NDUFA3 |
| Cluster20 | CL:4662 | Interferon alpha/beta signaling, and Interferon-induced protein 44 family | 20 | 2.67437 | bottom of list | 3.25E-05 | afc | ENSP00000228928,ENSP00000257570,ENSP00000306565,ENSP00000318982,ENSP00000333657,ENSP00000342513,ENSP00000345494,ENSP00000354822,ENSP00000359787,ENSP00000360869,ENSP00000360883,ENSP00000360891,ENSP00000368699,ENSP00000371471,ENSP00000380697,ENSP00000381601,ENSP00000382707,ENSP00000386187,ENSP00000395590,ENSP00000483430 | EPSTI1,IFI27,IFI35,IFI44L,IFI6,IFIT1,IFIT2,IFIT3,IFITM1,IFITM3,IRF7,ISG15,ISG20,MX1,MX2,OAS3,OASL,PLSCR1,RSAD2,XAF1 |
| Cluster20 | CL:22446 | cytochrome complex, and Cytochrome c oxidase subunit VII | 6 | 6.47367 | top of list | 3.25E-05 | afc | ENSP00000354499,ENSP00000354554,ENSP00000354632,ENSP00000354876,ENSP00000354961,ENSP00000354982 | MT-ATP6,MT-CO1,MT-CO2,MT-CO3,MT-CYB,MT-ND4 |
| Cluster20 | CL:4661 | Interferon alpha/beta signaling, and Interferon gamma signaling | 24 | 2.46991 | bottom of list | 3.25E-05 | ks | ENSP00000228928,ENSP00000245185,ENSP00000257570,ENSP00000306565,ENSP00000318982,ENSP00000333657,ENSP00000342513,ENSP00000345494,ENSP00000354822,ENSP00000358165,ENSP00000359504,ENSP00000359787,ENSP00000360869,ENSP00000360883,ENSP00000360891,ENSP00000368699,ENSP00000369299,ENSP00000371471,ENSP00000380697,ENSP00000381601,ENSP00000382707,ENSP00000386187,ENSP00000395590,ENSP00000483430 | EPSTI1,FCGR1A,GBP1,IFI27,IFI35,IFI44L,IFI6,IFIT1,IFIT2,IFIT3,IFITM1,IFITM3,IRF7,ISG15,ISG20,MT2A,MX1,MX2,OAS3,OASL,PLSCR1,RSAD2,TRIM22,XAF1 |
| Cluster20 | CL:4665 | Interferon alpha/beta signaling | 18 | 2.76521 | bottom of list | 5.80E-05 | afc | ENSP00000228928,ENSP00000257570,ENSP00000306565,ENSP00000333657,ENSP00000342513,ENSP00000354822,ENSP00000359787,ENSP00000360869,ENSP00000360883,ENSP00000360891,ENSP00000368699,ENSP00000371471,ENSP00000380697,ENSP00000381601,ENSP00000382707,ENSP00000386187,ENSP00000395590,ENSP00000483430 | IFI27,IFI35,IFI44L,IFI6,IFIT1,IFIT2,IFIT3,IFITM1,IFITM3,IRF7,ISG15,ISG20,MX1,MX2,OAS3,OASL,RSAD2,XAF1 |
| Cluster20 | CL:22502 | Leber hereditary optic neuropathy | 5 | 6.61582 | top of list | 0.00011 | afc | ENSP00000354499,ENSP00000354554,ENSP00000354632,ENSP00000354961,ENSP00000354982 | MT-ATP6,MT-CO1,MT-CO3,MT-CYB,MT-ND4 |
| Cluster20 | CL:4670 | type I interferon signaling pathway | 16 | 2.69791 | bottom of list | 0.00019 | afc | ENSP00000228928,ENSP00000257570,ENSP00000306565,ENSP00000333657,ENSP00000342513,ENSP00000354822,ENSP00000360869,ENSP00000360883,ENSP00000360891,ENSP00000371471,ENSP00000380697,ENSP00000381601,ENSP00000382707,ENSP00000386187,ENSP00000395590,ENSP00000483430 | IFI27,IFI35,IFI6,IFIT1,IFIT2,IFIT3,IFITM1,IFITM3,IRF7,ISG20,MX1,MX2,OAS3,OASL,RSAD2,XAF1 |
| **Cluster** | **#term ID** | **term description** | **genes mapped** | **enrichment score** | **direction** | **false discovery rate** | **method** | **matching proteins in your input (IDs)** | **matching proteins in your input (labels)** |
| Cluster20 | CL:14966 | GTP hydrolysis and joining of the 60S ribosomal subunit, and Protein export | 20 | 3.26209 | top of list | 0.00044 | afc | ENSP00000307940,ENSP00000311430,ENSP00000313007,ENSP00000331019,ENSP00000339063,ENSP00000341885,ENSP00000346001,ENSP00000346027,ENSP00000359345,ENSP00000362744,ENSP00000363018,ENSP00000369757,ENSP00000376056,ENSP00000388806,ENSP00000403172,ENSP00000413436,ENSP00000431800,ENSP00000452909,ENSP00000463784,ENSP00000485663 | EEF1A1,EEF1B2,EEF2,EIF3F,EIF3L,EIF4B,PABPC1,RPL10,RPL10A,RPL21,RPL26,RPL28,RPL3,RPL4,RPL5,RPL6,RPS2,RPS27L,RPS4X,RPS6 |
| Cluster20 | CL:14967 | GTP hydrolysis and joining of the 60S ribosomal subunit, and Protein export | 19 | 3.23809 | top of list | 0.00054 | afc | ENSP00000307940,ENSP00000311430,ENSP00000313007,ENSP00000331019,ENSP00000339063,ENSP00000341885,ENSP00000346001,ENSP00000346027,ENSP00000359345,ENSP00000362744,ENSP00000363018,ENSP00000369757,ENSP00000388806,ENSP00000403172,ENSP00000413436,ENSP00000431800,ENSP00000452909,ENSP00000463784,ENSP00000485663 | EEF1A1,EEF2,EIF3F,EIF3L,EIF4B,PABPC1,RPL10,RPL10A,RPL21,RPL26,RPL28,RPL3,RPL4,RPL5,RPL6,RPS2,RPS27L,RPS4X,RPS6 |
| Cluster20 | CL:14963 | GTP hydrolysis and joining of the 60S ribosomal subunit, and Nonsense-mediated mRNA decay | 21 | 3.26007 | top of list | 0.00054 | ks | ENSP00000307940,ENSP00000311430,ENSP00000313007,ENSP00000331019,ENSP00000339063,ENSP00000341885,ENSP00000346001,ENSP00000346027,ENSP00000359345,ENSP00000362744,ENSP00000363018,ENSP00000369757,ENSP00000376056,ENSP00000388806,ENSP00000403172,ENSP00000413436,ENSP00000431800,ENSP00000448035,ENSP00000452909,ENSP00000463784,ENSP00000485663 | EEF1A1,EEF1B2,EEF2,EIF3F,EIF3L,EIF4B,NACA,PABPC1,RPL10,RPL10A,RPL21,RPL26,RPL28,RPL3,RPL4,RPL5,RPL6,RPS2,RPS27L,RPS4X,RPS6 |
| Cluster20 | CL:14978 | Peptide chain elongation | 15 | 3.03466 | top of list | 0.0063 | afc | ENSP00000307940,ENSP00000311430,ENSP00000331019,ENSP00000339063,ENSP00000341885,ENSP00000346001,ENSP00000346027,ENSP00000359345,ENSP00000362744,ENSP00000363018,ENSP00000369757,ENSP00000403172,ENSP00000413436,ENSP00000452909,ENSP00000463784 | EEF1A1,EEF2,RPL10,RPL10A,RPL21,RPL26,RPL28,RPL3,RPL4,RPL5,RPL6,RPS2,RPS27L,RPS4X,RPS6 |
| Cluster20 | CL:22332 | mitochondrial respiratory chain complex I | 6 | 4.56545 | top of list | 0.0077 | afc | ENSP00000354687,ENSP00000354728,ENSP00000354813,ENSP00000355046,ENSP00000355206,ENSP00000418438 | MT-ND1,MT-ND2,MT-ND3,MT-ND4L,MT-ND5,NDUFA3 |
| Cluster20 | CL:14982 | Peptide chain elongation | 12 | 3.20677 | top of list | 0.0083 | afc | ENSP00000307940,ENSP00000341885,ENSP00000346001,ENSP00000346027,ENSP00000359345,ENSP00000362744,ENSP00000363018,ENSP00000369757,ENSP00000403172,ENSP00000413436,ENSP00000452909,ENSP00000463784 | EEF2,RPL10,RPL10A,RPL21,RPL26,RPL28,RPL3,RPL5,RPL6,RPS2,RPS4X,RPS6 |
| Cluster | #term ID | term description | genes mapped | enrichment score | direction | false discovery rate | method | matching proteins in your input (IDs) | matching proteins in your input (labels) |
| Cluster21 | CL:14966 | GTP hydrolysis and joining of the 60S ribosomal subunit, and Protein export | 16 | 5.53519 | top of list | 8.80E-06 | afc | ENSP00000307940,ENSP00000311430,ENSP00000313007,ENSP00000331019,ENSP00000341885,ENSP00000346001,ENSP00000348849,ENSP00000359345,ENSP00000362744,ENSP00000369757,ENSP00000376056,ENSP00000379888,ENSP00000388806,ENSP00000420311,ENSP00000449328,ENSP00000477781 | EEF1B2,EEF2,EIF4B,PABPC1,RPL23,RPL3,RPL4,RPL5,RPLP0,RPS2,RPS26,RPS27L,RPS4X,RPS6,RPS8,TPT1 |
| Cluster21 | CL:14967 | GTP hydrolysis and joining of the 60S ribosomal subunit, and Protein export | 14 | 5.43127 | top of list | 2.90E-05 | afc | ENSP00000307940,ENSP00000311430,ENSP00000313007,ENSP00000331019,ENSP00000341885,ENSP00000346001,ENSP00000348849,ENSP00000359345,ENSP00000362744,ENSP00000369757,ENSP00000379888,ENSP00000388806,ENSP00000420311,ENSP00000449328 | EEF2,EIF4B,PABPC1,RPL23,RPL3,RPL4,RPL5,RPLP0,RPS2,RPS26,RPS27L,RPS4X,RPS6,RPS8 |
| Cluster21 | CL:14982 | Peptide chain elongation | 10 | 5.85038 | top of list | 0.00012 | afc | ENSP00000307940,ENSP00000341885,ENSP00000346001,ENSP00000348849,ENSP00000359345,ENSP00000362744,ENSP00000369757,ENSP00000379888,ENSP00000420311,ENSP00000449328 | EEF2,RPL23,RPL3,RPL5,RPLP0,RPS2,RPS26,RPS4X,RPS6,RPS8 |
| Cluster21 | CL:14978 | Peptide chain elongation | 12 | 5.3073 | top of list | 0.00015 | afc | ENSP00000307940,ENSP00000311430,ENSP00000331019,ENSP00000341885,ENSP00000346001,ENSP00000348849,ENSP00000359345,ENSP00000362744,ENSP00000369757,ENSP00000379888,ENSP00000420311,ENSP00000449328 | EEF2,RPL23,RPL3,RPL4,RPL5,RPLP0,RPS2,RPS26,RPS27L,RPS4X,RPS6,RPS8 |
| Cluster21 | CL:14980 | Peptide chain elongation | 11 | 5.27979 | top of list | 0.00028 | afc | ENSP00000307940,ENSP00000331019,ENSP00000341885,ENSP00000346001,ENSP00000348849,ENSP00000359345,ENSP00000362744,ENSP00000369757,ENSP00000379888,ENSP00000420311,ENSP00000449328 | EEF2,RPL23,RPL3,RPL5,RPLP0,RPS2,RPS26,RPS27L,RPS4X,RPS6,RPS8 |
| Cluster21 | CL:14983 | Peptide chain elongation | 8 | 5.88552 | top of list | 0.00062 | afc | ENSP00000307940,ENSP00000341885,ENSP00000346001,ENSP00000348849,ENSP00000362744,ENSP00000369757,ENSP00000379888,ENSP00000420311 | EEF2,RPL23,RPL3,RPS2,RPS26,RPS4X,RPS6,RPS8 |
| Cluster21 | CL:22328 | respirasome | 6 | 6.52688 | top of list | 0.00074 | afc | ENSP00000354632,ENSP00000354728,ENSP00000354813,ENSP00000354961,ENSP00000355046,ENSP00000355206 | MT-ATP6,MT-ND2,MT-ND3,MT-ND4,MT-ND4L,MT-ND5 |
| Cluster21 | CL:4662 | Interferon alpha/beta signaling, and Interferon-induced protein 44 family | 20 | 1.92716 | bottom of list | 0.0011 | afc | ENSP00000228928,ENSP00000252593,ENSP00000306565,ENSP00000318982,ENSP00000333657,ENSP00000342513,ENSP00000354822,ENSP00000359783,ENSP00000359787,ENSP00000360883,ENSP00000360891,ENSP00000368699,ENSP00000380697,ENSP00000381601,ENSP00000382707,ENSP00000386187,ENSP00000388001,ENSP00000395590,ENSP00000483430,ENSP00000484689 | BST2,EPSTI1,IFI27,IFI35,IFI44,IFI44L,IFI6,IFIT2,IFIT3,IFITM1,IFITM2,IFITM3,IRF7,ISG15,ISG20,MX1,MX2,OAS1,OAS3,XAF1 |
| Cluster21 | CL:14985 | Viral mRNA Translation | 7 | 5.87055 | top of list | 0.0011 | afc | ENSP00000341885,ENSP00000346001,ENSP00000348849,ENSP00000362744,ENSP00000369757,ENSP00000379888,ENSP00000420311 | RPL23,RPL3,RPS2,RPS26,RPS4X,RPS6,RPS8 |
| Cluster21 | CL:4665 | Interferon alpha/beta signaling | 19 | 1.95153 | bottom of list | 0.0012 | afc | ENSP00000228928,ENSP00000252593,ENSP00000306565,ENSP00000333657,ENSP00000342513,ENSP00000354822,ENSP00000359783,ENSP00000359787,ENSP00000360883,ENSP00000360891,ENSP00000368699,ENSP00000380697,ENSP00000381601,ENSP00000382707,ENSP00000386187,ENSP00000388001,ENSP00000395590,ENSP00000483430,ENSP00000484689 | BST2,IFI27,IFI35,IFI44,IFI44L,IFI6,IFIT2,IFIT3,IFITM1,IFITM2,IFITM3,IRF7,ISG15,ISG20,MX1,MX2,OAS1,OAS3,XAF1 |
| **Cluster** | **#term ID** | **term description** | **genes mapped** | **enrichment score** | **direction** | **false discovery rate** | **method** | **matching proteins in your input (IDs)** | **matching proteins in your input (labels)** |
| Cluster21 | CL:22327 | Oxidative phosphorylation | 7 | 5.45043 | top of list | 0.0026 | afc | ENSP00000354632,ENSP00000354728,ENSP00000354813,ENSP00000354961,ENSP00000355046,ENSP00000355206,ENSP00000362329 | MT-ATP6,MT-ND2,MT-ND3,MT-ND4,MT-ND4L,MT-ND5,PPA1 |
| Cluster21 | CL:4668 | type I interferon signaling pathway | 16 | 1.9938 | bottom of list | 0.0027 | afc | ENSP00000228928,ENSP00000252593,ENSP00000306565,ENSP00000333657,ENSP00000342513,ENSP00000354822,ENSP00000360883,ENSP00000360891,ENSP00000380697,ENSP00000381601,ENSP00000382707,ENSP00000386187,ENSP00000388001,ENSP00000395590,ENSP00000483430,ENSP00000484689 | BST2,IFI27,IFI35,IFI6,IFIT2,IFIT3,IFITM1,IFITM2,IFITM3,IRF7,ISG20,MX1,MX2,OAS1,OAS3,XAF1 |
| Cluster21 | CL:22332 | mitochondrial respiratory chain complex I | 4 | 6.5338 | top of list | 0.0053 | afc | ENSP00000354728,ENSP00000354813,ENSP00000355046,ENSP00000355206 | MT-ND2,MT-ND3,MT-ND4L,MT-ND5 |
| Cluster | #term ID | term description | genes mapped | enrichment score | direction | false discovery rate | method | matching proteins in your input (IDs) | matching proteins in your input (labels) |
| Cluster22 | CL:4665 | Interferon alpha/beta signaling | 11 | 5.51434 | bottom of list | 2.55E-05 | afc | ENSP00000252593,ENSP00000342513,ENSP00000354822,ENSP00000359787,ENSP00000368699,ENSP00000380697,ENSP00000381601,ENSP00000382707,ENSP00000386187,ENSP00000395590,ENSP00000483430 | BST2,IFI27,IFI35,IFI44L,IFI6,IFITM1,IFITM3,IRF7,ISG15,MX1,XAF1 |
| Cluster22 | CL:4668 | type I interferon signaling pathway | 9 | 5.47227 | bottom of list | 0.00012 | afc | ENSP00000252593,ENSP00000342513,ENSP00000354822,ENSP00000380697,ENSP00000381601,ENSP00000382707,ENSP00000386187,ENSP00000395590,ENSP00000483430 | BST2,IFI27,IFI35,IFI6,IFITM1,IFITM3,IRF7,MX1,XAF1 |
| Cluster22 | CL:4670 | type I interferon signaling pathway | 8 | 5.72362 | bottom of list | 0.00013 | afc | ENSP00000342513,ENSP00000354822,ENSP00000380697,ENSP00000381601,ENSP00000382707,ENSP00000386187,ENSP00000395590,ENSP00000483430 | IFI27,IFI35,IFI6,IFITM1,IFITM3,IRF7,MX1,XAF1 |
| Cluster | #term ID | term description | genes mapped | enrichment score | direction | false discovery rate | method | matching proteins in your input (IDs) | matching proteins in your input (labels) |
| Cluster24 | CL:22328 | respirasome | 12 | 6.83809 | top of list | 4.10E-07 | afc | ENSP00000332887,ENSP00000354499,ENSP00000354554,ENSP00000354632,ENSP00000354687,ENSP00000354728,ENSP00000354813,ENSP00000354876,ENSP00000354961,ENSP00000354982,ENSP00000355046,ENSP00000355206 | MT-ATP6,MT-CO1,MT-CO2,MT-CO3,MT-CYB,MT-ND1,MT-ND2,MT-ND3,MT-ND4,MT-ND4L,MT-ND5,UQCR10 |
| Cluster24 | CL:22327 | Oxidative phosphorylation | 14 | 6.25417 | top of list | 4.10E-07 | afc | ENSP00000332887,ENSP00000354499,ENSP00000354554,ENSP00000354632,ENSP00000354687,ENSP00000354728,ENSP00000354813,ENSP00000354876,ENSP00000354961,ENSP00000354982,ENSP00000355046,ENSP00000355206,ENSP00000355265,ENSP00000429690 | ATP6V0E1,MT-ATP6,MT-ATP8,MT-CO1,MT-CO2,MT-CO3,MT-CYB,MT-ND1,MT-ND2,MT-ND3,MT-ND4,MT-ND4L,MT-ND5,UQCR10 |
| Cluster24 | CL:4665 | Interferon alpha/beta signaling | 20 | 4.75003 | bottom of list | 6.29E-06 | afc | ENSP00000252593,ENSP00000257570,ENSP00000306565,ENSP00000326247,ENSP00000333657,ENSP00000342513,ENSP00000354822,ENSP00000359787,ENSP00000360869,ENSP00000360883,ENSP00000368699,ENSP00000371471,ENSP00000380697,ENSP00000381601,ENSP00000382707,ENSP00000386187,ENSP00000388001,ENSP00000395590,ENSP00000483430,ENSP00000484689 | BST2,IFI27,IFI35,IFI44L,IFI6,IFIT1,IFIT3,IFITM1,IFITM2,IFITM3,IRF7,ISG15,ISG20,MX1,MX2,OAS1,OASL,RSAD2,SAMD9L,XAF1 |
| Cluster24 | CL:22502 | Leber hereditary optic neuropathy | 5 | 8.24144 | top of list | 2.67E-05 | afc | ENSP00000354499,ENSP00000354554,ENSP00000354632,ENSP00000354961,ENSP00000354982 | MT-ATP6,MT-CO1,MT-CO3,MT-CYB,MT-ND4 |
| Cluster24 | CL:14966 | GTP hydrolysis and joining of the 60S ribosomal subunit, and Protein export | 14 | 5.01961 | top of list | 6.23E-05 | afc | ENSP00000272317,ENSP00000307889,ENSP00000339095,ENSP00000341885,ENSP00000346027,ENSP00000348849,ENSP00000362744,ENSP00000375730,ENSP00000376056,ENSP00000388806,ENSP00000404375,ENSP00000419449,ENSP00000463784,ENSP00000470972 | EEF1B2,EIF1,EIF4B,RPL13,RPL13A,RPL21,RPL26,RPL36A,RPS19,RPS2,RPS26,RPS27A,RPS4X,RPS7 |
| Cluster24 | CL:14967 | GTP hydrolysis and joining of the 60S ribosomal subunit, and Protein export | 13 | 5.06972 | top of list | 7.03E-05 | afc | ENSP00000272317,ENSP00000307889,ENSP00000339095,ENSP00000341885,ENSP00000346027,ENSP00000348849,ENSP00000362744,ENSP00000375730,ENSP00000388806,ENSP00000404375,ENSP00000419449,ENSP00000463784,ENSP00000470972 | EIF1,EIF4B,RPL13,RPL13A,RPL21,RPL26,RPL36A,RPS19,RPS2,RPS26,RPS27A,RPS4X,RPS7 |
| Cluster24 | CL:4668 | type I interferon signaling pathway | 17 | 4.51625 | bottom of list | 7.03E-05 | afc | ENSP00000252593,ENSP00000257570,ENSP00000306565,ENSP00000333657,ENSP00000342513,ENSP00000354822,ENSP00000360869,ENSP00000360883,ENSP00000371471,ENSP00000380697,ENSP00000381601,ENSP00000382707,ENSP00000386187,ENSP00000388001,ENSP00000395590,ENSP00000483430,ENSP00000484689 | BST2,IFI27,IFI35,IFI6,IFIT1,IFIT3,IFITM1,IFITM2,IFITM3,IRF7,ISG20,MX1,MX2,OAS1,OASL,RSAD2,XAF1 |
| Cluster24 | CL:22446 | cytochrome complex, and Cytochrome c oxidase subunit VII | 7 | 6.60951 | top of list | 0.00011 | afc | ENSP00000332887,ENSP00000354499,ENSP00000354554,ENSP00000354632,ENSP00000354876,ENSP00000354961,ENSP00000354982 | MT-ATP6,MT-CO1,MT-CO2,MT-CO3,MT-CYB,MT-ND4,UQCR10 |
| Cluster24 | CL:4670 | type I interferon signaling pathway | 16 | 4.50027 | bottom of list | 0.00013 | afc | ENSP00000257570,ENSP00000306565,ENSP00000333657,ENSP00000342513,ENSP00000354822,ENSP00000360869,ENSP00000360883,ENSP00000371471,ENSP00000380697,ENSP00000381601,ENSP00000382707,ENSP00000386187,ENSP00000388001,ENSP00000395590,ENSP00000483430,ENSP00000484689 | IFI27,IFI35,IFI6,IFIT1,IFIT3,IFITM1,IFITM2,IFITM3,IRF7,ISG20,MX1,MX2,OAS1,OASL,RSAD2,XAF1 |
| Cluster24 | CL:14983 | Peptide chain elongation | 11 | 5.16551 | top of list | 0.00019 | afc | ENSP00000272317,ENSP00000307889,ENSP00000339095,ENSP00000341885,ENSP00000346027,ENSP00000348849,ENSP00000362744,ENSP00000375730,ENSP00000404375,ENSP00000463784,ENSP00000470972 | RPL13,RPL13A,RPL21,RPL26,RPL36A,RPS19,RPS2,RPS26,RPS27A,RPS4X,RPS7 |
| **Cluster** | **#term ID** | **term description** | **genes mapped** | **enrichment score** | **direction** | **false discovery rate** | **method** | **matching proteins in your input (IDs)** | **matching proteins in your input (labels)** |
| Cluster24 | CL:14985 | Viral mRNA Translation | 10 | 5.24029 | top of list | 0.00025 | afc | ENSP00000272317,ENSP00000307889,ENSP00000339095,ENSP00000341885,ENSP00000346027,ENSP00000348849,ENSP00000362744,ENSP00000375730,ENSP00000463784,ENSP00000470972 | RPL13,RPL13A,RPL21,RPL26,RPS19,RPS2,RPS26,RPS27A,RPS4X,RPS7 |
| Cluster24 | CL:22332 | mitochondrial respiratory chain complex I | 5 | 7.1581 | top of list | 0.00038 | afc | ENSP00000354687,ENSP00000354728,ENSP00000354813,ENSP00000355046,ENSP00000355206 | MT-ND1,MT-ND2,MT-ND3,MT-ND4L,MT-ND5 |
| Cluster24 | CL:22408 | Leber hereditary optic neuropathy | 4 | 7.10827 | top of list | 0.0017 | afc | ENSP00000354687,ENSP00000354728,ENSP00000354813,ENSP00000355046 | MT-ND1,MT-ND2,MT-ND4L,MT-ND5 |
| Cluster24 | CL:4662 | Interferon alpha/beta signaling, and Interferon-induced protein 44 family | 22 | 4.63798 | bottom of list | 0.004 | ks | ENSP00000252593,ENSP00000257570,ENSP00000306565,ENSP00000318982,ENSP00000326247,ENSP00000333657,ENSP00000342513,ENSP00000345494,ENSP00000354822,ENSP00000359787,ENSP00000360869,ENSP00000360883,ENSP00000368699,ENSP00000371471,ENSP00000380697,ENSP00000381601,ENSP00000382707,ENSP00000386187,ENSP00000388001,ENSP00000395590,ENSP00000483430,ENSP00000484689 | BST2,EPSTI1,IFI27,IFI35,IFI44L,IFI6,IFIT1,IFIT3,IFITM1,IFITM2,IFITM3,IRF7,ISG15,ISG20,MX1,MX2,OAS1,OASL,PLSCR1,RSAD2,SAMD9L,XAF1 |
| Cluster24 | CL:4659 | Interferon alpha/beta signaling, and Interferon gamma signaling | 26 | 4.26662 | bottom of list | 0.005 | ks | ENSP00000245185,ENSP00000252593,ENSP00000257570,ENSP00000258381,ENSP00000306565,ENSP00000318982,ENSP00000326247,ENSP00000333657,ENSP00000342513,ENSP00000345494,ENSP00000354822,ENSP00000359504,ENSP00000359787,ENSP00000360869,ENSP00000360883,ENSP00000368699,ENSP00000369299,ENSP00000371471,ENSP00000380697,ENSP00000381601,ENSP00000382707,ENSP00000386187,ENSP00000388001,ENSP00000395590,ENSP00000483430,ENSP00000484689 | BST2,EPSTI1,GBP1,IFI27,IFI35,IFI44L,IFI6,IFIT1,IFIT3,IFITM1,IFITM2,IFITM3,IRF7,ISG15,ISG20,MT2A,MX1,MX2,OAS1,OASL,PLSCR1,RSAD2,SAMD9L,SP110,TRIM22,XAF1 |
| Cluster24 | CL:4661 | Interferon alpha/beta signaling, and Interferon gamma signaling | 25 | 4.36816 | bottom of list | 0.005 | ks | ENSP00000245185,ENSP00000252593,ENSP00000257570,ENSP00000306565,ENSP00000318982,ENSP00000326247,ENSP00000333657,ENSP00000342513,ENSP00000345494,ENSP00000354822,ENSP00000359504,ENSP00000359787,ENSP00000360869,ENSP00000360883,ENSP00000368699,ENSP00000369299,ENSP00000371471,ENSP00000380697,ENSP00000381601,ENSP00000382707,ENSP00000386187,ENSP00000388001,ENSP00000395590,ENSP00000483430,ENSP00000484689 | BST2,EPSTI1,GBP1,IFI27,IFI35,IFI44L,IFI6,IFIT1,IFIT3,IFITM1,IFITM2,IFITM3,IRF7,ISG15,ISG20,MT2A,MX1,MX2,OAS1,OASL,PLSCR1,RSAD2,SAMD9L,TRIM22,XAF1 |
| Cluster24 | CL:4673 | 2'-5'-oligoadenylate synthase, and type I interferon signaling pathway | 11 | 4.05035 | bottom of list | 0.0063 | afc | ENSP00000257570,ENSP00000333657,ENSP00000342513,ENSP00000354822,ENSP00000360869,ENSP00000360883,ENSP00000371471,ENSP00000380697,ENSP00000381601,ENSP00000388001,ENSP00000395590 | IFI35,IFI6,IFIT1,IFIT3,IRF7,MX1,MX2,OAS1,OASL,RSAD2,XAF1 |
| Cluster | #term ID | term description | genes mapped | enrichment score | direction | false discovery rate | method | matching proteins in your input (IDs) | matching proteins in your input (labels) |
| Cluster25 | CL:22327 | Oxidative phosphorylation | 13 | 4.1007 | top of list | 7.14E-05 | afc | ENSP00000354499,ENSP00000354554,ENSP00000354632,ENSP00000354728,ENSP00000354813,ENSP00000354876,ENSP00000354961,ENSP00000354982,ENSP00000355046,ENSP00000355206,ENSP00000355265,ENSP00000418438,ENSP00000429690 | ATP6V0E1,MT-ATP6,MT-ATP8,MT-CO1,MT-CO2,MT-CO3,MT-CYB,MT-ND2,MT-ND3,MT-ND4,MT-ND4L,MT-ND5,NDUFA3 |
| Cluster25 | CL:22328 | respirasome | 11 | 4.5424 | top of list | 7.14E-05 | afc | ENSP00000354499,ENSP00000354554,ENSP00000354632,ENSP00000354728,ENSP00000354813,ENSP00000354876,ENSP00000354961,ENSP00000354982,ENSP00000355046,ENSP00000355206,ENSP00000418438 | MT-ATP6,MT-CO1,MT-CO2,MT-CO3,MT-CYB,MT-ND2,MT-ND3,MT-ND4,MT-ND4L,MT-ND5,NDUFA3 |
| Cluster25 | CL:4662 | Interferon alpha/beta signaling, and Interferon-induced protein 44 family | 20 | 4.20205 | bottom of list | 7.73E-05 | afc | ENSP00000252593,ENSP00000257570,ENSP00000306565,ENSP00000318982,ENSP00000333657,ENSP00000342513,ENSP00000345494,ENSP00000354822,ENSP00000359783,ENSP00000359787,ENSP00000360869,ENSP00000360883,ENSP00000368699,ENSP00000380697,ENSP00000381601,ENSP00000382707,ENSP00000386187,ENSP00000388001,ENSP00000395590,ENSP00000483430 | BST2,EPSTI1,IFI27,IFI35,IFI44,IFI44L,IFI6,IFIT1,IFIT3,IFITM1,IFITM3,IRF7,ISG15,ISG20,MX1,MX2,OAS1,OASL,PLSCR1,XAF1 |
| Cluster25 | CL:4665 | Interferon alpha/beta signaling | 18 | 4.38513 | bottom of list | 7.73E-05 | afc | ENSP00000252593,ENSP00000257570,ENSP00000306565,ENSP00000333657,ENSP00000342513,ENSP00000354822,ENSP00000359783,ENSP00000359787,ENSP00000360869,ENSP00000360883,ENSP00000368699,ENSP00000380697,ENSP00000381601,ENSP00000382707,ENSP00000386187,ENSP00000388001,ENSP00000395590,ENSP00000483430 | BST2,IFI27,IFI35,IFI44,IFI44L,IFI6,IFIT1,IFIT3,IFITM1,IFITM3,IRF7,ISG15,ISG20,MX1,MX2,OAS1,OASL,XAF1 |
| Cluster25 | CL:22446 | cytochrome complex, and Cytochrome c oxidase subunit VII | 6 | 5.22184 | top of list | 0.00083 | afc | ENSP00000354499,ENSP00000354554,ENSP00000354632,ENSP00000354876,ENSP00000354961,ENSP00000354982 | MT-ATP6,MT-CO1,MT-CO2,MT-CO3,MT-CYB,MT-ND4 |
| Cluster25 | CL:22502 | Leber hereditary optic neuropathy | 5 | 5.49062 | top of list | 0.0011 | afc | ENSP00000354499,ENSP00000354554,ENSP00000354632,ENSP00000354961,ENSP00000354982 | MT-ATP6,MT-CO1,MT-CO3,MT-CYB,MT-ND4 |
| Cluster25 | CL:4668 | type I interferon signaling pathway | 15 | 4.07637 | bottom of list | 0.0011 | afc | ENSP00000252593,ENSP00000257570,ENSP00000306565,ENSP00000333657,ENSP00000342513,ENSP00000354822,ENSP00000360869,ENSP00000360883,ENSP00000380697,ENSP00000381601,ENSP00000382707,ENSP00000386187,ENSP00000388001,ENSP00000395590,ENSP00000483430 | BST2,IFI27,IFI35,IFI6,IFIT1,IFIT3,IFITM1,IFITM3,IRF7,ISG20,MX1,MX2,OAS1,OASL,XAF1 |
| Cluster25 | CL:4670 | type I interferon signaling pathway | 14 | 3.99255 | bottom of list | 0.0014 | afc | ENSP00000257570,ENSP00000306565,ENSP00000333657,ENSP00000342513,ENSP00000354822,ENSP00000360869,ENSP00000360883,ENSP00000380697,ENSP00000381601,ENSP00000382707,ENSP00000386187,ENSP00000388001,ENSP00000395590,ENSP00000483430 | IFI27,IFI35,IFI6,IFIT1,IFIT3,IFITM1,IFITM3,IRF7,ISG20,MX1,MX2,OAS1,OASL,XAF1 |
| Cluster | #term ID | term description | genes mapped | enrichment score | direction | false discovery rate | method | matching proteins in your input (IDs) | matching proteins in your input (labels) |
| **Cluster** | **#term ID** | **term description** | **genes mapped** | **enrichment score** | **direction** | **false discovery rate** | **method** | **matching proteins in your input (IDs)** | **matching proteins in your input (labels)** |
| Cluster26 | CL:22328 | respirasome | 10 | 8.75224 | top of list | 3.90E-07 | afc | ENSP00000354499,ENSP00000354554,ENSP00000354632,ENSP00000354687,ENSP00000354728,ENSP00000354813,ENSP00000354876,ENSP00000354961,ENSP00000354982,ENSP00000355206 | MT-ATP6,MT-CO1,MT-CO2,MT-CO3,MT-CYB,MT-ND1,MT-ND3,MT-ND4,MT-ND4L,MT-ND5 |
| Cluster26 | CL:22327 | Oxidative phosphorylation | 11 | 8.70692 | top of list | 3.90E-07 | afc | ENSP00000354499,ENSP00000354554,ENSP00000354632,ENSP00000354687,ENSP00000354728,ENSP00000354813,ENSP00000354876,ENSP00000354961,ENSP00000354982,ENSP00000355206,ENSP00000355265 | MT-ATP6,MT-ATP8,MT-CO1,MT-CO2,MT-CO3,MT-CYB,MT-ND1,MT-ND3,MT-ND4,MT-ND4L,MT-ND5 |
| Cluster26 | CL:22502 | Leber hereditary optic neuropathy | 5 | 9.61865 | top of list | 1.56E-06 | afc | ENSP00000354499,ENSP00000354554,ENSP00000354632,ENSP00000354961,ENSP00000354982 | MT-ATP6,MT-CO1,MT-CO3,MT-CYB,MT-ND4 |
| Cluster26 | CL:22446 | cytochrome complex, and Cytochrome c oxidase subunit VII | 6 | 9.10772 | top of list | 1.56E-06 | afc | ENSP00000354499,ENSP00000354554,ENSP00000354632,ENSP00000354876,ENSP00000354961,ENSP00000354982 | MT-ATP6,MT-CO1,MT-CO2,MT-CO3,MT-CYB,MT-ND4 |
| Cluster26 | CL:4665 | Interferon alpha/beta signaling | 19 | 4.70092 | bottom of list | 1.72E-06 | afc | ENSP00000252593,ENSP00000257570,ENSP00000306565,ENSP00000333657,ENSP00000342513,ENSP00000354822,ENSP00000359783,ENSP00000359787,ENSP00000360869,ENSP00000360883,ENSP00000368699,ENSP00000371471,ENSP00000380697,ENSP00000381601,ENSP00000382707,ENSP00000386187,ENSP00000388001,ENSP00000395590,ENSP00000483430 | BST2,IFI27,IFI35,IFI44,IFI44L,IFI6,IFIT1,IFIT3,IFITM1,IFITM3,IRF7,ISG15,ISG20,MX1,MX2,OAS1,OASL,RSAD2,XAF1 |
| Cluster26 | CL:4668 | type I interferon signaling pathway | 16 | 4.4616 | bottom of list | 4.94E-05 | afc | ENSP00000252593,ENSP00000257570,ENSP00000306565,ENSP00000333657,ENSP00000342513,ENSP00000354822,ENSP00000360869,ENSP00000360883,ENSP00000371471,ENSP00000380697,ENSP00000381601,ENSP00000382707,ENSP00000386187,ENSP00000388001,ENSP00000395590,ENSP00000483430 | BST2,IFI27,IFI35,IFI6,IFIT1,IFIT3,IFITM1,IFITM3,IRF7,ISG20,MX1,MX2,OAS1,OASL,RSAD2,XAF1 |
| Cluster26 | CL:4670 | type I interferon signaling pathway | 15 | 4.48074 | bottom of list | 6.24E-05 | afc | ENSP00000257570,ENSP00000306565,ENSP00000333657,ENSP00000342513,ENSP00000354822,ENSP00000360869,ENSP00000360883,ENSP00000371471,ENSP00000380697,ENSP00000381601,ENSP00000382707,ENSP00000386187,ENSP00000388001,ENSP00000395590,ENSP00000483430 | IFI27,IFI35,IFI6,IFIT1,IFIT3,IFITM1,IFITM3,IRF7,ISG20,MX1,MX2,OAS1,OASL,RSAD2,XAF1 |
| Cluster26 | CL:4662 | Interferon alpha/beta signaling, and Interferon-induced protein 44 family | 21 | 4.53689 | bottom of list | 0.00031 | ks | ENSP00000252593,ENSP00000257570,ENSP00000306565,ENSP00000318982,ENSP00000333657,ENSP00000342513,ENSP00000345494,ENSP00000354822,ENSP00000359783,ENSP00000359787,ENSP00000360869,ENSP00000360883,ENSP00000368699,ENSP00000371471,ENSP00000380697,ENSP00000381601,ENSP00000382707,ENSP00000386187,ENSP00000388001,ENSP00000395590,ENSP00000483430 | BST2,EPSTI1,IFI27,IFI35,IFI44,IFI44L,IFI6,IFIT1,IFIT3,IFITM1,IFITM3,IRF7,ISG15,ISG20,MX1,MX2,OAS1,OASL,PLSCR1,RSAD2,XAF1 |
| Cluster26 | CL:4659 | Interferon alpha/beta signaling, and Interferon gamma signaling | 25 | 4.23063 | bottom of list | 0.00044 | ks | ENSP00000245185,ENSP00000252593,ENSP00000257570,ENSP00000258381,ENSP00000306565,ENSP00000318982,ENSP00000333657,ENSP00000342513,ENSP00000345494,ENSP00000354822,ENSP00000359504,ENSP00000359783,ENSP00000359787,ENSP00000360869,ENSP00000360883,ENSP00000368699,ENSP00000369299,ENSP00000371471,ENSP00000380697,ENSP00000381601,ENSP00000382707,ENSP00000386187,ENSP00000388001,ENSP00000395590,ENSP00000483430 | BST2,EPSTI1,GBP1,IFI27,IFI35,IFI44,IFI44L,IFI6,IFIT1,IFIT3,IFITM1,IFITM3,IRF7,ISG15,ISG20,MT2A,MX1,MX2,OAS1,OASL,PLSCR1,RSAD2,SP110,TRIM22,XAF1 |
| Cluster26 | CL:4661 | Interferon alpha/beta signaling, and Interferon gamma signaling | 24 | 4.31487 | bottom of list | 0.0005 | ks | ENSP00000245185,ENSP00000252593,ENSP00000257570,ENSP00000306565,ENSP00000318982,ENSP00000333657,ENSP00000342513,ENSP00000345494,ENSP00000354822,ENSP00000359504,ENSP00000359783,ENSP00000359787,ENSP00000360869,ENSP00000360883,ENSP00000368699,ENSP00000369299,ENSP00000371471,ENSP00000380697,ENSP00000381601,ENSP00000382707,ENSP00000386187,ENSP00000388001,ENSP00000395590,ENSP00000483430 | BST2,EPSTI1,GBP1,IFI27,IFI35,IFI44,IFI44L,IFI6,IFIT1,IFIT3,IFITM1,IFITM3,IRF7,ISG15,ISG20,MT2A,MX1,MX2,OAS1,OASL,PLSCR1,RSAD2,TRIM22,XAF1 |
| Cluster26 | CL:22332 | mitochondrial respiratory chain complex I | 4 | 8.21901 | top of list | 0.00071 | afc | ENSP00000354687,ENSP00000354728,ENSP00000354813,ENSP00000355206 | MT-ND1,MT-ND3,MT-ND4L,MT-ND5 |
| Cluster26 | CL:4673 | 2'-5'-oligoadenylate synthase, and type I interferon signaling pathway | 11 | 3.99648 | bottom of list | 0.0037 | afc | ENSP00000257570,ENSP00000333657,ENSP00000342513,ENSP00000354822,ENSP00000360869,ENSP00000360883,ENSP00000371471,ENSP00000380697,ENSP00000381601,ENSP00000388001,ENSP00000395590 | IFI35,IFI6,IFIT1,IFIT3,IRF7,MX1,MX2,OAS1,OASL,RSAD2,XAF1 |
| Cluster26 | CL:22408 | Leber hereditary optic neuropathy | 3 | 8.1344 | top of list | 0.0066 | afc | ENSP00000354687,ENSP00000354728,ENSP00000354813 | MT-ND1,MT-ND4L,MT-ND5 |
| Cluster | #term ID | term description | genes mapped | enrichment score | direction | false discovery rate | method | matching proteins in your input (IDs) | matching proteins in your input (labels) |
| Cluster27 | CL:4665 | Interferon alpha/beta signaling | 16 | 3.22143 | bottom of list | 0.00047 | afc | ENSP00000215794,ENSP00000252593,ENSP00000306565,ENSP00000326247,ENSP00000342513,ENSP00000354822,ENSP00000359787,ENSP00000368699,ENSP00000380697,ENSP00000381601,ENSP00000382707,ENSP00000386187,ENSP00000388001,ENSP00000395590,ENSP00000483430,ENSP00000484689 | BST2,IFI27,IFI35,IFI44L,IFI6,IFITM1,IFITM2,IFITM3,IRF7,ISG15,ISG20,MX1,OAS1,SAMD9L,USP18,XAF1 |
| Cluster27 | CL:4662 | Interferon alpha/beta signaling, and Interferon-induced protein 44 family | 18 | 2.83701 | bottom of list | 0.00072 | afc | ENSP00000215794,ENSP00000252593,ENSP00000306565,ENSP00000318982,ENSP00000326247,ENSP00000342513,ENSP00000345494,ENSP00000354822,ENSP00000359787,ENSP00000368699,ENSP00000380697,ENSP00000381601,ENSP00000382707,ENSP00000386187,ENSP00000388001,ENSP00000395590,ENSP00000483430,ENSP00000484689 | BST2,EPSTI1,IFI27,IFI35,IFI44L,IFI6,IFITM1,IFITM2,IFITM3,IRF7,ISG15,ISG20,MX1,OAS1,PLSCR1,SAMD9L,USP18,XAF1 |
| Cluster27 | CL:4668 | type I interferon signaling pathway | 12 | 3.34708 | bottom of list | 0.00089 | afc | ENSP00000252593,ENSP00000306565,ENSP00000342513,ENSP00000354822,ENSP00000380697,ENSP00000381601,ENSP00000382707,ENSP00000386187,ENSP00000388001,ENSP00000395590,ENSP00000483430,ENSP00000484689 | BST2,IFI27,IFI35,IFI6,IFITM1,IFITM2,IFITM3,IRF7,ISG20,MX1,OAS1,XAF1 |
| **Cluster** | **#term ID** | **term description** | **genes mapped** | **enrichment score** | **direction** | **false discovery rate** | **method** | **matching proteins in your input (IDs)** | **matching proteins in your input (labels)** |
| Cluster27 | CL:4661 | Interferon alpha/beta signaling, and Interferon gamma signaling | 20 | 2.53629 | bottom of list | 0.00089 | afc | ENSP00000215794,ENSP00000245185,ENSP00000252593,ENSP00000306565,ENSP00000318982,ENSP00000326247,ENSP00000342513,ENSP00000345494,ENSP00000354822,ENSP00000359787,ENSP00000368699,ENSP00000369299,ENSP00000380697,ENSP00000381601,ENSP00000382707,ENSP00000386187,ENSP00000388001,ENSP00000395590,ENSP00000483430,ENSP00000484689 | BST2,EPSTI1,IFI27,IFI35,IFI44L,IFI6,IFITM1,IFITM2,IFITM3,IRF7,ISG15,ISG20,MT2A,MX1,OAS1,PLSCR1,SAMD9L,TRIM22,USP18,XAF1 |
| Cluster27 | CL:22328 | respirasome | 7 | 5.68366 | top of list | 0.00098 | afc | ENSP00000317780,ENSP00000354554,ENSP00000354632,ENSP00000354728,ENSP00000354813,ENSP00000354961,ENSP00000355206 | COX5A,MT-ATP6,MT-CYB,MT-ND3,MT-ND4,MT-ND4L,MT-ND5 |
| Cluster27 | CL:4670 | type I interferon signaling pathway | 11 | 3.32228 | bottom of list | 0.0016 | afc | ENSP00000306565,ENSP00000342513,ENSP00000354822,ENSP00000380697,ENSP00000381601,ENSP00000382707,ENSP00000386187,ENSP00000388001,ENSP00000395590,ENSP00000483430,ENSP00000484689 | IFI27,IFI35,IFI6,IFITM1,IFITM2,IFITM3,IRF7,ISG20,MX1,OAS1,XAF1 |
| Cluster27 | CL:22327 | Oxidative phosphorylation | 8 | 5.02983 | top of list | 0.0019 | afc | ENSP00000317780,ENSP00000354554,ENSP00000354632,ENSP00000354728,ENSP00000354813,ENSP00000354961,ENSP00000355206,ENSP00000429690 | ATP6V0E1,COX5A,MT-ATP6,MT-CYB,MT-ND3,MT-ND4,MT-ND4L,MT-ND5 |
| Cluster | #term ID | term description | genes mapped | enrichment score | direction | false discovery rate | method | matching proteins in your input (IDs) | matching proteins in your input (labels) |
| Cluster28 | CL:22502 | Leber hereditary optic neuropathy | 5 | 6.80357 | top of list | 0.0029 | afc | ENSP00000354499,ENSP00000354554,ENSP00000354632,ENSP00000354961,ENSP00000354982 | MT-ATP6,MT-CO1,MT-CO3,MT-CYB,MT-ND4 |
| Cluster28 | CL:4661 | Interferon alpha/beta signaling, and Interferon gamma signaling | 14 | 3.5748 | bottom of list | 0.0038 | afc | ENSP00000245185,ENSP00000252593,ENSP00000306565,ENSP00000318982,ENSP00000342513,ENSP00000345494,ENSP00000359787,ENSP00000360883,ENSP00000368699,ENSP00000369299,ENSP00000381601,ENSP00000382707,ENSP00000386187,ENSP00000483430 | BST2,EPSTI1,IFI27,IFI44L,IFI6,IFIT3,IFITM1,IFITM3,ISG15,ISG20,MT2A,MX1,PLSCR1,TRIM22 |
| Cluster28 | CL:4659 | Interferon alpha/beta signaling, and Interferon gamma signaling | 15 | 3.40643 | bottom of list | 0.0041 | afc | ENSP00000245185,ENSP00000252593,ENSP00000258381,ENSP00000306565,ENSP00000318982,ENSP00000342513,ENSP00000345494,ENSP00000359787,ENSP00000360883,ENSP00000368699,ENSP00000369299,ENSP00000381601,ENSP00000382707,ENSP00000386187,ENSP00000483430 | BST2,EPSTI1,IFI27,IFI44L,IFI6,IFIT3,IFITM1,IFITM3,ISG15,ISG20,MT2A,MX1,PLSCR1,SP110,TRIM22 |
| Cluster28 | CL:4662 | Interferon alpha/beta signaling, and Interferon-induced protein 44 family | 12 | 3.6771 | bottom of list | 0.0064 | afc | ENSP00000252593,ENSP00000306565,ENSP00000318982,ENSP00000342513,ENSP00000345494,ENSP00000359787,ENSP00000360883,ENSP00000368699,ENSP00000381601,ENSP00000382707,ENSP00000386187,ENSP00000483430 | BST2,EPSTI1,IFI27,IFI44L,IFI6,IFIT3,IFITM1,IFITM3,ISG15,ISG20,MX1,PLSCR1 |

**Table S4 Compared with the normal controls, significantly enriched pathways of each cell subtypes in the moderate condition (patients 4-9). Cell type refers to Fig. 1d.**

| **Cluster** | **#term ID** | **term description** | **genes mapped** | **enrichment score** | **direction** | **false discovery rate** | **method** | **matching proteins in your input (IDs)** | **matching proteins in your input (labels)** |
| --- | --- | --- | --- | --- | --- | --- | --- | --- | --- |
| Cluster2 | CL:4665 | Interferon alpha/beta signaling | 6 | 7.47645 | bottom of list | 0.0035 | afc | ENSP00000342513,ENSP00000354822,ENSP00000359787,ENSP00000368699,ENSP00000381601,ENSP00000386187 | IFI44L,IFI6,IFITM1,ISG15,MX1,XAF1 |
| Cluster2 | CL:22328 | respirasome | 7 | 8.76009 | top of list | 0.0035 | afc | ENSP00000354554,ENSP00000354632,ENSP00000354728,ENSP00000354813,ENSP00000354961,ENSP00000354982,ENSP00000355206 | MT-ATP6,MT-CO3,MT-CYB,MT-ND3,MT-ND4,MT-ND4L,MT-ND5 |
| Cluster4 | CL:2481 | bZIP transcription factor, and Early growth response, N-terminal | 4 | 8.55642 | bottom of list | 0.008 | afc | ENSP00000303315,ENSP00000306245,ENSP00000360266,ENSP00000465617 | FOS,IER2,JUN,JUNB |
| Cluster4 | CL:2403 | mixed, incl. MAPK targets/ Nuclear events mediated by MAP kinases, and bZIP Maf transcription factor | 5 | 8.05948 | bottom of list | 0.008 | afc | ENSP00000239223,ENSP00000303315,ENSP00000306245,ENSP00000360266,ENSP00000465617 | DUSP1,FOS,IER2,JUN,JUNB |
| Cluster4 | CL:2482 | AP-1 transcription factor, and Transcription factor Jun | 3 | 9.21563 | bottom of list | 0.008 | afc | ENSP00000303315,ENSP00000306245,ENSP00000360266 | FOS,JUN,JUNB |
| Cluster5 | CL:4665 | Interferon alpha/beta signaling | 5 | 6.47793 | bottom of list | 0.00099 | afc | ENSP00000354822,ENSP00000359787,ENSP00000368699,ENSP00000380697,ENSP00000386187 | IFI44L,IFITM1,IRF7,ISG15,XAF1 |
| Cluster5 | CL:2403 | mixed, incl. MAPK targets/ Nuclear events mediated by MAP kinases, and bZIP Maf transcription factor | 5 | 5.65485 | bottom of list | 0.0031 | afc | ENSP00000239223,ENSP00000303315,ENSP00000306245,ENSP00000360266,ENSP00000465617 | DUSP1,FOS,IER2,JUN,JUNB |
| Cluster5 | CL:2481 | bZIP transcription factor, and Early growth response, N-terminal | 4 | 5.73729 | bottom of list | 0.0055 | afc | ENSP00000303315,ENSP00000306245,ENSP00000360266,ENSP00000465617 | FOS,IER2,JUN,JUNB |
| Cluster5 | CL:4670 | type I interferon signaling pathway | 3 | 6.91639 | bottom of list | 0.0055 | afc | ENSP00000354822,ENSP00000380697,ENSP00000386187 | IFITM1,IRF7,XAF1 |
| Cluster5 | CL:22328 | respirasome | 9 | 4.8909 | top of list | 0.0076 | afc | ENSP00000354499,ENSP00000354554,ENSP00000354632,ENSP00000354687,ENSP00000354728,ENSP00000354876,ENSP00000354961,ENSP00000354982,ENSP00000355206 | MT-ATP6,MT-CO1,MT-CO2,MT-CO3,MT-CYB,MT-ND1,MT-ND3,MT-ND4,MT-ND4L |
| Cluster6 | CL:4665 | Interferon alpha/beta signaling | 6 | 7.46031 | bottom of list | 0.0059 | afc | ENSP00000342513,ENSP00000354822,ENSP00000359787,ENSP00000368699,ENSP00000381601,ENSP00000386187 | IFI44L,IFI6,IFITM1,ISG15,MX1,XAF1 |
| Cluster7 | CL:4665 | Interferon alpha/beta signaling | 7 | 8.18748 | bottom of list | 0.005 | afc | ENSP00000252593,ENSP00000342513,ENSP00000354822,ENSP00000368699,ENSP00000381601,ENSP00000382707,ENSP00000386187 | BST2,IFI6,IFITM1,IFITM3,ISG15,MX1,XAF1 |
| Cluster8 | CL:4665 | Interferon alpha/beta signaling | 5 | 8.24765 | bottom of list | 0.00045 | afc | ENSP00000342513,ENSP00000359787,ENSP00000368699,ENSP00000386187,ENSP00000483430 | IFI27,IFI44L,IFI6,IFITM1,ISG15 |
| Cluster8 | CL:4670 | type I interferon signaling pathway | 3 | 8.47212 | bottom of list | 0.0078 | afc | ENSP00000342513,ENSP00000386187,ENSP00000483430 | IFI27,IFI6,IFITM1 |
| Cluster9 | CL:4665 | Interferon alpha/beta signaling | 8 | 8.50635 | bottom of list | 0.00012 | afc | ENSP00000252593,ENSP00000342513,ENSP00000354822,ENSP00000359787,ENSP00000368699,ENSP00000381601,ENSP00000382707,ENSP00000386187 | BST2,IFI44L,IFI6,IFITM1,IFITM3,ISG15,MX1,XAF1 |
| Cluster9 | CL:4668 | type I interferon signaling pathway | 6 | 8.26666 | bottom of list | 0.0017 | afc | ENSP00000252593,ENSP00000342513,ENSP00000354822,ENSP00000381601,ENSP00000382707,ENSP00000386187 | BST2,IFI6,IFITM1,IFITM3,MX1,XAF1 |
| Cluster9 | CL:4670 | type I interferon signaling pathway | 5 | 8.41225 | bottom of list | 0.006 | afc | ENSP00000342513,ENSP00000354822,ENSP00000381601,ENSP00000382707,ENSP00000386187 | IFI6,IFITM1,IFITM3,MX1,XAF1 |
| Cluster10 | CL:14967 | GTP hydrolysis and joining of the 60S ribosomal subunit, and Protein export | 20 | 7.57258 | top of list | 0.00063 | afc | ENSP00000296674,ENSP00000307889,ENSP00000307940,ENSP00000309334,ENSP00000311430,ENSP00000313007,ENSP00000339063,ENSP00000346001,ENSP00000346037,ENSP00000348849,ENSP00000359345,ENSP00000362744,ENSP00000369757,ENSP00000379888,ENSP00000388806,ENSP00000403172,ENSP00000404375,ENSP00000449328,ENSP00000463784,ENSP00000485663 | EEF1A1,EEF2,EIF3L,EIF4B,PABPC1,RPL13,RPL15,RPL26,RPL3,RPL36A,RPL4,RPL5,RPL6,RPLP0,RPLP1,RPS23,RPS26,RPS4X,RPS6,RPS8 |
| Cluster10 | CL:4662 | Interferon alpha/beta signaling, and Interferon-induced protein 44 family | 9 | 4.09039 | bottom of list | 0.00064 | afc | ENSP00000318982,ENSP00000342513,ENSP00000359787,ENSP00000368699,ENSP00000381601,ENSP00000382707,ENSP00000386187,ENSP00000483430,ENSP00000484689 | EPSTI1,IFI27,IFI44L,IFI6,IFITM1,IFITM2,IFITM3,ISG15,MX1 |
| Cluster10 | CL:4665 | Interferon alpha/beta signaling | 8 | 4.3616 | bottom of list | 0.00064 | afc | ENSP00000342513,ENSP00000359787,ENSP00000368699,ENSP00000381601,ENSP00000382707,ENSP00000386187,ENSP00000483430,ENSP00000484689 | IFI27,IFI44L,IFI6,IFITM1,IFITM2,IFITM3,ISG15,MX1 |
| Cluster10 | CL:14978 | Peptide chain elongation | 17 | 7.59529 | top of list | 0.00064 | afc | ENSP00000296674,ENSP00000307889,ENSP00000307940,ENSP00000309334,ENSP00000311430,ENSP00000339063,ENSP00000346001,ENSP00000346037,ENSP00000348849,ENSP00000359345,ENSP00000362744,ENSP00000369757,ENSP00000379888,ENSP00000403172,ENSP00000404375,ENSP00000449328,ENSP00000463784 | EEF1A1,EEF2,RPL13,RPL15,RPL26,RPL3,RPL36A,RPL4,RPL5,RPL6,RPLP0,RPLP1,RPS23,RPS26,RPS4X,RPS6,RPS8 |
| Cluster10 | CL:4661 | Interferon alpha/beta signaling, and Interferon gamma signaling | 10 | 3.88909 | bottom of list | 0.00064 | afc | ENSP00000245185,ENSP00000318982,ENSP00000342513,ENSP00000359787,ENSP00000368699,ENSP00000381601,ENSP00000382707,ENSP00000386187,ENSP00000483430,ENSP00000484689 | EPSTI1,IFI27,IFI44L,IFI6,IFITM1,IFITM2,IFITM3,ISG15,MT2A,MX1 |
| **Cluster** | **#term ID** | **term description** | **genes mapped** | **enrichment score** | **direction** | **false discovery rate** | **method** | **matching proteins in your input (IDs)** | **matching proteins in your input (labels)** |
| Cluster10 | CL:14982 | Peptide chain elongation | 15 | 7.55193 | top of list | 0.001 | afc | ENSP00000296674,ENSP00000307889,ENSP00000307940,ENSP00000309334,ENSP00000346001,ENSP00000346037,ENSP00000348849,ENSP00000359345,ENSP00000362744,ENSP00000369757,ENSP00000379888,ENSP00000403172,ENSP00000404375,ENSP00000449328,ENSP00000463784 | EEF2,RPL13,RPL15,RPL26,RPL3,RPL36A,RPL5,RPL6,RPLP0,RPLP1,RPS23,RPS26,RPS4X,RPS6,RPS8 |
| Cluster10 | CL:4670 | type I interferon signaling pathway | 6 | 4.89662 | bottom of list | 0.001 | afc | ENSP00000342513,ENSP00000381601,ENSP00000382707,ENSP00000386187,ENSP00000483430,ENSP00000484689 | IFI27,IFI6,IFITM1,IFITM2,IFITM3,MX1 |
| Cluster12 | CL:4665 | Interferon alpha/beta signaling | 9 | 5.19792 | bottom of list | 0.0014 | afc | ENSP00000342513,ENSP00000354822,ENSP00000359787,ENSP00000368699,ENSP00000381601,ENSP00000382707,ENSP00000386187,ENSP00000483430,ENSP00000484689 | IFI27,IFI44L,IFI6,IFITM1,IFITM2,IFITM3,ISG15,MX1,XAF1 |
| Cluster12 | CL:4662 | Interferon alpha/beta signaling, and Interferon-induced protein 44 family | 10 | 4.9182 | bottom of list | 0.0014 | afc | ENSP00000318982,ENSP00000342513,ENSP00000354822,ENSP00000359787,ENSP00000368699,ENSP00000381601,ENSP00000382707,ENSP00000386187,ENSP00000483430,ENSP00000484689 | EPSTI1,IFI27,IFI44L,IFI6,IFITM1,IFITM2,IFITM3,ISG15,MX1,XAF1 |
| Cluster12 | CL:14966 | GTP hydrolysis and joining of the 60S ribosomal subunit, and Protein export | 14 | 6.95477 | top of list | 0.0014 | afc | ENSP00000307889,ENSP00000307940,ENSP00000313007,ENSP00000339063,ENSP00000346001,ENSP00000346037,ENSP00000348849,ENSP00000369757,ENSP00000379888,ENSP00000388806,ENSP00000403172,ENSP00000449328,ENSP00000463784,ENSP00000477781 | EEF1A1,EEF2,EIF4B,PABPC1,RPL13,RPL26,RPL3,RPL6,RPLP0,RPLP1,RPS26,RPS6,RPS8,TPT1 |
| Cluster12 | CL:4661 | Interferon alpha/beta signaling, and Interferon gamma signaling | 11 | 4.86934 | bottom of list | 0.0014 | afc | ENSP00000245185,ENSP00000318982,ENSP00000342513,ENSP00000354822,ENSP00000359787,ENSP00000368699,ENSP00000381601,ENSP00000382707,ENSP00000386187,ENSP00000483430,ENSP00000484689 | EPSTI1,IFI27,IFI44L,IFI6,IFITM1,IFITM2,IFITM3,ISG15,MT2A,MX1,XAF1 |
| Cluster12 | CL:4670 | type I interferon signaling pathway | 7 | 5.60074 | bottom of list | 0.0016 | afc | ENSP00000342513,ENSP00000354822,ENSP00000381601,ENSP00000382707,ENSP00000386187,ENSP00000483430,ENSP00000484689 | IFI27,IFI6,IFITM1,IFITM2,IFITM3,MX1,XAF1 |
| Cluster12 | CL:14967 | GTP hydrolysis and joining of the 60S ribosomal subunit, and Protein export | 13 | 6.79592 | top of list | 0.0016 | afc | ENSP00000307889,ENSP00000307940,ENSP00000313007,ENSP00000339063,ENSP00000346001,ENSP00000346037,ENSP00000348849,ENSP00000369757,ENSP00000379888,ENSP00000388806,ENSP00000403172,ENSP00000449328,ENSP00000463784 | EEF1A1,EEF2,EIF4B,PABPC1,RPL13,RPL26,RPL3,RPL6,RPLP0,RPLP1,RPS26,RPS6,RPS8 |
| Cluster14 | CL:4665 | Interferon alpha/beta signaling | 5 | 8.57065 | bottom of list | 0.0029 | afc | ENSP00000342513,ENSP00000354822,ENSP00000359787,ENSP00000368699,ENSP00000386187 | IFI44L,IFI6,IFITM1,ISG15,XAF1 |
| Cluster15 | CL:22328 | respirasome | 7 | 7.48625 | top of list | 0.0079 | afc | ENSP00000354554,ENSP00000354632,ENSP00000354728,ENSP00000354813,ENSP00000354961,ENSP00000354982,ENSP00000355206 | MT-ATP6,MT-CO3,MT-CYB,MT-ND3,MT-ND4,MT-ND4L,MT-ND5 |
| Cluster16 | CL:14967 | GTP hydrolysis and joining of the 60S ribosomal subunit, and Protein export | 42 | 6.16213 | top of list | 6.07E-05 | ks | ENSP00000225430,ENSP00000230050,ENSP00000251453,ENSP00000272317,ENSP00000278572,ENSP00000296674,ENSP00000307889,ENSP00000318646,ENSP00000322419,ENSP00000339063,ENSP00000339095,ENSP00000345957,ENSP00000346001,ENSP00000346027,ENSP00000346037,ENSP00000346050,ENSP00000348849,ENSP00000354739,ENSP00000355315,ENSP00000357555,ENSP00000361076,ENSP00000362744,ENSP00000363018,ENSP00000363676,ENSP00000369757,ENSP00000375730,ENSP00000378163,ENSP00000379506,ENSP00000379888,ENSP00000385958,ENSP00000388806,ENSP00000389103,ENSP00000393241,ENSP00000400467,ENSP00000403172,ENSP00000404375,ENSP00000413436,ENSP00000419449,ENSP00000428085,ENSP00000447001,ENSP00000463784,ENSP00000472469 | EEF1A1,EIF1,EIF4B,RPL10,RPL10A,RPL11,RPL12,RPL13,RPL13A,RPL14,RPL18,RPL19,RPL21,RPL23A,RPL26,RPL3,RPL30,RPL34,RPL36A,RPL39,RPL6,RPL7A,RPL9,RPLP1,RPLP2,RPS12,RPS14,RPS15A,RPS16,RPS18,RPS21,RPS23,RPS26,RPS27,RPS27A,RPS28,RPS3,RPS3A,RPS4X,RPS6,RPS7,RPS8 |
| Cluster16 | CL:14966 | GTP hydrolysis and joining of the 60S ribosomal subunit, and Protein export | 44 | 6.14343 | top of list | 6.07E-05 | ks | ENSP00000225430,ENSP00000230050,ENSP00000251453,ENSP00000272317,ENSP00000278572,ENSP00000296674,ENSP00000307889,ENSP00000318646,ENSP00000322419,ENSP00000339063,ENSP00000339095,ENSP00000345957,ENSP00000346001,ENSP00000346027,ENSP00000346037,ENSP00000346050,ENSP00000348849,ENSP00000354739,ENSP00000355315,ENSP00000357555,ENSP00000361076,ENSP00000362744,ENSP00000363018,ENSP00000363676,ENSP00000369757,ENSP00000375730,ENSP00000376056,ENSP00000378163,ENSP00000379506,ENSP00000379888,ENSP00000385958,ENSP00000388806,ENSP00000389103,ENSP00000393241,ENSP00000400467,ENSP00000403172,ENSP00000404375,ENSP00000413436,ENSP00000419449,ENSP00000428085,ENSP00000447001,ENSP00000463784,ENSP00000472469,ENSP00000477781 | EEF1A1,EEF1B2,EIF1,EIF4B,RPL10,RPL10A,RPL11,RPL12,RPL13,RPL13A,RPL14,RPL18,RPL19,RPL21,RPL23A,RPL26,RPL3,RPL30,RPL34,RPL36A,RPL39,RPL6,RPL7A,RPL9,RPLP1,RPLP2,RPS12,RPS14,RPS15A,RPS16,RPS18,RPS21,RPS23,RPS26,RPS27,RPS27A,RPS28,RPS3,RPS3A,RPS4X,RPS6,RPS7,RPS8,TPT1 |
| Cluster16 | CL:14978 | Peptide chain elongation | 38 | 6.16047 | top of list | 8.27E-05 | ks | ENSP00000225430,ENSP00000230050,ENSP00000251453,ENSP00000272317,ENSP00000296674,ENSP00000307889,ENSP00000318646,ENSP00000322419,ENSP00000339063,ENSP00000339095,ENSP00000345957,ENSP00000346001,ENSP00000346027,ENSP00000346037,ENSP00000346050,ENSP00000348849,ENSP00000354739,ENSP00000355315,ENSP00000357555,ENSP00000361076,ENSP00000362744,ENSP00000363018,ENSP00000363676,ENSP00000369757,ENSP00000375730,ENSP00000378163,ENSP00000379506,ENSP00000379888,ENSP00000385958,ENSP00000389103,ENSP00000393241,ENSP00000400467,ENSP00000403172,ENSP00000404375,ENSP00000413436,ENSP00000447001,ENSP00000463784,ENSP00000472469 | EEF1A1,RPL10,RPL10A,RPL11,RPL12,RPL13,RPL13A,RPL14,RPL18,RPL19,RPL21,RPL23A,RPL26,RPL3,RPL34,RPL36A,RPL39,RPL6,RPL7A,RPL9,RPLP1,RPLP2,RPS12,RPS14,RPS15A,RPS16,RPS18,RPS21,RPS23,RPS26,RPS27,RPS27A,RPS28,RPS3A,RPS4X,RPS6,RPS7,RPS8 |
| Cluster16 | CL:14976 | Peptide chain elongation | 39 | 6.15331 | top of list | 8.27E-05 | ks | ENSP00000225430,ENSP00000230050,ENSP00000251453,ENSP00000272317,ENSP00000296674,ENSP00000307889,ENSP00000318646,ENSP00000322419,ENSP00000339063,ENSP00000339095,ENSP00000345957,ENSP00000346001,ENSP00000346027,ENSP00000346037,ENSP00000346050,ENSP00000348849,ENSP00000354739,ENSP00000355315,ENSP00000357555,ENSP00000361076,ENSP00000362744,ENSP00000363018,ENSP00000363676,ENSP00000369757,ENSP00000375730,ENSP00000378163,ENSP00000379506,ENSP00000379888,ENSP00000385958,ENSP00000389103,ENSP00000393241,ENSP00000400467,ENSP00000403172,ENSP00000404375,ENSP00000413436,ENSP00000428085,ENSP00000447001,ENSP00000463784,ENSP00000472469 | EEF1A1,RPL10,RPL10A,RPL11,RPL12,RPL13,RPL13A,RPL14,RPL18,RPL19,RPL21,RPL23A,RPL26,RPL3,RPL30,RPL34,RPL36A,RPL39,RPL6,RPL7A,RPL9,RPLP1,RPLP2,RPS12,RPS14,RPS15A,RPS16,RPS18,RPS21,RPS23,RPS26,RPS27,RPS27A,RPS28,RPS3A,RPS4X,RPS6,RPS7,RPS8 |
| Cluster16 | CL:14980 | Peptide chain elongation | 36 | 6.15741 | top of list | 0.00011 | ks | ENSP00000225430,ENSP00000230050,ENSP00000251453,ENSP00000272317,ENSP00000296674,ENSP00000307889,ENSP00000318646,ENSP00000322419,ENSP00000339095,ENSP00000345957,ENSP00000346001,ENSP00000346027,ENSP00000346037,ENSP00000346050,ENSP00000348849,ENSP00000354739,ENSP00000355315,ENSP00000357555,ENSP00000361076,ENSP00000362744,ENSP00000363018,ENSP00000363676,ENSP00000369757,ENSP00000375730,ENSP00000379506,ENSP00000379888,ENSP00000385958,ENSP00000389103,ENSP00000393241,ENSP00000400467,ENSP00000403172,ENSP00000404375,ENSP00000413436,ENSP00000447001,ENSP00000463784,ENSP00000472469 | RPL10,RPL10A,RPL11,RPL12,RPL13,RPL13A,RPL14,RPL18,RPL19,RPL21,RPL23A,RPL26,RPL3,RPL36A,RPL39,RPL6,RPL7A,RPL9,RPLP1,RPLP2,RPS12,RPS14,RPS15A,RPS16,RPS18,RPS21,RPS23,RPS26,RPS27,RPS27A,RPS28,RPS3A,RPS4X,RPS6,RPS7,RPS8 |
| Cluster16 | CL:14982 | Peptide chain elongation | 35 | 6.17049 | top of list | 0.00012 | ks | ENSP00000225430,ENSP00000230050,ENSP00000251453,ENSP00000272317,ENSP00000296674,ENSP00000307889,ENSP00000318646,ENSP00000322419,ENSP00000339095,ENSP00000345957,ENSP00000346001,ENSP00000346027,ENSP00000346037,ENSP00000346050,ENSP00000348849,ENSP00000354739,ENSP00000355315,ENSP00000357555,ENSP00000361076,ENSP00000362744,ENSP00000363018,ENSP00000363676,ENSP00000369757,ENSP00000375730,ENSP00000379506,ENSP00000379888,ENSP00000385958,ENSP00000389103,ENSP00000393241,ENSP00000400467,ENSP00000403172,ENSP00000404375,ENSP00000413436,ENSP00000463784,ENSP00000472469 | RPL10,RPL10A,RPL11,RPL12,RPL13,RPL13A,RPL14,RPL19,RPL21,RPL23A,RPL26,RPL3,RPL36A,RPL39,RPL6,RPL7A,RPL9,RPLP1,RPLP2,RPS12,RPS14,RPS15A,RPS16,RPS18,RPS21,RPS23,RPS26,RPS27,RPS27A,RPS28,RPS3A,RPS4X,RPS6,RPS7,RPS8 |
| Cluster16 | CL:14985 | Viral mRNA Translation | 30 | 6.27109 | top of list | 0.00022 | ks | ENSP00000225430,ENSP00000230050,ENSP00000251453,ENSP00000272317,ENSP00000296674,ENSP00000307889,ENSP00000318646,ENSP00000339095,ENSP00000345957,ENSP00000346001,ENSP00000346027,ENSP00000346050,ENSP00000348849,ENSP00000354739,ENSP00000355315,ENSP00000361076,ENSP00000362744,ENSP00000363018,ENSP00000363676,ENSP00000369757,ENSP00000375730,ENSP00000379506,ENSP00000379888,ENSP00000385958,ENSP00000389103,ENSP00000393241,ENSP00000400467,ENSP00000403172,ENSP00000463784,ENSP00000472469 | RPL10A,RPL11,RPL12,RPL13,RPL13A,RPL14,RPL19,RPL21,RPL23A,RPL26,RPL3,RPL39,RPL6,RPL7A,RPL9,RPS12,RPS14,RPS15A,RPS16,RPS18,RPS21,RPS23,RPS26,RPS27A,RPS28,RPS3A,RPS4X,RPS6,RPS7,RPS8 |
| **Cluster** | **#term ID** | **term description** | **genes mapped** | **enrichment score** | **direction** | **false discovery rate** | **method** | **matching proteins in your input (IDs)** | **matching proteins in your input (labels)** |
| Cluster16 | CL:10246 | specific granule lumen, and azurophil granule lumen | 11 | 4.08753 | bottom of list | 0.00022 | afc | ENSP00000053867,ENSP00000219022,ENSP00000221515,ENSP00000231751,ENSP00000236826,ENSP00000261267,ENSP00000296435,ENSP00000357123,ENSP00000357981,ENSP00000362108,ENSP00000381448 | CAMP,CST3,CTSS,GRN,LCN2,LTF,LYZ,MMP8,MNDA,OLFM4,RETN |
| Cluster16 | CL:14983 | Peptide chain elongation | 32 | 6.21925 | top of list | 0.00022 | ks | ENSP00000225430,ENSP00000230050,ENSP00000251453,ENSP00000272317,ENSP00000296674,ENSP00000307889,ENSP00000318646,ENSP00000322419,ENSP00000339095,ENSP00000345957,ENSP00000346001,ENSP00000346027,ENSP00000346050,ENSP00000348849,ENSP00000354739,ENSP00000355315,ENSP00000361076,ENSP00000362744,ENSP00000363018,ENSP00000363676,ENSP00000369757,ENSP00000375730,ENSP00000379506,ENSP00000379888,ENSP00000385958,ENSP00000389103,ENSP00000393241,ENSP00000400467,ENSP00000403172,ENSP00000404375,ENSP00000463784,ENSP00000472469 | RPL10A,RPL11,RPL12,RPL13,RPL13A,RPL14,RPL19,RPL21,RPL23A,RPL26,RPL3,RPL36A,RPL39,RPL6,RPL7A,RPL9,RPLP2,RPS12,RPS14,RPS15A,RPS16,RPS18,RPS21,RPS23,RPS26,RPS27A,RPS28,RPS3A,RPS4X,RPS6,RPS7,RPS8 |
| Cluster16 | CL:10247 | specific granule lumen, and Vitamin B6 metabolism | 8 | 4.76529 | bottom of list | 0.00037 | afc | ENSP00000219022,ENSP00000231751,ENSP00000236826,ENSP00000261267,ENSP00000296435,ENSP00000357981,ENSP00000362108,ENSP00000381448 | CAMP,CST3,CTSS,LCN2,LTF,LYZ,MMP8,OLFM4 |
| Cluster16 | CL:4615 | neutrophil aggregation, and Aquaporin 9 | 3 | 7.48724 | bottom of list | 0.00056 | afc | ENSP00000357722,ENSP00000357726,ENSP00000357727 | S100A12,S100A8,S100A9 |
| Cluster16 | CL:4560 | immunoglobulin binding, and Immunoregulatory interactions between a Lymphoid and a non-Lymphoid cell | 5 | 5.6828 | bottom of list | 0.00081 | afc | ENSP00000262629,ENSP00000289902,ENSP00000357722,ENSP00000357726,ENSP00000357727 | FCER1G,S100A12,S100A8,S100A9,TYROBP |
| Cluster16 | CL:4594 | mixed, incl. lipoteichoic acid binding, and neutrophil aggregation | 4 | 6.21442 | bottom of list | 0.0011 | afc | ENSP00000262629,ENSP00000357722,ENSP00000357726,ENSP00000357727 | S100A12,S100A8,S100A9,TYROBP |
| Cluster16 | CL:22328 | respirasome | 8 | 6.73975 | top of list | 0.0027 | afc | ENSP00000354554,ENSP00000354632,ENSP00000354687,ENSP00000354728,ENSP00000354813,ENSP00000354961,ENSP00000354982,ENSP00000355046 | MT-ATP6,MT-CO3,MT-CYB,MT-ND1,MT-ND2,MT-ND4,MT-ND4L,MT-ND5 |
| Cluster16 | CL:10251 | specific granule lumen | 7 | 4.01747 | bottom of list | 0.0033 | afc | ENSP00000219022,ENSP00000231751,ENSP00000236826,ENSP00000261267,ENSP00000296435,ENSP00000357981,ENSP00000381448 | CAMP,CST3,CTSS,LTF,LYZ,MMP8,OLFM4 |
| Cluster19 | CL:4665 | Interferon alpha/beta signaling | 11 | 5.50081 | bottom of list | 5.60E-07 | afc | ENSP00000245414,ENSP00000342513,ENSP00000354822,ENSP00000359787,ENSP00000360883,ENSP00000368699,ENSP00000380697,ENSP00000381601,ENSP00000382707,ENSP00000386187,ENSP00000483430 | IFI27,IFI44L,IFI6,IFIT3,IFITM1,IFITM3,IRF1,IRF7,ISG15,MX1,XAF1 |
| Cluster19 | CL:4670 | type I interferon signaling pathway | 8 | 6.19195 | bottom of list | 1.12E-06 | afc | ENSP00000342513,ENSP00000354822,ENSP00000360883,ENSP00000380697,ENSP00000381601,ENSP00000382707,ENSP00000386187,ENSP00000483430 | IFI27,IFI6,IFIT3,IFITM1,IFITM3,IRF7,MX1,XAF1 |
| Cluster19 | CL:4668 | type I interferon signaling pathway | 9 | 5.39779 | bottom of list | 2.61E-06 | afc | ENSP00000245414,ENSP00000342513,ENSP00000354822,ENSP00000360883,ENSP00000380697,ENSP00000381601,ENSP00000382707,ENSP00000386187,ENSP00000483430 | IFI27,IFI6,IFIT3,IFITM1,IFITM3,IRF1,IRF7,MX1,XAF1 |
| Cluster19 | CL:22328 | respirasome | 10 | 6.46419 | top of list | 8.68E-06 | afc | ENSP00000354499,ENSP00000354554,ENSP00000354632,ENSP00000354687,ENSP00000354728,ENSP00000354813,ENSP00000354876,ENSP00000354961,ENSP00000354982,ENSP00000355206 | MT-ATP6,MT-CO1,MT-CO2,MT-CO3,MT-CYB,MT-ND1,MT-ND3,MT-ND4,MT-ND4L,MT-ND5 |
| Cluster19 | CL:4662 | Interferon alpha/beta signaling, and Interferon-induced protein 44 family | 8 | 5.62312 | bottom of list | 6.50E-06 | afc | ENSP00000318982,ENSP00000354822,ENSP00000359787,ENSP00000368699,ENSP00000380697,ENSP00000382707,ENSP00000386187,ENSP00000483430 | EPSTI1,IFI27,IFI44L,IFITM1,IFITM3,IRF7,ISG15,XAF1 |
| Cluster19 | CL:4665 | Interferon alpha/beta signaling | 7 | 5.79217 | bottom of list | 9.25E-06 | afc | ENSP00000354822,ENSP00000359787,ENSP00000368699,ENSP00000380697,ENSP00000382707,ENSP00000386187,ENSP00000483430 | IFI27,IFI44L,IFITM1,IFITM3,IRF7,ISG15,XAF1 |
| Cluster19 | CL:22327 | Oxidative phosphorylation | 11 | 6.12333 | top of list | 1.34E-05 | afc | ENSP00000354499,ENSP00000354554,ENSP00000354632,ENSP00000354687,ENSP00000354728,ENSP00000354813,ENSP00000354876,ENSP00000354961,ENSP00000354982,ENSP00000355206,ENSP00000355265 | MT-ATP6,MT-ATP8,MT-CO1,MT-CO2,MT-CO3,MT-CYB,MT-ND1,MT-ND3,MT-ND4,MT-ND4L,MT-ND5 |
| Cluster19 | CL:4665 | Interferon alpha/beta signaling | 7 | 7.83161 | bottom of list | 2.27E-05 | afc | ENSP00000354822,ENSP00000359787,ENSP00000360883,ENSP00000368699,ENSP00000380697,ENSP00000381601,ENSP00000386187 | IFI44L,IFIT3,IFITM1,IRF7,ISG15,MX1,XAF1 |
| Cluster19 | CL:22502 | Leber hereditary optic neuropathy | 5 | 6.68353 | top of list | 0.0016 | afc | ENSP00000354499,ENSP00000354554,ENSP00000354632,ENSP00000354961,ENSP00000354982 | MT-ATP6,MT-CO1,MT-CO3,MT-CYB,MT-ND4 |
| Cluster19 | CL:4673 | 2'-5'-oligoadenylate synthase, and type I interferon signaling pathway | 5 | 5.04292 | bottom of list | 0.0017 | afc | ENSP00000342513,ENSP00000354822,ENSP00000360883,ENSP00000380697,ENSP00000381601 | IFI6,IFIT3,IRF7,MX1,XAF1 |
| Cluster19 | CL:22446 | cytochrome complex, and Cytochrome c oxidase subunit VII | 6 | 6.09916 | top of list | 0.0017 | afc | ENSP00000354499,ENSP00000354554,ENSP00000354632,ENSP00000354876,ENSP00000354961,ENSP00000354982 | MT-ATP6,MT-CO1,MT-CO2,MT-CO3,MT-CYB,MT-ND4 |
| Cluster19 | CL:22332 | mitochondrial respiratory chain complex I | 4 | 7.01173 | top of list | 0.002 | afc | ENSP00000354687,ENSP00000354728,ENSP00000354813,ENSP00000355206 | MT-ND1,MT-ND3,MT-ND4L,MT-ND5 |
| Cluster19 | CL:4670 | type I interferon signaling pathway | 5 | 7.42609 | bottom of list | 0.0011 | afc | ENSP00000354822,ENSP00000360883,ENSP00000380697,ENSP00000381601,ENSP00000386187 | IFIT3,IFITM1,IRF7,MX1,XAF1 |
| Cluster19 | CL:4673 | 2'-5'-oligoadenylate synthase, and type I interferon signaling pathway | 4 | 7.30274 | bottom of list | 0.0058 | afc | ENSP00000354822,ENSP00000360883,ENSP00000380697,ENSP00000381601 | IFIT3,IRF7,MX1,XAF1 |
| Cluster19 | CL:2403 | mixed, incl. MAPK targets/ Nuclear events mediated by MAP kinases, and bZIP Maf transcription factor | 5 | 8.14799 | bottom of list | 0.0011 | afc | ENSP00000239223,ENSP00000303315,ENSP00000306245,ENSP00000360266,ENSP00000465617 | DUSP1,FOS,IER2,JUN,JUNB |
| **Cluster** | **#term ID** | **term description** | **genes mapped** | **enrichment score** | **direction** | **false discovery rate** | **method** | **matching proteins in your input (IDs)** | **matching proteins in your input (labels)** |
| Cluster19 | CL:2481 | bZIP transcription factor, and Early growth response, N-terminal | 4 | 8.16723 | bottom of list | 0.0024 | afc | ENSP00000303315,ENSP00000306245,ENSP00000360266,ENSP00000465617 | FOS,IER2,JUN,JUNB |
| Cluster19 | CL:22328 | respirasome | 9 | 6.07556 | top of list | 0.0024 | afc | ENSP00000354499,ENSP00000354554,ENSP00000354632,ENSP00000354687,ENSP00000354728,ENSP00000354876,ENSP00000354961,ENSP00000354982,ENSP00000355206 | MT-ATP6,MT-CO1,MT-CO2,MT-CO3,MT-CYB,MT-ND1,MT-ND3,MT-ND4,MT-ND4L |
| Cluster19 | CL:22327 | Oxidative phosphorylation | 10 | 5.69082 | top of list | 0.0032 | afc | ENSP00000354499,ENSP00000354554,ENSP00000354632,ENSP00000354687,ENSP00000354728,ENSP00000354876,ENSP00000354961,ENSP00000354982,ENSP00000355206,ENSP00000355265 | MT-ATP6,MT-ATP8,MT-CO1,MT-CO2,MT-CO3,MT-CYB,MT-ND1,MT-ND3,MT-ND4,MT-ND4L |
| Cluster19 | CL:4665 | Interferon alpha/beta signaling | 6 | 6.82242 | bottom of list | 0.0032 | afc | ENSP00000354822,ENSP00000359787,ENSP00000368699,ENSP00000382707,ENSP00000386187,ENSP00000483430 | IFI27,IFI44L,IFITM1,IFITM3,ISG15,XAF1 |
| Cluster19 | CL:2482 | AP-1 transcription factor, and Transcription factor Jun | 3 | 8.39416 | bottom of list | 0.0048 | afc | ENSP00000303315,ENSP00000306245,ENSP00000360266 | FOS,JUN,JUNB |
| Cluster19 | CL:4670 | type I interferon signaling pathway | 5 | 5.86887 | bottom of list | 0.0004 | afc | ENSP00000354822,ENSP00000380697,ENSP00000382707,ENSP00000386187,ENSP00000483430 | IFI27,IFITM1,IFITM3,IRF7,XAF1 |
| Cluster20 | CL:22327 | Oxidative phosphorylation | 12 | 4.98623 | top of list | 0.00023 | afc | ENSP00000354499,ENSP00000354554,ENSP00000354632,ENSP00000354687,ENSP00000354728,ENSP00000354813,ENSP00000354876,ENSP00000354961,ENSP00000354982,ENSP00000355046,ENSP00000355206,ENSP00000355265 | MT-ATP6,MT-ATP8,MT-CO1,MT-CO2,MT-CO3,MT-CYB,MT-ND1,MT-ND2,MT-ND3,MT-ND4,MT-ND4L,MT-ND5 |
| Cluster20 | CL:22328 | respirasome | 11 | 5.16716 | top of list | 0.00023 | afc | ENSP00000354499,ENSP00000354554,ENSP00000354632,ENSP00000354687,ENSP00000354728,ENSP00000354813,ENSP00000354876,ENSP00000354961,ENSP00000354982,ENSP00000355046,ENSP00000355206 | MT-ATP6,MT-CO1,MT-CO2,MT-CO3,MT-CYB,MT-ND1,MT-ND2,MT-ND3,MT-ND4,MT-ND4L,MT-ND5 |
| Cluster20 | CL:22446 | cytochrome complex, and Cytochrome c oxidase subunit VII | 6 | 5.48518 | top of list | 0.0016 | afc | ENSP00000354499,ENSP00000354554,ENSP00000354632,ENSP00000354876,ENSP00000354961,ENSP00000354982 | MT-ATP6,MT-CO1,MT-CO2,MT-CO3,MT-CYB,MT-ND4 |
| Cluster20 | CL:4670 | type I interferon signaling pathway | 7 | 3.9383 | bottom of list | 0.0016 | afc | ENSP00000342513,ENSP00000354822,ENSP00000360883,ENSP00000381601,ENSP00000382707,ENSP00000386187,ENSP00000483430 | IFI27,IFI6,IFIT3,IFITM1,IFITM3,MX1,XAF1 |
| Cluster20 | CL:4665 | Interferon alpha/beta signaling | 9 | 3.69486 | bottom of list | 0.0016 | afc | ENSP00000342513,ENSP00000354822,ENSP00000359787,ENSP00000360883,ENSP00000368699,ENSP00000381601,ENSP00000382707,ENSP00000386187,ENSP00000483430 | IFI27,IFI44L,IFI6,IFIT3,IFITM1,IFITM3,ISG15,MX1,XAF1 |
| Cluster20 | CL:4661 | Interferon alpha/beta signaling, and Interferon gamma signaling | 12 | 3.24658 | bottom of list | 0.0016 | afc | ENSP00000245185,ENSP00000318982,ENSP00000342513,ENSP00000345494,ENSP00000354822,ENSP00000359787,ENSP00000360883,ENSP00000368699,ENSP00000381601,ENSP00000382707,ENSP00000386187,ENSP00000483430 | EPSTI1,IFI27,IFI44L,IFI6,IFIT3,IFITM1,IFITM3,ISG15,MT2A,MX1,PLSCR1,XAF1 |
| Cluster20 | CL:4662 | Interferon alpha/beta signaling, and Interferon-induced protein 44 family | 11 | 3.34256 | bottom of list | 0.0016 | afc | ENSP00000318982,ENSP00000342513,ENSP00000345494,ENSP00000354822,ENSP00000359787,ENSP00000360883,ENSP00000368699,ENSP00000381601,ENSP00000382707,ENSP00000386187,ENSP00000483430 | EPSTI1,IFI27,IFI44L,IFI6,IFIT3,IFITM1,IFITM3,ISG15,MX1,PLSCR1,XAF1 |
| Cluster20 | CL:22502 | Leber hereditary optic neuropathy | 5 | 5.63088 | top of list | 0.0042 | afc | ENSP00000354499,ENSP00000354554,ENSP00000354632,ENSP00000354961,ENSP00000354982 | MT-ATP6,MT-CO1,MT-CO3,MT-CYB,MT-ND4 |
| Cluster21 | CL:14967 | GTP hydrolysis and joining of the 60S ribosomal subunit, and Protein export | 9 | 8.60485 | top of list | 0.0024 | afc | ENSP00000307889,ENSP00000307940,ENSP00000313007,ENSP00000346001,ENSP00000388806,ENSP00000404375,ENSP00000449328,ENSP00000463784,ENSP00000485663 | EEF2,EIF3L,EIF4B,PABPC1,RPL13,RPL26,RPL3,RPL36A,RPLP0 |
| Cluster21 | CL:4670 | type I interferon signaling pathway | 6 | 5.09968 | bottom of list | 0.0024 | afc | ENSP00000342513,ENSP00000381601,ENSP00000382707,ENSP00000386187,ENSP00000483430,ENSP00000484689 | IFI27,IFI6,IFITM1,IFITM2,IFITM3,MX1 |
| Cluster21 | CL:4662 | Interferon alpha/beta signaling, and Interferon-induced protein 44 family | 9 | 4.13164 | bottom of list | 0.0024 | afc | ENSP00000318982,ENSP00000342513,ENSP00000359787,ENSP00000368699,ENSP00000381601,ENSP00000382707,ENSP00000386187,ENSP00000483430,ENSP00000484689 | EPSTI1,IFI27,IFI44L,IFI6,IFITM1,IFITM2,IFITM3,ISG15,MX1 |
| Cluster21 | CL:4665 | Interferon alpha/beta signaling | 8 | 4.47648 | bottom of list | 0.0024 | afc | ENSP00000342513,ENSP00000359787,ENSP00000368699,ENSP00000381601,ENSP00000382707,ENSP00000386187,ENSP00000483430,ENSP00000484689 | IFI27,IFI44L,IFI6,IFITM1,IFITM2,IFITM3,ISG15,MX1 |
| Cluster21 | CL:14966 | GTP hydrolysis and joining of the 60S ribosomal subunit, and Protein export | 11 | 8.52649 | top of list | 0.0024 | afc | ENSP00000307889,ENSP00000307940,ENSP00000313007,ENSP00000346001,ENSP00000376056,ENSP00000388806,ENSP00000404375,ENSP00000449328,ENSP00000463784,ENSP00000477781,ENSP00000485663 | EEF1B2,EEF2,EIF3L,EIF4B,PABPC1,RPL13,RPL26,RPL3,RPL36A,RPLP0,TPT1 |
| Cluster22 | CL:4665 | Interferon alpha/beta signaling | 4 | 6.72806 | bottom of list | 0.0022 | afc | ENSP00000342513,ENSP00000368699,ENSP00000386187,ENSP00000483430 | IFI27,IFI6,IFITM1,ISG15 |
| Cluster22 | CL:4670 | type I interferon signaling pathway | 3 | 6.83743 | bottom of list | 0.0093 | afc | ENSP00000342513,ENSP00000386187,ENSP00000483430 | IFI27,IFI6,IFITM1 |
| Cluster24 | CL:4665 | Interferon alpha/beta signaling | 5 | 8.82492 | bottom of list | 0.0014 | afc | ENSP00000359787,ENSP00000368699,ENSP00000381601,ENSP00000386187,ENSP00000483430 | IFI27,IFI44L,IFITM1,ISG15,MX1 |
| Cluster25 | CL:22328 | respirasome | 9 | 7.09545 | top of list | 0.007 | afc | ENSP00000354499,ENSP00000354554,ENSP00000354632,ENSP00000354728,ENSP00000354813,ENSP00000354876,ENSP00000354961,ENSP00000354982,ENSP00000355206 | MT-ATP6,MT-CO1,MT-CO2,MT-CO3,MT-CYB,MT-ND3,MT-ND4,MT-ND4L,MT-ND5 |
| Cluster25 | CL:22502 | Leber hereditary optic neuropathy | 5 | 7.73848 | top of list | 0.0096 | afc | ENSP00000354499,ENSP00000354554,ENSP00000354632,ENSP00000354961,ENSP00000354982 | MT-ATP6,MT-CO1,MT-CO3,MT-CYB,MT-ND4 |
| **Cluster** | **#term ID** | **term description** | **genes mapped** | **enrichment score** | **direction** | **false discovery rate** | **method** | **matching proteins in your input (IDs)** | **matching proteins in your input (labels)** |
| Cluster26 | CL:4665 | Interferon alpha/beta signaling | 8 | 6.53665 | bottom of list | 0.0035 | afc | ENSP00000342513,ENSP00000354822,ENSP00000359787,ENSP00000368699,ENSP00000381601,ENSP00000382707,ENSP00000386187,ENSP00000483430 | IFI27,IFI44L,IFI6,IFITM1,IFITM3,ISG15,MX1,XAF1 |
| Cluster29 | CL:4661 | Interferon alpha/beta signaling, and Interferon gamma signaling | 3 | 7.99432 | bottom of list | 0.0057 | afc | ENSP00000245185,ENSP00000382707,ENSP00000386187 | IFITM1,IFITM3,MT2A |

**Table S5 Compared with the normal controls, significantly enriched pathways of each cell (sub)types in mild patient 10. Cell type refers to Fig. 1d.**

| **Cluster** | **#term ID** | **term description** | **genes mapped** | **enrichment score** | **direction** | **false discovery rate** | **method** | **matching proteins in your input (labels)** |
| --- | --- | --- | --- | --- | --- | --- | --- | --- |
| Cluster3 | CL:4212 | Immunoregulatory interactions between a Lymphoid and a non-Lymphoid cell, and Cytolysis | 10 | 3.35199 | top of list | 0.006 | afc | B2M,GNLY,GZMB,GZMH,HLA-A,HLA-B,HLA-C,HLA-E,NKG7,PRF1 |
| Cluster4 | CL:2403 | mixed, incl. MAPK targets/ Nuclear events mediated by MAP kinases, and bZIP Maf transcription factor | 4 | 9.56577 | bottom of list | 0.0061 | afc | DUSP1,FOS,JUN,JUNB |
| Cluster4 | CL:2482 | AP-1 transcription factor, and Transcription factor Jun | 3 | 9.74427 | bottom of list | 0.007 | afc | FOS,JUN,JUNB |
| Cluster5 | CL:14966 | GTP hydrolysis and joining of the 60S ribosomal subunit, and Protein export | 78 | 1.21139 | top of list | 0.0034 | ks | EEF1A1,EEF1B2,EEF1D,EEF2,EIF1,EIF3F,EIF3H,EIF3L,EIF4B,FAU,GNB2L1,PABPC1,RPL10,RPL10A,RPL11,RPL12,RPL13,RPL14,RPL15,RPL17,RPL18,RPL18A,RPL19,RPL21,RPL22,RPL23A,RPL24,RPL26,RPL27,RPL28,RPL29,RPL3,RPL30,RPL32,RPL34,RPL35,RPL35A,RPL36,RPL36A,RPL36AL,RPL37,RPL37A,RPL39,RPL4,RPL5,RPL6,RPL7A,RPL8,RPL9,RPLP0,RPLP1,RPS12,RPS13,RPS14,RPS15,RPS15A,RPS16,RPS18,RPS19,RPS21,RPS23,RPS24,RPS25,RPS26,RPS27,RPS27A,RPS28,RPS29,RPS3,RPS3A,RPS4X,RPS5,RPS6,RPS7,RPS8,RPS9,RPSA,TPT1 |
| Cluster5 | CL:14982 | Peptide chain elongation | 60 | 1.46967 | top of list | 0.0034 | ks | EEF2,FAU,RPL10,RPL10A,RPL11,RPL12,RPL13,RPL14,RPL15,RPL17,RPL18A,RPL19,RPL21,RPL22,RPL23A,RPL24,RPL26,RPL27,RPL28,RPL29,RPL3,RPL32,RPL35,RPL35A,RPL36,RPL36A,RPL37,RPL37A,RPL39,RPL5,RPL6,RPL7A,RPL8,RPL9,RPLP0,RPLP1,RPS12,RPS13,RPS14,RPS15,RPS15A,RPS16,RPS18,RPS19,RPS21,RPS23,RPS24,RPS25,RPS26,RPS27,RPS27A,RPS28,RPS29,RPS3A,RPS4X,RPS5,RPS6,RPS7,RPS8,RPS9 |
| Cluster5 | CL:14983 | Peptide chain elongation | 52 | 1.44523 | top of list | 0.0034 | ks | EEF2,FAU,RPL10A,RPL11,RPL12,RPL13,RPL14,RPL15,RPL17,RPL18A,RPL19,RPL21,RPL22,RPL23A,RPL24,RPL26,RPL27,RPL29,RPL3,RPL32,RPL35,RPL35A,RPL36,RPL36A,RPL37,RPL37A,RPL39,RPL6,RPL7A,RPL8,RPL9,RPS12,RPS13,RPS14,RPS15,RPS15A,RPS16,RPS18,RPS19,RPS21,RPS23,RPS25,RPS26,RPS27A,RPS28,RPS3A,RPS4X,RPS5,RPS6,RPS7,RPS8,RPS9 |
| Cluster5 | CL:14980 | Peptide chain elongation | 61 | 1.46934 | top of list | 0.0034 | ks | EEF2,FAU,RPL10,RPL10A,RPL11,RPL12,RPL13,RPL14,RPL15,RPL17,RPL18,RPL18A,RPL19,RPL21,RPL22,RPL23A,RPL24,RPL26,RPL27,RPL28,RPL29,RPL3,RPL32,RPL35,RPL35A,RPL36,RPL36A,RPL37,RPL37A,RPL39,RPL5,RPL6,RPL7A,RPL8,RPL9,RPLP0,RPLP1,RPS12,RPS13,RPS14,RPS15,RPS15A,RPS16,RPS18,RPS19,RPS21,RPS23,RPS24,RPS25,RPS26,RPS27,RPS27A,RPS28,RPS29,RPS3A,RPS4X,RPS5,RPS6,RPS7,RPS8,RPS9 |
| Cluster5 | CL:14963 | GTP hydrolysis and joining of the 60S ribosomal subunit, and Nonsense-mediated mRNA decay | 80 | 1.19598 | top of list | 0.0034 | ks | BTF3,EEF1A1,EEF1B2,EEF1D,EEF2,EIF1,EIF3F,EIF3H,EIF3L,EIF4B,FAU,GNB2L1,NACA,PABPC1,RPL10,RPL10A,RPL11,RPL12,RPL13,RPL14,RPL15,RPL17,RPL18,RPL18A,RPL19,RPL21,RPL22,RPL23A,RPL24,RPL26,RPL27,RPL28,RPL29,RPL3,RPL30,RPL32,RPL34,RPL35,RPL35A,RPL36,RPL36A,RPL36AL,RPL37,RPL37A,RPL39,RPL4,RPL5,RPL6,RPL7A,RPL8,RPL9,RPLP0,RPLP1,RPS12,RPS13,RPS14,RPS15,RPS15A,RPS16,RPS18,RPS19,RPS21,RPS23,RPS24,RPS25,RPS26,RPS27,RPS27A,RPS28,RPS29,RPS3,RPS3A,RPS4X,RPS5,RPS6,RPS7,RPS8,RPS9,RPSA,TPT1 |
| Cluster5 | CL:14976 | Peptide chain elongation | 67 | 1.44496 | top of list | 0.0034 | ks | EEF1A1,EEF2,FAU,GNB2L1,RPL10,RPL10A,RPL11,RPL12,RPL13,RPL14,RPL15,RPL17,RPL18,RPL18A,RPL19,RPL21,RPL22,RPL23A,RPL24,RPL26,RPL27,RPL28,RPL29,RPL3,RPL30,RPL32,RPL34,RPL35,RPL35A,RPL36,RPL36A,RPL36AL,RPL37,RPL37A,RPL39,RPL4,RPL5,RPL6,RPL7A,RPL8,RPL9,RPLP0,RPLP1,RPS12,RPS13,RPS14,RPS15,RPS15A,RPS16,RPS18,RPS19,RPS21,RPS23,RPS24,RPS25,RPS26,RPS27,RPS27A,RPS28,RPS29,RPS3A,RPS4X,RPS5,RPS6,RPS7,RPS8,RPS9 |
| Cluster5 | CL:14978 | Peptide chain elongation | 65 | 1.50716 | top of list | 0.0034 | ks | EEF1A1,EEF2,FAU,GNB2L1,RPL10,RPL10A,RPL11,RPL12,RPL13,RPL14,RPL15,RPL17,RPL18,RPL18A,RPL19,RPL21,RPL22,RPL23A,RPL24,RPL26,RPL27,RPL28,RPL29,RPL3,RPL32,RPL34,RPL35,RPL35A,RPL36,RPL36A,RPL37,RPL37A,RPL39,RPL4,RPL5,RPL6,RPL7A,RPL8,RPL9,RPLP0,RPLP1,RPS12,RPS13,RPS14,RPS15,RPS15A,RPS16,RPS18,RPS19,RPS21,RPS23,RPS24,RPS25,RPS26,RPS27,RPS27A,RPS28,RPS29,RPS3A,RPS4X,RPS5,RPS6,RPS7,RPS8,RPS9 |
| Cluster5 | CL:14967 | GTP hydrolysis and joining of the 60S ribosomal subunit, and Protein export | 75 | 1.21231 | top of list | 0.0065 | ks | EEF1A1,EEF2,EIF1,EIF3F,EIF3H,EIF3L,EIF4B,FAU,GNB2L1,PABPC1,RPL10,RPL10A,RPL11,RPL12,RPL13,RPL14,RPL15,RPL17,RPL18,RPL18A,RPL19,RPL21,RPL22,RPL23A,RPL24,RPL26,RPL27,RPL28,RPL29,RPL3,RPL30,RPL32,RPL34,RPL35,RPL35A,RPL36,RPL36A,RPL36AL,RPL37,RPL37A,RPL39,RPL4,RPL5,RPL6,RPL7A,RPL8,RPL9,RPLP0,RPLP1,RPS12,RPS13,RPS14,RPS15,RPS15A,RPS16,RPS18,RPS19,RPS21,RPS23,RPS24,RPS25,RPS26,RPS27,RPS27A,RPS28,RPS29,RPS3,RPS3A,RPS4X,RPS5,RPS6,RPS7,RPS8,RPS9,RPSA |
| Cluster5 | CL:14985 | Viral mRNA Translation | 48 | 1.24966 | top of list | 0.008 | ks | FAU,RPL10A,RPL11,RPL12,RPL13,RPL14,RPL15,RPL17,RPL18A,RPL19,RPL21,RPL22,RPL23A,RPL24,RPL26,RPL27,RPL29,RPL3,RPL35,RPL35A,RPL36,RPL37A,RPL39,RPL6,RPL7A,RPL8,RPL9,RPS12,RPS13,RPS14,RPS15,RPS15A,RPS16,RPS18,RPS19,RPS21,RPS23,RPS25,RPS26,RPS27A,RPS28,RPS3A,RPS4X,RPS5,RPS6,RPS7,RPS8,RPS9 |
| Cluster6 | CL:22328 | respirasome | 7 | 4.62803 | top of list | 0.0043 | afc | MT-ATP6,MT-CO1,MT-CO2,MT-CO3,MT-CYB,MT-ND4L,MT-ND5 |
| Cluster6 | CL:22327 | Oxidative phosphorylation | 8 | 4.33173 | top of list | 0.0043 | afc | MT-ATP6,MT-ATP8,MT-CO1,MT-CO2,MT-CO3,MT-CYB,MT-ND4L,MT-ND5 |
| Cluster10 | CL:7134 | G alpha (i) signalling events | 6 | 6.63159 | bottom of list | 1.40E-05 | afc | ANXA1,CCL3,CCL4,CCL4L1,CXCL2,CXCL8 |
| Cluster10 | CL:7361 | Chemokine receptors bind chemokines | 3 | 7.07548 | bottom of list | 0.0011 | afc | CCL4L1,CXCL2,CXCL8 |
| Cluster10 | CL:7357 | Chemokine receptors bind chemokines | 4 | 5.10428 | bottom of list | 0.0089 | afc | ANXA1,CCL4L1,CXCL2,CXCL8 |
| Cluster12 | CL:7134 | G alpha (i) signalling events | 8 | 4.9836 | bottom of list | 9.56E-05 | afc | ANXA1,CCL3,CCL4,CCL4L1,CXCL2,CXCL8,GPSM3,PPBP |
| Cluster12 | CL:14973 | Peptide chain elongation | 68 | 2.5636 | top of list | 0.0028 | ks | EEF1A1,EEF2,FAU,GNB2L1,RPL10,RPL10A,RPL11,RPL12,RPL13,RPL14,RPL15,RPL18,RPL18A,RPL19,RPL21,RPL22,RPL23A,RPL24,RPL26,RPL27,RPL28,RPL29,RPL3,RPL30,RPL31,RPL32,RPL34,RPL35,RPL35A,RPL36,RPL36A,RPL36AL,RPL37,RPL39,RPL4,RPL5,RPL6,RPL7,RPL7A,RPL8,RPL9,RPLP0,RPLP1,RPLP2,RPS12,RPS13,RPS14,RPS15,RPS15A,RPS16,RPS18,RPS19,RPS21,RPS23,RPS24,RPS25,RPS26,RPS27A,RPS28,RPS3A,RPS4X,RPS5,RPS6,RPS7,RPS8,RPS9,SRP14,UBA52 |
| Cluster12 | CL:14963 | GTP hydrolysis and joining of the 60S ribosomal subunit, and Nonsense-mediated mRNA decay | 81 | 2.52938 | top of list | 0.0028 | ks | BTF3,EEF1A1,EEF1B2,EEF1D,EEF2,EIF1,EIF3F,EIF3H,EIF3K,EIF4B,FAU,GNB2L1,NACA,PABPC1,RPL10,RPL10A,RPL11,RPL12,RPL13,RPL14,RPL15,RPL18,RPL18A,RPL19,RPL21,RPL22,RPL23A,RPL24,RPL26,RPL27,RPL28,RPL29,RPL3,RPL30,RPL31,RPL32,RPL34,RPL35,RPL35A,RPL36,RPL36A,RPL36AL,RPL37,RPL39,RPL4,RPL5,RPL6,RPL7,RPL7A,RPL8,RPL9,RPLP0,RPLP1,RPLP2,RPS12,RPS13,RPS14,RPS15,RPS15A,RPS16,RPS18,RPS19,RPS21,RPS23,RPS24,RPS25,RPS26,RPS27A,RPS28,RPS3,RPS3A,RPS4X,RPS5,RPS6,RPS7,RPS8,RPS9,RPSA,SRP14,TPT1,UBA52 |
| Cluster12 | CL:7361 | Chemokine receptors bind chemokines | 4 | 5.35025 | bottom of list | 0.0028 | afc | CCL4L1,CXCL2,CXCL8,PPBP |
| **Cluster** | **#term ID** | **term description** | **genes mapped** | **enrichment score** | **direction** | **false discovery rate** | **method** | **matching proteins in your input (labels)** |
| Cluster12 | CL:14982 | Peptide chain elongation | 60 | 2.63808 | top of list | 0.0028 | ks | EEF2,FAU,RPL10,RPL10A,RPL11,RPL12,RPL13,RPL14,RPL15,RPL18A,RPL19,RPL21,RPL22,RPL23A,RPL24,RPL26,RPL27,RPL28,RPL29,RPL3,RPL31,RPL32,RPL35,RPL35A,RPL36,RPL36A,RPL37,RPL39,RPL5,RPL6,RPL7,RPL7A,RPL8,RPL9,RPLP0,RPLP1,RPLP2,RPS12,RPS13,RPS14,RPS15,RPS15A,RPS16,RPS18,RPS19,RPS21,RPS23,RPS24,RPS25,RPS26,RPS27A,RPS28,RPS3A,RPS4X,RPS5,RPS6,RPS7,RPS8,RPS9,UBA52 |
| Cluster12 | CL:14980 | Peptide chain elongation | 61 | 2.6953 | top of list | 0.0028 | ks | EEF2,FAU,RPL10,RPL10A,RPL11,RPL12,RPL13,RPL14,RPL15,RPL18,RPL18A,RPL19,RPL21,RPL22,RPL23A,RPL24,RPL26,RPL27,RPL28,RPL29,RPL3,RPL31,RPL32,RPL35,RPL35A,RPL36,RPL36A,RPL37,RPL39,RPL5,RPL6,RPL7,RPL7A,RPL8,RPL9,RPLP0,RPLP1,RPLP2,RPS12,RPS13,RPS14,RPS15,RPS15A,RPS16,RPS18,RPS19,RPS21,RPS23,RPS24,RPS25,RPS26,RPS27A,RPS28,RPS3A,RPS4X,RPS5,RPS6,RPS7,RPS8,RPS9,UBA52 |
| Cluster12 | CL:14976 | Peptide chain elongation | 67 | 2.6322 | top of list | 0.0028 | ks | EEF1A1,EEF2,FAU,GNB2L1,RPL10,RPL10A,RPL11,RPL12,RPL13,RPL14,RPL15,RPL18,RPL18A,RPL19,RPL21,RPL22,RPL23A,RPL24,RPL26,RPL27,RPL28,RPL29,RPL3,RPL30,RPL31,RPL32,RPL34,RPL35,RPL35A,RPL36,RPL36A,RPL36AL,RPL37,RPL39,RPL4,RPL5,RPL6,RPL7,RPL7A,RPL8,RPL9,RPLP0,RPLP1,RPLP2,RPS12,RPS13,RPS14,RPS15,RPS15A,RPS16,RPS18,RPS19,RPS21,RPS23,RPS24,RPS25,RPS26,RPS27A,RPS28,RPS3A,RPS4X,RPS5,RPS6,RPS7,RPS8,RPS9,UBA52 |
| Cluster12 | CL:14978 | Peptide chain elongation | 65 | 2.64477 | top of list | 0.0028 | ks | EEF1A1,EEF2,FAU,GNB2L1,RPL10,RPL10A,RPL11,RPL12,RPL13,RPL14,RPL15,RPL18,RPL18A,RPL19,RPL21,RPL22,RPL23A,RPL24,RPL26,RPL27,RPL28,RPL29,RPL3,RPL31,RPL32,RPL34,RPL35,RPL35A,RPL36,RPL36A,RPL37,RPL39,RPL4,RPL5,RPL6,RPL7,RPL7A,RPL8,RPL9,RPLP0,RPLP1,RPLP2,RPS12,RPS13,RPS14,RPS15,RPS15A,RPS16,RPS18,RPS19,RPS21,RPS23,RPS24,RPS25,RPS26,RPS27A,RPS28,RPS3A,RPS4X,RPS5,RPS6,RPS7,RPS8,RPS9,UBA52 |
| Cluster12 | CL:14966 | GTP hydrolysis and joining of the 60S ribosomal subunit, and Protein export | 79 | 2.46556 | top of list | 0.0037 | ks | EEF1A1,EEF1B2,EEF1D,EEF2,EIF1,EIF3F,EIF3H,EIF3K,EIF4B,FAU,GNB2L1,PABPC1,RPL10,RPL10A,RPL11,RPL12,RPL13,RPL14,RPL15,RPL18,RPL18A,RPL19,RPL21,RPL22,RPL23A,RPL24,RPL26,RPL27,RPL28,RPL29,RPL3,RPL30,RPL31,RPL32,RPL34,RPL35,RPL35A,RPL36,RPL36A,RPL36AL,RPL37,RPL39,RPL4,RPL5,RPL6,RPL7,RPL7A,RPL8,RPL9,RPLP0,RPLP1,RPLP2,RPS12,RPS13,RPS14,RPS15,RPS15A,RPS16,RPS18,RPS19,RPS21,RPS23,RPS24,RPS25,RPS26,RPS27A,RPS28,RPS3,RPS3A,RPS4X,RPS5,RPS6,RPS7,RPS8,RPS9,RPSA,SRP14,TPT1,UBA52 |
| Cluster12 | CL:14967 | GTP hydrolysis and joining of the 60S ribosomal subunit, and Protein export | 76 | 2.43268 | top of list | 0.0057 | ks | EEF1A1,EEF2,EIF1,EIF3F,EIF3H,EIF3K,EIF4B,FAU,GNB2L1,PABPC1,RPL10,RPL10A,RPL11,RPL12,RPL13,RPL14,RPL15,RPL18,RPL18A,RPL19,RPL21,RPL22,RPL23A,RPL24,RPL26,RPL27,RPL28,RPL29,RPL3,RPL30,RPL31,RPL32,RPL34,RPL35,RPL35A,RPL36,RPL36A,RPL36AL,RPL37,RPL39,RPL4,RPL5,RPL6,RPL7,RPL7A,RPL8,RPL9,RPLP0,RPLP1,RPLP2,RPS12,RPS13,RPS14,RPS15,RPS15A,RPS16,RPS18,RPS19,RPS21,RPS23,RPS24,RPS25,RPS26,RPS27A,RPS28,RPS3,RPS3A,RPS4X,RPS5,RPS6,RPS7,RPS8,RPS9,RPSA,SRP14,UBA52 |
| Cluster12 | CL:14985 | Viral mRNA Translation | 48 | 2.62445 | top of list | 0.0057 | ks | FAU,RPL10A,RPL11,RPL12,RPL13,RPL14,RPL15,RPL18A,RPL19,RPL21,RPL22,RPL23A,RPL24,RPL26,RPL27,RPL29,RPL3,RPL35,RPL35A,RPL36,RPL39,RPL6,RPL7,RPL7A,RPL8,RPL9,RPS12,RPS13,RPS14,RPS15,RPS15A,RPS16,RPS18,RPS19,RPS21,RPS23,RPS25,RPS26,RPS27A,RPS28,RPS3A,RPS4X,RPS5,RPS6,RPS7,RPS8,RPS9,UBA52 |
| Cluster12 | CL:14983 | Peptide chain elongation | 53 | 2.39563 | top of list | 0.0057 | ks | EEF2,FAU,RPL10A,RPL11,RPL12,RPL13,RPL14,RPL15,RPL18A,RPL19,RPL21,RPL22,RPL23A,RPL24,RPL26,RPL27,RPL29,RPL3,RPL32,RPL35,RPL35A,RPL36,RPL36A,RPL37,RPL39,RPL6,RPL7,RPL7A,RPL8,RPL9,RPLP2,RPS12,RPS13,RPS14,RPS15,RPS15A,RPS16,RPS18,RPS19,RPS21,RPS23,RPS25,RPS26,RPS27A,RPS28,RPS3A,RPS4X,RPS5,RPS6,RPS7,RPS8,RPS9,UBA52 |
| Cluster13 | CL:4300 | mixed, incl. Granzyme B, and Granulysin-like | 5 | 6.15733 | top of list | 0.0017 | afc | GNLY,GZMB,GZMH,NKG7,PRF1 |
| Cluster13 | CL:2403 | mixed, incl. MAPK targets/ Nuclear events mediated by MAP kinases, and bZIP Maf transcription factor | 3 | 9.06862 | bottom of list | 0.0017 | afc | DUSP1,FOS,JUN |
| Cluster16 | CL:14976 | Peptide chain elongation | 77 | 3.23795 | top of list | 3.50E-08 | ks | EEF1A1,EEF2,FAU,GNB2L1,RPL10,RPL10A,RPL11,RPL12,RPL13,RPL13A,RPL14,RPL15,RPL17,RPL18,RPL18A,RPL19,RPL21,RPL22,RPL23A,RPL24,RPL26,RPL27,RPL27A,RPL28,RPL29,RPL3,RPL30,RPL31,RPL32,RPL34,RPL35,RPL35A,RPL36,RPL36A,RPL36AL,RPL37,RPL37A,RPL38,RPL39,RPL4,RPL5,RPL6,RPL7,RPL7A,RPL8,RPL9,RPLP0,RPLP1,RPLP2,RPS11,RPS12,RPS13,RPS14,RPS15,RPS15A,RPS16,RPS18,RPS19,RPS2,RPS20,RPS21,RPS23,RPS24,RPS25,RPS26,RPS27,RPS27A,RPS28,RPS29,RPS3A,RPS4X,RPS5,RPS6,RPS7,RPS8,RPS9,UBA52 |
| Cluster16 | CL:14973 | Peptide chain elongation | 78 | 3.20628 | top of list | 3.50E-08 | ks | EEF1A1,EEF2,FAU,GNB2L1,RPL10,RPL10A,RPL11,RPL12,RPL13,RPL13A,RPL14,RPL15,RPL17,RPL18,RPL18A,RPL19,RPL21,RPL22,RPL23A,RPL24,RPL26,RPL27,RPL27A,RPL28,RPL29,RPL3,RPL30,RPL31,RPL32,RPL34,RPL35,RPL35A,RPL36,RPL36A,RPL36AL,RPL37,RPL37A,RPL38,RPL39,RPL4,RPL5,RPL6,RPL7,RPL7A,RPL8,RPL9,RPLP0,RPLP1,RPLP2,RPS11,RPS12,RPS13,RPS14,RPS15,RPS15A,RPS16,RPS18,RPS19,RPS2,RPS20,RPS21,RPS23,RPS24,RPS25,RPS26,RPS27,RPS27A,RPS28,RPS29,RPS3A,RPS4X,RPS5,RPS6,RPS7,RPS8,RPS9,SRP14,UBA52 |
| Cluster16 | CL:14978 | Peptide chain elongation | 75 | 3.20394 | top of list | 3.93E-08 | ks | EEF1A1,EEF2,FAU,GNB2L1,RPL10,RPL10A,RPL11,RPL12,RPL13,RPL13A,RPL14,RPL15,RPL17,RPL18,RPL18A,RPL19,RPL21,RPL22,RPL23A,RPL24,RPL26,RPL27,RPL27A,RPL28,RPL29,RPL3,RPL31,RPL32,RPL34,RPL35,RPL35A,RPL36,RPL36A,RPL37,RPL37A,RPL38,RPL39,RPL4,RPL5,RPL6,RPL7,RPL7A,RPL8,RPL9,RPLP0,RPLP1,RPLP2,RPS11,RPS12,RPS13,RPS14,RPS15,RPS15A,RPS16,RPS18,RPS19,RPS2,RPS20,RPS21,RPS23,RPS24,RPS25,RPS26,RPS27,RPS27A,RPS28,RPS29,RPS3A,RPS4X,RPS5,RPS6,RPS7,RPS8,RPS9,UBA52 |
| Cluster16 | CL:14963 | GTP hydrolysis and joining of the 60S ribosomal subunit, and Nonsense-mediated mRNA decay | 95 | 2.87205 | top of list | 4.46E-08 | ks | BTF3,EEF1A1,EEF1B2,EEF1D,EEF2,EIF1,EIF3F,EIF3H,EIF3K,EIF3L,EIF4B,FAU,GNB2L1,NACA,PABPC1,RPL10,RPL10A,RPL11,RPL12,RPL13,RPL13A,RPL14,RPL15,RPL17,RPL18,RPL18A,RPL19,RPL21,RPL22,RPL23A,RPL24,RPL26,RPL27,RPL27A,RPL28,RPL29,RPL3,RPL30,RPL31,RPL32,RPL34,RPL35,RPL35A,RPL36,RPL36A,RPL36AL,RPL37,RPL37A,RPL38,RPL39,RPL4,RPL5,RPL6,RPL7,RPL7A,RPL8,RPL9,RPLP0,RPLP1,RPLP2,RPS11,RPS12,RPS13,RPS14,RPS15,RPS15A,RPS16,RPS18,RPS19,RPS2,RPS20,RPS21,RPS23,RPS24,RPS25,RPS26,RPS27,RPS27A,RPS28,RPS29,RPS3,RPS3A,RPS4X,RPS5,RPS6,RPS7,RPS8,RPS9,RPSA,SRP14,SRRM1,SSR2,SSR4,TPT1,UBA52 |
| Cluster16 | CL:14966 | GTP hydrolysis and joining of the 60S ribosomal subunit, and Protein export | 92 | 2.90427 | top of list | 4.46E-08 | ks | EEF1A1,EEF1B2,EEF1D,EEF2,EIF1,EIF3F,EIF3H,EIF3K,EIF3L,EIF4B,FAU,GNB2L1,PABPC1,RPL10,RPL10A,RPL11,RPL12,RPL13,RPL13A,RPL14,RPL15,RPL17,RPL18,RPL18A,RPL19,RPL21,RPL22,RPL23A,RPL24,RPL26,RPL27,RPL27A,RPL28,RPL29,RPL3,RPL30,RPL31,RPL32,RPL34,RPL35,RPL35A,RPL36,RPL36A,RPL36AL,RPL37,RPL37A,RPL38,RPL39,RPL4,RPL5,RPL6,RPL7,RPL7A,RPL8,RPL9,RPLP0,RPLP1,RPLP2,RPS11,RPS12,RPS13,RPS14,RPS15,RPS15A,RPS16,RPS18,RPS19,RPS2,RPS20,RPS21,RPS23,RPS24,RPS25,RPS26,RPS27,RPS27A,RPS28,RPS29,RPS3,RPS3A,RPS4X,RPS5,RPS6,RPS7,RPS8,RPS9,RPSA,SRP14,SSR2,SSR4,TPT1,UBA52 |
| Cluster16 | CL:14985 | Viral mRNA Translation | 54 | 3.52564 | top of list | 4.46E-08 | ks | FAU,RPL10A,RPL11,RPL12,RPL13,RPL13A,RPL14,RPL15,RPL17,RPL18A,RPL19,RPL21,RPL22,RPL23A,RPL24,RPL26,RPL27,RPL29,RPL3,RPL35,RPL35A,RPL36,RPL37A,RPL38,RPL39,RPL6,RPL7,RPL7A,RPL8,RPL9,RPS11,RPS12,RPS13,RPS14,RPS15,RPS15A,RPS16,RPS18,RPS19,RPS2,RPS21,RPS23,RPS25,RPS26,RPS27A,RPS28,RPS3A,RPS4X,RPS5,RPS6,RPS7,RPS8,RPS9,UBA52 |
| Cluster16 | CL:14980 | Peptide chain elongation | 71 | 3.23139 | top of list | 4.46E-08 | ks | EEF2,FAU,RPL10,RPL10A,RPL11,RPL12,RPL13,RPL13A,RPL14,RPL15,RPL17,RPL18,RPL18A,RPL19,RPL21,RPL22,RPL23A,RPL24,RPL26,RPL27,RPL27A,RPL28,RPL29,RPL3,RPL31,RPL32,RPL35,RPL35A,RPL36,RPL36A,RPL37,RPL37A,RPL38,RPL39,RPL5,RPL6,RPL7,RPL7A,RPL8,RPL9,RPLP0,RPLP1,RPLP2,RPS11,RPS12,RPS13,RPS14,RPS15,RPS15A,RPS16,RPS18,RPS19,RPS2,RPS20,RPS21,RPS23,RPS24,RPS25,RPS26,RPS27,RPS27A,RPS28,RPS29,RPS3A,RPS4X,RPS5,RPS6,RPS7,RPS8,RPS9,UBA52 |
| Cluster16 | CL:14968 | SRP-dependent cotranslational protein targeting to membrane | 80 | 3.07064 | top of list | 4.46E-08 | ks | EEF1A1,EEF2,FAU,GNB2L1,RPL10,RPL10A,RPL11,RPL12,RPL13,RPL13A,RPL14,RPL15,RPL17,RPL18,RPL18A,RPL19,RPL21,RPL22,RPL23A,RPL24,RPL26,RPL27,RPL27A,RPL28,RPL29,RPL3,RPL30,RPL31,RPL32,RPL34,RPL35,RPL35A,RPL36,RPL36A,RPL36AL,RPL37,RPL37A,RPL38,RPL39,RPL4,RPL5,RPL6,RPL7,RPL7A,RPL8,RPL9,RPLP0,RPLP1,RPLP2,RPS11,RPS12,RPS13,RPS14,RPS15,RPS15A,RPS16,RPS18,RPS19,RPS2,RPS20,RPS21,RPS23,RPS24,RPS25,RPS26,RPS27,RPS27A,RPS28,RPS29,RPS3A,RPS4X,RPS5,RPS6,RPS7,RPS8,RPS9,SRP14,SSR2,SSR4,UBA52 |
| Cluster16 | CL:14982 | Peptide chain elongation | 69 | 3.2569 | top of list | 4.46E-08 | ks | EEF2,FAU,RPL10,RPL10A,RPL11,RPL12,RPL13,RPL13A,RPL14,RPL15,RPL17,RPL18A,RPL19,RPL21,RPL22,RPL23A,RPL24,RPL26,RPL27,RPL28,RPL29,RPL3,RPL31,RPL32,RPL35,RPL35A,RPL36,RPL36A,RPL37,RPL37A,RPL38,RPL39,RPL5,RPL6,RPL7,RPL7A,RPL8,RPL9,RPLP0,RPLP1,RPLP2,RPS11,RPS12,RPS13,RPS14,RPS15,RPS15A,RPS16,RPS18,RPS19,RPS2,RPS20,RPS21,RPS23,RPS24,RPS25,RPS26,RPS27,RPS27A,RPS28,RPS29,RPS3A,RPS4X,RPS5,RPS6,RPS7,RPS8,RPS9,UBA52 |
| Cluster16 | CL:14965 | GTP hydrolysis and joining of the 60S ribosomal subunit, and Nonsense-mediated mRNA decay | 93 | 2.86642 | top of list | 8.37E-08 | ks | EEF1A1,EEF1B2,EEF1D,EEF2,EIF1,EIF3F,EIF3H,EIF3K,EIF3L,EIF4B,FAU,GNB2L1,PABPC1,RPL10,RPL10A,RPL11,RPL12,RPL13,RPL13A,RPL14,RPL15,RPL17,RPL18,RPL18A,RPL19,RPL21,RPL22,RPL23A,RPL24,RPL26,RPL27,RPL27A,RPL28,RPL29,RPL3,RPL30,RPL31,RPL32,RPL34,RPL35,RPL35A,RPL36,RPL36A,RPL36AL,RPL37,RPL37A,RPL38,RPL39,RPL4,RPL5,RPL6,RPL7,RPL7A,RPL8,RPL9,RPLP0,RPLP1,RPLP2,RPS11,RPS12,RPS13,RPS14,RPS15,RPS15A,RPS16,RPS18,RPS19,RPS2,RPS20,RPS21,RPS23,RPS24,RPS25,RPS26,RPS27,RPS27A,RPS28,RPS29,RPS3,RPS3A,RPS4X,RPS5,RPS6,RPS7,RPS8,RPS9,RPSA,SRP14,SRRM1,SSR2,SSR4,TPT1,UBA52 |
| **Cluster** | **#term ID** | **term description** | **genes mapped** | **enrichment score** | **direction** | **false discovery rate** | **method** | **matching proteins in your input (labels)** |
| Cluster16 | CL:14983 | Peptide chain elongation | 59 | 3.39707 | top of list | 9.20E-08 | ks | EEF2,FAU,RPL10A,RPL11,RPL12,RPL13,RPL13A,RPL14,RPL15,RPL17,RPL18A,RPL19,RPL21,RPL22,RPL23A,RPL24,RPL26,RPL27,RPL29,RPL3,RPL32,RPL35,RPL35A,RPL36,RPL36A,RPL37,RPL37A,RPL38,RPL39,RPL6,RPL7,RPL7A,RPL8,RPL9,RPLP2,RPS11,RPS12,RPS13,RPS14,RPS15,RPS15A,RPS16,RPS18,RPS19,RPS2,RPS21,RPS23,RPS25,RPS26,RPS27A,RPS28,RPS3A,RPS4X,RPS5,RPS6,RPS7,RPS8,RPS9,UBA52 |
| Cluster16 | CL:14967 | GTP hydrolysis and joining of the 60S ribosomal subunit, and Protein export | 89 | 2.89751 | top of list | 9.80E-08 | ks | EEF1A1,EEF2,EIF1,EIF3F,EIF3H,EIF3K,EIF3L,EIF4B,FAU,GNB2L1,PABPC1,RPL10,RPL10A,RPL11,RPL12,RPL13,RPL13A,RPL14,RPL15,RPL17,RPL18,RPL18A,RPL19,RPL21,RPL22,RPL23A,RPL24,RPL26,RPL27,RPL27A,RPL28,RPL29,RPL3,RPL30,RPL31,RPL32,RPL34,RPL35,RPL35A,RPL36,RPL36A,RPL36AL,RPL37,RPL37A,RPL38,RPL39,RPL4,RPL5,RPL6,RPL7,RPL7A,RPL8,RPL9,RPLP0,RPLP1,RPLP2,RPS11,RPS12,RPS13,RPS14,RPS15,RPS15A,RPS16,RPS18,RPS19,RPS2,RPS20,RPS21,RPS23,RPS24,RPS25,RPS26,RPS27,RPS27A,RPS28,RPS29,RPS3,RPS3A,RPS4X,RPS5,RPS6,RPS7,RPS8,RPS9,RPSA,SRP14,SSR2,SSR4,UBA52 |
| Cluster16 | CL:7134 | G alpha (i) signalling events | 6 | 4.45482 | bottom of list | 0.0021 | afc | CCL3,CCL4,CCL4L1,CCL5,CXCL10,GPSM3 |
| Cluster19 | CL:4560 | immunoglobulin binding, and Immunoregulatory interactions between a Lymphoid and a non-Lymphoid cell | 3 | 6.49368 | bottom of list | 0.00079 | afc | KLRG1,S100A8,S100A9 |
| Cluster19 | CL:4212 | Immunoregulatory interactions between a Lymphoid and a non-Lymphoid cell, and Cytolysis | 11 | 3.48459 | top of list | 0.00079 | afc | B2M,GNLY,GZMB,GZMH,HCST,HLA-A,HLA-B,HLA-C,HLA-E,HLA-F,NKG7 |
| Cluster19 | CL:14980 | Peptide chain elongation | 66 | 1.11225 | top of list | 0.00079 | ks | EEF2,FAU,RPL10,RPL10A,RPL11,RPL12,RPL13,RPL13A,RPL14,RPL15,RPL18,RPL18A,RPL19,RPL21,RPL22,RPL23A,RPL24,RPL26,RPL27,RPL27A,RPL28,RPL29,RPL3,RPL31,RPL32,RPL35,RPL35A,RPL36,RPL36A,RPL37,RPL37A,RPL5,RPL6,RPL7,RPL7A,RPL8,RPL9,RPLP0,RPLP1,RPLP2,RPS12,RPS13,RPS14,RPS15,RPS15A,RPS16,RPS18,RPS19,RPS2,RPS20,RPS21,RPS23,RPS24,RPS25,RPS26,RPS27,RPS27A,RPS28,RPS3A,RPS4X,RPS5,RPS6,RPS7,RPS8,RPS9,UBA52 |
| Cluster19 | CL:14973 | Peptide chain elongation | 73 | 1.13391 | top of list | 0.00079 | ks | EEF1A1,EEF2,FAU,GNB2L1,RPL10,RPL10A,RPL11,RPL12,RPL13,RPL13A,RPL14,RPL15,RPL18,RPL18A,RPL19,RPL21,RPL22,RPL23A,RPL24,RPL26,RPL27,RPL27A,RPL28,RPL29,RPL3,RPL30,RPL31,RPL32,RPL34,RPL35,RPL35A,RPL36,RPL36A,RPL36AL,RPL37,RPL37A,RPL4,RPL5,RPL6,RPL7,RPL7A,RPL8,RPL9,RPLP0,RPLP1,RPLP2,RPS12,RPS13,RPS14,RPS15,RPS15A,RPS16,RPS18,RPS19,RPS2,RPS20,RPS21,RPS23,RPS24,RPS25,RPS26,RPS27,RPS27A,RPS28,RPS3A,RPS4X,RPS5,RPS6,RPS7,RPS8,RPS9,SRP14,UBA52 |
| Cluster19 | CL:14982 | Peptide chain elongation | 64 | 1.13563 | top of list | 0.00079 | ks | EEF2,FAU,RPL10,RPL10A,RPL11,RPL12,RPL13,RPL13A,RPL14,RPL15,RPL18A,RPL19,RPL21,RPL22,RPL23A,RPL24,RPL26,RPL27,RPL28,RPL29,RPL3,RPL31,RPL32,RPL35,RPL35A,RPL36,RPL36A,RPL37,RPL37A,RPL5,RPL6,RPL7,RPL7A,RPL8,RPL9,RPLP0,RPLP1,RPLP2,RPS12,RPS13,RPS14,RPS15,RPS15A,RPS16,RPS18,RPS19,RPS2,RPS20,RPS21,RPS23,RPS24,RPS25,RPS26,RPS27,RPS27A,RPS28,RPS3A,RPS4X,RPS5,RPS6,RPS7,RPS8,RPS9,UBA52 |
| Cluster19 | CL:14976 | Peptide chain elongation | 72 | 1.16435 | top of list | 0.00079 | ks | EEF1A1,EEF2,FAU,GNB2L1,RPL10,RPL10A,RPL11,RPL12,RPL13,RPL13A,RPL14,RPL15,RPL18,RPL18A,RPL19,RPL21,RPL22,RPL23A,RPL24,RPL26,RPL27,RPL27A,RPL28,RPL29,RPL3,RPL30,RPL31,RPL32,RPL34,RPL35,RPL35A,RPL36,RPL36A,RPL36AL,RPL37,RPL37A,RPL4,RPL5,RPL6,RPL7,RPL7A,RPL8,RPL9,RPLP0,RPLP1,RPLP2,RPS12,RPS13,RPS14,RPS15,RPS15A,RPS16,RPS18,RPS19,RPS2,RPS20,RPS21,RPS23,RPS24,RPS25,RPS26,RPS27,RPS27A,RPS28,RPS3A,RPS4X,RPS5,RPS6,RPS7,RPS8,RPS9,UBA52 |
| Cluster19 | CL:14978 | Peptide chain elongation | 70 | 1.17872 | top of list | 0.00079 | ks | EEF1A1,EEF2,FAU,GNB2L1,RPL10,RPL10A,RPL11,RPL12,RPL13,RPL13A,RPL14,RPL15,RPL18,RPL18A,RPL19,RPL21,RPL22,RPL23A,RPL24,RPL26,RPL27,RPL27A,RPL28,RPL29,RPL3,RPL31,RPL32,RPL34,RPL35,RPL35A,RPL36,RPL36A,RPL37,RPL37A,RPL4,RPL5,RPL6,RPL7,RPL7A,RPL8,RPL9,RPLP0,RPLP1,RPLP2,RPS12,RPS13,RPS14,RPS15,RPS15A,RPS16,RPS18,RPS19,RPS2,RPS20,RPS21,RPS23,RPS24,RPS25,RPS26,RPS27,RPS27A,RPS28,RPS3A,RPS4X,RPS5,RPS6,RPS7,RPS8,RPS9,UBA52 |
| Cluster19 | CL:4218 | MHC class I protein complex, and inhibitory MHC class I receptor activity | 6 | 4.46978 | top of list | 0.0014 | afc | B2M,HLA-A,HLA-B,HLA-C,HLA-E,HLA-F |
| Cluster19 | CL:4211 | Immunoregulatory interactions between a Lymphoid and a non-Lymphoid cell, and Cytolysis | 12 | 2.98798 | top of list | 0.0014 | afc | B2M,CD48,GNLY,GZMB,GZMH,HCST,HLA-A,HLA-B,HLA-C,HLA-E,HLA-F,NKG7 |
| Cluster19 | CL:14968 | SRP-dependent cotranslational protein targeting to membrane | 75 | 1.05302 | top of list | 0.0014 | ks | EEF1A1,EEF2,FAU,GNB2L1,RPL10,RPL10A,RPL11,RPL12,RPL13,RPL13A,RPL14,RPL15,RPL18,RPL18A,RPL19,RPL21,RPL22,RPL23A,RPL24,RPL26,RPL27,RPL27A,RPL28,RPL29,RPL3,RPL30,RPL31,RPL32,RPL34,RPL35,RPL35A,RPL36,RPL36A,RPL36AL,RPL37,RPL37A,RPL4,RPL5,RPL6,RPL7,RPL7A,RPL8,RPL9,RPLP0,RPLP1,RPLP2,RPS12,RPS13,RPS14,RPS15,RPS15A,RPS16,RPS18,RPS19,RPS2,RPS20,RPS21,RPS23,RPS24,RPS25,RPS26,RPS27,RPS27A,RPS28,RPS3A,RPS4X,RPS5,RPS6,RPS7,RPS8,RPS9,SRP14,SSR2,SSR4,UBA52 |
| Cluster19 | CL:14963 | GTP hydrolysis and joining of the 60S ribosomal subunit, and Nonsense-mediated mRNA decay | 88 | 1.01831 | top of list | 0.0014 | ks | BTF3,EEF1A1,EEF1B2,EEF1D,EEF2,EIF1,EIF3F,EIF3H,EIF3K,EIF4B,FAU,GNB2L1,NACA,PABPC1,RPL10,RPL10A,RPL11,RPL12,RPL13,RPL13A,RPL14,RPL15,RPL18,RPL18A,RPL19,RPL21,RPL22,RPL23A,RPL24,RPL26,RPL27,RPL27A,RPL28,RPL29,RPL3,RPL30,RPL31,RPL32,RPL34,RPL35,RPL35A,RPL36,RPL36A,RPL36AL,RPL37,RPL37A,RPL4,RPL5,RPL6,RPL7,RPL7A,RPL8,RPL9,RPLP0,RPLP1,RPLP2,RPS12,RPS13,RPS14,RPS15,RPS15A,RPS16,RPS18,RPS19,RPS2,RPS20,RPS21,RPS23,RPS24,RPS25,RPS26,RPS27,RPS27A,RPS28,RPS3,RPS3A,RPS4X,RPS5,RPS6,RPS7,RPS8,RPS9,RPSA,SRP14,SSR2,SSR4,TPT1,UBA52 |
| Cluster19 | CL:14966 | GTP hydrolysis and joining of the 60S ribosomal subunit, and Protein export | 86 | 1.00045 | top of list | 0.0019 | ks | EEF1A1,EEF1B2,EEF1D,EEF2,EIF1,EIF3F,EIF3H,EIF3K,EIF4B,FAU,GNB2L1,PABPC1,RPL10,RPL10A,RPL11,RPL12,RPL13,RPL13A,RPL14,RPL15,RPL18,RPL18A,RPL19,RPL21,RPL22,RPL23A,RPL24,RPL26,RPL27,RPL27A,RPL28,RPL29,RPL3,RPL30,RPL31,RPL32,RPL34,RPL35,RPL35A,RPL36,RPL36A,RPL36AL,RPL37,RPL37A,RPL4,RPL5,RPL6,RPL7,RPL7A,RPL8,RPL9,RPLP0,RPLP1,RPLP2,RPS12,RPS13,RPS14,RPS15,RPS15A,RPS16,RPS18,RPS19,RPS2,RPS20,RPS21,RPS23,RPS24,RPS25,RPS26,RPS27,RPS27A,RPS28,RPS3,RPS3A,RPS4X,RPS5,RPS6,RPS7,RPS8,RPS9,RPSA,SRP14,SSR2,SSR4,TPT1,UBA52 |
| Cluster19 | CL:14985 | Viral mRNA Translation | 50 | 1.23645 | top of list | 0.002 | ks | FAU,RPL10A,RPL11,RPL12,RPL13,RPL13A,RPL14,RPL15,RPL18A,RPL19,RPL21,RPL22,RPL23A,RPL24,RPL26,RPL27,RPL29,RPL3,RPL35,RPL35A,RPL36,RPL37A,RPL6,RPL7,RPL7A,RPL8,RPL9,RPS12,RPS13,RPS14,RPS15,RPS15A,RPS16,RPS18,RPS19,RPS2,RPS21,RPS23,RPS25,RPS26,RPS27A,RPS28,RPS3A,RPS4X,RPS5,RPS6,RPS7,RPS8,RPS9,UBA52 |
| Cluster19 | CL:4213 | Immunoregulatory interactions between a Lymphoid and a non-Lymphoid cell, and FCHSD2, SH3 domain 2 | 7 | 3.92771 | top of list | 0.002 | afc | B2M,HCST,HLA-A,HLA-B,HLA-C,HLA-E,HLA-F |
| Cluster19 | CL:4220 | MHC_I C-terminus, and MHC class I protein complex binding | 3 | 6.64446 | top of list | 0.002 | afc | HLA-B,HLA-C,HLA-E |
| Cluster19 | CL:14983 | Peptide chain elongation | 55 | 1.12733 | top of list | 0.0025 | ks | EEF2,FAU,RPL10A,RPL11,RPL12,RPL13,RPL13A,RPL14,RPL15,RPL18A,RPL19,RPL21,RPL22,RPL23A,RPL24,RPL26,RPL27,RPL29,RPL3,RPL32,RPL35,RPL35A,RPL36,RPL36A,RPL37,RPL37A,RPL6,RPL7,RPL7A,RPL8,RPL9,RPLP2,RPS12,RPS13,RPS14,RPS15,RPS15A,RPS16,RPS18,RPS19,RPS2,RPS21,RPS23,RPS25,RPS26,RPS27A,RPS28,RPS3A,RPS4X,RPS5,RPS6,RPS7,RPS8,RPS9,UBA52 |
| **Cluster** | **#term ID** | **term description** | **genes mapped** | **enrichment score** | **direction** | **false discovery rate** | **method** | **matching proteins in your input (labels)** |
| Cluster19 | CL:14967 | GTP hydrolysis and joining of the 60S ribosomal subunit, and Protein export | 83 | 0.975313 | top of list | 0.0035 | ks | EEF1A1,EEF2,EIF1,EIF3F,EIF3H,EIF3K,EIF4B,FAU,GNB2L1,PABPC1,RPL10,RPL10A,RPL11,RPL12,RPL13,RPL13A,RPL14,RPL15,RPL18,RPL18A,RPL19,RPL21,RPL22,RPL23A,RPL24,RPL26,RPL27,RPL27A,RPL28,RPL29,RPL3,RPL30,RPL31,RPL32,RPL34,RPL35,RPL35A,RPL36,RPL36A,RPL36AL,RPL37,RPL37A,RPL4,RPL5,RPL6,RPL7,RPL7A,RPL8,RPL9,RPLP0,RPLP1,RPLP2,RPS12,RPS13,RPS14,RPS15,RPS15A,RPS16,RPS18,RPS19,RPS2,RPS20,RPS21,RPS23,RPS24,RPS25,RPS26,RPS27,RPS27A,RPS28,RPS3,RPS3A,RPS4X,RPS5,RPS6,RPS7,RPS8,RPS9,RPSA,SRP14,SSR2,SSR4,UBA52 |
| Cluster19 | CL:4209 | mixed, incl. Immunoregulatory interactions between a Lymphoid and a non-Lymphoid cell, and T cell costimulation | 13 | 2.55558 | top of list | 0.0049 | afc | B2M,CD48,GNLY,GZMB,GZMH,HCST,HLA-A,HLA-B,HLA-C,HLA-E,HLA-F,NKG7,TNFRSF14 |
| Cluster19 | CL:2403 | mixed, incl. MAPK targets/ Nuclear events mediated by MAP kinases, and bZIP Maf transcription factor | 5 | 7.66341 | bottom of list | 0.0036 | afc | DUSP1,FOS,JUN,JUNB,JUND |
| Cluster19 | CL:14963 | GTP hydrolysis and joining of the 60S ribosomal subunit, and Nonsense-mediated mRNA decay | 75 | 1.48044 | top of list | 0.0013 | ks | EEF1A1,EEF1B2,EEF1D,EEF2,EIF1,EIF2S3,EIF4B,FAU,GNB2L1,NACA,PABPC1,PNRC2,RPL10,RPL10A,RPL11,RPL12,RPL13,RPL14,RPL15,RPL18,RPL18A,RPL19,RPL21,RPL22,RPL22L1,RPL23A,RPL26,RPL27,RPL29,RPL3,RPL30,RPL32,RPL34,RPL35A,RPL36,RPL36A,RPL36AL,RPL37,RPL37A,RPL39,RPL4,RPL5,RPL6,RPL7A,RPL8,RPL9,RPLP0,RPLP1,RPLP2,RPS12,RPS13,RPS14,RPS15,RPS15A,RPS18,RPS19,RPS20,RPS21,RPS23,RPS24,RPS27,RPS27A,RPS28,RPS3,RPS3A,RPS4X,RPS5,RPS6,RPS7,RPS8,RPS9,RPSA,SPCS2,SSR2,TPT1 |
| Cluster19 | CL:14967 | GTP hydrolysis and joining of the 60S ribosomal subunit, and Protein export | 70 | 1.49368 | top of list | 0.0013 | ks | EEF1A1,EEF2,EIF1,EIF2S3,EIF4B,FAU,GNB2L1,PABPC1,RPL10,RPL10A,RPL11,RPL12,RPL13,RPL14,RPL15,RPL18,RPL18A,RPL19,RPL21,RPL22,RPL22L1,RPL23A,RPL26,RPL27,RPL29,RPL3,RPL30,RPL32,RPL34,RPL35A,RPL36,RPL36A,RPL36AL,RPL37,RPL37A,RPL39,RPL4,RPL5,RPL6,RPL7A,RPL8,RPL9,RPLP0,RPLP1,RPLP2,RPS12,RPS13,RPS14,RPS15,RPS15A,RPS18,RPS19,RPS20,RPS21,RPS23,RPS24,RPS27,RPS27A,RPS28,RPS3,RPS3A,RPS4X,RPS5,RPS6,RPS7,RPS8,RPS9,RPSA,SPCS2,SSR2 |
| Cluster19 | CL:14966 | GTP hydrolysis and joining of the 60S ribosomal subunit, and Protein export | 73 | 1.48784 | top of list | 0.0013 | ks | EEF1A1,EEF1B2,EEF1D,EEF2,EIF1,EIF2S3,EIF4B,FAU,GNB2L1,PABPC1,RPL10,RPL10A,RPL11,RPL12,RPL13,RPL14,RPL15,RPL18,RPL18A,RPL19,RPL21,RPL22,RPL22L1,RPL23A,RPL26,RPL27,RPL29,RPL3,RPL30,RPL32,RPL34,RPL35A,RPL36,RPL36A,RPL36AL,RPL37,RPL37A,RPL39,RPL4,RPL5,RPL6,RPL7A,RPL8,RPL9,RPLP0,RPLP1,RPLP2,RPS12,RPS13,RPS14,RPS15,RPS15A,RPS18,RPS19,RPS20,RPS21,RPS23,RPS24,RPS27,RPS27A,RPS28,RPS3,RPS3A,RPS4X,RPS5,RPS6,RPS7,RPS8,RPS9,RPSA,SPCS2,SSR2,TPT1 |
| Cluster19 | CL:14976 | Peptide chain elongation | 62 | 1.62064 | top of list | 0.0013 | ks | EEF1A1,EEF2,FAU,GNB2L1,RPL10,RPL10A,RPL11,RPL12,RPL13,RPL14,RPL15,RPL18,RPL18A,RPL19,RPL21,RPL22,RPL22L1,RPL23A,RPL26,RPL27,RPL29,RPL3,RPL30,RPL32,RPL34,RPL35A,RPL36,RPL36A,RPL36AL,RPL37,RPL37A,RPL39,RPL4,RPL5,RPL6,RPL7A,RPL8,RPL9,RPLP0,RPLP1,RPLP2,RPS12,RPS13,RPS14,RPS15,RPS15A,RPS18,RPS19,RPS20,RPS21,RPS23,RPS24,RPS27,RPS27A,RPS28,RPS3A,RPS4X,RPS5,RPS6,RPS7,RPS8,RPS9 |
| Cluster19 | CL:14982 | Peptide chain elongation | 54 | 1.67814 | top of list | 0.0013 | ks | EEF2,FAU,RPL10,RPL10A,RPL11,RPL12,RPL13,RPL14,RPL15,RPL18A,RPL19,RPL21,RPL22,RPL23A,RPL26,RPL27,RPL29,RPL3,RPL32,RPL35A,RPL36,RPL36A,RPL37,RPL37A,RPL39,RPL5,RPL6,RPL7A,RPL8,RPL9,RPLP0,RPLP1,RPLP2,RPS12,RPS13,RPS14,RPS15,RPS15A,RPS18,RPS19,RPS20,RPS21,RPS23,RPS24,RPS27,RPS27A,RPS28,RPS3A,RPS4X,RPS5,RPS6,RPS7,RPS8,RPS9 |
| Cluster19 | CL:14978 | Peptide chain elongation | 60 | 1.67607 | top of list | 0.0013 | ks | EEF1A1,EEF2,FAU,GNB2L1,RPL10,RPL10A,RPL11,RPL12,RPL13,RPL14,RPL15,RPL18,RPL18A,RPL19,RPL21,RPL22,RPL22L1,RPL23A,RPL26,RPL27,RPL29,RPL3,RPL32,RPL34,RPL35A,RPL36,RPL36A,RPL37,RPL37A,RPL39,RPL4,RPL5,RPL6,RPL7A,RPL8,RPL9,RPLP0,RPLP1,RPLP2,RPS12,RPS13,RPS14,RPS15,RPS15A,RPS18,RPS19,RPS20,RPS21,RPS23,RPS24,RPS27,RPS27A,RPS28,RPS3A,RPS4X,RPS5,RPS6,RPS7,RPS8,RPS9 |
| Cluster19 | CL:14980 | Peptide chain elongation | 55 | 1.72686 | top of list | 0.0013 | ks | EEF2,FAU,RPL10,RPL10A,RPL11,RPL12,RPL13,RPL14,RPL15,RPL18,RPL18A,RPL19,RPL21,RPL22,RPL23A,RPL26,RPL27,RPL29,RPL3,RPL32,RPL35A,RPL36,RPL36A,RPL37,RPL37A,RPL39,RPL5,RPL6,RPL7A,RPL8,RPL9,RPLP0,RPLP1,RPLP2,RPS12,RPS13,RPS14,RPS15,RPS15A,RPS18,RPS19,RPS20,RPS21,RPS23,RPS24,RPS27,RPS27A,RPS28,RPS3A,RPS4X,RPS5,RPS6,RPS7,RPS8,RPS9 |
| Cluster19 | CL:14985 | Viral mRNA Translation | 42 | 1.73617 | top of list | 0.0014 | ks | FAU,RPL10A,RPL11,RPL12,RPL13,RPL14,RPL15,RPL18A,RPL19,RPL21,RPL22,RPL23A,RPL26,RPL27,RPL29,RPL3,RPL35A,RPL36,RPL37A,RPL39,RPL6,RPL7A,RPL8,RPL9,RPS12,RPS13,RPS14,RPS15,RPS15A,RPS18,RPS19,RPS21,RPS23,RPS27A,RPS28,RPS3A,RPS4X,RPS5,RPS6,RPS7,RPS8,RPS9 |
| Cluster19 | CL:14965 | GTP hydrolysis and joining of the 60S ribosomal subunit, and Nonsense-mediated mRNA decay | 74 | 1.46098 | top of list | 0.0014 | ks | EEF1A1,EEF1B2,EEF1D,EEF2,EIF1,EIF2S3,EIF4B,FAU,GNB2L1,PABPC1,PNRC2,RPL10,RPL10A,RPL11,RPL12,RPL13,RPL14,RPL15,RPL18,RPL18A,RPL19,RPL21,RPL22,RPL22L1,RPL23A,RPL26,RPL27,RPL29,RPL3,RPL30,RPL32,RPL34,RPL35A,RPL36,RPL36A,RPL36AL,RPL37,RPL37A,RPL39,RPL4,RPL5,RPL6,RPL7A,RPL8,RPL9,RPLP0,RPLP1,RPLP2,RPS12,RPS13,RPS14,RPS15,RPS15A,RPS18,RPS19,RPS20,RPS21,RPS23,RPS24,RPS27,RPS27A,RPS28,RPS3,RPS3A,RPS4X,RPS5,RPS6,RPS7,RPS8,RPS9,RPSA,SPCS2,SSR2,TPT1 |
| Cluster19 | CL:14968 | SRP-dependent cotranslational protein targeting to membrane | 64 | 1.49458 | top of list | 0.0017 | ks | EEF1A1,EEF2,FAU,GNB2L1,RPL10,RPL10A,RPL11,RPL12,RPL13,RPL14,RPL15,RPL18,RPL18A,RPL19,RPL21,RPL22,RPL22L1,RPL23A,RPL26,RPL27,RPL29,RPL3,RPL30,RPL32,RPL34,RPL35A,RPL36,RPL36A,RPL36AL,RPL37,RPL37A,RPL39,RPL4,RPL5,RPL6,RPL7A,RPL8,RPL9,RPLP0,RPLP1,RPLP2,RPS12,RPS13,RPS14,RPS15,RPS15A,RPS18,RPS19,RPS20,RPS21,RPS23,RPS24,RPS27,RPS27A,RPS28,RPS3A,RPS4X,RPS5,RPS6,RPS7,RPS8,RPS9,SPCS2,SSR2 |
| Cluster19 | CL:14983 | Peptide chain elongation | 47 | 1.71852 | top of list | 0.0017 | ks | EEF2,FAU,RPL10A,RPL11,RPL12,RPL13,RPL14,RPL15,RPL18A,RPL19,RPL21,RPL22,RPL23A,RPL26,RPL27,RPL29,RPL3,RPL32,RPL35A,RPL36,RPL36A,RPL37,RPL37A,RPL39,RPL6,RPL7A,RPL8,RPL9,RPLP2,RPS12,RPS13,RPS14,RPS15,RPS15A,RPS18,RPS19,RPS21,RPS23,RPS27A,RPS28,RPS3A,RPS4X,RPS5,RPS6,RPS7,RPS8,RPS9 |
| Cluster22 | CL:14976 | Peptide chain elongation | 76 | 2.07547 | top of list | 2.06E-08 | ks | EEF1A1,EEF2,FAU,GNB2L1,RPL10,RPL10A,RPL11,RPL12,RPL13,RPL14,RPL15,RPL17,RPL18,RPL18A,RPL19,RPL21,RPL22,RPL22L1,RPL23A,RPL24,RPL26,RPL27,RPL28,RPL29,RPL3,RPL30,RPL31,RPL32,RPL34,RPL35,RPL35A,RPL36,RPL36A,RPL36AL,RPL37,RPL37A,RPL38,RPL39,RPL4,RPL5,RPL6,RPL7,RPL7A,RPL8,RPL9,RPLP0,RPLP1,RPLP2,RPS12,RPS13,RPS14,RPS15,RPS15A,RPS16,RPS17,RPS18,RPS19,RPS21,RPS23,RPS24,RPS25,RPS26,RPS27,RPS27A,RPS27L,RPS28,RPS29,RPS3A,RPS4X,RPS5,RPS6,RPS7,RPS8,RPS9,SEC61G,UBA52 |
| Cluster22 | CL:14968 | SRP-dependent cotranslational protein targeting to membrane | 89 | 1.92319 | top of list | 2.06E-08 | ks | EEF1A1,EEF2,FAU,GNB2L1,RPL10,RPL10A,RPL11,RPL12,RPL13,RPL14,RPL15,RPL17,RPL18,RPL18A,RPL19,RPL21,RPL22,RPL22L1,RPL23A,RPL24,RPL26,RPL27,RPL28,RPL29,RPL3,RPL30,RPL31,RPL32,RPL34,RPL35,RPL35A,RPL36,RPL36A,RPL36AL,RPL37,RPL37A,RPL38,RPL39,RPL4,RPL5,RPL6,RPL7,RPL7A,RPL8,RPL9,RPLP0,RPLP1,RPLP2,RPS12,RPS13,RPS14,RPS15,RPS15A,RPS16,RPS17,RPS18,RPS19,RPS21,RPS23,RPS24,RPS25,RPS26,RPS27,RPS27A,RPS27L,RPS28,RPS29,RPS3A,RPS4X,RPS5,RPS6,RPS7,RPS8,RPS9,SEC11A,SEC11C,SEC61G,SPCS1,SPCS2,SPCS3,SRP54,SRP72,SRPR,SSR1,SSR2,SSR3,SSR4,TRAM1,UBA52 |
| Cluster22 | CL:14978 | Peptide chain elongation | 73 | 2.11924 | top of list | 2.96E-08 | ks | EEF1A1,EEF2,FAU,GNB2L1,RPL10,RPL10A,RPL11,RPL12,RPL13,RPL14,RPL15,RPL17,RPL18,RPL18A,RPL19,RPL21,RPL22,RPL22L1,RPL23A,RPL24,RPL26,RPL27,RPL28,RPL29,RPL3,RPL31,RPL32,RPL34,RPL35,RPL35A,RPL36,RPL36A,RPL37,RPL37A,RPL38,RPL39,RPL4,RPL5,RPL6,RPL7,RPL7A,RPL8,RPL9,RPLP0,RPLP1,RPLP2,RPS12,RPS13,RPS14,RPS15,RPS15A,RPS16,RPS17,RPS18,RPS19,RPS21,RPS23,RPS24,RPS25,RPS26,RPS27,RPS27A,RPS27L,RPS28,RPS29,RPS3A,RPS4X,RPS5,RPS6,RPS7,RPS8,RPS9,UBA52 |
| Cluster22 | CL:14980 | Peptide chain elongation | 68 | 2.09871 | top of list | 3.88E-08 | ks | EEF2,FAU,RPL10,RPL10A,RPL11,RPL12,RPL13,RPL14,RPL15,RPL17,RPL18,RPL18A,RPL19,RPL21,RPL22,RPL23A,RPL24,RPL26,RPL27,RPL28,RPL29,RPL3,RPL31,RPL32,RPL35,RPL35A,RPL36,RPL36A,RPL37,RPL37A,RPL38,RPL39,RPL5,RPL6,RPL7,RPL7A,RPL8,RPL9,RPLP0,RPLP1,RPLP2,RPS12,RPS13,RPS14,RPS15,RPS15A,RPS16,RPS17,RPS18,RPS19,RPS21,RPS23,RPS24,RPS25,RPS26,RPS27,RPS27A,RPS27L,RPS28,RPS29,RPS3A,RPS4X,RPS5,RPS6,RPS7,RPS8,RPS9,UBA52 |
| Cluster22 | CL:14982 | Peptide chain elongation | 66 | 2.1341 | top of list | 3.88E-08 | ks | EEF2,FAU,RPL10,RPL10A,RPL11,RPL12,RPL13,RPL14,RPL15,RPL17,RPL18A,RPL19,RPL21,RPL22,RPL23A,RPL24,RPL26,RPL27,RPL28,RPL29,RPL3,RPL31,RPL32,RPL35,RPL35A,RPL36,RPL36A,RPL37,RPL37A,RPL38,RPL39,RPL5,RPL6,RPL7,RPL7A,RPL8,RPL9,RPLP0,RPLP1,RPLP2,RPS12,RPS13,RPS14,RPS15,RPS15A,RPS16,RPS17,RPS18,RPS19,RPS21,RPS23,RPS24,RPS25,RPS26,RPS27,RPS27A,RPS28,RPS29,RPS3A,RPS4X,RPS5,RPS6,RPS7,RPS8,RPS9,UBA52 |
| Cluster22 | CL:14963 | GTP hydrolysis and joining of the 60S ribosomal subunit, and Nonsense-mediated mRNA decay | 105 | 1.70813 | top of list | 5.02E-08 | ks | BTF3,EEF1A1,EEF1B2,EEF1D,EEF2,EIF1,EIF2S2,EIF3A,EIF3F,EIF3K,EIF3L,EIF4G1,FAU,GNB2L1,GSPT1,NACA,PABPC1,RPL10,RPL10A,RPL11,RPL12,RPL13,RPL14,RPL15,RPL17,RPL18,RPL18A,RPL19,RPL21,RPL22,RPL22L1,RPL23A,RPL24,RPL26,RPL27,RPL28,RPL29,RPL3,RPL30,RPL31,RPL32,RPL34,RPL35,RPL35A,RPL36,RPL36A,RPL36AL,RPL37,RPL37A,RPL38,RPL39,RPL4,RPL5,RPL6,RPL7,RPL7A,RPL8,RPL9,RPLP0,RPLP1,RPLP2,RPS12,RPS13,RPS14,RPS15,RPS15A,RPS16,RPS17,RPS18,RPS19,RPS21,RPS23,RPS24,RPS25,RPS26,RPS27,RPS27A,RPS27L,RPS28,RPS29,RPS3,RPS3A,RPS4X,RPS5,RPS6,RPS7,RPS8,RPS9,RPSA,SEC11A,SEC11C,SEC61G,SPCS1,SPCS2,SPCS3,SRP54,SRP72,SRPR,SSR1,SSR2,SSR3,SSR4,TPT1,TRAM1,UBA52 |
| **Cluster** | **#term ID** | **term description** | **genes mapped** | **enrichment score** | **direction** | **false discovery rate** | **method** | **matching proteins in your input (labels)** |
| Cluster22 | CL:14973 | Peptide chain elongation | 79 | 1.94478 | top of list | 5.02E-08 | ks | EEF1A1,EEF2,FAU,GNB2L1,RPL10,RPL10A,RPL11,RPL12,RPL13,RPL14,RPL15,RPL17,RPL18,RPL18A,RPL19,RPL21,RPL22,RPL22L1,RPL23A,RPL24,RPL26,RPL27,RPL28,RPL29,RPL3,RPL30,RPL31,RPL32,RPL34,RPL35,RPL35A,RPL36,RPL36A,RPL36AL,RPL37,RPL37A,RPL38,RPL39,RPL4,RPL5,RPL6,RPL7,RPL7A,RPL8,RPL9,RPLP0,RPLP1,RPLP2,RPS12,RPS13,RPS14,RPS15,RPS15A,RPS16,RPS17,RPS18,RPS19,RPS21,RPS23,RPS24,RPS25,RPS26,RPS27,RPS27A,RPS27L,RPS28,RPS29,RPS3A,RPS4X,RPS5,RPS6,RPS7,RPS8,RPS9,SEC61G,SRP54,SRP72,SRPR,UBA52 |
| Cluster22 | CL:14967 | GTP hydrolysis and joining of the 60S ribosomal subunit, and Protein export | 99 | 1.74234 | top of list | 6.06E-08 | ks | EEF1A1,EEF2,EIF1,EIF2S2,EIF3A,EIF3F,EIF3K,EIF3L,EIF4G1,FAU,GNB2L1,PABPC1,RPL10,RPL10A,RPL11,RPL12,RPL13,RPL14,RPL15,RPL17,RPL18,RPL18A,RPL19,RPL21,RPL22,RPL22L1,RPL23A,RPL24,RPL26,RPL27,RPL28,RPL29,RPL3,RPL30,RPL31,RPL32,RPL34,RPL35,RPL35A,RPL36,RPL36A,RPL36AL,RPL37,RPL37A,RPL38,RPL39,RPL4,RPL5,RPL6,RPL7,RPL7A,RPL8,RPL9,RPLP0,RPLP1,RPLP2,RPS12,RPS13,RPS14,RPS15,RPS15A,RPS16,RPS17,RPS18,RPS19,RPS21,RPS23,RPS24,RPS25,RPS26,RPS27,RPS27A,RPS27L,RPS28,RPS29,RPS3,RPS3A,RPS4X,RPS5,RPS6,RPS7,RPS8,RPS9,RPSA,SEC11A,SEC11C,SEC61G,SPCS1,SPCS2,SPCS3,SRP54,SRP72,SRPR,SSR1,SSR2,SSR3,SSR4,TRAM1,UBA52 |
| Cluster22 | CL:14966 | GTP hydrolysis and joining of the 60S ribosomal subunit, and Protein export | 102 | 1.71682 | top of list | 6.23E-08 | ks | EEF1A1,EEF1B2,EEF1D,EEF2,EIF1,EIF2S2,EIF3A,EIF3F,EIF3K,EIF3L,EIF4G1,FAU,GNB2L1,PABPC1,RPL10,RPL10A,RPL11,RPL12,RPL13,RPL14,RPL15,RPL17,RPL18,RPL18A,RPL19,RPL21,RPL22,RPL22L1,RPL23A,RPL24,RPL26,RPL27,RPL28,RPL29,RPL3,RPL30,RPL31,RPL32,RPL34,RPL35,RPL35A,RPL36,RPL36A,RPL36AL,RPL37,RPL37A,RPL38,RPL39,RPL4,RPL5,RPL6,RPL7,RPL7A,RPL8,RPL9,RPLP0,RPLP1,RPLP2,RPS12,RPS13,RPS14,RPS15,RPS15A,RPS16,RPS17,RPS18,RPS19,RPS21,RPS23,RPS24,RPS25,RPS26,RPS27,RPS27A,RPS27L,RPS28,RPS29,RPS3,RPS3A,RPS4X,RPS5,RPS6,RPS7,RPS8,RPS9,RPSA,SEC11A,SEC11C,SEC61G,SPCS1,SPCS2,SPCS3,SRP54,SRP72,SRPR,SSR1,SSR2,SSR3,SSR4,TPT1,TRAM1,UBA52 |
| Cluster22 | CL:14965 | GTP hydrolysis and joining of the 60S ribosomal subunit, and Nonsense-mediated mRNA decay | 103 | 1.69139 | top of list | 1.06E-07 | ks | EEF1A1,EEF1B2,EEF1D,EEF2,EIF1,EIF2S2,EIF3A,EIF3F,EIF3K,EIF3L,EIF4G1,FAU,GNB2L1,GSPT1,PABPC1,RPL10,RPL10A,RPL11,RPL12,RPL13,RPL14,RPL15,RPL17,RPL18,RPL18A,RPL19,RPL21,RPL22,RPL22L1,RPL23A,RPL24,RPL26,RPL27,RPL28,RPL29,RPL3,RPL30,RPL31,RPL32,RPL34,RPL35,RPL35A,RPL36,RPL36A,RPL36AL,RPL37,RPL37A,RPL38,RPL39,RPL4,RPL5,RPL6,RPL7,RPL7A,RPL8,RPL9,RPLP0,RPLP1,RPLP2,RPS12,RPS13,RPS14,RPS15,RPS15A,RPS16,RPS17,RPS18,RPS19,RPS21,RPS23,RPS24,RPS25,RPS26,RPS27,RPS27A,RPS27L,RPS28,RPS29,RPS3,RPS3A,RPS4X,RPS5,RPS6,RPS7,RPS8,RPS9,RPSA,SEC11A,SEC11C,SEC61G,SPCS1,SPCS2,SPCS3,SRP54,SRP72,SRPR,SSR1,SSR2,SSR3,SSR4,TPT1,TRAM1,UBA52 |
| Cluster22 | CL:14983 | Peptide chain elongation | 57 | 2.04127 | top of list | 1.88E-07 | ks | EEF2,FAU,RPL10A,RPL11,RPL12,RPL13,RPL14,RPL15,RPL17,RPL18A,RPL19,RPL21,RPL22,RPL23A,RPL24,RPL26,RPL27,RPL29,RPL3,RPL32,RPL35,RPL35A,RPL36,RPL36A,RPL37,RPL37A,RPL38,RPL39,RPL6,RPL7,RPL7A,RPL8,RPL9,RPLP2,RPS12,RPS13,RPS14,RPS15,RPS15A,RPS16,RPS17,RPS18,RPS19,RPS21,RPS23,RPS25,RPS26,RPS27A,RPS28,RPS3A,RPS4X,RPS5,RPS6,RPS7,RPS8,RPS9,UBA52 |
| Cluster22 | CL:14985 | Viral mRNA Translation | 52 | 2.03856 | top of list | 8.23E-07 | ks | FAU,RPL10A,RPL11,RPL12,RPL13,RPL14,RPL15,RPL17,RPL18A,RPL19,RPL21,RPL22,RPL23A,RPL24,RPL26,RPL27,RPL29,RPL3,RPL35,RPL35A,RPL36,RPL37A,RPL38,RPL39,RPL6,RPL7,RPL7A,RPL8,RPL9,RPS12,RPS13,RPS14,RPS15,RPS15A,RPS16,RPS17,RPS18,RPS19,RPS21,RPS23,RPS25,RPS26,RPS27A,RPS28,RPS3A,RPS4X,RPS5,RPS6,RPS7,RPS8,RPS9,UBA52 |
| Cluster22 | CL:2403 | mixed, incl. MAPK targets/ Nuclear events mediated by MAP kinases, and bZIP Maf transcription factor | 4 | 5.84633 | bottom of list | 0.00014 | afc | DUSP1,FOS,JUN,JUNB |
| Cluster22 | CL:2190 | mixed, incl. regulation of TOR signaling, and MAPK targets/ Nuclear events mediated by MAP kinases | 8 | 3.34724 | bottom of list | 0.0007 | afc | CDC25B,DUSP1,FOS,JUN,JUNB,YWHAB,YWHAE,YWHAZ |
| Cluster22 | CL:14161 | endoplasmic reticulum chaperone complex, and Unfolded protein response | 15 | 2.85524 | top of list | 0.0009 | afc | CALR,CRELD2,DNAJB11,DNAJC3,EDEM2,HERPUD1,HSP90B1,HSPA5,HYOU1,MANF,P4HB,PDIA4,PDIA6,SDF2L1,XBP1 |
| Cluster22 | CL:14160 | mixed, incl. Unfolded protein response, and endoplasmic reticulum chaperone complex | 19 | 2.36997 | top of list | 0.0017 | afc | CALR,CRELD2,DNAJB11,DNAJB9,DNAJC3,EDEM2,HERPUD1,HSP90B1,HSPA5,HYOU1,MANF,P4HB,PDIA4,PDIA6,SDF2L1,SEC61A1,SEC61B,SERP1,XBP1 |
| Cluster22 | CL:14156 | mixed, incl. Unfolded Protein Response (UPR), and Thioredoxin, conserved site | 23 | 2.23284 | top of list | 0.0036 | ks | CALR,CANX,CRELD2,DNAJB11,DNAJB9,DNAJC3,EDEM2,HERPUD1,HSP90B1,HSPA5,HYOU1,MANF,P4HB,PDIA3,PDIA4,PDIA6,SDF2L1,SEC61A1,SEC61B,SERP1,TAP1,TXNDC11,XBP1 |
| Cluster22 | CL:14159 | mixed, incl. Unfolded protein response, and endoplasmic reticulum chaperone complex | 22 | 2.29358 | top of list | 0.0075 | ks | CALR,CANX,CRELD2,DNAJB11,DNAJB9,DNAJC3,EDEM2,HERPUD1,HSP90B1,HSPA5,HYOU1,MANF,P4HB,PDIA3,PDIA4,PDIA6,SDF2L1,SEC61A1,SEC61B,SERP1,TAP1,XBP1 |
| Cluster23 | CL:4209 | mixed, incl. Immunoregulatory interactions between a Lymphoid and a non-Lymphoid cell, and T cell costimulation | 14 | 3.05065 | top of list | 0.0031 | afc | B2M,CD7,GNLY,GZMB,GZMH,HCST,HLA-A,HLA-C,HLA-E,HLA-F,KLRB1,KLRD1,NKG7,PRF1 |
| Cluster23 | CL:4212 | Immunoregulatory interactions between a Lymphoid and a non-Lymphoid cell, and Cytolysis | 13 | 3.22557 | top of list | 0.0031 | afc | B2M,GNLY,GZMB,GZMH,HCST,HLA-A,HLA-C,HLA-E,HLA-F,KLRB1,KLRD1,NKG7,PRF1 |
| Cluster24 | CL:2403 | mixed, incl. MAPK targets/ Nuclear events mediated by MAP kinases, and bZIP Maf transcription factor | 3 | 8.46937 | bottom of list | 0.00062 | afc | DUSP1,FOS,JUN |
| Cluster24 | CL:22327 | Oxidative phosphorylation | 9 | 4.09987 | top of list | 0.0086 | afc | MT-ATP6,MT-ATP8,MT-CO1,MT-CO2,MT-CO3,MT-CYB,MT-ND4,MT-ND4L,MT-ND5 |
| Cluster25 | CL:22327 | Oxidative phosphorylation | 11 | 4.75372 | top of list | 0.0003 | afc | COX4I1,MT-ATP6,MT-ATP8,MT-CO1,MT-CO2,MT-CO3,MT-CYB,MT-ND1,MT-ND4,MT-ND4L,MT-ND5 |
| Cluster25 | CL:22328 | respirasome | 10 | 4.47091 | top of list | 0.001 | afc | COX4I1,MT-ATP6,MT-CO1,MT-CO2,MT-CO3,MT-CYB,MT-ND1,MT-ND4,MT-ND4L,MT-ND5 |
| **Cluster** | **#term ID** | **term description** | **genes mapped** | **enrichment score** | **direction** | **false discovery rate** | **method** | **matching proteins in your input (labels)** |
| Cluster25 | CL:22502 | Leber hereditary optic neuropathy | 5 | 6.01529 | top of list | 0.0024 | afc | MT-ATP6,MT-CO1,MT-CO3,MT-CYB,MT-ND4 |
| Cluster25 | CL:22446 | cytochrome complex, and Cytochrome c oxidase subunit VII | 7 | 4.59933 | top of list | 0.0039 | afc | COX4I1,MT-ATP6,MT-CO1,MT-CO2,MT-CO3,MT-CYB,MT-ND4 |
| Cluster27 | CL:14966 | GTP hydrolysis and joining of the 60S ribosomal subunit, and Protein export | 77 | 1.64922 | top of list | 0.00087 | ks | EEF1A1,EEF1B2,EEF1D,EEF2,EIF1,EIF3F,EIF3H,EIF3L,EIF4B,FAU,GNB2L1,PABPC1,RPL10,RPL10A,RPL11,RPL12,RPL13,RPL14,RPL15,RPL17,RPL18,RPL18A,RPL19,RPL21,RPL22,RPL23A,RPL24,RPL26,RPL27,RPL28,RPL29,RPL3,RPL30,RPL31,RPL32,RPL34,RPL35,RPL35A,RPL36,RPL36A,RPL37,RPL4,RPL5,RPL6,RPL7,RPL7A,RPL8,RPL9,RPLP0,RPLP1,RPS12,RPS13,RPS14,RPS15,RPS15A,RPS16,RPS18,RPS19,RPS21,RPS23,RPS24,RPS25,RPS26,RPS27,RPS27A,RPS28,RPS3,RPS3A,RPS4X,RPS5,RPS6,RPS7,RPS8,RPS9,RPSA,TPT1,TRAM1 |
| Cluster27 | CL:14968 | SRP-dependent cotranslational protein targeting to membrane | 66 | 1.7282 | top of list | 0.00087 | ks | EEF1A1,EEF2,FAU,GNB2L1,RPL10,RPL10A,RPL11,RPL12,RPL13,RPL14,RPL15,RPL17,RPL18,RPL18A,RPL19,RPL21,RPL22,RPL23A,RPL24,RPL26,RPL27,RPL28,RPL29,RPL3,RPL30,RPL31,RPL32,RPL34,RPL35,RPL35A,RPL36,RPL36A,RPL37,RPL4,RPL5,RPL6,RPL7,RPL7A,RPL8,RPL9,RPLP0,RPLP1,RPS12,RPS13,RPS14,RPS15,RPS15A,RPS16,RPS18,RPS19,RPS21,RPS23,RPS24,RPS25,RPS26,RPS27,RPS27A,RPS28,RPS3A,RPS4X,RPS5,RPS6,RPS7,RPS8,RPS9,TRAM1 |
| Cluster27 | CL:14967 | GTP hydrolysis and joining of the 60S ribosomal subunit, and Protein export | 74 | 1.66636 | top of list | 0.00087 | ks | EEF1A1,EEF2,EIF1,EIF3F,EIF3H,EIF3L,EIF4B,FAU,GNB2L1,PABPC1,RPL10,RPL10A,RPL11,RPL12,RPL13,RPL14,RPL15,RPL17,RPL18,RPL18A,RPL19,RPL21,RPL22,RPL23A,RPL24,RPL26,RPL27,RPL28,RPL29,RPL3,RPL30,RPL31,RPL32,RPL34,RPL35,RPL35A,RPL36,RPL36A,RPL37,RPL4,RPL5,RPL6,RPL7,RPL7A,RPL8,RPL9,RPLP0,RPLP1,RPS12,RPS13,RPS14,RPS15,RPS15A,RPS16,RPS18,RPS19,RPS21,RPS23,RPS24,RPS25,RPS26,RPS27,RPS27A,RPS28,RPS3,RPS3A,RPS4X,RPS5,RPS6,RPS7,RPS8,RPS9,RPSA,TRAM1 |
| Cluster27 | CL:14963 | GTP hydrolysis and joining of the 60S ribosomal subunit, and Nonsense-mediated mRNA decay | 79 | 1.69672 | top of list | 0.00087 | ks | BTF3,EEF1A1,EEF1B2,EEF1D,EEF2,EIF1,EIF3F,EIF3H,EIF3L,EIF4B,FAU,GNB2L1,NACA,PABPC1,RPL10,RPL10A,RPL11,RPL12,RPL13,RPL14,RPL15,RPL17,RPL18,RPL18A,RPL19,RPL21,RPL22,RPL23A,RPL24,RPL26,RPL27,RPL28,RPL29,RPL3,RPL30,RPL31,RPL32,RPL34,RPL35,RPL35A,RPL36,RPL36A,RPL37,RPL4,RPL5,RPL6,RPL7,RPL7A,RPL8,RPL9,RPLP0,RPLP1,RPS12,RPS13,RPS14,RPS15,RPS15A,RPS16,RPS18,RPS19,RPS21,RPS23,RPS24,RPS25,RPS26,RPS27,RPS27A,RPS28,RPS3,RPS3A,RPS4X,RPS5,RPS6,RPS7,RPS8,RPS9,RPSA,TPT1,TRAM1 |
| Cluster27 | CL:14978 | Peptide chain elongation | 64 | 1.79844 | top of list | 0.00087 | ks | EEF1A1,EEF2,FAU,GNB2L1,RPL10,RPL10A,RPL11,RPL12,RPL13,RPL14,RPL15,RPL17,RPL18,RPL18A,RPL19,RPL21,RPL22,RPL23A,RPL24,RPL26,RPL27,RPL28,RPL29,RPL3,RPL31,RPL32,RPL34,RPL35,RPL35A,RPL36,RPL36A,RPL37,RPL4,RPL5,RPL6,RPL7,RPL7A,RPL8,RPL9,RPLP0,RPLP1,RPS12,RPS13,RPS14,RPS15,RPS15A,RPS16,RPS18,RPS19,RPS21,RPS23,RPS24,RPS25,RPS26,RPS27,RPS27A,RPS28,RPS3A,RPS4X,RPS5,RPS6,RPS7,RPS8,RPS9 |
| Cluster27 | CL:14976 | Peptide chain elongation | 65 | 1.79347 | top of list | 0.00087 | ks | EEF1A1,EEF2,FAU,GNB2L1,RPL10,RPL10A,RPL11,RPL12,RPL13,RPL14,RPL15,RPL17,RPL18,RPL18A,RPL19,RPL21,RPL22,RPL23A,RPL24,RPL26,RPL27,RPL28,RPL29,RPL3,RPL30,RPL31,RPL32,RPL34,RPL35,RPL35A,RPL36,RPL36A,RPL37,RPL4,RPL5,RPL6,RPL7,RPL7A,RPL8,RPL9,RPLP0,RPLP1,RPS12,RPS13,RPS14,RPS15,RPS15A,RPS16,RPS18,RPS19,RPS21,RPS23,RPS24,RPS25,RPS26,RPS27,RPS27A,RPS28,RPS3A,RPS4X,RPS5,RPS6,RPS7,RPS8,RPS9 |
| Cluster27 | CL:14980 | Peptide chain elongation | 60 | 1.70764 | top of list | 0.0012 | ks | EEF2,FAU,RPL10,RPL10A,RPL11,RPL12,RPL13,RPL14,RPL15,RPL17,RPL18,RPL18A,RPL19,RPL21,RPL22,RPL23A,RPL24,RPL26,RPL27,RPL28,RPL29,RPL3,RPL31,RPL32,RPL35,RPL35A,RPL36,RPL36A,RPL37,RPL5,RPL6,RPL7,RPL7A,RPL8,RPL9,RPLP0,RPLP1,RPS12,RPS13,RPS14,RPS15,RPS15A,RPS16,RPS18,RPS19,RPS21,RPS23,RPS24,RPS25,RPS26,RPS27,RPS27A,RPS28,RPS3A,RPS4X,RPS5,RPS6,RPS7,RPS8,RPS9 |
| Cluster27 | CL:14982 | Peptide chain elongation | 59 | 1.69744 | top of list | 0.0013 | ks | EEF2,FAU,RPL10,RPL10A,RPL11,RPL12,RPL13,RPL14,RPL15,RPL17,RPL18A,RPL19,RPL21,RPL22,RPL23A,RPL24,RPL26,RPL27,RPL28,RPL29,RPL3,RPL31,RPL32,RPL35,RPL35A,RPL36,RPL36A,RPL37,RPL5,RPL6,RPL7,RPL7A,RPL8,RPL9,RPLP0,RPLP1,RPS12,RPS13,RPS14,RPS15,RPS15A,RPS16,RPS18,RPS19,RPS21,RPS23,RPS24,RPS25,RPS26,RPS27,RPS27A,RPS28,RPS3A,RPS4X,RPS5,RPS6,RPS7,RPS8,RPS9 |
| Cluster27 | CL:14983 | Peptide chain elongation | 51 | 1.52632 | top of list | 0.0033 | ks | EEF2,FAU,RPL10A,RPL11,RPL12,RPL13,RPL14,RPL15,RPL17,RPL18A,RPL19,RPL21,RPL22,RPL23A,RPL24,RPL26,RPL27,RPL29,RPL3,RPL32,RPL35,RPL35A,RPL36,RPL36A,RPL37,RPL6,RPL7,RPL7A,RPL8,RPL9,RPS12,RPS13,RPS14,RPS15,RPS15A,RPS16,RPS18,RPS19,RPS21,RPS23,RPS25,RPS26,RPS27A,RPS28,RPS3A,RPS4X,RPS5,RPS6,RPS7,RPS8,RPS9 |
| Cluster27 | CL:14985 | Viral mRNA Translation | 47 | 1.38691 | top of list | 0.0095 | ks | FAU,RPL10A,RPL11,RPL12,RPL13,RPL14,RPL15,RPL17,RPL18A,RPL19,RPL21,RPL22,RPL23A,RPL24,RPL26,RPL27,RPL29,RPL3,RPL35,RPL35A,RPL36,RPL6,RPL7,RPL7A,RPL8,RPL9,RPS12,RPS13,RPS14,RPS15,RPS15A,RPS16,RPS18,RPS19,RPS21,RPS23,RPS25,RPS26,RPS27A,RPS28,RPS3A,RPS4X,RPS5,RPS6,RPS7,RPS8,RPS9 |
| Cluster28 | CL:18630 | MHC class II protein complex | 8 | 4.17559 | top of list | 0.0042 | afc | CD74,HLA-DMA,HLA-DMB,HLA-DPA1,HLA-DQA1,HLA-DRA,HLA-DRB1,HLA-DRB5 |
| Cluster29 | CL:18630 | MHC class II protein complex | 9 | 5.4361 | top of list | 8.53E-07 | afc | CD74,HLA-DMA,HLA-DPA1,HLA-DPB1,HLA-DQA1,HLA-DQB1,HLA-DRA,HLA-DRB1,HLA-DRB5 |
| Cluster29 | CL:18627 | MHC class II protein complex, and dynactin complex | 10 | 4.8633 | top of list | 8.53E-07 | afc | CD74,HLA-DMA,HLA-DOA,HLA-DPA1,HLA-DPB1,HLA-DQA1,HLA-DQB1,HLA-DRA,HLA-DRB1,HLA-DRB5 |
| Cluster29 | CL:18626 | MHC class II protein complex, and dynactin complex | 11 | 4.3703 | top of list | 8.53E-07 | afc | CAPZB,CD74,HLA-DMA,HLA-DOA,HLA-DPA1,HLA-DPB1,HLA-DQA1,HLA-DQB1,HLA-DRA,HLA-DRB1,HLA-DRB5 |
| Cluster29 | CL:18498 | Golgi-to-ER retrograde transport, and MHC class II protein complex | 12 | 3.86337 | top of list | 9.60E-06 | afc | ARF3,CAPZB,CD74,HLA-DMA,HLA-DOA,HLA-DPA1,HLA-DPB1,HLA-DQA1,HLA-DQB1,HLA-DRA,HLA-DRB1,HLA-DRB5 |
| Cluster29 | CL:14968 | SRP-dependent cotranslational protein targeting to membrane | 52 | 1.47367 | top of list | 4.00E-05 | ks | EEF2,FAU,GNB2L1,RPL10,RPL11,RPL12,RPL14,RPL15,RPL17,RPL18,RPL18A,RPL19,RPL22,RPL23A,RPL24,RPL26,RPL27,RPL28,RPL30,RPL31,RPL32,RPL34,RPL35,RPL35A,RPL36A,RPL36AL,RPL37,RPL39,RPL5,RPL6,RPL7A,RPL8,RPLP0,RPLP1,RPLP2,RPS12,RPS13,RPS14,RPS15,RPS15A,RPS18,RPS21,RPS23,RPS24,RPS26,RPS27A,RPS28,RPS4X,RPS7,RPS8,SEC11A,SRP14 |
| Cluster29 | CL:14973 | Peptide chain elongation | 51 | 1.50745 | top of list | 4.00E-05 | ks | EEF2,FAU,GNB2L1,RPL10,RPL11,RPL12,RPL14,RPL15,RPL17,RPL18,RPL18A,RPL19,RPL22,RPL23A,RPL24,RPL26,RPL27,RPL28,RPL30,RPL31,RPL32,RPL34,RPL35,RPL35A,RPL36A,RPL36AL,RPL37,RPL39,RPL5,RPL6,RPL7A,RPL8,RPLP0,RPLP1,RPLP2,RPS12,RPS13,RPS14,RPS15,RPS15A,RPS18,RPS21,RPS23,RPS24,RPS26,RPS27A,RPS28,RPS4X,RPS7,RPS8,SRP14 |
| Cluster29 | CL:14976 | Peptide chain elongation | 50 | 1.51926 | top of list | 6.02E-05 | ks | EEF2,FAU,GNB2L1,RPL10,RPL11,RPL12,RPL14,RPL15,RPL17,RPL18,RPL18A,RPL19,RPL22,RPL23A,RPL24,RPL26,RPL27,RPL28,RPL30,RPL31,RPL32,RPL34,RPL35,RPL35A,RPL36A,RPL36AL,RPL37,RPL39,RPL5,RPL6,RPL7A,RPL8,RPLP0,RPLP1,RPLP2,RPS12,RPS13,RPS14,RPS15,RPS15A,RPS18,RPS21,RPS23,RPS24,RPS26,RPS27A,RPS28,RPS4X,RPS7,RPS8 |
| Cluster29 | CL:14980 | Peptide chain elongation | 46 | 1.58097 | top of list | 7.45E-05 | ks | EEF2,FAU,RPL10,RPL11,RPL12,RPL14,RPL15,RPL17,RPL18,RPL18A,RPL19,RPL22,RPL23A,RPL24,RPL26,RPL27,RPL28,RPL31,RPL32,RPL35,RPL35A,RPL36A,RPL37,RPL39,RPL5,RPL6,RPL7A,RPL8,RPLP0,RPLP1,RPLP2,RPS12,RPS13,RPS14,RPS15,RPS15A,RPS18,RPS21,RPS23,RPS24,RPS26,RPS27A,RPS28,RPS4X,RPS7,RPS8 |
| Cluster29 | CL:14982 | Peptide chain elongation | 45 | 1.53949 | top of list | 9.85E-05 | ks | EEF2,FAU,RPL10,RPL11,RPL12,RPL14,RPL15,RPL17,RPL18A,RPL19,RPL22,RPL23A,RPL24,RPL26,RPL27,RPL28,RPL31,RPL32,RPL35,RPL35A,RPL36A,RPL37,RPL39,RPL5,RPL6,RPL7A,RPL8,RPLP0,RPLP1,RPLP2,RPS12,RPS13,RPS14,RPS15,RPS15A,RPS18,RPS21,RPS23,RPS24,RPS26,RPS27A,RPS28,RPS4X,RPS7,RPS8 |
| Cluster29 | CL:14978 | Peptide chain elongation | 48 | 1.49355 | top of list | 0.00011 | ks | EEF2,FAU,GNB2L1,RPL10,RPL11,RPL12,RPL14,RPL15,RPL17,RPL18,RPL18A,RPL19,RPL22,RPL23A,RPL24,RPL26,RPL27,RPL28,RPL31,RPL32,RPL34,RPL35,RPL35A,RPL36A,RPL37,RPL39,RPL5,RPL6,RPL7A,RPL8,RPLP0,RPLP1,RPLP2,RPS12,RPS13,RPS14,RPS15,RPS15A,RPS18,RPS21,RPS23,RPS24,RPS26,RPS27A,RPS28,RPS4X,RPS7,RPS8 |
| Cluster29 | CL:14967 | GTP hydrolysis and joining of the 60S ribosomal subunit, and Protein export | 59 | 1.28826 | top of list | 0.00012 | ks | ABCE1,EEF2,EIF1B,EIF2S2,EIF3K,EIF3M,EIF4G3,FAU,GNB2L1,RPL10,RPL11,RPL12,RPL14,RPL15,RPL17,RPL18,RPL18A,RPL19,RPL22,RPL23A,RPL24,RPL26,RPL27,RPL28,RPL30,RPL31,RPL32,RPL34,RPL35,RPL35A,RPL36A,RPL36AL,RPL37,RPL39,RPL5,RPL6,RPL7A,RPL8,RPLP0,RPLP1,RPLP2,RPS12,RPS13,RPS14,RPS15,RPS15A,RPS18,RPS21,RPS23,RPS24,RPS26,RPS27A,RPS28,RPS3,RPS4X,RPS7,RPS8,SEC11A,SRP14 |
| **Cluster** | **#term ID** | **term description** | **genes mapped** | **enrichment score** | **direction** | **false discovery rate** | **method** | **matching proteins in your input (labels)** |
| Cluster29 | CL:14963 | GTP hydrolysis and joining of the 60S ribosomal subunit, and Nonsense-mediated mRNA decay | 62 | 1.27596 | top of list | 0.00013 | ks | ABCE1,BTF3,EEF2,EIF1B,EIF2S2,EIF3K,EIF3M,EIF4G3,FAU,GNB2L1,NACA,RPL10,RPL11,RPL12,RPL14,RPL15,RPL17,RPL18,RPL18A,RPL19,RPL22,RPL23A,RPL24,RPL26,RPL27,RPL28,RPL30,RPL31,RPL32,RPL34,RPL35,RPL35A,RPL36A,RPL36AL,RPL37,RPL39,RPL5,RPL6,RPL7A,RPL8,RPLP0,RPLP1,RPLP2,RPS12,RPS13,RPS14,RPS15,RPS15A,RPS18,RPS21,RPS23,RPS24,RPS26,RPS27A,RPS28,RPS3,RPS4X,RPS7,RPS8,SEC11A,SRP14,TPT1 |
| Cluster29 | CL:14966 | GTP hydrolysis and joining of the 60S ribosomal subunit, and Protein export | 60 | 1.23893 | top of list | 0.00024 | ks | ABCE1,EEF2,EIF1B,EIF2S2,EIF3K,EIF3M,EIF4G3,FAU,GNB2L1,RPL10,RPL11,RPL12,RPL14,RPL15,RPL17,RPL18,RPL18A,RPL19,RPL22,RPL23A,RPL24,RPL26,RPL27,RPL28,RPL30,RPL31,RPL32,RPL34,RPL35,RPL35A,RPL36A,RPL36AL,RPL37,RPL39,RPL5,RPL6,RPL7A,RPL8,RPLP0,RPLP1,RPLP2,RPS12,RPS13,RPS14,RPS15,RPS15A,RPS18,RPS21,RPS23,RPS24,RPS26,RPS27A,RPS28,RPS3,RPS4X,RPS7,RPS8,SEC11A,SRP14,TPT1 |
| Cluster29 | CL:14983 | Peptide chain elongation | 38 | 1.42304 | top of list | 0.00086 | ks | EEF2,FAU,RPL11,RPL12,RPL14,RPL15,RPL17,RPL18A,RPL19,RPL22,RPL23A,RPL24,RPL26,RPL27,RPL32,RPL35,RPL35A,RPL36A,RPL37,RPL39,RPL6,RPL7A,RPL8,RPLP2,RPS12,RPS13,RPS14,RPS15,RPS15A,RPS18,RPS21,RPS23,RPS26,RPS27A,RPS28,RPS4X,RPS7,RPS8 |
| Cluster29 | CL:18633 | peptide antigen assembly with MHC class II protein complex | 4 | 5.00643 | top of list | 0.0016 | afc | CD74,HLA-DMA,HLA-DRA,HLA-DRB1 |
| Cluster29 | CL:14985 | Viral mRNA Translation | 33 | 1.61096 | top of list | 0.0016 | ks | FAU,RPL11,RPL12,RPL14,RPL15,RPL17,RPL18A,RPL19,RPL22,RPL23A,RPL24,RPL26,RPL27,RPL35,RPL35A,RPL39,RPL6,RPL7A,RPL8,RPS12,RPS13,RPS14,RPS15,RPS15A,RPS18,RPS21,RPS23,RPS26,RPS27A,RPS28,RPS4X,RPS7,RPS8 |

**Table S6 Compared with the normal controls, significantly enriched pathways of each cell (sub)types in cured patients (patient 11 and patient 12). Cell type refers to Fig. 1d.**

| **Cluster** | **#term ID** | **term description** | **genes mapped** | **enrichment score** | **direction** | **false discovery rate** | **method** | **matching proteins in your input (labels)** |
| --- | --- | --- | --- | --- | --- | --- | --- | --- |
| Cluster2 | CL:2403 | mixed, incl. MAPK targets/ Nuclear events mediated by MAP kinases, and bZIP Maf transcription factor | 4 | 6.38579 | top of list | 0.0072 | afc | DUSP1,FOS,JUN,JUNB |
| Cluster6 | CL:2403 | mixed, incl. MAPK targets/ Nuclear events mediated by MAP kinases, and bZIP Maf transcription factor | 5 | 6.9493 | top of list | 0.0049 | afc | DUSP1,DUSP2,FOS,JUN,JUNB |
| Cluster6 | CL:2482 | AP-1 transcription factor, and Transcription factor Jun | 3 | 8.04085 | top of list | 0.0099 | afc | FOS,JUN,JUNB |
| Cluster11 | CL:4300 | mixed, incl. Granzyme B, and Granulysin-like | 3 | 9.3506 | bottom of list | 0.0035 | afc | GZMB,NKG7,PRF1 |
| Cluster11 | CL:22328 | respirasome | 10 | 3.95079 | top of list | 0.0053 | afc | MT-ATP6,MT-CO1,MT-CO2,MT-CO3,MT-CYB,MT-ND1,MT-ND2,MT-ND4,MT-ND4L,MT-ND5 |
| Cluster11 | CL:22327 | Oxidative phosphorylation | 11 | 3.73292 | top of list | 0.0053 | afc | MT-ATP6,MT-ATP8,MT-CO1,MT-CO2,MT-CO3,MT-CYB,MT-ND1,MT-ND2,MT-ND4,MT-ND4L,MT-ND5 |
| Cluster11 | CL:4211 | Immunoregulatory interactions between a Lymphoid and a non-Lymphoid cell, and Cytolysis | 9 | 3.7213 | both ends | 0.0053 | afc | B2M,CD48,GZMB,HLA-A,HLA-B,HLA-C,HLA-E,NKG7,PRF1 |
| Cluster11 | CL:4212 | Immunoregulatory interactions between a Lymphoid and a non-Lymphoid cell, and Cytolysis | 8 | 4.24936 | both ends | 0.0053 | afc | B2M,GZMB,HLA-A,HLA-B,HLA-C,HLA-E,NKG7,PRF1 |
| Cluster13 | CL:22327 | Oxidative phosphorylation | 11 | 4.63121 | top of list | 5.29E-05 | afc | MT-ATP6,MT-ATP8,MT-CO1,MT-CO2,MT-CO3,MT-CYB,MT-ND1,MT-ND2,MT-ND4,MT-ND4L,MT-ND5 |
| Cluster13 | CL:22328 | respirasome | 10 | 4.75263 | top of list | 7.14E-05 | afc | MT-ATP6,MT-CO1,MT-CO2,MT-CO3,MT-CYB,MT-ND1,MT-ND2,MT-ND4,MT-ND4L,MT-ND5 |
| Cluster13 | CL:22446 | cytochrome complex, and Cytochrome c oxidase subunit VII | 6 | 4.96112 | top of list | 0.0014 | afc | MT-ATP6,MT-CO1,MT-CO2,MT-CO3,MT-CYB,MT-ND4 |
| Cluster13 | CL:22502 | Leber hereditary optic neuropathy | 5 | 5.64186 | top of list | 0.0014 | afc | MT-ATP6,MT-CO1,MT-CO3,MT-CYB,MT-ND4 |
| Cluster17 | CL:14976 | Peptide chain elongation | 78 | 2.24567 | top of list | 3.59E-17 | ks | EEF1A1,EEF2,FAU,GNB2L1,RPL10,RPL10A,RPL11,RPL12,RPL13,RPL13A,RPL14,RPL15,RPL17,RPL18,RPL18A,RPL19,RPL21,RPL22,RPL23,RPL23A,RPL24,RPL26,RPL27,RPL27A,RPL28,RPL29,RPL3,RPL30,RPL31,RPL32,RPL34,RPL35,RPL35A,RPL36,RPL36A,RPL36AL,RPL37,RPL37A,RPL38,RPL39,RPL4,RPL5,RPL6,RPL7,RPL7A,RPL8,RPL9,RPLP0,RPLP1,RPLP2,RPS11,RPS12,RPS13,RPS14,RPS15,RPS15A,RPS16,RPS18,RPS19,RPS2,RPS20,RPS21,RPS23,RPS24,RPS25,RPS26,RPS27,RPS27A,RPS28,RPS29,RPS3A,RPS4X,RPS5,RPS6,RPS7,RPS8,RPS9,UBA52 |
| Cluster17 | CL:14978 | Peptide chain elongation | 76 | 2.29497 | top of list | 3.59E-17 | ks | EEF1A1,EEF2,FAU,GNB2L1,RPL10,RPL10A,RPL11,RPL12,RPL13,RPL13A,RPL14,RPL15,RPL17,RPL18,RPL18A,RPL19,RPL21,RPL22,RPL23,RPL23A,RPL24,RPL26,RPL27,RPL27A,RPL28,RPL29,RPL3,RPL31,RPL32,RPL34,RPL35,RPL35A,RPL36,RPL36A,RPL37,RPL37A,RPL38,RPL39,RPL4,RPL5,RPL6,RPL7,RPL7A,RPL8,RPL9,RPLP0,RPLP1,RPLP2,RPS11,RPS12,RPS13,RPS14,RPS15,RPS15A,RPS16,RPS18,RPS19,RPS2,RPS20,RPS21,RPS23,RPS24,RPS25,RPS26,RPS27,RPS27A,RPS28,RPS29,RPS3A,RPS4X,RPS5,RPS6,RPS7,RPS8,RPS9,UBA52 |
| Cluster17 | CL:14973 | Peptide chain elongation | 79 | 2.20078 | top of list | 8.34E-17 | ks | EEF1A1,EEF2,FAU,GNB2L1,RPL10,RPL10A,RPL11,RPL12,RPL13,RPL13A,RPL14,RPL15,RPL17,RPL18,RPL18A,RPL19,RPL21,RPL22,RPL23,RPL23A,RPL24,RPL26,RPL27,RPL27A,RPL28,RPL29,RPL3,RPL30,RPL31,RPL32,RPL34,RPL35,RPL35A,RPL36,RPL36A,RPL36AL,RPL37,RPL37A,RPL38,RPL39,RPL4,RPL5,RPL6,RPL7,RPL7A,RPL8,RPL9,RPLP0,RPLP1,RPLP2,RPS11,RPS12,RPS13,RPS14,RPS15,RPS15A,RPS16,RPS18,RPS19,RPS2,RPS20,RPS21,RPS23,RPS24,RPS25,RPS26,RPS27,RPS27A,RPS28,RPS29,RPS3A,RPS4X,RPS5,RPS6,RPS7,RPS8,RPS9,SRP14,UBA52 |
| Cluster17 | CL:14980 | Peptide chain elongation | 72 | 2.25682 | top of list | 1.09E-16 | ks | EEF2,FAU,RPL10,RPL10A,RPL11,RPL12,RPL13,RPL13A,RPL14,RPL15,RPL17,RPL18,RPL18A,RPL19,RPL21,RPL22,RPL23,RPL23A,RPL24,RPL26,RPL27,RPL27A,RPL28,RPL29,RPL3,RPL31,RPL32,RPL35,RPL35A,RPL36,RPL36A,RPL37,RPL37A,RPL38,RPL39,RPL5,RPL6,RPL7,RPL7A,RPL8,RPL9,RPLP0,RPLP1,RPLP2,RPS11,RPS12,RPS13,RPS14,RPS15,RPS15A,RPS16,RPS18,RPS19,RPS2,RPS20,RPS21,RPS23,RPS24,RPS25,RPS26,RPS27,RPS27A,RPS28,RPS29,RPS3A,RPS4X,RPS5,RPS6,RPS7,RPS8,RPS9,UBA52 |
| Cluster17 | CL:14968 | SRP-dependent cotranslational protein targeting to membrane | 80 | 2.14643 | top of list | 1.68E-16 | ks | EEF1A1,EEF2,FAU,GNB2L1,RPL10,RPL10A,RPL11,RPL12,RPL13,RPL13A,RPL14,RPL15,RPL17,RPL18,RPL18A,RPL19,RPL21,RPL22,RPL23,RPL23A,RPL24,RPL26,RPL27,RPL27A,RPL28,RPL29,RPL3,RPL30,RPL31,RPL32,RPL34,RPL35,RPL35A,RPL36,RPL36A,RPL36AL,RPL37,RPL37A,RPL38,RPL39,RPL4,RPL5,RPL6,RPL7,RPL7A,RPL8,RPL9,RPLP0,RPLP1,RPLP2,RPS11,RPS12,RPS13,RPS14,RPS15,RPS15A,RPS16,RPS18,RPS19,RPS2,RPS20,RPS21,RPS23,RPS24,RPS25,RPS26,RPS27,RPS27A,RPS28,RPS29,RPS3A,RPS4X,RPS5,RPS6,RPS7,RPS8,RPS9,SRP14,SSR2,UBA52 |
| **Cluster** | **#term ID** | **term description** | **genes mapped** | **enrichment score** | **direction** | **false discovery rate** | **method** | **matching proteins in your input (labels)** |
| Cluster17 | CL:14982 | Peptide chain elongation | 70 | 2.25502 | top of list | 2.55E-16 | ks | EEF2,FAU,RPL10,RPL10A,RPL11,RPL12,RPL13,RPL13A,RPL14,RPL15,RPL17,RPL18A,RPL19,RPL21,RPL22,RPL23,RPL23A,RPL24,RPL26,RPL27,RPL28,RPL29,RPL3,RPL31,RPL32,RPL35,RPL35A,RPL36,RPL36A,RPL37,RPL37A,RPL38,RPL39,RPL5,RPL6,RPL7,RPL7A,RPL8,RPL9,RPLP0,RPLP1,RPLP2,RPS11,RPS12,RPS13,RPS14,RPS15,RPS15A,RPS16,RPS18,RPS19,RPS2,RPS20,RPS21,RPS23,RPS24,RPS25,RPS26,RPS27,RPS27A,RPS28,RPS29,RPS3A,RPS4X,RPS5,RPS6,RPS7,RPS8,RPS9,UBA52 |
| Cluster17 | CL:14966 | GTP hydrolysis and joining of the 60S ribosomal subunit, and Protein export | 95 | 1.88383 | top of list | 9.24E-14 | ks | EEF1A1,EEF1B2,EEF1D,EEF2,EIF1,EIF2S3,EIF3E,EIF3F,EIF3H,EIF3K,EIF3L,EIF4A2,EIF4B,FAU,GNB2L1,PABPC1,RPL10,RPL10A,RPL11,RPL12,RPL13,RPL13A,RPL14,RPL15,RPL17,RPL18,RPL18A,RPL19,RPL21,RPL22,RPL23,RPL23A,RPL24,RPL26,RPL27,RPL27A,RPL28,RPL29,RPL3,RPL30,RPL31,RPL32,RPL34,RPL35,RPL35A,RPL36,RPL36A,RPL36AL,RPL37,RPL37A,RPL38,RPL39,RPL4,RPL5,RPL6,RPL7,RPL7A,RPL8,RPL9,RPLP0,RPLP1,RPLP2,RPS11,RPS12,RPS13,RPS14,RPS15,RPS15A,RPS16,RPS18,RPS19,RPS2,RPS20,RPS21,RPS23,RPS24,RPS25,RPS26,RPS27,RPS27A,RPS28,RPS29,RPS3,RPS3A,RPS4X,RPS5,RPS6,RPS7,RPS8,RPS9,RPSA,SRP14,SSR2,TPT1,UBA52 |
| Cluster17 | CL:14967 | GTP hydrolysis and joining of the 60S ribosomal subunit, and Protein export | 92 | 1.87332 | top of list | 9.85E-14 | ks | EEF1A1,EEF2,EIF1,EIF2S3,EIF3E,EIF3F,EIF3H,EIF3K,EIF3L,EIF4A2,EIF4B,FAU,GNB2L1,PABPC1,RPL10,RPL10A,RPL11,RPL12,RPL13,RPL13A,RPL14,RPL15,RPL17,RPL18,RPL18A,RPL19,RPL21,RPL22,RPL23,RPL23A,RPL24,RPL26,RPL27,RPL27A,RPL28,RPL29,RPL3,RPL30,RPL31,RPL32,RPL34,RPL35,RPL35A,RPL36,RPL36A,RPL36AL,RPL37,RPL37A,RPL38,RPL39,RPL4,RPL5,RPL6,RPL7,RPL7A,RPL8,RPL9,RPLP0,RPLP1,RPLP2,RPS11,RPS12,RPS13,RPS14,RPS15,RPS15A,RPS16,RPS18,RPS19,RPS2,RPS20,RPS21,RPS23,RPS24,RPS25,RPS26,RPS27,RPS27A,RPS28,RPS29,RPS3,RPS3A,RPS4X,RPS5,RPS6,RPS7,RPS8,RPS9,RPSA,SRP14,SSR2,UBA52 |
| Cluster17 | CL:14983 | Peptide chain elongation | 60 | 2.23718 | top of list | 2.16E-13 | ks | EEF2,FAU,RPL10A,RPL11,RPL12,RPL13,RPL13A,RPL14,RPL15,RPL17,RPL18A,RPL19,RPL21,RPL22,RPL23,RPL23A,RPL24,RPL26,RPL27,RPL29,RPL3,RPL32,RPL35,RPL35A,RPL36,RPL36A,RPL37,RPL37A,RPL38,RPL39,RPL6,RPL7,RPL7A,RPL8,RPL9,RPLP2,RPS11,RPS12,RPS13,RPS14,RPS15,RPS15A,RPS16,RPS18,RPS19,RPS2,RPS21,RPS23,RPS25,RPS26,RPS27A,RPS28,RPS3A,RPS4X,RPS5,RPS6,RPS7,RPS8,RPS9,UBA52 |
| Cluster17 | CL:14965 | GTP hydrolysis and joining of the 60S ribosomal subunit, and Nonsense-mediated mRNA decay | 97 | 1.8138 | top of list | 4.00E-13 | ks | EEF1A1,EEF1B2,EEF1D,EEF2,EIF1,EIF2S3,EIF3E,EIF3F,EIF3H,EIF3K,EIF3L,EIF4A2,EIF4B,FAU,GNB2L1,PABPC1,PNRC2,RPL10,RPL10A,RPL11,RPL12,RPL13,RPL13A,RPL14,RPL15,RPL17,RPL18,RPL18A,RPL19,RPL21,RPL22,RPL23,RPL23A,RPL24,RPL26,RPL27,RPL27A,RPL28,RPL29,RPL3,RPL30,RPL31,RPL32,RPL34,RPL35,RPL35A,RPL36,RPL36A,RPL36AL,RPL37,RPL37A,RPL38,RPL39,RPL4,RPL5,RPL6,RPL7,RPL7A,RPL8,RPL9,RPLP0,RPLP1,RPLP2,RPS11,RPS12,RPS13,RPS14,RPS15,RPS15A,RPS16,RPS18,RPS19,RPS2,RPS20,RPS21,RPS23,RPS24,RPS25,RPS26,RPS27,RPS27A,RPS28,RPS29,RPS3,RPS3A,RPS4X,RPS5,RPS6,RPS7,RPS8,RPS9,RPSA,SRP14,SRRM1,SSR2,TPT1,UBA52 |
| Cluster17 | CL:14985 | Viral mRNA Translation | 55 | 2.29165 | top of list | 6.94E-13 | ks | FAU,RPL10A,RPL11,RPL12,RPL13,RPL13A,RPL14,RPL15,RPL17,RPL18A,RPL19,RPL21,RPL22,RPL23,RPL23A,RPL24,RPL26,RPL27,RPL29,RPL3,RPL35,RPL35A,RPL36,RPL37A,RPL38,RPL39,RPL6,RPL7,RPL7A,RPL8,RPL9,RPS11,RPS12,RPS13,RPS14,RPS15,RPS15A,RPS16,RPS18,RPS19,RPS2,RPS21,RPS23,RPS25,RPS26,RPS27A,RPS28,RPS3A,RPS4X,RPS5,RPS6,RPS7,RPS8,RPS9,UBA52 |
| Cluster17 | CL:14963 | GTP hydrolysis and joining of the 60S ribosomal subunit, and Nonsense-mediated mRNA decay | 100 | 1.763 | top of list | 1.08E-12 | ks | BTF3,EEF1A1,EEF1B2,EEF1D,EEF2,EIF1,EIF2S3,EIF3E,EIF3F,EIF3H,EIF3K,EIF3L,EIF4A2,EIF4B,EIF5A,FAU,GNB2L1,NACA,PABPC1,PNRC2,RPL10,RPL10A,RPL11,RPL12,RPL13,RPL13A,RPL14,RPL15,RPL17,RPL18,RPL18A,RPL19,RPL21,RPL22,RPL23,RPL23A,RPL24,RPL26,RPL27,RPL27A,RPL28,RPL29,RPL3,RPL30,RPL31,RPL32,RPL34,RPL35,RPL35A,RPL36,RPL36A,RPL36AL,RPL37,RPL37A,RPL38,RPL39,RPL4,RPL5,RPL6,RPL7,RPL7A,RPL8,RPL9,RPLP0,RPLP1,RPLP2,RPS11,RPS12,RPS13,RPS14,RPS15,RPS15A,RPS16,RPS18,RPS19,RPS2,RPS20,RPS21,RPS23,RPS24,RPS25,RPS26,RPS27,RPS27A,RPS28,RPS29,RPS3,RPS3A,RPS4X,RPS5,RPS6,RPS7,RPS8,RPS9,RPSA,SRP14,SRRM1,SSR2,TPT1,UBA52 |
| Cluster19 | CL:14976 | Peptide chain elongation | 78 | 2.59884 | top of list | 7.12E-16 | ks | EEF1A1,EEF2,FAU,GNB2L1,RPL10,RPL10A,RPL11,RPL12,RPL13,RPL13A,RPL14,RPL15,RPL17,RPL18,RPL18A,RPL19,RPL21,RPL22,RPL23,RPL23A,RPL24,RPL26,RPL27,RPL27A,RPL28,RPL29,RPL3,RPL30,RPL31,RPL32,RPL34,RPL35,RPL35A,RPL36,RPL36A,RPL36AL,RPL37,RPL37A,RPL38,RPL39,RPL4,RPL5,RPL6,RPL7,RPL7A,RPL8,RPL9,RPLP0,RPLP1,RPLP2,RPS11,RPS12,RPS13,RPS14,RPS15,RPS15A,RPS16,RPS18,RPS19,RPS2,RPS20,RPS21,RPS23,RPS24,RPS25,RPS26,RPS27,RPS27A,RPS28,RPS29,RPS3A,RPS4X,RPS5,RPS6,RPS7,RPS8,RPS9,UBA52 |
| Cluster19 | CL:14978 | Peptide chain elongation | 76 | 2.62968 | top of list | 7.12E-16 | ks | EEF1A1,EEF2,FAU,GNB2L1,RPL10,RPL10A,RPL11,RPL12,RPL13,RPL13A,RPL14,RPL15,RPL17,RPL18,RPL18A,RPL19,RPL21,RPL22,RPL23,RPL23A,RPL24,RPL26,RPL27,RPL27A,RPL28,RPL29,RPL3,RPL31,RPL32,RPL34,RPL35,RPL35A,RPL36,RPL36A,RPL37,RPL37A,RPL38,RPL39,RPL4,RPL5,RPL6,RPL7,RPL7A,RPL8,RPL9,RPLP0,RPLP1,RPLP2,RPS11,RPS12,RPS13,RPS14,RPS15,RPS15A,RPS16,RPS18,RPS19,RPS2,RPS20,RPS21,RPS23,RPS24,RPS25,RPS26,RPS27,RPS27A,RPS28,RPS29,RPS3A,RPS4X,RPS5,RPS6,RPS7,RPS8,RPS9,UBA52 |
| Cluster19 | CL:14973 | Peptide chain elongation | 79 | 2.57072 | top of list | 1.61E-15 | ks | EEF1A1,EEF2,FAU,GNB2L1,RPL10,RPL10A,RPL11,RPL12,RPL13,RPL13A,RPL14,RPL15,RPL17,RPL18,RPL18A,RPL19,RPL21,RPL22,RPL23,RPL23A,RPL24,RPL26,RPL27,RPL27A,RPL28,RPL29,RPL3,RPL30,RPL31,RPL32,RPL34,RPL35,RPL35A,RPL36,RPL36A,RPL36AL,RPL37,RPL37A,RPL38,RPL39,RPL4,RPL5,RPL6,RPL7,RPL7A,RPL8,RPL9,RPLP0,RPLP1,RPLP2,RPS11,RPS12,RPS13,RPS14,RPS15,RPS15A,RPS16,RPS18,RPS19,RPS2,RPS20,RPS21,RPS23,RPS24,RPS25,RPS26,RPS27,RPS27A,RPS28,RPS29,RPS3A,RPS4X,RPS5,RPS6,RPS7,RPS8,RPS9,SRP14,UBA52 |
| Cluster19 | CL:14980 | Peptide chain elongation | 72 | 2.57196 | top of list | 7.27E-15 | ks | EEF2,FAU,RPL10,RPL10A,RPL11,RPL12,RPL13,RPL13A,RPL14,RPL15,RPL17,RPL18,RPL18A,RPL19,RPL21,RPL22,RPL23,RPL23A,RPL24,RPL26,RPL27,RPL27A,RPL28,RPL29,RPL3,RPL31,RPL32,RPL35,RPL35A,RPL36,RPL36A,RPL37,RPL37A,RPL38,RPL39,RPL5,RPL6,RPL7,RPL7A,RPL8,RPL9,RPLP0,RPLP1,RPLP2,RPS11,RPS12,RPS13,RPS14,RPS15,RPS15A,RPS16,RPS18,RPS19,RPS2,RPS20,RPS21,RPS23,RPS24,RPS25,RPS26,RPS27,RPS27A,RPS28,RPS29,RPS3A,RPS4X,RPS5,RPS6,RPS7,RPS8,RPS9,UBA52 |
| Cluster19 | CL:14982 | Peptide chain elongation | 70 | 2.58982 | top of list | 1.16E-14 | ks | EEF2,FAU,RPL10,RPL10A,RPL11,RPL12,RPL13,RPL13A,RPL14,RPL15,RPL17,RPL18A,RPL19,RPL21,RPL22,RPL23,RPL23A,RPL24,RPL26,RPL27,RPL28,RPL29,RPL3,RPL31,RPL32,RPL35,RPL35A,RPL36,RPL36A,RPL37,RPL37A,RPL38,RPL39,RPL5,RPL6,RPL7,RPL7A,RPL8,RPL9,RPLP0,RPLP1,RPLP2,RPS11,RPS12,RPS13,RPS14,RPS15,RPS15A,RPS16,RPS18,RPS19,RPS2,RPS20,RPS21,RPS23,RPS24,RPS25,RPS26,RPS27,RPS27A,RPS28,RPS29,RPS3A,RPS4X,RPS5,RPS6,RPS7,RPS8,RPS9,UBA52 |
| Cluster19 | CL:14968 | SRP-dependent cotranslational protein targeting to membrane | 82 | 2.41165 | top of list | 4.85E-14 | ks | EEF1A1,EEF2,FAU,GNB2L1,RPL10,RPL10A,RPL11,RPL12,RPL13,RPL13A,RPL14,RPL15,RPL17,RPL18,RPL18A,RPL19,RPL21,RPL22,RPL23,RPL23A,RPL24,RPL26,RPL27,RPL27A,RPL28,RPL29,RPL3,RPL30,RPL31,RPL32,RPL34,RPL35,RPL35A,RPL36,RPL36A,RPL36AL,RPL37,RPL37A,RPL38,RPL39,RPL4,RPL5,RPL6,RPL7,RPL7A,RPL8,RPL9,RPLP0,RPLP1,RPLP2,RPS11,RPS12,RPS13,RPS14,RPS15,RPS15A,RPS16,RPS18,RPS19,RPS2,RPS20,RPS21,RPS23,RPS24,RPS25,RPS26,RPS27,RPS27A,RPS28,RPS29,RPS3A,RPS4X,RPS5,RPS6,RPS7,RPS8,RPS9,SPCS1,SRP14,SSR2,SSR4,UBA52 |
| Cluster19 | CL:14983 | Peptide chain elongation | 60 | 2.52423 | top of list | 9.85E-13 | ks | EEF2,FAU,RPL10A,RPL11,RPL12,RPL13,RPL13A,RPL14,RPL15,RPL17,RPL18A,RPL19,RPL21,RPL22,RPL23,RPL23A,RPL24,RPL26,RPL27,RPL29,RPL3,RPL32,RPL35,RPL35A,RPL36,RPL36A,RPL37,RPL37A,RPL38,RPL39,RPL6,RPL7,RPL7A,RPL8,RPL9,RPLP2,RPS11,RPS12,RPS13,RPS14,RPS15,RPS15A,RPS16,RPS18,RPS19,RPS2,RPS21,RPS23,RPS25,RPS26,RPS27A,RPS28,RPS3A,RPS4X,RPS5,RPS6,RPS7,RPS8,RPS9,UBA52 |
| **Cluster** | **#term ID** | **term description** | **genes mapped** | **enrichment score** | **direction** | **false discovery rate** | **method** | **matching proteins in your input (labels)** |
| Cluster19 | CL:14963 | GTP hydrolysis and joining of the 60S ribosomal subunit, and Nonsense-mediated mRNA decay | 97 | 2.16469 | top of list | 1.35E-12 | ks | BTF3,EEF1A1,EEF1B2,EEF1D,EEF2,EIF1,EIF3F,EIF3G,EIF3H,EIF3K,EIF4B,FAU,GNB2L1,NACA,PABPC1,RPL10,RPL10A,RPL11,RPL12,RPL13,RPL13A,RPL14,RPL15,RPL17,RPL18,RPL18A,RPL19,RPL21,RPL22,RPL23,RPL23A,RPL24,RPL26,RPL27,RPL27A,RPL28,RPL29,RPL3,RPL30,RPL31,RPL32,RPL34,RPL35,RPL35A,RPL36,RPL36A,RPL36AL,RPL37,RPL37A,RPL38,RPL39,RPL4,RPL5,RPL6,RPL7,RPL7A,RPL8,RPL9,RPLP0,RPLP1,RPLP2,RPS11,RPS12,RPS13,RPS14,RPS15,RPS15A,RPS16,RPS18,RPS19,RPS2,RPS20,RPS21,RPS23,RPS24,RPS25,RPS26,RPS27,RPS27A,RPS28,RPS29,RPS3,RPS3A,RPS4X,RPS5,RPS6,RPS7,RPS8,RPS9,RPSA,SPCS1,SRP14,SRRM1,SSR2,SSR4,TPT1,UBA52 |
| Cluster19 | CL:14966 | GTP hydrolysis and joining of the 60S ribosomal subunit, and Protein export | 94 | 2.21369 | top of list | 1.64E-12 | ks | EEF1A1,EEF1B2,EEF1D,EEF2,EIF1,EIF3F,EIF3G,EIF3H,EIF3K,EIF4B,FAU,GNB2L1,PABPC1,RPL10,RPL10A,RPL11,RPL12,RPL13,RPL13A,RPL14,RPL15,RPL17,RPL18,RPL18A,RPL19,RPL21,RPL22,RPL23,RPL23A,RPL24,RPL26,RPL27,RPL27A,RPL28,RPL29,RPL3,RPL30,RPL31,RPL32,RPL34,RPL35,RPL35A,RPL36,RPL36A,RPL36AL,RPL37,RPL37A,RPL38,RPL39,RPL4,RPL5,RPL6,RPL7,RPL7A,RPL8,RPL9,RPLP0,RPLP1,RPLP2,RPS11,RPS12,RPS13,RPS14,RPS15,RPS15A,RPS16,RPS18,RPS19,RPS2,RPS20,RPS21,RPS23,RPS24,RPS25,RPS26,RPS27,RPS27A,RPS28,RPS29,RPS3,RPS3A,RPS4X,RPS5,RPS6,RPS7,RPS8,RPS9,RPSA,SPCS1,SRP14,SSR2,SSR4,TPT1,UBA52 |
| Cluster19 | CL:14967 | GTP hydrolysis and joining of the 60S ribosomal subunit, and Protein export | 91 | 2.20964 | top of list | 2.00E-12 | ks | EEF1A1,EEF2,EIF1,EIF3F,EIF3G,EIF3H,EIF3K,EIF4B,FAU,GNB2L1,PABPC1,RPL10,RPL10A,RPL11,RPL12,RPL13,RPL13A,RPL14,RPL15,RPL17,RPL18,RPL18A,RPL19,RPL21,RPL22,RPL23,RPL23A,RPL24,RPL26,RPL27,RPL27A,RPL28,RPL29,RPL3,RPL30,RPL31,RPL32,RPL34,RPL35,RPL35A,RPL36,RPL36A,RPL36AL,RPL37,RPL37A,RPL38,RPL39,RPL4,RPL5,RPL6,RPL7,RPL7A,RPL8,RPL9,RPLP0,RPLP1,RPLP2,RPS11,RPS12,RPS13,RPS14,RPS15,RPS15A,RPS16,RPS18,RPS19,RPS2,RPS20,RPS21,RPS23,RPS24,RPS25,RPS26,RPS27,RPS27A,RPS28,RPS29,RPS3,RPS3A,RPS4X,RPS5,RPS6,RPS7,RPS8,RPS9,RPSA,SPCS1,SRP14,SSR2,SSR4,UBA52 |
| Cluster19 | CL:14985 | Viral mRNA Translation | 55 | 2.56769 | top of list | 2.51E-12 | ks | FAU,RPL10A,RPL11,RPL12,RPL13,RPL13A,RPL14,RPL15,RPL17,RPL18A,RPL19,RPL21,RPL22,RPL23,RPL23A,RPL24,RPL26,RPL27,RPL29,RPL3,RPL35,RPL35A,RPL36,RPL37A,RPL38,RPL39,RPL6,RPL7,RPL7A,RPL8,RPL9,RPS11,RPS12,RPS13,RPS14,RPS15,RPS15A,RPS16,RPS18,RPS19,RPS2,RPS21,RPS23,RPS25,RPS26,RPS27A,RPS28,RPS3A,RPS4X,RPS5,RPS6,RPS7,RPS8,RPS9,UBA52 |
| Cluster19 | CL:14965 | GTP hydrolysis and joining of the 60S ribosomal subunit, and Nonsense-mediated mRNA decay | 95 | 2.16988 | top of list | 3.02E-12 | ks | EEF1A1,EEF1B2,EEF1D,EEF2,EIF1,EIF3F,EIF3G,EIF3H,EIF3K,EIF4B,FAU,GNB2L1,PABPC1,RPL10,RPL10A,RPL11,RPL12,RPL13,RPL13A,RPL14,RPL15,RPL17,RPL18,RPL18A,RPL19,RPL21,RPL22,RPL23,RPL23A,RPL24,RPL26,RPL27,RPL27A,RPL28,RPL29,RPL3,RPL30,RPL31,RPL32,RPL34,RPL35,RPL35A,RPL36,RPL36A,RPL36AL,RPL37,RPL37A,RPL38,RPL39,RPL4,RPL5,RPL6,RPL7,RPL7A,RPL8,RPL9,RPLP0,RPLP1,RPLP2,RPS11,RPS12,RPS13,RPS14,RPS15,RPS15A,RPS16,RPS18,RPS19,RPS2,RPS20,RPS21,RPS23,RPS24,RPS25,RPS26,RPS27,RPS27A,RPS28,RPS29,RPS3,RPS3A,RPS4X,RPS5,RPS6,RPS7,RPS8,RPS9,RPSA,SPCS1,SRP14,SRRM1,SSR2,SSR4,TPT1,UBA52 |
| Cluster19 | CL:4212 | Immunoregulatory interactions between a Lymphoid and a non-Lymphoid cell, and Cytolysis | 11 | 4.74404 | top of list | 2.62E-05 | afc | B2M,GNLY,GZMB,GZMH,HCST,HLA-A,HLA-B,HLA-C,HLA-E,HLA-F,NKG7 |
| Cluster19 | CL:4218 | MHC class I protein complex, and inhibitory MHC class I receptor activity | 6 | 6.55393 | top of list | 2.62E-05 | afc | B2M,HLA-A,HLA-B,HLA-C,HLA-E,HLA-F |
| Cluster19 | CL:4213 | Immunoregulatory interactions between a Lymphoid and a non-Lymphoid cell, and FCHSD2, SH3 domain 2 | 7 | 5.8483 | top of list | 3.67E-05 | afc | B2M,HCST,HLA-A,HLA-B,HLA-C,HLA-E,HLA-F |
| Cluster19 | CL:4211 | Immunoregulatory interactions between a Lymphoid and a non-Lymphoid cell, and Cytolysis | 13 | 3.69319 | top of list | 0.00033 | afc | B2M,CD2,CD48,GNLY,GZMB,GZMH,HCST,HLA-A,HLA-B,HLA-C,HLA-E,HLA-F,NKG7 |
| Cluster19 | CL:4220 | MHC_I C-terminus, and MHC class I protein complex binding | 3 | 8.06582 | top of list | 0.00037 | afc | HLA-B,HLA-C,HLA-E |
| Cluster19 | CL:1667 | mixed, incl. dense body, and Cofilin 1 | 4 | 6.18581 | top of list | 0.0019 | afc | ACTB,ACTG1,CFL1,PFN1 |
| Cluster19 | CL:22502 | Leber hereditary optic neuropathy | 5 | 5.40806 | top of list | 0.0037 | afc | MT-ATP6,MT-CO1,MT-CO3,MT-CYB,MT-ND4 |
| Cluster24 | CL:2403 | mixed, incl. MAPK targets/ Nuclear events mediated by MAP kinases, and bZIP Maf transcription factor | 4 | 7.04443 | top of list | 0.0069 | afc | DUSP1,FOS,JUN,JUNB |
| Cluster24 | CL:18630 | MHC class II protein complex | 5 | 7.66837 | bottom of list | 0.0069 | afc | CD74,HLA-DPB1,HLA-DRA,HLA-DRB1,HLA-DRB5 |
| Cluster24 | CL:18627 | MHC class II protein complex, and dynactin complex | 6 | 7.39903 | bottom of list | 0.0069 | afc | CD74,HLA-DPB1,HLA-DQA2,HLA-DRA,HLA-DRB1,HLA-DRB5 |
| Cluster27 | CL:14978 | Peptide chain elongation | 75 | 1.74682 | top of list | 6.13E-05 | ks | EEF1A1,EEF2,FAU,GNB2L1,RPL10,RPL10A,RPL11,RPL12,RPL13,RPL13A,RPL14,RPL15,RPL18,RPL18A,RPL19,RPL21,RPL22,RPL23,RPL23A,RPL24,RPL26,RPL27,RPL27A,RPL28,RPL29,RPL3,RPL31,RPL32,RPL34,RPL35,RPL35A,RPL36,RPL36A,RPL37,RPL37A,RPL38,RPL39,RPL4,RPL5,RPL6,RPL7,RPL7A,RPL8,RPL9,RPLP0,RPLP1,RPLP2,RPS11,RPS12,RPS13,RPS14,RPS15,RPS15A,RPS16,RPS18,RPS19,RPS2,RPS20,RPS21,RPS23,RPS24,RPS25,RPS26,RPS27,RPS27A,RPS28,RPS29,RPS3A,RPS4X,RPS5,RPS6,RPS7,RPS8,RPS9,UBA52 |
| **Cluster** | **#term ID** | **term description** | **genes mapped** | **enrichment score** | **direction** | **false discovery rate** | **method** | **matching proteins in your input (labels)** |
| Cluster27 | CL:14976 | Peptide chain elongation | 76 | 1.73445 | top of list | 6.13E-05 | ks | EEF1A1,EEF2,FAU,GNB2L1,RPL10,RPL10A,RPL11,RPL12,RPL13,RPL13A,RPL14,RPL15,RPL18,RPL18A,RPL19,RPL21,RPL22,RPL23,RPL23A,RPL24,RPL26,RPL27,RPL27A,RPL28,RPL29,RPL3,RPL30,RPL31,RPL32,RPL34,RPL35,RPL35A,RPL36,RPL36A,RPL37,RPL37A,RPL38,RPL39,RPL4,RPL5,RPL6,RPL7,RPL7A,RPL8,RPL9,RPLP0,RPLP1,RPLP2,RPS11,RPS12,RPS13,RPS14,RPS15,RPS15A,RPS16,RPS18,RPS19,RPS2,RPS20,RPS21,RPS23,RPS24,RPS25,RPS26,RPS27,RPS27A,RPS28,RPS29,RPS3A,RPS4X,RPS5,RPS6,RPS7,RPS8,RPS9,UBA52 |
| Cluster27 | CL:14982 | Peptide chain elongation | 69 | 1.67953 | top of list | 7.31E-05 | ks | EEF2,FAU,RPL10,RPL10A,RPL11,RPL12,RPL13,RPL13A,RPL14,RPL15,RPL18A,RPL19,RPL21,RPL22,RPL23,RPL23A,RPL24,RPL26,RPL27,RPL28,RPL29,RPL3,RPL31,RPL32,RPL35,RPL35A,RPL36,RPL36A,RPL37,RPL37A,RPL38,RPL39,RPL5,RPL6,RPL7,RPL7A,RPL8,RPL9,RPLP0,RPLP1,RPLP2,RPS11,RPS12,RPS13,RPS14,RPS15,RPS15A,RPS16,RPS18,RPS19,RPS2,RPS20,RPS21,RPS23,RPS24,RPS25,RPS26,RPS27,RPS27A,RPS28,RPS29,RPS3A,RPS4X,RPS5,RPS6,RPS7,RPS8,RPS9,UBA52 |
| Cluster27 | CL:14963 | GTP hydrolysis and joining of the 60S ribosomal subunit, and Nonsense-mediated mRNA decay | 90 | 1.57788 | top of list | 7.31E-05 | ks | BTF3,EEF1A1,EEF1B2,EEF1D,EEF2,EIF1,EIF3E,EIF3F,EIF3H,EIF3L,FAU,GNB2L1,NACA,PABPC1,RPL10,RPL10A,RPL11,RPL12,RPL13,RPL13A,RPL14,RPL15,RPL18,RPL18A,RPL19,RPL21,RPL22,RPL23,RPL23A,RPL24,RPL26,RPL27,RPL27A,RPL28,RPL29,RPL3,RPL30,RPL31,RPL32,RPL34,RPL35,RPL35A,RPL36,RPL36A,RPL37,RPL37A,RPL38,RPL39,RPL4,RPL5,RPL6,RPL7,RPL7A,RPL8,RPL9,RPLP0,RPLP1,RPLP2,RPS11,RPS12,RPS13,RPS14,RPS15,RPS15A,RPS16,RPS18,RPS19,RPS2,RPS20,RPS21,RPS23,RPS24,RPS25,RPS26,RPS27,RPS27A,RPS28,RPS29,RPS3,RPS3A,RPS4X,RPS5,RPS6,RPS7,RPS8,RPS9,RPSA,SRRM1,TPT1,UBA52 |
| Cluster27 | CL:14966 | GTP hydrolysis and joining of the 60S ribosomal subunit, and Protein export | 87 | 1.6186 | top of list | 7.31E-05 | ks | EEF1A1,EEF1B2,EEF1D,EEF2,EIF1,EIF3E,EIF3F,EIF3H,EIF3L,FAU,GNB2L1,PABPC1,RPL10,RPL10A,RPL11,RPL12,RPL13,RPL13A,RPL14,RPL15,RPL18,RPL18A,RPL19,RPL21,RPL22,RPL23,RPL23A,RPL24,RPL26,RPL27,RPL27A,RPL28,RPL29,RPL3,RPL30,RPL31,RPL32,RPL34,RPL35,RPL35A,RPL36,RPL36A,RPL37,RPL37A,RPL38,RPL39,RPL4,RPL5,RPL6,RPL7,RPL7A,RPL8,RPL9,RPLP0,RPLP1,RPLP2,RPS11,RPS12,RPS13,RPS14,RPS15,RPS15A,RPS16,RPS18,RPS19,RPS2,RPS20,RPS21,RPS23,RPS24,RPS25,RPS26,RPS27,RPS27A,RPS28,RPS29,RPS3,RPS3A,RPS4X,RPS5,RPS6,RPS7,RPS8,RPS9,RPSA,TPT1,UBA52 |
| Cluster27 | CL:14980 | Peptide chain elongation | 71 | 1.68464 | top of list | 7.31E-05 | ks | EEF2,FAU,RPL10,RPL10A,RPL11,RPL12,RPL13,RPL13A,RPL14,RPL15,RPL18,RPL18A,RPL19,RPL21,RPL22,RPL23,RPL23A,RPL24,RPL26,RPL27,RPL27A,RPL28,RPL29,RPL3,RPL31,RPL32,RPL35,RPL35A,RPL36,RPL36A,RPL37,RPL37A,RPL38,RPL39,RPL5,RPL6,RPL7,RPL7A,RPL8,RPL9,RPLP0,RPLP1,RPLP2,RPS11,RPS12,RPS13,RPS14,RPS15,RPS15A,RPS16,RPS18,RPS19,RPS2,RPS20,RPS21,RPS23,RPS24,RPS25,RPS26,RPS27,RPS27A,RPS28,RPS29,RPS3A,RPS4X,RPS5,RPS6,RPS7,RPS8,RPS9,UBA52 |
| Cluster27 | CL:14967 | GTP hydrolysis and joining of the 60S ribosomal subunit, and Protein export | 84 | 1.63325 | top of list | 7.31E-05 | ks | EEF1A1,EEF2,EIF1,EIF3E,EIF3F,EIF3H,EIF3L,FAU,GNB2L1,PABPC1,RPL10,RPL10A,RPL11,RPL12,RPL13,RPL13A,RPL14,RPL15,RPL18,RPL18A,RPL19,RPL21,RPL22,RPL23,RPL23A,RPL24,RPL26,RPL27,RPL27A,RPL28,RPL29,RPL3,RPL30,RPL31,RPL32,RPL34,RPL35,RPL35A,RPL36,RPL36A,RPL37,RPL37A,RPL38,RPL39,RPL4,RPL5,RPL6,RPL7,RPL7A,RPL8,RPL9,RPLP0,RPLP1,RPLP2,RPS11,RPS12,RPS13,RPS14,RPS15,RPS15A,RPS16,RPS18,RPS19,RPS2,RPS20,RPS21,RPS23,RPS24,RPS25,RPS26,RPS27,RPS27A,RPS28,RPS29,RPS3,RPS3A,RPS4X,RPS5,RPS6,RPS7,RPS8,RPS9,RPSA,UBA52 |
| Cluster27 | CL:14965 | GTP hydrolysis and joining of the 60S ribosomal subunit, and Nonsense-mediated mRNA decay | 88 | 1.57071 | top of list | 9.86E-05 | ks | EEF1A1,EEF1B2,EEF1D,EEF2,EIF1,EIF3E,EIF3F,EIF3H,EIF3L,FAU,GNB2L1,PABPC1,RPL10,RPL10A,RPL11,RPL12,RPL13,RPL13A,RPL14,RPL15,RPL18,RPL18A,RPL19,RPL21,RPL22,RPL23,RPL23A,RPL24,RPL26,RPL27,RPL27A,RPL28,RPL29,RPL3,RPL30,RPL31,RPL32,RPL34,RPL35,RPL35A,RPL36,RPL36A,RPL37,RPL37A,RPL38,RPL39,RPL4,RPL5,RPL6,RPL7,RPL7A,RPL8,RPL9,RPLP0,RPLP1,RPLP2,RPS11,RPS12,RPS13,RPS14,RPS15,RPS15A,RPS16,RPS18,RPS19,RPS2,RPS20,RPS21,RPS23,RPS24,RPS25,RPS26,RPS27,RPS27A,RPS28,RPS29,RPS3,RPS3A,RPS4X,RPS5,RPS6,RPS7,RPS8,RPS9,RPSA,SRRM1,TPT1,UBA52 |
| Cluster27 | CL:14985 | Viral mRNA Translation | 54 | 1.58823 | top of list | 0.00011 | ks | FAU,RPL10A,RPL11,RPL12,RPL13,RPL13A,RPL14,RPL15,RPL18A,RPL19,RPL21,RPL22,RPL23,RPL23A,RPL24,RPL26,RPL27,RPL29,RPL3,RPL35,RPL35A,RPL36,RPL37A,RPL38,RPL39,RPL6,RPL7,RPL7A,RPL8,RPL9,RPS11,RPS12,RPS13,RPS14,RPS15,RPS15A,RPS16,RPS18,RPS19,RPS2,RPS21,RPS23,RPS25,RPS26,RPS27A,RPS28,RPS3A,RPS4X,RPS5,RPS6,RPS7,RPS8,RPS9,UBA52 |
| Cluster27 | CL:14983 | Peptide chain elongation | 59 | 1.61482 | top of list | 0.00012 | ks | EEF2,FAU,RPL10A,RPL11,RPL12,RPL13,RPL13A,RPL14,RPL15,RPL18A,RPL19,RPL21,RPL22,RPL23,RPL23A,RPL24,RPL26,RPL27,RPL29,RPL3,RPL32,RPL35,RPL35A,RPL36,RPL36A,RPL37,RPL37A,RPL38,RPL39,RPL6,RPL7,RPL7A,RPL8,RPL9,RPLP2,RPS11,RPS12,RPS13,RPS14,RPS15,RPS15A,RPS16,RPS18,RPS19,RPS2,RPS21,RPS23,RPS25,RPS26,RPS27A,RPS28,RPS3A,RPS4X,RPS5,RPS6,RPS7,RPS8,RPS9,UBA52 |
| Cluster29 | CL:14963 | GTP hydrolysis and joining of the 60S ribosomal subunit, and Nonsense-mediated mRNA decay | 44 | 1.04431 | top of list | 0.00053 | ks | BTF3,EIF2S2,EIF3M,FAU,GNB2L1,NACA,RPL11,RPL14,RPL15,RPL17,RPL18,RPL18A,RPL19,RPL28,RPL30,RPL31,RPL32,RPL34,RPL35,RPL35A,RPL37,RPL5,RPL6,RPL7A,RPL8,RPLP1,RPLP2,RPS12,RPS14,RPS15,RPS18,RPS19,RPS23,RPS24,RPS26,RPS27,RPS27A,RPS28,RPS3,RPS7,SEC11A,SRRM1,TPT1,UBA52 |
| Cluster29 | CL:14976 | Peptide chain elongation | 36 | 1.14662 | top of list | 0.00053 | ks | FAU,GNB2L1,RPL11,RPL14,RPL15,RPL17,RPL18,RPL18A,RPL19,RPL28,RPL30,RPL31,RPL32,RPL34,RPL35,RPL35A,RPL37,RPL5,RPL6,RPL7A,RPL8,RPLP1,RPLP2,RPS12,RPS14,RPS15,RPS18,RPS19,RPS23,RPS24,RPS26,RPS27,RPS27A,RPS28,RPS7,UBA52 |
| Cluster29 | CL:14982 | Peptide chain elongation | 32 | 1.21214 | top of list | 0.00053 | ks | FAU,RPL11,RPL14,RPL15,RPL17,RPL18A,RPL19,RPL28,RPL31,RPL32,RPL35,RPL35A,RPL37,RPL5,RPL6,RPL7A,RPL8,RPLP1,RPLP2,RPS12,RPS14,RPS15,RPS18,RPS19,RPS23,RPS24,RPS26,RPS27,RPS27A,RPS28,RPS7,UBA52 |
| Cluster29 | CL:14966 | GTP hydrolysis and joining of the 60S ribosomal subunit, and Protein export | 41 | 1.08911 | top of list | 0.00053 | ks | EIF2S2,EIF3M,FAU,GNB2L1,RPL11,RPL14,RPL15,RPL17,RPL18,RPL18A,RPL19,RPL28,RPL30,RPL31,RPL32,RPL34,RPL35,RPL35A,RPL37,RPL5,RPL6,RPL7A,RPL8,RPLP1,RPLP2,RPS12,RPS14,RPS15,RPS18,RPS19,RPS23,RPS24,RPS26,RPS27,RPS27A,RPS28,RPS3,RPS7,SEC11A,TPT1,UBA52 |
| Cluster29 | CL:14968 | SRP-dependent cotranslational protein targeting to membrane | 37 | 1.11724 | top of list | 0.00053 | ks | FAU,GNB2L1,RPL11,RPL14,RPL15,RPL17,RPL18,RPL18A,RPL19,RPL28,RPL30,RPL31,RPL32,RPL34,RPL35,RPL35A,RPL37,RPL5,RPL6,RPL7A,RPL8,RPLP1,RPLP2,RPS12,RPS14,RPS15,RPS18,RPS19,RPS23,RPS24,RPS26,RPS27,RPS27A,RPS28,RPS7,SEC11A,UBA52 |
| Cluster29 | CL:14980 | Peptide chain elongation | 33 | 1.23312 | top of list | 0.00053 | ks | FAU,RPL11,RPL14,RPL15,RPL17,RPL18,RPL18A,RPL19,RPL28,RPL31,RPL32,RPL35,RPL35A,RPL37,RPL5,RPL6,RPL7A,RPL8,RPLP1,RPLP2,RPS12,RPS14,RPS15,RPS18,RPS19,RPS23,RPS24,RPS26,RPS27,RPS27A,RPS28,RPS7,UBA52 |
| Cluster29 | CL:14967 | GTP hydrolysis and joining of the 60S ribosomal subunit, and Protein export | 40 | 1.13852 | top of list | 0.00053 | ks | EIF2S2,EIF3M,FAU,GNB2L1,RPL11,RPL14,RPL15,RPL17,RPL18,RPL18A,RPL19,RPL28,RPL30,RPL31,RPL32,RPL34,RPL35,RPL35A,RPL37,RPL5,RPL6,RPL7A,RPL8,RPLP1,RPLP2,RPS12,RPS14,RPS15,RPS18,RPS19,RPS23,RPS24,RPS26,RPS27,RPS27A,RPS28,RPS3,RPS7,SEC11A,UBA52 |
| Cluster29 | CL:14978 | Peptide chain elongation | 35 | 1.13411 | top of list | 0.0006 | ks | FAU,GNB2L1,RPL11,RPL14,RPL15,RPL17,RPL18,RPL18A,RPL19,RPL28,RPL31,RPL32,RPL34,RPL35,RPL35A,RPL37,RPL5,RPL6,RPL7A,RPL8,RPLP1,RPLP2,RPS12,RPS14,RPS15,RPS18,RPS19,RPS23,RPS24,RPS26,RPS27,RPS27A,RPS28,RPS7,UBA52 |
| Cluster29 | CL:14965 | GTP hydrolysis and joining of the 60S ribosomal subunit, and Nonsense-mediated mRNA decay | 42 | 1.02539 | top of list | 0.00078 | ks | EIF2S2,EIF3M,FAU,GNB2L1,RPL11,RPL14,RPL15,RPL17,RPL18,RPL18A,RPL19,RPL28,RPL30,RPL31,RPL32,RPL34,RPL35,RPL35A,RPL37,RPL5,RPL6,RPL7A,RPL8,RPLP1,RPLP2,RPS12,RPS14,RPS15,RPS18,RPS19,RPS23,RPS24,RPS26,RPS27,RPS27A,RPS28,RPS3,RPS7,SEC11A,SRRM1,TPT1,UBA52 |
| **Cluster** | **#term ID** | **term description** | **genes mapped** | **enrichment score** | **direction** | **false discovery rate** | **method** | **matching proteins in your input (labels)** |
| Cluster29 | CL:14983 | Peptide chain elongation | 26 | 1.18982 | top of list | 0.0025 | ks | FAU,RPL11,RPL14,RPL15,RPL17,RPL18A,RPL19,RPL32,RPL35,RPL35A,RPL37,RPL6,RPL7A,RPL8,RPLP2,RPS12,RPS14,RPS15,RPS18,RPS19,RPS23,RPS26,RPS27A,RPS28,RPS7,UBA52 |
| Cluster29 | CL:14985 | Viral mRNA Translation | 23 | 1.2034 | top of list | 0.0069 | ks | FAU,RPL11,RPL14,RPL15,RPL17,RPL18A,RPL19,RPL35,RPL35A,RPL6,RPL7A,RPL8,RPS12,RPS14,RPS15,RPS18,RPS19,RPS23,RPS26,RPS27A,RPS28,RPS7,UBA52 |

**Table S7 List of the most enriched V(D)J clonotypic T cells in each patient by TCR single-cell transcriptome analysis.**

| **TRA-CDR3-TRB-CDR3** | **Patient1** | **Patient2** | **Patient3** | **Patient4** | **Patient5** | **Patient6** | **Patient7** | **Patient8** | **Patient9** | **Patient10** | **Patient11** | **Patient12** | **Patient13** | **Patient14** | **Patient15** | **Patient16** |
| --- | --- | --- | --- | --- | --- | --- | --- | --- | --- | --- | --- | --- | --- | --- | --- | --- |
| CGTDADNNNDMRF-CASSLTSGRGEQYF | 6 | 0 | 0 | 0 | 0 | 0 | 0 | 0 | 0 | 0 | 0 | 0 | 0 | 0 | 0 | 0 |
| CAVTSGSRLTF-CASSLLPWRELFF | 6 | 0 | 0 | 0 | 0 | 0 | 0 | 0 | 0 | 0 | 0 | 0 | 0 | 0 | 0 | 0 |
| CVVNVGNGNKLVF-CASSPPEGGSSYNEQFF | 5 | 0 | 0 | 0 | 0 | 0 | 0 | 0 | 0 | 0 | 0 | 0 | 0 | 0 | 0 | 0 |
| CALSDADMDTGRRALTF-CASSTEQGARSDTQYF | 5 | 0 | 0 | 0 | 0 | 0 | 0 | 0 | 0 | 0 | 0 | 0 | 0 | 0 | 0 | 0 |
| CALRSGGSYIPTF-CASSSLDSYEQYF | 5 | 0 | 0 | 0 | 0 | 0 | 0 | 0 | 0 | 0 | 0 | 0 | 0 | 0 | 0 | 0 |
| CATDPWNNNDMRF-CASSQGTGLQETQYF | 0 | 11 | 0 | 0 | 0 | 0 | 0 | 0 | 0 | 0 | 0 | 0 | 0 | 0 | 0 | 0 |
| CVVNYYKAAGNKLTF-CASSLGSAPRELFF | 0 | 11 | 0 | 0 | 0 | 0 | 0 | 0 | 0 | 0 | 0 | 0 | 0 | 0 | 0 | 0 |
| CAASPPYNTDKLIF-CASSLTWGQGAPDTQYF | 0 | 8 | 0 | 0 | 0 | 0 | 0 | 0 | 0 | 0 | 0 | 0 | 0 | 0 | 0 | 0 |
| CAVLLERSKAAGNKLTF-CASRGAYSPLHF | 0 | 7 | 0 | 0 | 0 | 0 | 0 | 0 | 0 | 0 | 0 | 0 | 0 | 0 | 0 | 0 |
| CAVSCSQGGSEKLVF-CASSLFVAAYNNEQFF | 0 | 6 | 0 | 0 | 0 | 0 | 0 | 0 | 0 | 0 | 0 | 0 | 0 | 0 | 0 | 0 |
| CAMKTSYDKVIF-CASTPGDTIYF | 0 | 6 | 0 | 0 | 0 | 0 | 0 | 0 | 0 | 0 | 0 | 0 | 0 | 0 | 0 | 0 |
| CAVQGGANNLFF-CASSSLAGGYEQYF | 0 | 4 | 0 | 0 | 0 | 0 | 0 | 0 | 0 | 0 | 0 | 0 | 0 | 0 | 0 | 0 |
| CAFRKDTGRRALTF-CASSPQGGGGYTF | 0 | 4 | 0 | 0 | 0 | 0 | 0 | 0 | 0 | 0 | 0 | 0 | 0 | 0 | 0 | 0 |
| CAIDLSSGSARQLTF-CASKGNSYEQYF | 0 | 0 | 0 | 182 | 0 | 0 | 0 | 0 | 0 | 0 | 0 | 0 | 0 | 0 | 0 | 0 |
| CAVRTAGNKLTF-CASSDPVTQYF | 0 | 0 | 0 | 143 | 0 | 0 | 0 | 0 | 0 | 0 | 0 | 0 | 0 | 0 | 0 | 0 |
| CAVKNAGNMLTF-CASQGPNTEAFF | 0 | 0 | 0 | 69 | 0 | 0 | 0 | 0 | 0 | 0 | 0 | 0 | 0 | 0 | 0 | 0 |
| CVVNRDFQKLVF-CASRALGVSSGNYGYTF | 0 | 0 | 0 | 44 | 0 | 0 | 0 | 0 | 0 | 0 | 0 | 0 | 0 | 0 | 0 | 0 |
| CAVLAQANDYKLSF-CASSGNTGGSYEQYF | 0 | 0 | 0 | 36 | 0 | 0 | 0 | 0 | 0 | 0 | 0 | 0 | 0 | 0 | 0 | 0 |
| CAVKGTYKYIF-CASSVGGTLSSYNEQFF | 0 | 0 | 0 | 23 | 0 | 0 | 0 | 0 | 0 | 0 | 0 | 0 | 0 | 0 | 0 | 0 |
| CAVRQAGNQFYF-CASSGNTGGSYEQYF | 0 | 0 | 0 | 16 | 0 | 0 | 0 | 0 | 0 | 0 | 0 | 0 | 0 | 0 | 0 | 0 |
| CAASANNARLMF-CSASVQRENDEQFF | 0 | 0 | 0 | 15 | 0 | 0 | 0 | 0 | 0 | 0 | 0 | 0 | 0 | 0 | 0 | 0 |
| CAFMGGSARQLTF-CASRERGGNTEAFF | 0 | 0 | 0 | 15 | 0 | 0 | 0 | 0 | 0 | 0 | 0 | 0 | 0 | 0 | 0 | 0 |
| CAGPRDLYNQGGKLIF-CAAKTATNEKLFF | 0 | 0 | 0 | 12 | 0 | 0 | 0 | 0 | 0 | 0 | 0 | 0 | 0 | 0 | 0 | 0 |
| CVVSESGNTPLVF-CASSQDGGFGETQYF | 0 | 0 | 0 | 12 | 0 | 0 | 0 | 0 | 0 | 0 | 0 | 0 | 0 | 0 | 0 | 0 |
| CAGLNGSSNTGKLIF-CASSAHGRNNEQFF | 0 | 0 | 0 | 12 | 0 | 0 | 0 | 0 | 0 | 0 | 0 | 0 | 0 | 0 | 0 | 0 |
| CALRDTGFQKLVF-CASSPLLSDSYNEQFF | 0 | 0 | 0 | 11 | 0 | 0 | 0 | 0 | 0 | 0 | 0 | 0 | 0 | 0 | 0 | 0 |
| CLVGHQTGANNLFF-CASSGRAVAGETQYF | 0 | 0 | 0 | 11 | 0 | 0 | 0 | 0 | 0 | 0 | 0 | 0 | 0 | 0 | 0 | 0 |
| CALSEAYSGGGADGLTF-CAISGTGGTIEQYF | 0 | 0 | 0 | 10 | 0 | 0 | 0 | 0 | 0 | 0 | 0 | 0 | 0 | 0 | 0 | 0 |
| CAASRTGANNLFF-CASSHEDSRQETQYF | 0 | 0 | 0 | 10 | 0 | 0 | 0 | 0 | 0 | 0 | 0 | 0 | 0 | 0 | 0 | 0 |
| CAVKDSNYQLIW-CATSNRADGANVLTF | 0 | 0 | 0 | 9 | 0 | 0 | 0 | 0 | 0 | 0 | 0 | 0 | 0 | 0 | 0 | 0 |
| CAERISDGQKLLF-CASSGTGGSGANVLTF | 0 | 0 | 0 | 8 | 0 | 0 | 0 | 0 | 0 | 0 | 0 | 0 | 0 | 0 | 0 | 0 |
| CADSGGYNKLIF-CASRLLDRAAEQFF | 0 | 0 | 0 | 8 | 0 | 0 | 0 | 0 | 0 | 0 | 0 | 0 | 0 | 0 | 0 | 0 |
| CAVSGRGGYNKLIF-CASSLSTASTDTQYF | 0 | 0 | 0 | 8 | 0 | 0 | 0 | 0 | 0 | 0 | 0 | 0 | 0 | 0 | 0 | 0 |
| CAASLNAGNMLTF-CASSLKDRVTNTEAFF | 0 | 0 | 0 | 8 | 0 | 0 | 0 | 0 | 0 | 0 | 0 | 0 | 0 | 0 | 0 | 0 |
| CAVRDFYQKVTF-CASSFRDRADEQFF | 0 | 0 | 0 | 8 | 0 | 0 | 0 | 0 | 0 | 0 | 0 | 0 | 0 | 0 | 0 | 0 |
| CAFPRGGSNYKLTF-CSARDTQGAGHYEQYF | 0 | 0 | 0 | 7 | 0 | 0 | 0 | 0 | 0 | 0 | 0 | 0 | 0 | 0 | 0 | 0 |
| CAVMDSNYQLIW-CSASHRDYDYNEQFF | 0 | 0 | 0 | 7 | 0 | 0 | 0 | 0 | 0 | 0 | 0 | 0 | 0 | 0 | 0 | 0 |
| CAGYTGFQKLVF-CASSSTRRGPLYEQYF | 0 | 0 | 0 | 0 | 0 | 147 | 0 | 0 | 0 | 0 | 0 | 0 | 0 | 0 | 0 | 0 |
| CLVDRNTGNQFYF-CASSRSGVITDTQYF | 0 | 0 | 0 | 0 | 0 | 75 | 0 | 0 | 0 | 0 | 0 | 0 | 0 | 0 | 0 | 0 |
| CAENNSQGGKLIF-CASSATGFPTEAFF | 0 | 0 | 0 | 0 | 0 | 34 | 0 | 0 | 0 | 0 | 0 | 0 | 0 | 0 | 0 | 0 |
| CVVNSGGYQKVTF-CASSAPNFYEQYF | 0 | 0 | 0 | 0 | 0 | 30 | 0 | 0 | 0 | 0 | 0 | 0 | 0 | 0 | 0 | 0 |
| CAVLDSNYQLIW-CASSQNRDLVGELFF | 0 | 0 | 0 | 0 | 0 | 26 | 0 | 0 | 0 | 0 | 0 | 0 | 0 | 0 | 0 | 0 |
| CAGESGGSYIPTF-CASSPPPGHSNRNRIGTEAFF | 0 | 0 | 0 | 0 | 0 | 16 | 0 | 0 | 0 | 0 | 0 | 0 | 0 | 0 | 0 | 0 |
| CAYDSNYQLIW-CASSSTRRGPLYEQYF | 0 | 0 | 0 | 0 | 0 | 13 | 0 | 0 | 0 | 0 | 0 | 0 | 0 | 0 | 0 | 0 |
| CASLDSNYQLIW-CASSDSHTDTQYF | 0 | 0 | 0 | 0 | 0 | 11 | 0 | 0 | 0 | 0 | 0 | 0 | 0 | 0 | 0 | 0 |
| CAVRDSNYQLIW-CASSHREHTEAFF | 0 | 0 | 0 | 0 | 0 | 7 | 0 | 0 | 0 | 0 | 0 | 0 | 0 | 0 | 0 | 0 |
| CAVRGQYNFNKFYF-CASSEGVGTPFDEQFF | 0 | 0 | 337 | 0 | 0 | 0 | 0 | 0 | 0 | 0 | 0 | 0 | 0 | 0 | 0 | 0 |
| CAMREPADSSYKLIF-CASSYPTSGTPRNEQYF | 0 | 0 | 150 | 0 | 0 | 0 | 0 | 0 | 0 | 0 | 0 | 0 | 0 | 0 | 0 | 0 |
| CASRGGGGSNYKLTF-CSAPGQNYEQYF | 0 | 0 | 85 | 0 | 0 | 0 | 0 | 0 | 0 | 0 | 0 | 0 | 0 | 0 | 0 | 0 |
| CAVRGYRDDKIIF-CASSSRVAGTDTQYF | 0 | 0 | 53 | 0 | 0 | 0 | 0 | 0 | 0 | 0 | 0 | 0 | 0 | 0 | 0 | 0 |
| CAMSSRF-CASSIYGQGYQPQHF | 0 | 0 | 50 | 0 | 0 | 0 | 0 | 0 | 0 | 0 | 0 | 0 | 0 | 0 | 0 | 0 |
| CAMREEYNNNDMRF-CASSSQRAGVYTGELFF | 0 | 0 | 34 | 0 | 0 | 0 | 0 | 0 | 0 | 0 | 0 | 0 | 0 | 0 | 0 | 0 |
| CAVGDFGNEKLTF-CSASRLRIGHGETQYF | 0 | 0 | 32 | 0 | 0 | 0 | 0 | 0 | 0 | 0 | 0 | 0 | 0 | 0 | 0 | 0 |
| CAILNNNDMRF-CSAKTGPPGEKLFF | 0 | 0 | 30 | 0 | 0 | 0 | 0 | 0 | 0 | 0 | 0 | 0 | 0 | 0 | 0 | 0 |
| CAYRSKNRDDKIIF-CASSLRWASVYEQYF | 0 | 0 | 26 | 0 | 0 | 0 | 0 | 0 | 0 | 0 | 0 | 0 | 0 | 0 | 0 | 0 |
| CIVRAYSGGSNYKLTF-CASSYHSNQPQHF | 0 | 0 | 25 | 0 | 0 | 0 | 0 | 0 | 0 | 0 | 0 | 0 | 0 | 0 | 0 | 0 |
| CAVTTPPNAGGTSYGKLTF-CASRHTGAGMWTDTQYF | 0 | 0 | 22 | 0 | 0 | 0 | 0 | 0 | 0 | 0 | 0 | 0 | 0 | 0 | 0 | 0 |
| CAVRPGRDDKIIF-CASSSRLAGGTDTQYF | 0 | 0 | 21 | 0 | 0 | 0 | 0 | 0 | 0 | 0 | 0 | 0 | 0 | 0 | 0 | 0 |
| CAVRPDSNYQLIW-CASSLRTSGLSYEQYF | 0 | 0 | 20 | 0 | 0 | 0 | 0 | 0 | 0 | 0 | 0 | 0 | 0 | 0 | 0 | 0 |
| CAVGYSGAGSYQLTF-CASSISGGLSTDTQYF | 0 | 0 | 20 | 0 | 0 | 0 | 0 | 0 | 0 | 0 | 0 | 0 | 0 | 0 | 0 | 0 |
| CLVGANTNAGKSTF-CASSQDGGGPRQVEQFF | 0 | 0 | 19 | 0 | 0 | 0 | 0 | 0 | 0 | 0 | 0 | 0 | 0 | 0 | 0 | 0 |
| CAGITDYGQNFVF-CSVEGGTLAGHYNEQFF | 0 | 0 | 19 | 0 | 0 | 0 | 0 | 0 | 0 | 0 | 0 | 0 | 0 | 0 | 0 | 0 |
| CVVNGGNTPLVF-CASSLGWRRATGETQYF | 0 | 0 | 18 | 0 | 0 | 0 | 0 | 0 | 0 | 0 | 0 | 0 | 0 | 0 | 0 | 0 |
| CSMEYGNKLVF-CASSLEFGVSNEQYF | 0 | 0 | 18 | 0 | 0 | 0 | 0 | 0 | 0 | 0 | 0 | 0 | 0 | 0 | 0 | 0 |
| CALSDRYTGNQFYF-CASSLVARGATDTQYF | 0 | 0 | 17 | 0 | 0 | 0 | 0 | 0 | 0 | 0 | 0 | 0 | 0 | 0 | 0 | 0 |
| CAYSQETSGSRLTF-CASSVVWWSSQYF | 0 | 0 | 17 | 0 | 0 | 0 | 0 | 0 | 0 | 0 | 0 | 0 | 0 | 0 | 0 | 0 |
| CAAGGSARQLTF-CASSFITYGATGELFF | 0 | 0 | 16 | 0 | 0 | 0 | 0 | 0 | 0 | 0 | 0 | 0 | 0 | 0 | 0 | 0 |
| CAVRAF-CASSSGSLNTEAFF | 0 | 0 | 16 | 0 | 0 | 0 | 0 | 0 | 0 | 0 | 0 | 0 | 0 | 0 | 0 | 0 |
| CLVVPSGGYNKLIF-CASSFFFGVGEQYF | 0 | 0 | 16 | 0 | 0 | 0 | 0 | 0 | 0 | 0 | 0 | 0 | 0 | 0 | 0 | 0 |
| CAYRRLGNTPLVF-CASSAVDGQGFRTDTQYF | 0 | 0 | 15 | 0 | 0 | 0 | 0 | 0 | 0 | 0 | 0 | 0 | 0 | 0 | 0 | 0 |
| CAVGDNFNKFYF-CASSPTSATGELFF | 0 | 0 | 15 | 0 | 0 | 0 | 0 | 0 | 0 | 0 | 0 | 0 | 0 | 0 | 0 | 0 |
| CASTHQAGTALIF-CASSETSGGVKTGELFF | 0 | 0 | 14 | 0 | 0 | 0 | 0 | 0 | 0 | 0 | 0 | 0 | 0 | 0 | 0 | 0 |
| CAVPSGYALNF-CASSPDSSADTQYF | 0 | 0 | 13 | 0 | 0 | 0 | 0 | 0 | 0 | 0 | 0 | 0 | 0 | 0 | 0 | 0 |
| CAVGDNFNKFYF-CASTMTSATGELFF | 0 | 0 | 13 | 0 | 0 | 0 | 0 | 0 | 0 | 0 | 0 | 0 | 0 | 0 | 0 | 0 |
| CLVGEPLNYGGSQGNLIF-CASSLEGAVPQNSYEQYF | 0 | 0 | 13 | 0 | 0 | 0 | 0 | 0 | 0 | 0 | 0 | 0 | 0 | 0 | 0 | 0 |
| CAVIGRGGYQKVTF-CASSLASGGRTYEQYF | 0 | 0 | 12 | 0 | 0 | 0 | 0 | 0 | 0 | 0 | 0 | 0 | 0 | 0 | 0 | 0 |
| CAMRLNRDDKIIF-CASSRRGPRPDEQYF | 0 | 0 | 12 | 0 | 0 | 0 | 0 | 0 | 0 | 0 | 0 | 0 | 0 | 0 | 0 | 0 |
| CAGRTGNQFYF-CASSLLAAPYEQYF | 0 | 0 | 11 | 0 | 0 | 0 | 0 | 0 | 0 | 0 | 0 | 0 | 0 | 0 | 0 | 0 |
| CAVEAAGNKLTF-CASSLISGRSETQYF | 0 | 0 | 11 | 0 | 0 | 0 | 0 | 0 | 0 | 0 | 0 | 0 | 0 | 0 | 0 | 0 |
| CAVGAATDKLIF-CSASPQSYEQYF | 0 | 0 | 11 | 0 | 0 | 0 | 0 | 0 | 0 | 0 | 0 | 0 | 0 | 0 | 0 | 0 |
| CAVRGTGTASKLTF-CSGRAGGNEQFF | 0 | 0 | 11 | 0 | 0 | 0 | 0 | 0 | 0 | 0 | 0 | 0 | 0 | 0 | 0 | 0 |
| CATDEVGGNEKLTF-CASSRRGPRPDEQYF | 0 | 0 | 10 | 0 | 0 | 0 | 0 | 0 | 0 | 0 | 0 | 0 | 0 | 0 | 0 | 0 |
| CALSELYQAGTALIF-CASSGGRGDEKLFF | 0 | 0 | 10 | 0 | 0 | 0 | 0 | 0 | 0 | 0 | 0 | 0 | 0 | 0 | 0 | 0 |
| CATDGGGADGLTF-CSAREGSGSEKYF | 0 | 0 | 10 | 0 | 0 | 0 | 0 | 0 | 0 | 0 | 0 | 0 | 0 | 0 | 0 | 0 |
| CVVRAYRDDKIIF-CASSSRLAGGITDTQYF | 0 | 0 | 10 | 0 | 0 | 0 | 0 | 0 | 0 | 0 | 0 | 0 | 0 | 0 | 0 | 0 |
| CAVNRNYGGSQGNLIF-CASGDGTGELFF | 0 | 0 | 10 | 0 | 0 | 0 | 0 | 0 | 0 | 0 | 0 | 0 | 0 | 0 | 0 | 0 |
| CAFYSNSGYALNF-CASEKGLGAYEQYF | 0 | 0 | 9 | 0 | 0 | 0 | 0 | 0 | 0 | 0 | 0 | 0 | 0 | 0 | 0 | 0 |
| CAVYWGQKLLF-CASTDRDSSNIYEQYF | 0 | 0 | 9 | 0 | 0 | 0 | 0 | 0 | 0 | 0 | 0 | 0 | 0 | 0 | 0 | 0 |
| CAASENYGQNFVF-CASSSRIQGGSYEQYF | 0 | 0 | 9 | 0 | 0 | 0 | 0 | 0 | 0 | 0 | 0 | 0 | 0 | 0 | 0 | 0 |
| CAMRENSKLTF-CASSPVAGDHSGNTIYF | 0 | 0 | 9 | 0 | 0 | 0 | 0 | 0 | 0 | 0 | 0 | 0 | 0 | 0 | 0 | 0 |
| CAVGAQAREYGNKLVF-CASSLNSGTYEQYF | 0 | 0 | 9 | 0 | 0 | 0 | 0 | 0 | 0 | 0 | 0 | 0 | 0 | 0 | 0 | 0 |
| CAMSLLKGFGNVLHC-CASSVGERMTEAFF | 0 | 0 | 8 | 0 | 0 | 0 | 0 | 0 | 0 | 0 | 0 | 0 | 0 | 0 | 0 | 0 |
| CAVSDGTNQFYF-CASSYGGRSSYEQYF | 0 | 0 | 8 | 0 | 0 | 0 | 0 | 0 | 0 | 0 | 0 | 0 | 0 | 0 | 0 | 0 |
| CAVSDGQKLLF-CASSHWEEQYF | 0 | 0 | 8 | 0 | 0 | 0 | 0 | 0 | 0 | 0 | 0 | 0 | 0 | 0 | 0 | 0 |
| CAVNSGGGADGLTF-CASSSRTSGRDYEQYF | 0 | 0 | 8 | 0 | 0 | 0 | 0 | 0 | 0 | 0 | 0 | 0 | 0 | 0 | 0 | 0 |
| CAPQTGANNLFF-CASSPGSDYGYTF | 0 | 0 | 8 | 0 | 0 | 0 | 0 | 0 | 0 | 0 | 0 | 0 | 0 | 0 | 0 | 0 |
| CAGREGGSYIPTF-CASSLGVGSYNEQFF | 0 | 0 | 8 | 0 | 0 | 0 | 0 | 0 | 0 | 0 | 0 | 0 | 0 | 0 | 0 | 0 |
| CAFMKHDYKLSF-CASSGQFPSPTDTQYF | 0 | 0 | 7 | 0 | 0 | 0 | 0 | 0 | 0 | 0 | 0 | 0 | 0 | 0 | 0 | 0 |
| CAASGTSGSRLTF-CATSLAGFSYNEQFF | 0 | 0 | 7 | 0 | 0 | 0 | 0 | 0 | 0 | 0 | 0 | 0 | 0 | 0 | 0 | 0 |
| CAASENYGQNFVF-CASSSRIAGGNYEQYF | 0 | 0 | 7 | 0 | 0 | 0 | 0 | 0 | 0 | 0 | 0 | 0 | 0 | 0 | 0 | 0 |
| CIVVPNFGNEKLTF-CAWSEDRGQGKPEAFF | 0 | 0 | 0 | 0 | 0 | 0 | 0 | 60 | 0 | 0 | 0 | 0 | 0 | 0 | 0 | 0 |
| CFYGGATNKLIF-CASSLERGRDEQYF | 0 | 0 | 0 | 0 | 0 | 0 | 0 | 46 | 0 | 0 | 0 | 0 | 0 | 0 | 0 | 0 |
| CAVRYTGFQKLVF-CASSRESASRVGYGYTF | 0 | 0 | 0 | 0 | 0 | 0 | 0 | 11 | 0 | 0 | 0 | 0 | 0 | 0 | 0 | 0 |
| CERSGSNYQLIW-CASSRESASRVGYGYTF | 0 | 0 | 0 | 0 | 0 | 0 | 0 | 10 | 0 | 0 | 0 | 0 | 0 | 0 | 0 | 0 |
| CAVRPYRDDKIIF-CASSSRLAGGSYEQYF | 0 | 0 | 0 | 0 | 0 | 0 | 0 | 8 | 0 | 0 | 0 | 0 | 0 | 0 | 0 | 0 |
| CAMREDTGRRALTF-CASSLGQGAANTEAFF | 0 | 0 | 0 | 0 | 0 | 0 | 0 | 6 | 0 | 0 | 0 | 0 | 0 | 0 | 0 | 0 |
| CAGPISASKIIF-CAWSLRGDAEQYF | 0 | 0 | 0 | 0 | 0 | 0 | 0 | 6 | 0 | 0 | 0 | 0 | 0 | 0 | 0 | 0 |
| CALSDMDSNYQLIW-CASSTDLGRGSTEAFF | 0 | 0 | 0 | 0 | 0 | 0 | 0 | 0 | 0 | 0 | 0 | 0 | 134 | 0 | 0 | 0 |
| CAVNPRNDYKLSF-CASSEQIEQYF | 0 | 0 | 0 | 0 | 0 | 0 | 0 | 0 | 0 | 0 | 0 | 0 | 18 | 0 | 0 | 0 |
| CAVDMWDMRF-CATSDPRQGVGTGELFF | 0 | 0 | 0 | 0 | 0 | 0 | 0 | 0 | 0 | 0 | 0 | 0 | 9 | 0 | 0 | 0 |
| CAAGTTGNQFYF-CASSQWAANTEAFF | 0 | 0 | 0 | 0 | 0 | 0 | 0 | 0 | 0 | 0 | 0 | 0 | 9 | 0 | 0 | 0 |
| CAAIANDYKLSF-CASSLGSTPNEQFF | 0 | 0 | 0 | 0 | 0 | 0 | 0 | 0 | 0 | 0 | 0 | 0 | 8 | 0 | 0 | 0 |
| CAVAANTDKLIF-CSASPASYEQYF | 0 | 0 | 0 | 0 | 0 | 0 | 0 | 0 | 0 | 0 | 0 | 0 | 8 | 0 | 0 | 0 |
| CAVGTGGFKTIF-CASSPTGPRGGGTEAFF | 0 | 0 | 0 | 0 | 0 | 0 | 0 | 0 | 0 | 0 | 0 | 0 | 7 | 0 | 0 | 0 |
| CAVRDGRAAGNKLTF-CATSDFKGPDGGVTQYF | 0 | 0 | 0 | 0 | 0 | 0 | 0 | 0 | 0 | 0 | 0 | 0 | 7 | 0 | 0 | 0 |
| CAVSGRTDKLIF-CSASPSSYEQYF | 0 | 0 | 0 | 0 | 0 | 0 | 0 | 0 | 0 | 0 | 0 | 0 | 7 | 0 | 0 | 0 |
| CAMDSNYQLIW-CASGWESPLHF | 0 | 0 | 0 | 0 | 0 | 0 | 0 | 0 | 0 | 0 | 0 | 0 | 6 | 0 | 0 | 0 |
| CAATPPASNFGNEKLTF-CASSRKWTSGDQETQYF | 0 | 0 | 0 | 0 | 0 | 0 | 0 | 0 | 98 | 0 | 0 | 0 | 0 | 0 | 0 | 0 |
| CILRDNFGNEKLTF-CASSRKWTSGDQETQYF | 0 | 0 | 0 | 0 | 0 | 0 | 0 | 0 | 71 | 0 | 0 | 0 | 0 | 0 | 0 | 0 |
| CAVRDQNAGNMLTF-CASSLNLGQGMNQPQHF | 0 | 0 | 0 | 0 | 0 | 0 | 0 | 0 | 48 | 0 | 0 | 0 | 0 | 0 | 0 | 0 |
| CAASGGYNKLIF-CASSLQREQYF | 0 | 0 | 0 | 0 | 0 | 0 | 0 | 0 | 38 | 0 | 0 | 0 | 0 | 0 | 0 | 0 |
| CAVVDSNYQLIW-CASSHADSSSGANVLTF | 0 | 0 | 0 | 0 | 0 | 0 | 0 | 0 | 29 | 0 | 0 | 0 | 0 | 0 | 0 | 0 |
| CAMSAWEGAQKLVF-CASSYKGGVTYNEQFF | 0 | 0 | 0 | 0 | 0 | 0 | 0 | 0 | 27 | 0 | 0 | 0 | 0 | 0 | 0 | 0 |
| CAMRGINTGNQFYF-CASSPTLGPRAGTDTQYF | 0 | 0 | 0 | 0 | 0 | 0 | 0 | 0 | 20 | 0 | 0 | 0 | 0 | 0 | 0 | 0 |
| CAVRDPRPGRKAYLRT-CASSHADSSSGANVLTF | 0 | 0 | 0 | 0 | 0 | 0 | 0 | 0 | 18 | 0 | 0 | 0 | 0 | 0 | 0 | 0 |
| CALGSNYQLIW-CASSYRGGNTIYF | 0 | 0 | 0 | 0 | 0 | 0 | 0 | 0 | 17 | 0 | 0 | 0 | 0 | 0 | 0 | 0 |
| CIVPQTGANNLFF-CASSTPSQGTEAFF | 0 | 0 | 0 | 0 | 0 | 0 | 0 | 0 | 16 | 0 | 0 | 0 | 0 | 0 | 0 | 0 |
| CILRDVGGSQGNLIF-CASSLAVATGELFF | 0 | 0 | 0 | 0 | 0 | 0 | 0 | 0 | 15 | 0 | 0 | 0 | 0 | 0 | 0 | 0 |
| CALSEGSGTYKYIF-CASSGSGTSGDEEFF | 0 | 0 | 0 | 0 | 0 | 0 | 0 | 0 | 12 | 0 | 0 | 0 | 0 | 0 | 0 | 0 |
| CALEADAGGTSYGKLTF-CASSPKIEAFF | 0 | 0 | 0 | 0 | 0 | 0 | 0 | 0 | 12 | 0 | 0 | 0 | 0 | 0 | 0 | 0 |
| CAVVNQMDSNYQLIW-CASSQGGTGVAFF | 0 | 0 | 0 | 0 | 0 | 0 | 0 | 0 | 11 | 0 | 0 | 0 | 0 | 0 | 0 | 0 |
| CAVAYQAGTALIF-CASEGDTQYF | 0 | 0 | 0 | 0 | 0 | 0 | 0 | 0 | 11 | 0 | 0 | 0 | 0 | 0 | 0 | 0 |
| CAVGDPNNAGNMLTF-CASSLAGEETQYF | 0 | 0 | 0 | 0 | 0 | 0 | 0 | 0 | 10 | 0 | 0 | 0 | 0 | 0 | 0 | 0 |
| CALGGMDSSYKLIF-CASRSGDTMNTEAFF | 0 | 0 | 0 | 0 | 0 | 0 | 0 | 0 | 9 | 0 | 0 | 0 | 0 | 0 | 0 | 0 |
| CIRTNFGNEKLTF-CSVEDEAPRVYNEQFF | 0 | 0 | 0 | 0 | 0 | 0 | 0 | 0 | 8 | 0 | 0 | 0 | 0 | 0 | 0 | 0 |
| CALSSDRSGANNLFF-CASSMYSGSSYEQYF | 0 | 0 | 0 | 0 | 0 | 0 | 0 | 0 | 8 | 0 | 0 | 0 | 0 | 0 | 0 | 0 |
| CAMRVLTQGGSEKLVF-CASSPGTVYEQYF | 0 | 0 | 0 | 0 | 0 | 0 | 0 | 0 | 8 | 0 | 0 | 0 | 0 | 0 | 0 | 0 |
| CAVAIPHTGANSKLTF-CASSFGTGGTPYNEQFF | 0 | 0 | 0 | 0 | 0 | 0 | 0 | 0 | 8 | 0 | 0 | 0 | 0 | 0 | 0 | 0 |
| CAVILRDQAGTALIF-CASSLGTGNTEAFF | 0 | 0 | 0 | 0 | 0 | 0 | 0 | 0 | 7 | 0 | 0 | 0 | 0 | 0 | 0 | 0 |
| CAVGALYGNKLVF-CATSGGGNTEAFF | 0 | 0 | 0 | 0 | 0 | 0 | 0 | 0 | 7 | 0 | 0 | 0 | 0 | 0 | 0 | 0 |
| CAVMDSNYQLIW-CASSDSTSGTDTQYF | 0 | 0 | 0 | 0 | 0 | 0 | 0 | 0 | 7 | 0 | 0 | 0 | 0 | 0 | 0 | 0 |
| CAVRDIGSGGYQKVTF-CASSQDPGSTGELFF | 0 | 0 | 0 | 0 | 0 | 0 | 0 | 0 | 7 | 0 | 0 | 0 | 0 | 0 | 0 | 0 |
| CAFTLIQGAQKLVF-CASSHRVTEAFF | 0 | 0 | 0 | 0 | 0 | 0 | 0 | 0 | 7 | 0 | 0 | 0 | 0 | 0 | 0 | 0 |
| CAVRDRGSQGNLIF-CASSPQGGTEAFF | 0 | 0 | 0 | 0 | 0 | 0 | 0 | 0 | 7 | 0 | 0 | 0 | 0 | 0 | 0 | 0 |
| CALSEAYRDDKIIF-CASSSPSGTGSSYNEQFF | 0 | 0 | 0 | 0 | 0 | 0 | 0 | 0 | 7 | 0 | 0 | 0 | 0 | 0 | 0 | 0 |
| CAMRGGSGTYKYIF-CASSQDRATDTQYF | 0 | 0 | 0 | 0 | 303 | 0 | 0 | 0 | 0 | 0 | 0 | 0 | 0 | 0 | 0 | 0 |
| CAGDRDDKIIF-CASSPMVGWTDTQYF | 0 | 0 | 0 | 0 | 134 | 0 | 0 | 0 | 0 | 0 | 0 | 0 | 0 | 0 | 0 | 0 |
| CAGVVASHAGNMLTF-CASSPMVGWTDTQYF | 0 | 0 | 0 | 0 | 131 | 0 | 0 | 0 | 0 | 0 | 0 | 0 | 0 | 0 | 0 | 0 |
| CAASNGNNDMRF-CASSLGLAGDLDEQFF | 0 | 0 | 0 | 0 | 130 | 0 | 0 | 0 | 0 | 0 | 0 | 0 | 0 | 0 | 0 | 0 |
| CAMRERGGGSNYKLTF-CASSTSQGGEQFF | 0 | 0 | 0 | 0 | 94 | 0 | 0 | 0 | 0 | 0 | 0 | 0 | 0 | 0 | 0 | 0 |
| CAVQEAAGNKLTF-CASNQGLAGGRLYNEQFF | 0 | 0 | 0 | 0 | 81 | 0 | 0 | 0 | 0 | 0 | 0 | 0 | 0 | 0 | 0 | 0 |
| CAIQIGDYGQNFVF-CASSVLTSGGQETQYF | 0 | 0 | 0 | 0 | 80 | 0 | 0 | 0 | 0 | 0 | 0 | 0 | 0 | 0 | 0 | 0 |
| CALISNFGNEKLTF-CASSAGQVQETQYF | 0 | 0 | 0 | 0 | 73 | 0 | 0 | 0 | 0 | 0 | 0 | 0 | 0 | 0 | 0 | 0 |
| CAVGLSGSARQLTF-CASSQVPGLAGGLHEQYF | 0 | 0 | 0 | 0 | 56 | 0 | 0 | 0 | 0 | 0 | 0 | 0 | 0 | 0 | 0 | 0 |
| CAVRIWAEDFGNEKLTF-CASSIGADMYEQYF | 0 | 0 | 0 | 0 | 55 | 0 | 0 | 0 | 0 | 0 | 0 | 0 | 0 | 0 | 0 | 0 |
| CAALTSGTYKYIF-CASSLDSVDQETQYF | 0 | 0 | 0 | 0 | 54 | 0 | 0 | 0 | 0 | 0 | 0 | 0 | 0 | 0 | 0 | 0 |
| CAVRDEGSGAGSYQLTF-CASSQDYAGGYEQYF | 0 | 0 | 0 | 0 | 52 | 0 | 0 | 0 | 0 | 0 | 0 | 0 | 0 | 0 | 0 | 0 |
| CAVPTSGTYKYIF-CASSLVPGLAGGLVEQFF | 0 | 0 | 0 | 0 | 46 | 0 | 0 | 0 | 0 | 0 | 0 | 0 | 0 | 0 | 0 | 0 |
| CAEGGGSYIPTF-CASSLTQGAQETQYF | 0 | 0 | 0 | 0 | 45 | 0 | 0 | 0 | 0 | 0 | 0 | 0 | 0 | 0 | 0 | 0 |
| CALSELYSGGGADGLTF-CASSPGLAGDLYEQYF | 0 | 0 | 0 | 0 | 35 | 0 | 0 | 0 | 0 | 0 | 0 | 0 | 0 | 0 | 0 | 0 |
| CVVSDRGSTLGRLYF-CASSEGGKGQPQHF | 0 | 0 | 0 | 0 | 33 | 0 | 0 | 0 | 0 | 0 | 0 | 0 | 0 | 0 | 0 | 0 |
| CAGLFQGGSEKLVF-CASSAWTDDYNEQFF | 0 | 0 | 0 | 0 | 30 | 0 | 0 | 0 | 0 | 0 | 0 | 0 | 0 | 0 | 0 | 0 |
| CIVRGGGSNYKLTF-CASSEDPGGRPQHF | 0 | 0 | 0 | 0 | 28 | 0 | 0 | 0 | 0 | 0 | 0 | 0 | 0 | 0 | 0 | 0 |
| CIVMGGSNYKLTF-CSARDRGSSNSPLHF | 0 | 0 | 0 | 0 | 28 | 0 | 0 | 0 | 0 | 0 | 0 | 0 | 0 | 0 | 0 | 0 |
| CAMSKFTSGTYKYIF-CASSPYDREGGYTF | 0 | 0 | 0 | 0 | 26 | 0 | 0 | 0 | 0 | 0 | 0 | 0 | 0 | 0 | 0 | 0 |
| CIVRVDNTGKLIF-CASRAGPHYEQYF | 0 | 0 | 0 | 0 | 26 | 0 | 0 | 0 | 0 | 0 | 0 | 0 | 0 | 0 | 0 | 0 |
| CASEMKTSYDKVIF-CASSPGLAGDLYEQYF | 0 | 0 | 0 | 0 | 24 | 0 | 0 | 0 | 0 | 0 | 0 | 0 | 0 | 0 | 0 | 0 |
| CALSEYAQGGSEKLVF-CASSAPSGDTYEQYF | 0 | 0 | 0 | 0 | 24 | 0 | 0 | 0 | 0 | 0 | 0 | 0 | 0 | 0 | 0 | 0 |
| CIVRVVRGAGGTSYGKLTF-CSARDRGSSNSPLHF | 0 | 0 | 0 | 0 | 22 | 0 | 0 | 0 | 0 | 0 | 0 | 0 | 0 | 0 | 0 | 0 |
| CIVSGGSNYKLTF-CSVRGRGSTNSPLHF | 0 | 0 | 0 | 0 | 20 | 0 | 0 | 0 | 0 | 0 | 0 | 0 | 0 | 0 | 0 | 0 |
| CAVNFF-CASSTGTSLYNEQFF | 0 | 0 | 0 | 0 | 19 | 0 | 0 | 0 | 0 | 0 | 0 | 0 | 0 | 0 | 0 | 0 |
| CILIYSGAGSYQLTF-CASSTLFFEGQVRGYTF | 0 | 0 | 0 | 0 | 19 | 0 | 0 | 0 | 0 | 0 | 0 | 0 | 0 | 0 | 0 | 0 |
| CLVGSYGGATNKLIF-CASSYHAPGQGNTEAFF | 0 | 0 | 0 | 0 | 19 | 0 | 0 | 0 | 0 | 0 | 0 | 0 | 0 | 0 | 0 | 0 |
| CAASIMGNTPLVF-CASSSGTGVYNEQFF | 0 | 0 | 0 | 0 | 18 | 0 | 0 | 0 | 0 | 0 | 0 | 0 | 0 | 0 | 0 | 0 |
| CAMSQGAQKLVF-CASSPGAGSIVEKLFF | 0 | 0 | 0 | 0 | 17 | 0 | 0 | 0 | 0 | 0 | 0 | 0 | 0 | 0 | 0 | 0 |
| CAVGATSYSGYSTLTF-CASSENRGTYNEQFF | 0 | 0 | 0 | 0 | 16 | 0 | 0 | 0 | 0 | 0 | 0 | 0 | 0 | 0 | 0 | 0 |
| CVAREAGGGNKLTF-CASSQGQGTGYTF | 0 | 0 | 0 | 0 | 16 | 0 | 0 | 0 | 0 | 0 | 0 | 0 | 0 | 0 | 0 | 0 |
| CVVTTTSGTYKYIF-CASSQEAYGYTF | 0 | 0 | 0 | 0 | 15 | 0 | 0 | 0 | 0 | 0 | 0 | 0 | 0 | 0 | 0 | 0 |
| CVVRPGSHALNF-CASSLASGVGTGELFF | 0 | 0 | 0 | 0 | 15 | 0 | 0 | 0 | 0 | 0 | 0 | 0 | 0 | 0 | 0 | 0 |
| CALPSYNTDKLIF-CSVVAGGPRTDTQYF | 0 | 0 | 0 | 0 | 14 | 0 | 0 | 0 | 0 | 0 | 0 | 0 | 0 | 0 | 0 | 0 |
| CAGHPSNTGKLIF-CASSPVPGQEYGYTF | 0 | 0 | 0 | 0 | 14 | 0 | 0 | 0 | 0 | 0 | 0 | 0 | 0 | 0 | 0 | 0 |
| CAVCDYKLSF-CASSSHGQGLDEQFF | 0 | 0 | 0 | 0 | 13 | 0 | 0 | 0 | 0 | 0 | 0 | 0 | 0 | 0 | 0 | 0 |
| CAVKDSNYQLIW-CASGTDDSTDTQYF | 0 | 0 | 0 | 0 | 13 | 0 | 0 | 0 | 0 | 0 | 0 | 0 | 0 | 0 | 0 | 0 |
| CALTGAHSGGYQKVTF-CASSPGSHEQYF | 0 | 0 | 0 | 0 | 13 | 0 | 0 | 0 | 0 | 0 | 0 | 0 | 0 | 0 | 0 | 0 |
| CAVSRQGSYIPTF-CASSSGQGHSTDTQYF | 0 | 0 | 0 | 0 | 12 | 0 | 0 | 0 | 0 | 0 | 0 | 0 | 0 | 0 | 0 | 0 |
| CVVNKGGKLIF-CASSQEVGTSSEQYF | 0 | 0 | 0 | 0 | 11 | 0 | 0 | 0 | 0 | 0 | 0 | 0 | 0 | 0 | 0 | 0 |
| CVVSDSGSARQLTF-CASSLVPGGLPYNEQFF | 0 | 0 | 0 | 0 | 11 | 0 | 0 | 0 | 0 | 0 | 0 | 0 | 0 | 0 | 0 | 0 |
| CAENPLQGAQKLVF-CASTPRGRQGIRGYTF | 0 | 0 | 0 | 0 | 10 | 0 | 0 | 0 | 0 | 0 | 0 | 0 | 0 | 0 | 0 | 0 |
| CATQGAQKLVF-CSVRGRGSTNSPLHF | 0 | 0 | 0 | 0 | 10 | 0 | 0 | 0 | 0 | 0 | 0 | 0 | 0 | 0 | 0 | 0 |
| CAGRSHNTGNQFYF-CASSQDLGQGGTGELFF | 0 | 0 | 0 | 0 | 10 | 0 | 0 | 0 | 0 | 0 | 0 | 0 | 0 | 0 | 0 | 0 |
| CAVRDQYNFNKFYF-CASSQDAGSVPHGYTF | 0 | 0 | 0 | 0 | 10 | 0 | 0 | 0 | 0 | 0 | 0 | 0 | 0 | 0 | 0 | 0 |
| CAVSPGNTGKLIF-CSARDESTGVRDTQYF | 0 | 0 | 0 | 0 | 10 | 0 | 0 | 0 | 0 | 0 | 0 | 0 | 0 | 0 | 0 | 0 |
| CAVEGGVTSGTYKYIF-CAISGPGPGGYYEQYF | 0 | 0 | 0 | 0 | 9 | 0 | 0 | 0 | 0 | 0 | 0 | 0 | 0 | 0 | 0 | 0 |
| CALSGGSNYKLTF-CSARQGNEKLFF | 0 | 0 | 0 | 0 | 9 | 0 | 0 | 0 | 0 | 0 | 0 | 0 | 0 | 0 | 0 | 0 |
| CAVMDSSYKLIF-CASSELAGGPNEQFF | 0 | 0 | 0 | 0 | 9 | 0 | 0 | 0 | 0 | 0 | 0 | 0 | 0 | 0 | 0 | 0 |
| CALSEEYYGGSQGNLIF-CATSDSDRGRDEQYF | 0 | 0 | 0 | 0 | 8 | 0 | 0 | 0 | 0 | 0 | 0 | 0 | 0 | 0 | 0 | 0 |
| CAVLLWKAAGNKLTF-CSARDGGSGSAREQFF | 0 | 0 | 0 | 0 | 8 | 0 | 0 | 0 | 0 | 0 | 0 | 0 | 0 | 0 | 0 | 0 |
| CAVSRDRDDKIIF-CASSQVIQVNYGYTF | 0 | 0 | 0 | 0 | 8 | 0 | 0 | 0 | 0 | 0 | 0 | 0 | 0 | 0 | 0 | 0 |
| CVVSVRMDSSYKLIF-CASSSLSGGGGNEQFF | 0 | 0 | 0 | 0 | 7 | 0 | 0 | 0 | 0 | 0 | 0 | 0 | 0 | 0 | 0 | 0 |
| CAVRPFNTGFQKLVF-CSALPGQGGNSPLHF | 0 | 0 | 0 | 0 | 7 | 0 | 0 | 0 | 0 | 0 | 0 | 0 | 0 | 0 | 0 | 0 |
| CAVGRGSNYKLTF-CASSFGSQPQHF | 0 | 0 | 0 | 0 | 7 | 0 | 0 | 0 | 0 | 0 | 0 | 0 | 0 | 0 | 0 | 0 |
| CAVNSFTSGTYKYIF-CASSLAGDPYNEQFF | 0 | 0 | 0 | 0 | 7 | 0 | 0 | 0 | 0 | 0 | 0 | 0 | 0 | 0 | 0 | 0 |
| CAYRSPTSGTYKYIF-CASSAGQERAFF | 0 | 0 | 0 | 0 | 7 | 0 | 0 | 0 | 0 | 0 | 0 | 0 | 0 | 0 | 0 | 0 |
| CAVRDWDSNYQLIW-CASIVDTEAFF | 0 | 0 | 0 | 0 | 7 | 0 | 0 | 0 | 0 | 0 | 0 | 0 | 0 | 0 | 0 | 0 |
| CAASKGNARLMF-CSATSTGSISYEQYF | 0 | 0 | 0 | 0 | 7 | 0 | 0 | 0 | 0 | 0 | 0 | 0 | 0 | 0 | 0 | 0 |
| CAMRERLGSASKIIF-CSARDTGPYEQYF | 0 | 0 | 0 | 0 | 0 | 0 | 85 | 0 | 0 | 0 | 0 | 0 | 0 | 0 | 0 | 0 |
| CASDNAGNMLTF-CASSPQSQNIQYF | 0 | 0 | 0 | 0 | 0 | 0 | 72 | 0 | 0 | 0 | 0 | 0 | 0 | 0 | 0 | 0 |
| CAENSHFNAGGTSYGKLTF-CASSYSGHPGRAFF | 0 | 0 | 0 | 0 | 0 | 0 | 23 | 0 | 0 | 0 | 0 | 0 | 0 | 0 | 0 | 0 |
| CALPPDGQKLLF-CSARDTGPYEQYF | 0 | 0 | 0 | 0 | 0 | 0 | 22 | 0 | 0 | 0 | 0 | 0 | 0 | 0 | 0 | 0 |
| CALEAGNMLTF-CSVEPDRGRKETQYF | 0 | 0 | 0 | 0 | 0 | 0 | 16 | 0 | 0 | 0 | 0 | 0 | 0 | 0 | 0 | 0 |
| CLVGGYNKLIF-CASSPTTRSTSYNEQFF | 0 | 0 | 0 | 0 | 0 | 0 | 14 | 0 | 0 | 0 | 0 | 0 | 0 | 0 | 0 | 0 |
| CAVYDYKLSF-CASSQERGVYNEQFF | 0 | 0 | 0 | 0 | 0 | 0 | 14 | 0 | 0 | 0 | 0 | 0 | 0 | 0 | 0 | 0 |
| CATDSTSGTYKYIF-CASSLEGASGYTF | 0 | 0 | 0 | 0 | 0 | 0 | 14 | 0 | 0 | 0 | 0 | 0 | 0 | 0 | 0 | 0 |
| CAGEDYGQNFVF-CASSLGWNEQFF | 0 | 0 | 0 | 0 | 0 | 0 | 12 | 0 | 0 | 0 | 0 | 0 | 0 | 0 | 0 | 0 |
| CAVSSSGGYQKVTF-CSARDPRWGEKLFF | 0 | 0 | 0 | 0 | 0 | 0 | 9 | 0 | 0 | 0 | 0 | 0 | 0 | 0 | 0 | 0 |
| CAVSDRTDKLIF-CSASPSSYEQYF | 0 | 0 | 0 | 0 | 0 | 0 | 9 | 0 | 0 | 0 | 0 | 0 | 0 | 0 | 0 | 0 |
| CAVIDLYSGAGSYQLTF-CASSIVGNSGELFF | 0 | 0 | 0 | 0 | 0 | 0 | 9 | 0 | 0 | 0 | 0 | 0 | 0 | 0 | 0 | 0 |
| CAPPLSSGSARQLTF-CASSLGWNEQFF | 0 | 0 | 0 | 0 | 0 | 0 | 8 | 0 | 0 | 0 | 0 | 0 | 0 | 0 | 0 | 0 |
| CAMWSSGGSNYKLTF-CASNLGATGELFF | 0 | 0 | 0 | 0 | 0 | 0 | 8 | 0 | 0 | 0 | 0 | 0 | 0 | 0 | 0 | 0 |
| CAARIQGAQKLVF-CASSPGGAAKNIQYF | 0 | 0 | 0 | 0 | 0 | 0 | 8 | 0 | 0 | 0 | 0 | 0 | 0 | 0 | 0 | 0 |
| CAPQDSNYQLIW-CSAFSGSEHNEQFF | 0 | 0 | 0 | 0 | 0 | 0 | 7 | 0 | 0 | 0 | 0 | 0 | 0 | 0 | 0 | 0 |
| CALVETGANNLFF-CASSLPGMNTEAFF | 0 | 0 | 0 | 0 | 0 | 0 | 0 | 0 | 0 | 0 | 0 | 0 | 0 | 147 | 0 | 0 |
| CASPGAGSYQLTF-CASSLWSATNEKLFF | 0 | 0 | 0 | 0 | 0 | 0 | 0 | 0 | 0 | 0 | 0 | 0 | 0 | 48 | 0 | 0 |
| CAVGDNAGNMLTF-CASSEVWASDHEQYF | 0 | 0 | 0 | 0 | 0 | 0 | 0 | 0 | 0 | 0 | 0 | 0 | 0 | 45 | 0 | 0 |
| CATDRGDYKLSF-CASSLFLSSYNSPLHF | 0 | 0 | 0 | 0 | 0 | 0 | 0 | 0 | 0 | 0 | 0 | 0 | 0 | 43 | 0 | 0 |
| CIVRVQPLNDYKLSF-CASSFGGREAGNTIYF | 0 | 0 | 0 | 0 | 0 | 0 | 0 | 0 | 0 | 0 | 0 | 0 | 0 | 37 | 0 | 0 |
| CALGPDKLIF-CASSPHTSTDTQYF | 0 | 0 | 0 | 0 | 0 | 0 | 0 | 0 | 0 | 0 | 0 | 0 | 0 | 23 | 0 | 0 |
| CALPGPHFNKFYF-CASSDRDRELFF | 0 | 0 | 0 | 0 | 0 | 0 | 0 | 0 | 0 | 0 | 0 | 0 | 0 | 17 | 0 | 0 |
| CAAYTGRRALTF-CASSRQGVLEAFF | 0 | 0 | 0 | 0 | 0 | 0 | 0 | 0 | 0 | 0 | 0 | 0 | 0 | 14 | 0 | 0 |
| CAVGASNDYKLSF-CASSLYRDISYEQYF | 0 | 0 | 0 | 0 | 0 | 0 | 0 | 0 | 0 | 0 | 0 | 0 | 0 | 13 | 0 | 0 |
| CAFATGNQFYF-CASSAVLAGGTDTQYF | 0 | 0 | 0 | 0 | 0 | 0 | 0 | 0 | 0 | 0 | 0 | 0 | 0 | 12 | 0 | 0 |
| CAGQAGTALIF-CASSQGEGGYTF | 0 | 0 | 0 | 0 | 0 | 0 | 0 | 0 | 0 | 0 | 0 | 0 | 0 | 12 | 0 | 0 |
| CAVQAEGNAGNMLTF-CASSFQAGSISGNTIYF | 0 | 0 | 0 | 0 | 0 | 0 | 0 | 0 | 0 | 0 | 0 | 0 | 0 | 12 | 0 | 0 |
| CAVRDEGGSYIPTF-CASSLEWVDTQYF | 0 | 0 | 0 | 0 | 0 | 0 | 0 | 0 | 0 | 0 | 0 | 0 | 0 | 9 | 0 | 0 |
| CAVRGNTDKLIF-CASSFQAGSISGNTIYF | 0 | 0 | 0 | 0 | 0 | 0 | 0 | 0 | 0 | 0 | 0 | 0 | 0 | 9 | 0 | 0 |
| CASGGEYGNKLVF-CATSRDQRGVYGYTF | 0 | 0 | 0 | 0 | 0 | 0 | 0 | 0 | 0 | 0 | 0 | 0 | 0 | 8 | 0 | 0 |
| CALSGTSF-CASSPPSTGTGELFF | 0 | 0 | 0 | 0 | 0 | 0 | 0 | 0 | 0 | 0 | 0 | 0 | 0 | 8 | 0 | 0 |
| CAASQGAQKLVF-CASSQDGRQTSYEQYF | 0 | 0 | 0 | 0 | 0 | 0 | 0 | 0 | 0 | 0 | 0 | 0 | 0 | 7 | 0 | 0 |
| CAVGDNFNKFYF-CASSRTSATGELFF | 0 | 0 | 0 | 0 | 0 | 0 | 0 | 0 | 0 | 0 | 0 | 0 | 0 | 7 | 0 | 0 |
| CAESSGGYQKVTF-CASGFGSGNTIYF | 0 | 0 | 0 | 0 | 0 | 0 | 0 | 0 | 0 | 0 | 0 | 0 | 0 | 7 | 0 | 0 |
| CAESILTGGGNKLTF-CASSQDWTNAYNEQFF | 0 | 0 | 0 | 0 | 0 | 0 | 0 | 0 | 0 | 207 | 0 | 0 | 0 | 0 | 0 | 0 |
| CAVFYNTDKLIF-CASLRTSGGIGELFF | 0 | 0 | 0 | 0 | 0 | 0 | 0 | 0 | 0 | 25 | 0 | 0 | 0 | 0 | 0 | 0 |
| CAASYLSGGSYIPTF-CASSPRLAETQYF | 0 | 0 | 0 | 0 | 0 | 0 | 0 | 0 | 0 | 25 | 0 | 0 | 0 | 0 | 0 | 0 |
| CIVRVATGRNDMRF-CASSPSLAGSYEQYF | 0 | 0 | 0 | 0 | 0 | 0 | 0 | 0 | 0 | 15 | 0 | 0 | 0 | 0 | 0 | 0 |
| CAVQQGGSEKLVF-CASIPLGGFREKLFF | 0 | 0 | 0 | 0 | 0 | 0 | 0 | 0 | 0 | 14 | 0 | 0 | 0 | 0 | 0 | 0 |
| CAVNTGGFKTIF-CASSPPGPSGSVVYEQYF | 0 | 0 | 0 | 0 | 0 | 0 | 0 | 0 | 0 | 13 | 0 | 0 | 0 | 0 | 0 | 0 |
| CAENTGYGGSQGNLIF-CASSFTAYNEQFF | 0 | 0 | 0 | 0 | 0 | 0 | 0 | 0 | 0 | 12 | 0 | 0 | 0 | 0 | 0 | 0 |
| CAMRDNAGNMLTF-CASGTAFSENTIYF | 0 | 0 | 0 | 0 | 0 | 0 | 0 | 0 | 0 | 11 | 0 | 0 | 0 | 0 | 0 | 0 |
| CAMSDWGFGNEKLTF-CASSYGGGEQYF | 0 | 0 | 0 | 0 | 0 | 0 | 0 | 0 | 0 | 0 | 81 | 0 | 0 | 0 | 0 | 0 |
| CAMSDWGFGNEKLTF | 0 | 0 | 0 | 0 | 0 | 0 | 0 | 0 | 0 | 0 | 75 | 0 | 0 | 0 | 0 | 0 |
| CASSYGGGEQYF | 0 | 0 | 0 | 0 | 0 | 0 | 0 | 0 | 0 | 0 | 72 | 0 | 0 | 0 | 0 | 0 |
| CATDKGSYIPTF-CASSVGSGSDTEAFF | 0 | 0 | 0 | 0 | 0 | 0 | 0 | 0 | 0 | 0 | 59 | 0 | 0 | 0 | 0 | 0 |
| CASSFTGGLAAASYEQYF | 0 | 0 | 0 | 0 | 0 | 0 | 0 | 0 | 0 | 0 | 55 | 0 | 0 | 0 | 0 | 0 |
| CAARRTGTASKLTF-CASSERRQTNQPQHF | 0 | 0 | 0 | 0 | 0 | 0 | 0 | 0 | 0 | 0 | 52 | 0 | 0 | 0 | 0 | 0 |
| CASSERRQTNQPQHF | 0 | 0 | 0 | 0 | 0 | 0 | 0 | 0 | 0 | 0 | 25 | 0 | 0 | 0 | 0 | 0 |
| CAMRENYGQNFVF-CASSLELGLAGVNQETQYF | 0 | 0 | 0 | 0 | 0 | 0 | 0 | 0 | 0 | 0 | 25 | 0 | 0 | 0 | 0 | 0 |
| CASSSQPRPQHF | 0 | 0 | 0 | 0 | 0 | 0 | 0 | 0 | 0 | 0 | 25 | 0 | 0 | 0 | 0 | 0 |
| CAFRPSGTYKYIF-CASSFTGGLAAASYEQYF | 0 | 0 | 0 | 0 | 0 | 0 | 0 | 0 | 0 | 0 | 24 | 0 | 0 | 0 | 0 | 0 |
| CASSLESSYNSPLHF | 0 | 0 | 0 | 0 | 0 | 0 | 0 | 0 | 0 | 0 | 23 | 0 | 0 | 0 | 0 | 0 |
| CASSLELGLAGVNQETQYF | 0 | 0 | 0 | 0 | 0 | 0 | 0 | 0 | 0 | 0 | 23 | 0 | 0 | 0 | 0 | 0 |
| CASSGTSGGPHTGELFF | 0 | 0 | 0 | 0 | 0 | 0 | 0 | 0 | 0 | 0 | 23 | 0 | 0 | 0 | 0 | 0 |
| CAVETYNTDKLIF-CASSSQPRPQHF | 0 | 0 | 0 | 0 | 0 | 0 | 0 | 0 | 0 | 0 | 20 | 0 | 0 | 0 | 0 | 0 |
| CASSVGSGSDTEAFF | 0 | 0 | 0 | 0 | 0 | 0 | 0 | 0 | 0 | 0 | 20 | 0 | 0 | 0 | 0 | 0 |
| CVVRAYNFNKFYF-CATSRDSPVPYEQYF | 0 | 0 | 0 | 0 | 0 | 0 | 0 | 0 | 0 | 0 | 19 | 0 | 0 | 0 | 0 | 0 |
| CASSQELGGAETQYF | 0 | 0 | 0 | 0 | 0 | 0 | 0 | 0 | 0 | 0 | 19 | 0 | 0 | 0 | 0 | 0 |
| CAVNTNAGKSTF-CASSHEQGFSNYGYTF | 0 | 0 | 0 | 0 | 0 | 0 | 0 | 0 | 0 | 0 | 18 | 0 | 0 | 0 | 0 | 0 |
| CAAYNTNAGKSTF-CASSQGPPGQGVHEQYF | 0 | 0 | 0 | 0 | 0 | 0 | 0 | 0 | 0 | 0 | 17 | 0 | 0 | 0 | 0 | 0 |
| CAVQVNYGQNFVF-CASRPGLRTEAFF | 0 | 0 | 0 | 0 | 0 | 0 | 0 | 0 | 0 | 0 | 17 | 0 | 0 | 0 | 0 | 0 |
| CAVILFNNAGNMLTF-CASSLRDRWAFF | 0 | 0 | 0 | 0 | 0 | 0 | 0 | 0 | 0 | 0 | 15 | 0 | 0 | 0 | 0 | 0 |
| CAVMDSNYQLIW-CASSGTSGGPHTGELFF | 0 | 0 | 0 | 0 | 0 | 0 | 0 | 0 | 0 | 0 | 15 | 0 | 0 | 0 | 0 | 0 |
| CAAALPDGGATNKLIF-CASSHEKGEQYF | 0 | 0 | 0 | 0 | 0 | 0 | 0 | 0 | 0 | 0 | 14 | 0 | 0 | 0 | 0 | 0 |
| CAARRTGTASKLTF | 0 | 0 | 0 | 0 | 0 | 0 | 0 | 0 | 0 | 0 | 14 | 0 | 0 | 0 | 0 | 0 |
| CASSHEKGEQYF | 0 | 0 | 0 | 0 | 0 | 0 | 0 | 0 | 0 | 0 | 14 | 0 | 0 | 0 | 0 | 0 |
| CAGYNFNKFYF-CATSRDSPVPYEQYF | 0 | 0 | 0 | 0 | 0 | 0 | 0 | 0 | 0 | 0 | 14 | 0 | 0 | 0 | 0 | 0 |
| CAVRDGGAGTALIF-CASSFRTGAYGYTF | 0 | 0 | 0 | 0 | 0 | 0 | 0 | 0 | 0 | 0 | 13 | 0 | 0 | 0 | 0 | 0 |
| CASSQTSGGADNEQFF | 0 | 0 | 0 | 0 | 0 | 0 | 0 | 0 | 0 | 0 | 13 | 0 | 0 | 0 | 0 | 0 |
| CASSHRGYTNEKLFF | 0 | 0 | 0 | 0 | 0 | 0 | 0 | 0 | 0 | 0 | 12 | 0 | 0 | 0 | 0 | 0 |
| CAMREETAGSYQLTF-CASSPAENTEAFF | 0 | 0 | 0 | 0 | 0 | 0 | 0 | 0 | 0 | 0 | 12 | 0 | 0 | 0 | 0 | 0 |
| CASSPAENTEAFF | 0 | 0 | 0 | 0 | 0 | 0 | 0 | 0 | 0 | 0 | 12 | 0 | 0 | 0 | 0 | 0 |
| CASSIPGGGSSYNEQFF | 0 | 0 | 0 | 0 | 0 | 0 | 0 | 0 | 0 | 0 | 12 | 0 | 0 | 0 | 0 | 0 |
| CAGPRRKGQGFSFIF-CSARRTGHSNQPQHF | 0 | 0 | 0 | 0 | 0 | 0 | 0 | 0 | 0 | 0 | 12 | 0 | 0 | 0 | 0 | 0 |
| CAGGFSGYALNF-CASSIPGGGSSYNEQFF | 0 | 0 | 0 | 0 | 0 | 0 | 0 | 0 | 0 | 0 | 11 | 0 | 0 | 0 | 0 | 0 |
| CAVGALNNNDMRF-CASSDRQGAGGELFF | 0 | 0 | 0 | 0 | 0 | 0 | 0 | 0 | 0 | 0 | 11 | 0 | 0 | 0 | 0 | 0 |
| CASTTPPRGRGTEAFF | 0 | 0 | 0 | 0 | 0 | 0 | 0 | 0 | 0 | 0 | 11 | 0 | 0 | 0 | 0 | 0 |
| CALRNTGGFKTIF-CASSHRGYTNEKLFF | 0 | 0 | 0 | 0 | 0 | 0 | 0 | 0 | 0 | 0 | 10 | 0 | 0 | 0 | 0 | 0 |
| CAVHSSGSARQLTF-CASSQTSGGADNEQFF | 0 | 0 | 0 | 0 | 0 | 0 | 0 | 0 | 0 | 0 | 10 | 0 | 0 | 0 | 0 | 0 |
| CAVQVNYGQNFVF | 0 | 0 | 0 | 0 | 0 | 0 | 0 | 0 | 0 | 0 | 10 | 0 | 0 | 0 | 0 | 0 |
| CAAFTSGTYKYIF-CARRAGANVLTF | 0 | 0 | 0 | 0 | 0 | 0 | 0 | 0 | 0 | 0 | 0 | 212 | 0 | 0 | 0 | 0 |
| CARRAGANVLTF | 0 | 0 | 0 | 0 | 0 | 0 | 0 | 0 | 0 | 0 | 0 | 101 | 0 | 0 | 0 | 0 |
| CVVMVQAGTALIF-CASSSSRDRDGAFF | 0 | 0 | 0 | 0 | 0 | 0 | 0 | 0 | 0 | 0 | 0 | 52 | 0 | 0 | 0 | 0 |
| CAVKRYSGGGADGLTF-CSASHIEPSTRWRLGLPLKQYF | 0 | 0 | 0 | 0 | 0 | 0 | 0 | 0 | 0 | 0 | 0 | 37 | 0 | 0 | 0 | 0 |
| CASSSGQDYEQYF | 0 | 0 | 0 | 0 | 0 | 0 | 0 | 0 | 0 | 0 | 0 | 36 | 0 | 0 | 0 | 0 |
| CVVTSTSGTYKYIF-CSASQFTNNYGYTF | 0 | 0 | 0 | 0 | 0 | 0 | 0 | 0 | 0 | 0 | 0 | 34 | 0 | 0 | 0 | 0 |
| CSASQFTNNYGYTF | 0 | 0 | 0 | 0 | 0 | 0 | 0 | 0 | 0 | 0 | 0 | 33 | 0 | 0 | 0 | 0 |
| CLVGANTNAGKSTF-CASSSGQDYEQYF | 0 | 0 | 0 | 0 | 0 | 0 | 0 | 0 | 0 | 0 | 0 | 31 | 0 | 0 | 0 | 0 |
| CAVNDPNYGGSQGNLIF-CASSKIQGARDGYTF | 0 | 0 | 0 | 0 | 0 | 0 | 0 | 0 | 0 | 0 | 0 | 30 | 0 | 0 | 0 | 0 |
| CVVSSIEPMEYGNKLVF-CASSYSGRGVDEQFF | 0 | 0 | 0 | 0 | 0 | 0 | 0 | 0 | 0 | 0 | 0 | 30 | 0 | 0 | 0 | 0 |
| CAMRGIYNQGGKLIF-CASSTGTGFSNQPQHF | 0 | 0 | 0 | 0 | 0 | 0 | 0 | 0 | 0 | 0 | 0 | 30 | 0 | 0 | 0 | 0 |
| CASSYSGRGVDEQFF | 0 | 0 | 0 | 0 | 0 | 0 | 0 | 0 | 0 | 0 | 0 | 22 | 0 | 0 | 0 | 0 |
| CVVTLGEDYKLSF-CSARPDREAKNIQYF | 0 | 0 | 0 | 0 | 0 | 0 | 0 | 0 | 0 | 0 | 0 | 21 | 0 | 0 | 0 | 0 |
| CASSEGSNQPQHF | 0 | 0 | 0 | 0 | 0 | 0 | 0 | 0 | 0 | 0 | 0 | 19 | 0 | 0 | 0 | 0 |
| CAMREPGTSYGKLTF-CASSQGGTKSTQYF | 0 | 0 | 0 | 0 | 0 | 0 | 0 | 0 | 0 | 0 | 0 | 17 | 0 | 0 | 0 | 0 |
| CALSEAREGFQKLVF-CASSLLGGNTGELFF | 0 | 0 | 0 | 0 | 0 | 0 | 0 | 0 | 0 | 0 | 0 | 17 | 0 | 0 | 0 | 0 |
| CAVNDPNYGGSQGNLIF | 0 | 0 | 0 | 0 | 0 | 0 | 0 | 0 | 0 | 0 | 0 | 16 | 0 | 0 | 0 | 0 |
| CSATTGEALGTGELFF | 0 | 0 | 0 | 0 | 0 | 0 | 0 | 0 | 0 | 0 | 0 | 15 | 0 | 0 | 0 | 0 |
| CAVRDGDYKLSF-CSATTGEALGTGELFF | 0 | 0 | 0 | 0 | 0 | 0 | 0 | 0 | 0 | 0 | 0 | 13 | 0 | 0 | 0 | 0 |
| CASSSSRDRDGAFF | 0 | 0 | 0 | 0 | 0 | 0 | 0 | 0 | 0 | 0 | 0 | 13 | 0 | 0 | 0 | 0 |
| CSARSNAQRGYTF | 0 | 0 | 0 | 0 | 0 | 0 | 0 | 0 | 0 | 0 | 0 | 13 | 0 | 0 | 0 | 0 |
| CATSDWSGDTQYF | 0 | 0 | 0 | 0 | 0 | 0 | 0 | 0 | 0 | 0 | 0 | 13 | 0 | 0 | 0 | 0 |
| CALSDDNDMRF-CASSGGSGRSQETQYF | 0 | 0 | 0 | 0 | 0 | 0 | 0 | 0 | 0 | 0 | 0 | 12 | 0 | 0 | 0 | 0 |
| CALSLTGANNLFF-CISAPAVRDRENGEQYF-CASSREGYDQPQHF | 0 | 0 | 0 | 0 | 0 | 0 | 0 | 0 | 0 | 0 | 0 | 12 | 0 | 0 | 0 | 0 |
| CALSLTGANNLFF-CASSREGYDQPQHF | 0 | 0 | 0 | 0 | 0 | 0 | 0 | 0 | 0 | 0 | 0 | 12 | 0 | 0 | 0 | 0 |
| CASSLARTIRGNTQYF | 0 | 0 | 0 | 0 | 0 | 0 | 0 | 0 | 0 | 0 | 0 | 12 | 0 | 0 | 0 | 0 |
| CAVVHSGGGADGLTF-CASSLWRTETQYF | 0 | 0 | 0 | 0 | 0 | 0 | 0 | 0 | 0 | 0 | 0 | 11 | 0 | 0 | 0 | 0 |
| CASSREGYDQPQHF | 0 | 0 | 0 | 0 | 0 | 0 | 0 | 0 | 0 | 0 | 0 | 11 | 0 | 0 | 0 | 0 |
| CSASHIEPSTRWRLGLPLKQYF | 0 | 0 | 0 | 0 | 0 | 0 | 0 | 0 | 0 | 0 | 0 | 11 | 0 | 0 | 0 | 0 |
| CAVMDSNYQLIW-CASSEGSNQPQHF | 0 | 0 | 0 | 0 | 0 | 0 | 0 | 0 | 0 | 0 | 0 | 10 | 0 | 0 | 0 | 0 |
| CASSLLGGNTGELFF | 0 | 0 | 0 | 0 | 0 | 0 | 0 | 0 | 0 | 0 | 0 | 10 | 0 | 0 | 0 | 0 |
| CASSTGTGFSNQPQHF | 0 | 0 | 0 | 0 | 0 | 0 | 0 | 0 | 0 | 0 | 0 | 10 | 0 | 0 | 0 | 0 |
| CASSGLNSPLHF | 0 | 0 | 0 | 0 | 0 | 0 | 0 | 0 | 0 | 0 | 0 | 10 | 0 | 0 | 0 | 0 |
| CASKGINTEAFF | 0 | 0 | 0 | 0 | 0 | 0 | 0 | 0 | 0 | 0 | 0 | 10 | 0 | 0 | 0 | 0 |
| CAATNTGNQFYF-CASSYWTSRTDTQYF | 0 | 0 | 0 | 0 | 0 | 0 | 0 | 0 | 0 | 0 | 0 | 10 | 0 | 0 | 0 | 0 |
| CAASYTGNQFYF-CATSHAAGRLYEQYF | 0 | 0 | 0 | 0 | 0 | 0 | 0 | 0 | 0 | 0 | 0 | 10 | 0 | 0 | 0 | 0 |
| CASSSGTGYYEQYF | 0 | 0 | 0 | 0 | 0 | 0 | 0 | 0 | 0 | 0 | 0 | 0 | 0 | 0 | 0 | 13 |
| CASSFVPGANTGELFF | 0 | 0 | 0 | 0 | 0 | 0 | 0 | 0 | 0 | 0 | 0 | 0 | 0 | 0 | 0 | 9 |
| CASGTGYNEQFF | 0 | 0 | 0 | 0 | 0 | 0 | 0 | 0 | 0 | 0 | 0 | 0 | 0 | 0 | 0 | 8 |
| CAATLYGNNRLAF | 0 | 0 | 0 | 0 | 0 | 0 | 0 | 0 | 0 | 0 | 0 | 0 | 0 | 0 | 0 | 7 |
| CAVGDTGFQKLVF-CASSSGTGYYEQYF | 0 | 0 | 0 | 0 | 0 | 0 | 0 | 0 | 0 | 0 | 0 | 0 | 0 | 0 | 0 | 7 |
| CASGGADGLTF | 0 | 0 | 0 | 0 | 0 | 0 | 0 | 0 | 0 | 0 | 0 | 0 | 0 | 0 | 0 | 6 |
| CAATLYGNNRLAF-CASSVAGLNNEQFF | 0 | 0 | 0 | 0 | 0 | 0 | 0 | 0 | 0 | 0 | 0 | 0 | 0 | 0 | 0 | 6 |
| CASSVAGLNNEQFF | 0 | 0 | 0 | 0 | 0 | 0 | 0 | 0 | 0 | 0 | 0 | 0 | 0 | 0 | 0 | 6 |
| CAVIGGYSTLTF-CASSFVPGANTGELFF | 0 | 0 | 0 | 0 | 0 | 0 | 0 | 0 | 0 | 0 | 0 | 0 | 0 | 0 | 0 | 5 |
| CAVGSSNTGKLIF-CASSATSGGYNEQFF | 0 | 0 | 0 | 0 | 0 | 0 | 0 | 0 | 0 | 0 | 0 | 0 | 0 | 0 | 0 | 5 |
| CAVRDSNYQLIW | 0 | 0 | 0 | 0 | 0 | 0 | 0 | 0 | 0 | 0 | 0 | 0 | 0 | 0 | 0 | 5 |
| CAVLGRASGGYQKVTF-CASSPLLLDEQYF | 0 | 0 | 0 | 0 | 0 | 0 | 0 | 0 | 0 | 0 | 0 | 0 | 0 | 0 | 0 | 5 |
| CASGGADGLTF-CASSVATGTDTQYF | 0 | 0 | 0 | 0 | 0 | 0 | 0 | 0 | 0 | 0 | 0 | 0 | 0 | 0 | 0 | 4 |
| CVVNGDLTGRRALTF-CASSQDRLGGYEQYF | 0 | 0 | 0 | 0 | 0 | 0 | 0 | 0 | 0 | 0 | 0 | 0 | 0 | 0 | 0 | 4 |
| CASSVATGTDTQYF | 0 | 0 | 0 | 0 | 0 | 0 | 0 | 0 | 0 | 0 | 0 | 0 | 0 | 0 | 0 | 4 |
| CAATYNTDKLIF-CASSPLVIGYNEQFF | 0 | 0 | 0 | 0 | 0 | 0 | 0 | 0 | 0 | 0 | 0 | 0 | 0 | 0 | 36 | 0 |
| CAVRSGMKTSYDKVIF-CASSLQPITEAFF | 0 | 0 | 0 | 0 | 0 | 0 | 0 | 0 | 0 | 0 | 0 | 0 | 0 | 0 | 24 | 0 |
| CAERDGGFKTIF;CAVNGNTGGFKTIF-CASSQRGSGDTQYF | 0 | 0 | 0 | 0 | 0 | 0 | 0 | 0 | 0 | 0 | 0 | 0 | 0 | 0 | 23 | 0 |
| CILGSDAGNMLTF-CASSPGQKNQPQHF | 0 | 0 | 0 | 0 | 0 | 0 | 0 | 0 | 0 | 0 | 0 | 0 | 0 | 0 | 15 | 0 |
| CAATPRGTGGGNKLTF-CASTFGSYAMNTEAFF | 0 | 0 | 0 | 0 | 0 | 0 | 0 | 0 | 0 | 0 | 0 | 0 | 0 | 0 | 13 | 0 |
| CVVNVLGGYQKVTF-CATKWGGSEDTQYF | 0 | 0 | 0 | 0 | 0 | 0 | 0 | 0 | 0 | 0 | 0 | 0 | 0 | 0 | 13 | 0 |
| CATAFTGGGNKLTF-CASSPPGGGEKLFF | 0 | 0 | 0 | 0 | 0 | 0 | 0 | 0 | 0 | 0 | 0 | 0 | 0 | 0 | 12 | 0 |
| CAWSGGGYSPLHF | 0 | 0 | 0 | 0 | 0 | 0 | 0 | 0 | 0 | 0 | 0 | 0 | 0 | 0 | 12 | 0 |
| CAASAGGFKTIF-CASSQPSTGYNEKLFF | 0 | 0 | 0 | 0 | 0 | 0 | 0 | 0 | 0 | 0 | 0 | 0 | 0 | 0 | 11 | 0 |
| CAALDYYGGATNKLIF-CAWSGQFTEAFF | 0 | 0 | 0 | 0 | 0 | 0 | 0 | 0 | 0 | 0 | 0 | 0 | 0 | 0 | 11 | 0 |

**Table S8 List of the most enriched V(D)J clonotypic B cells in each patient by BCR single-cell transcriptome analysis.**

| **IGLorK_CDR3_IGH_CDR3** | **Patient1** | **Patient2** | **Patient3** | **Patient4** | **Patient5** | **Patient6** | **Patient7** | **Patient8** | **Patient9** | **Patient10** | **Patient11** | **Patient12** | **Patient13** | **Patient14** | **Patient15** | **Patient16** |
| --- | --- | --- | --- | --- | --- | --- | --- | --- | --- | --- | --- | --- | --- | --- | --- | --- |
| CQAGDSSTHVIL_CAGNTGYYYYYGMDVW | 4 | 0 | 0 | 0 | 0 | 0 | 0 | 0 | 0 | 0 | 0 | 0 | 0 | 0 | 0 | 0 |
| CQAGDNNIHVVL_CAGNTGYYYYYGLDVW | 3 | 0 | 0 | 0 | 0 | 0 | 0 | 0 | 0 | 0 | 0 | 0 | 0 | 0 | 0 | 0 |
| CQTWGTGLQVF_CAREWADSTSSAFDYW | 3 | 0 | 0 | 0 | 0 | 0 | 0 | 0 | 0 | 0 | 0 | 0 | 0 | 0 | 0 | 0 |
| CMQATQFPLTF_CVREVYGDPFDYW | 0 | 0 | 0 | 14 | 0 | 0 | 0 | 0 | 0 | 0 | 0 | 0 | 0 | 0 | 0 | 0 |
| CQQYYNTPYTF_CARDAVGATDRIPFDYW | 0 | 0 | 0 | 3 | 0 | 0 | 0 | 0 | 0 | 0 | 0 | 0 | 0 | 0 | 0 | 0 |
| CQQYYTTWTF_CSSTVAEGIDYW | 0 | 0 | 0 | 3 | 0 | 0 | 0 | 0 | 0 | 0 | 0 | 0 | 0 | 0 | 0 | 0 |
| CHQYYRNPYTF_CARVKDWELLPLSYW | 0 | 0 | 0 | 0 | 0 | 5 | 0 | 0 | 0 | 0 | 0 | 0 | 0 | 0 | 0 | 0 |
| CQQSYRTPFTF_CARHKGGDNRFDPW | 0 | 0 | 0 | 0 | 0 | 5 | 0 | 0 | 0 | 0 | 0 | 0 | 0 | 0 | 0 | 0 |
| CQVAGISGDHVIF_CGRGRVGRTHVAVDVW | 0 | 0 | 0 | 0 | 0 | 4 | 0 | 0 | 0 | 0 | 0 | 0 | 0 | 0 | 0 | 0 |
| CATRDDSLSVRVF_CASGQEYSGYDSAYW | 0 | 0 | 5 | 0 | 0 | 0 | 0 | 0 | 0 | 0 | 0 | 0 | 0 | 0 | 0 | 0 |
| CELYLGRGISVF_CATGGREQFNW | 0 | 0 | 3 | 0 | 0 | 0 | 0 | 0 | 0 | 0 | 0 | 0 | 0 | 0 | 0 | 0 |
| CTQATQFPYTF_CARDRDYIDYW | 0 | 0 | 0 | 0 | 0 | 0 | 0 | 5 | 0 | 0 | 0 | 0 | 0 | 0 | 0 | 0 |
| CMQGTHWITF_CARDDDTDPSHFDHW | 0 | 0 | 0 | 0 | 0 | 0 | 0 | 3 | 0 | 0 | 0 | 0 | 0 | 0 | 0 | 0 |
| CMQTTQFPQTF_CAKRREIFWLGEGRGTPDYW | 0 | 0 | 0 | 0 | 0 | 0 | 0 | 3 | 0 | 0 | 0 | 0 | 0 | 0 | 0 | 0 |
| CQQLSDNLPWTF_CARKIKEPGRYFDYW | 0 | 0 | 0 | 0 | 0 | 0 | 0 | 3 | 0 | 0 | 0 | 0 | 0 | 0 | 0 | 0 |
| CQQYGSSPWTF_CARVAESYYDFWSAKWEGAFDIW | 0 | 0 | 0 | 0 | 0 | 0 | 0 | 0 | 361 | 0 | 0 | 0 | 0 | 0 | 0 | 0 |
| CQQYNSYSF_CARSGPITIFGVVIKGRGGDAFDIW | 0 | 0 | 0 | 0 | 0 | 0 | 0 | 0 | 119 | 0 | 0 | 0 | 0 | 0 | 0 | 0 |
| CYSTDSSGNHYVF_CARVAESYYDFWSAKWEGAFDIW | 0 | 0 | 0 | 0 | 0 | 0 | 0 | 0 | 7 | 0 | 0 | 0 | 0 | 0 | 0 | 0 |
| CKQGIHLPYTF_CARDGDSYGHDFDYW | 0 | 0 | 0 | 0 | 0 | 0 | 0 | 0 | 7 | 0 | 0 | 0 | 0 | 0 | 0 | 0 |
| CMQGSHWPRTF_CARDRFFRTFDYW | 0 | 0 | 0 | 0 | 0 | 0 | 0 | 0 | 6 | 0 | 0 | 0 | 0 | 0 | 0 | 0 |
| CSYAGGYTWVF_CTRDVSYSSNCW | 0 | 0 | 0 | 0 | 0 | 0 | 0 | 0 | 5 | 0 | 0 | 0 | 0 | 0 | 0 | 0 |
| CQSYDSSLYGVIF_CARVAESYYDFWSAKWEGAFDIW | 0 | 0 | 0 | 0 | 0 | 0 | 0 | 0 | 5 | 0 | 0 | 0 | 0 | 0 | 0 | 0 |
| CSYAGSSTFVF_CAKNTDYDFWSDYPKGYWFGPW | 0 | 0 | 0 | 0 | 0 | 0 | 0 | 0 | 4 | 0 | 0 | 0 | 0 | 0 | 0 | 0 |
| CMQTLQTPHSF_CARGHSNYWSLSAVSLGYW | 0 | 0 | 0 | 0 | 0 | 0 | 0 | 0 | 4 | 0 | 0 | 0 | 0 | 0 | 0 | 0 |
| CSSYTTSTSPGF_CARDRTPNLSVLLSQFDYW | 0 | 0 | 0 | 0 | 0 | 0 | 0 | 0 | 4 | 0 | 0 | 0 | 0 | 0 | 0 | 0 |
| CMQALQTPLTF_CAKDLTPDGRWDIDYW | 0 | 0 | 0 | 0 | 0 | 0 | 0 | 0 | 3 | 0 | 0 | 0 | 0 | 0 | 0 | 0 |
| CQSYDSSLYGVIF_CARSGPITIFGVVIKGRGGDAFDIW | 0 | 0 | 0 | 0 | 0 | 0 | 0 | 0 | 3 | 0 | 0 | 0 | 0 | 0 | 0 | 0 |
| CQPYNTYWTF_CARDRSRFCSGDSCFPFDLW | 0 | 0 | 0 | 0 | 7 | 0 | 0 | 0 | 0 | 0 | 0 | 0 | 0 | 0 | 0 | 0 |
| CGTWDSSLSAGVF_CARDPATFSIYFPSGAPILGW | 0 | 0 | 0 | 0 | 5 | 0 | 0 | 0 | 0 | 0 | 0 | 0 | 0 | 0 | 0 | 0 |
| CQQYFSTPHTF_CARKQWLGPIDYW | 0 | 0 | 0 | 0 | 4 | 0 | 0 | 0 | 0 | 0 | 0 | 0 | 0 | 0 | 0 | 0 |
| CQQYYSIPSLTF_CATHRTYFDWLLPFDYW | 0 | 0 | 0 | 0 | 0 | 0 | 179 | 0 | 0 | 0 | 0 | 0 | 0 | 0 | 0 | 0 |
| CQHYYSSPPWTF_CARHRRSGSYGEALDLW | 0 | 0 | 0 | 0 | 0 | 0 | 154 | 0 | 0 | 0 | 0 | 0 | 0 | 0 | 0 | 0 |
| CSSYTSAATLVF_CARDPHTYDFWSEGDFL | 0 | 0 | 0 | 0 | 0 | 0 | 134 | 0 | 0 | 0 | 0 | 0 | 0 | 0 | 0 | 0 |
| CQQSYNTPYTF_CARDRNYDFWNGFYTSNYYYMDVW | 0 | 0 | 0 | 0 | 0 | 0 | 129 | 0 | 0 | 0 | 0 | 0 | 0 | 0 | 0 | 0 |
| CQVWDSTRDHPGVF_CAKSETSFGVTAPFDYW | 0 | 0 | 0 | 0 | 0 | 0 | 93 | 0 | 0 | 0 | 0 | 0 | 0 | 0 | 0 | 0 |
| CQQYNNLVLTF_CAKDERAYYHYMDVW | 0 | 0 | 0 | 0 | 0 | 0 | 76 | 0 | 0 | 0 | 0 | 0 | 0 | 0 | 0 | 0 |
| CLAWDDKNVVF_CARIHYYESNVYDHW | 0 | 0 | 0 | 0 | 0 | 0 | 70 | 0 | 0 | 0 | 0 | 0 | 0 | 0 | 0 | 0 |
| CMQALQTPETF_CARTQYSSSSDYW | 0 | 0 | 0 | 0 | 0 | 0 | 60 | 0 | 0 | 0 | 0 | 0 | 0 | 0 | 0 | 0 |
| CSSYAGSNNWVF_CARDDSAGILTGIGGYW | 0 | 0 | 0 | 0 | 0 | 0 | 40 | 0 | 0 | 0 | 0 | 0 | 0 | 0 | 0 | 0 |
| CQQAKSFPLTF_CTRYSLSSGWIDPW | 0 | 0 | 0 | 0 | 0 | 0 | 37 | 0 | 0 | 0 | 0 | 0 | 0 | 0 | 0 | 0 |
| CQAWDSTTVLF_CARDPHTYDFWSEGDFL | 0 | 0 | 0 | 0 | 0 | 0 | 23 | 0 | 0 | 0 | 0 | 0 | 0 | 0 | 0 | 0 |
| CGAWDDTLSAAVF_CARVFMLRDVDWSKTSQNYYMDVW | 0 | 0 | 0 | 0 | 0 | 0 | 10 | 0 | 0 | 0 | 0 | 0 | 0 | 0 | 0 | 0 |
| CQQYNNLVLTF_CARGIAARTFDYW | 0 | 0 | 0 | 0 | 0 | 0 | 9 | 0 | 0 | 0 | 0 | 0 | 0 | 0 | 0 | 0 |
| CNSFTTSSTLVF_CAKDERAYYHYMDVW | 0 | 0 | 0 | 0 | 0 | 0 | 7 | 0 | 0 | 0 | 0 | 0 | 0 | 0 | 0 | 0 |
| CSSYTSAATLVF_CVRQVFTLLGVPKNNYFDPW | 0 | 0 | 0 | 0 | 0 | 0 | 7 | 0 | 0 | 0 | 0 | 0 | 0 | 0 | 0 | 0 |
| CMQALQTPETF_CARGTAYYYGSGSYYKVLSGHHFDYW | 0 | 0 | 0 | 0 | 0 | 0 | 3 | 0 | 0 | 0 | 0 | 0 | 0 | 0 | 0 | 0 |
| CLLYYGGAQRVVF_CARVFSAIFLKIDYW | 0 | 0 | 0 | 0 | 0 | 0 | 3 | 0 | 0 | 0 | 0 | 0 | 0 | 0 | 0 | 0 |
| CKSYEGSQSYVF_CAKYHYGSGTSSGYW | 0 | 0 | 0 | 0 | 0 | 0 | 0 | 0 | 0 | 0 | 0 | 0 | 3 | 0 | 0 | 0 |
| CQQSYSTPALTF_CARSAPVVGATISYYGMDVW | 0 | 0 | 0 | 0 | 0 | 0 | 0 | 0 | 0 | 0 | 0 | 0 | 3 | 0 | 0 | 0 |
| CQQTYSTPRLTF_CARSHDSSDYYYRTYYYGMDVW | 0 | 0 | 0 | 0 | 0 | 0 | 0 | 0 | 0 | 0 | 0 | 0 | 3 | 0 | 0 | 0 |
| CQQYYSTQTF_CARDLTGYFDYW | 0 | 0 | 0 | 0 | 0 | 0 | 0 | 0 | 0 | 0 | 0 | 0 | 3 | 0 | 0 | 0 |
| CSSYAGSNNYVF_CARHRSGGYYDYW | 0 | 0 | 0 | 0 | 0 | 0 | 0 | 0 | 0 | 0 | 0 | 0 | 3 | 0 | 0 | 0 |
| CLLSYSGPWVF_CARARGGSYSVSQSEGRLDYW | 0 | 0 | 0 | 0 | 0 | 0 | 0 | 0 | 0 | 0 | 0 | 0 | 3 | 0 | 0 | 0 |
| CAAWDDSLSGNWVF_CARDQSIHDILTGYYYGMDVW | 0 | 0 | 0 | 0 | 0 | 0 | 0 | 0 | 0 | 0 | 0 | 0 | 0 | 11 | 0 | 0 |
| CMQGIHLPFTF_CARAGYSSGWYLGDYYYYGMDVW | 0 | 0 | 0 | 0 | 0 | 0 | 0 | 0 | 0 | 0 | 0 | 0 | 0 | 5 | 0 | 0 |
| CQKYNSAPRTF_CARIQLWLDYYYYYGMDVW | 0 | 0 | 0 | 0 | 0 | 0 | 0 | 0 | 0 | 0 | 0 | 0 | 0 | 5 | 0 | 0 |
| CAAWDDSLNGWVF_CARDEYYGSGSYYSLGYYYYGMDVW | 0 | 0 | 0 | 0 | 0 | 0 | 0 | 0 | 0 | 0 | 0 | 0 | 0 | 5 | 0 | 0 |
| CMQGTHWPPLTF_CARGPPRLNFDWLLLENNWFDPW | 0 | 0 | 0 | 0 | 0 | 0 | 0 | 0 | 0 | 0 | 0 | 0 | 0 | 4 | 0 | 0 |
| CSSYTSSSISYVF_CARVRRVWTTGTGVAFDIW | 0 | 0 | 0 | 0 | 0 | 0 | 0 | 0 | 0 | 0 | 0 | 0 | 0 | 4 | 0 | 0 |
| CGTWDSDLSAWVF_CARIRRESGSQFSIDYW | 0 | 0 | 0 | 0 | 0 | 0 | 0 | 0 | 0 | 0 | 0 | 0 | 0 | 4 | 0 | 0 |
| CQQYNSYSYSF-CAKVPSNMVLYFSHVW | 0 | 0 | 0 | 0 | 0 | 0 | 0 | 0 | 0 | 5 | 0 | 0 | 0 | 4 | 0 | 0 |
| CARHVLRRITIFGVVSPNWYFDLW-CQQANSFPLTF | 0 | 10 | 0 | 0 | 0 | 0 | 0 | 0 | 0 | 0 | 0 | 0 | 0 | 0 | 0 | 0 |
| CARVPPARITMFGVVTDLREQWFDSW-CDHPPQTF | 0 | 8 | 0 | 0 | 0 | 0 | 0 | 0 | 0 | 0 | 0 | 0 | 0 | 0 | 0 | 0 |
| CARVPPARITMFGVVTDLREQWFDSW-CQQYDNLPLTF | 0 | 6 | 0 | 0 | 0 | 0 | 0 | 0 | 0 | 0 | 0 | 0 | 0 | 0 | 0 | 0 |
| CARAYNWDYRKNYMDVW-CSSYTRSNTLVF | 0 | 6 | 0 | 0 | 0 | 0 | 0 | 0 | 0 | 0 | 121 | 0 | 0 | 0 | 0 | 0 |
| CARDGLGVTPLDSW-CLQYVYSSWTF | 0 | 6 | 0 | 0 | 0 | 0 | 0 | 0 | 0 | 0 | 63 | 0 | 0 | 0 | 0 | 0 |
| CAKDKRGWLYDRGSIKGSFDYW-CSYAGSSTYVF | 0 | 6 | 0 | 0 | 0 | 0 | 0 | 0 | 0 | 0 | 55 | 0 | 0 | 0 | 0 | 0 |
| CAKEGGDYGEGGGYW-CQSADSSGTWVF | 0 | 6 | 0 | 0 | 0 | 0 | 0 | 0 | 0 | 0 | 43 | 0 | 0 | 0 | 0 | 0 |
| CAKDRGSDYGDQADYW-CSSYAGSKVVF | 0 | 6 | 0 | 0 | 0 | 0 | 0 | 0 | 0 | 0 | 39 | 0 | 0 | 0 | 0 | 0 |
| CQQYGTSYTF | 0 | 6 | 0 | 0 | 0 | 0 | 0 | 0 | 0 | 0 | 36 | 0 | 0 | 0 | 0 | 0 |
| CATVGVSAHGYFDYW-CQQYGTSYTF | 0 | 6 | 0 | 0 | 0 | 0 | 0 | 0 | 0 | 0 | 33 | 0 | 0 | 0 | 0 | 0 |
| CARHLTHIVVVVAAIGWFDPW-CQQYGRSPYTF | 0 | 6 | 0 | 0 | 0 | 0 | 0 | 0 | 0 | 0 | 27 | 0 | 0 | 0 | 0 | 0 |
| CQQYGRSPYTF | 0 | 6 | 0 | 0 | 0 | 0 | 0 | 0 | 0 | 0 | 27 | 0 | 0 | 0 | 0 | 0 |
| CATVGVSAHGYFDYW-CQQYGRSPYTF | 0 | 6 | 0 | 0 | 0 | 0 | 0 | 0 | 0 | 0 | 25 | 0 | 0 | 0 | 0 | 0 |
| CARDPLWSSSWLFQHW-CQQYDNLSITF | 0 | 6 | 0 | 0 | 0 | 0 | 0 | 0 | 0 | 0 | 23 | 0 | 0 | 0 | 0 | 0 |
| CARHLTHIVVVVAAIGWFDPW-CQQYGTSYTF | 0 | 6 | 0 | 0 | 0 | 0 | 0 | 0 | 0 | 0 | 22 | 0 | 0 | 0 | 0 | 0 |
| CAKSVRDFYRYGAFDFW-CTSHTYYDTVVF | 0 | 6 | 0 | 0 | 0 | 0 | 0 | 0 | 0 | 0 | 22 | 0 | 0 | 0 | 0 | 0 |
| CARYESASWAGNRFGMW-CSSYTTTNTHWVF | 0 | 6 | 0 | 0 | 0 | 0 | 0 | 0 | 0 | 0 | 21 | 0 | 0 | 0 | 0 | 0 |
| CAGWDDSLPAWVF | 0 | 6 | 0 | 0 | 0 | 0 | 0 | 0 | 0 | 0 | 18 | 0 | 0 | 0 | 0 | 0 |
| CARAHRIAVAGHPPFFDYW-CQTWDSSLVVF | 0 | 6 | 0 | 0 | 0 | 0 | 0 | 0 | 0 | 0 | 18 | 0 | 0 | 0 | 0 | 0 |
| CQSADSSGTWVF | 0 | 6 | 0 | 0 | 0 | 0 | 0 | 0 | 0 | 0 | 17 | 0 | 0 | 0 | 0 | 0 |
| CSSYTRSNTLVF | 0 | 6 | 0 | 0 | 0 | 0 | 0 | 0 | 0 | 0 | 16 | 0 | 0 | 0 | 0 | 0 |
| CAAWDDSLNGWVF | 0 | 6 | 0 | 0 | 0 | 0 | 0 | 0 | 0 | 0 | 15 | 0 | 0 | 0 | 0 | 0 |
| CARILPFGEFFDYW-CQQYGSSPMYTF | 0 | 6 | 0 | 0 | 0 | 0 | 0 | 0 | 0 | 0 | 15 | 0 | 0 | 0 | 0 | 0 |
| CARDARRTSPQEGLGSTW-CAAWDDSLNGWVF | 0 | 6 | 0 | 0 | 0 | 0 | 0 | 0 | 0 | 0 | 15 | 0 | 0 | 0 | 0 | 0 |
| CQQYNNWPPWTF | 0 | 6 | 0 | 0 | 0 | 0 | 0 | 0 | 0 | 0 | 13 | 0 | 0 | 0 | 0 | 0 |
| CSSYAGSKVVF | 0 | 6 | 0 | 0 | 0 | 0 | 0 | 0 | 0 | 0 | 13 | 0 | 0 | 0 | 0 | 0 |
| CTTGVVKFLERYMDVW-CAGWDDSLPAWVF | 0 | 6 | 0 | 0 | 0 | 0 | 0 | 0 | 0 | 0 | 12 | 0 | 0 | 0 | 0 | 0 |
| CAAWDDSLGNVF | 0 | 6 | 0 | 0 | 0 | 0 | 0 | 0 | 0 | 0 | 12 | 0 | 0 | 0 | 0 | 0 |
| CQQYNNWPPLTF | 0 | 6 | 0 | 0 | 0 | 0 | 0 | 0 | 0 | 0 | 10 | 0 | 0 | 0 | 0 | 0 |
| CASWDDKMSGVLF | 0 | 6 | 0 | 0 | 0 | 0 | 0 | 0 | 0 | 0 | 0 | 1085 | 0 | 0 | 0 | 0 |
| CAAADHGDHDISKYYYIDVW-CAGWDDGLSAWVF | 0 | 6 | 0 | 0 | 0 | 0 | 0 | 0 | 0 | 0 | 0 | 167 | 0 | 0 | 0 | 0 |
| CATGGEPWDSSSYLDFW-CSSYTSSSTSYVF | 0 | 6 | 0 | 0 | 0 | 0 | 0 | 0 | 0 | 0 | 0 | 121 | 0 | 0 | 0 | 0 |
| CAGWDDGLSAWVF | 0 | 6 | 0 | 0 | 0 | 0 | 0 | 0 | 0 | 0 | 0 | 64 | 0 | 0 | 0 | 0 |
| CSSYTSSSTSYVF | 0 | 6 | 0 | 0 | 0 | 0 | 0 | 0 | 0 | 0 | 0 | 36 | 0 | 0 | 0 | 0 |
| CQQYNNWPLTF-CASWDDKMSGVLF | 0 | 6 | 0 | 0 | 0 | 0 | 0 | 0 | 0 | 0 | 0 | 14 | 0 | 0 | 0 | 0 |
| CQQYNTSPYSF-CASWDDKMSGVLF | 0 | 6 | 0 | 0 | 0 | 0 | 0 | 0 | 0 | 0 | 0 | 11 | 0 | 0 | 0 | 0 |
| CVRHYDFWSGTPPPL-CASWDDKMSGVLF | 0 | 6 | 0 | 0 | 0 | 0 | 0 | 0 | 0 | 0 | 0 | 10 | 0 | 0 | 0 | 0 |
| CQAWDSSTVVF | 0 | 6 | 0 | 0 | 0 | 0 | 0 | 0 | 0 | 0 | 0 | 0 | 0 | 0 | 0 | 3 |
| CSSYTSSSTLVF | 0 | 6 | 0 | 0 | 0 | 0 | 0 | 0 | 0 | 0 | 0 | 0 | 0 | 0 | 0 | 3 |
| CQQYNSYPYTF | 0 | 6 | 0 | 0 | 0 | 0 | 0 | 0 | 0 | 0 | 0 | 0 | 0 | 0 | 0 | 3 |
| CAKRVHLYRSSQGYFDSR;IGK:CDNWPDTF | 0 | 6 | 0 | 0 | 0 | 0 | 0 | 0 | 0 | 0 | 0 | 0 | 0 | 0 | 5 | 0 |

**Table S9. The distribution of technical features of each sample.**

| **Sample** | **Library** | **Estimated Number of Cells** | **Cell Count Confidence** | **Mean Read Pairs per Cell** | **Mean Used Read Pairs per Cell** | **Fraction Reads in Cells** | **Sequencing** | **Number of Read Pairs** | **Valid Barcodes** | **Q30 Bases in Barcode** | **Q30 Bases in RNA Read 1** | **Q30 Bases in RNA Read 2** | **Q30 Bases in Sample Index** | **Q30 Bases in UMI** | **Enrichment** | **Reads Mapped to Any V(D)J Gene** | **Reads Mapped to IGH** | **Reads Mapped to IGK** | **Reads Mapped to IGL** |
| --- | --- | --- | --- | --- | --- | --- | --- | --- | --- | --- | --- | --- | --- | --- | --- | --- | --- | --- | --- |
| P1-1 | 5'mRNA | 6427 | 0.832 | 59426 | 1345 | 19646 | 3758 |  | 381,936,821 | 89.40% | 83.20% | 95.00% | 88.20% | 91.20% | 94.70% |  |  |  |  |
| P1-1 | BCR | 3,805 | 97.20% | 14,999 | 4,960 | 92.90% |  | 57,074,054 | 96.90% | 95.00% | 93.70% | 91.80% | 91.50% | 94.70% |  | 97.00% | 17.80% | 44.90% | 31.60% |
| P1-1 | TCR | 2,679 | 85.60% | 19,995 | 13,146 | 84.00% |  | 53,566,869 | 90.30% | 95.50% | 93.50% | 92.10% | 92.00% | 95.20% |  | 82.40% | 15.90% | 51.30% |  |
| P1-2 | 5'mRNA | 11,059 | 75.10% | 28,694 | 1,355 | 20,425 | 2,996 |  | 317,336,529 | 84.30% | 61.60% | 96.20% | 88.00% | 89.10% | 96.00% |  |  |  |  |
| P1-2 | BCR | 324 | 88.00% | 113,779 | 21,897 | 73.00% |  | 36,864,583 | 93.00% | 95.60% | 94.20% | 88.70% | 94.10% | 94.80% |  | 90.40% | 17.80% | 43.00% | 23.80% |
| P1-2 | TCR | 8,976 | 80.70% | 45,363 | 20,416 | 81.40% |  | 407,178,574 | 82.00% | 95.70% | 93.60% | 90.40% | 93.00% | 95.00% |  | 64.20% | 10.00% | 32.90% |  |
| P1-3 | 5'mRNA | 13,792 | 98.10% | 30,832 | 1,192 | 20,156 | 3,149 |  | 425,235,293 | 92.90% | 81.40% | 96.00% | 92.00% | 94.60% | 95.90% |  |  |  |  |
| P1-3 | BCR | 797 | 98.70% | 35,547 | 8,555 | 97.10% |  | 28,331,572 | 95.80% | 94.00% | 92.10% | 88.50% | 88.60% | 93.00% |  | 97.40% | 20.80% | 51.20% | 22.80% |
| P1-3 | TCR | 8,807 | 85.70% | 3,432 | 3,113 | 97.70% |  | 30,230,287 | 96.50% | 94.90% | 91.90% | 89.70% | 84.90% | 93.70% |  | 89.20% | 18.60% | 56.20% |  |
| P2-1 | 5'mRNA | 1,812 | 72.10% | 171,396 | 1,665 | 17,560 | 4,249 |  | 310,569,595 | 73.60% | 81.80% | 96.00% | 88.70% | 94.10% | 96.00% |  |  |  |  |
| P2-1 | BCR | 164 | 97.00% | 188,749 | 23,942 | 83.80% |  | 30,954,983 | 92.80% | 95.70% | 93.90% | 88.00% | 93.80% | 94.10% |  | 90.70% | 18.00% | 42.70% | 25.60% |
| P2-1 | TCR | 235 | 100.00% | 140,970 | 26,720 | 37.60% |  | 33,128,040 | 74.60% | 95.40% | 93.20% | 87.60% | 92.90% | 95.10% |  | 50.80% | 10.60% | 19.60% |  |
| P2-2 | 5'mRNA | 13,905 | 95.10% | 26,084 | 1,144 | 19,992 | 2,995 |  | 362,700,712 | 93.80% | 78.60% | 96.00% | 91.30% | 86.50% | 95.90% |  |  |  |  |
| P2-2 | BCR | 353 | 95.80% | 77,410 | 25,501 | 97.40% |  | 27,326,025 | 97.00% | 94.40% | 92.30% | 88.60% | 90.20% | 93.20% |  | 97.80% | 13.30% | 46.00% | 36.40% |
| P2-2 | TCR | 8,958 | 88.40% | 3,514 | 3,143 | 96.00% |  | 31,486,384 | 96.80% | 94.90% | 91.90% | 89.40% | 89.60% | 93.70% |  | 90.10% | 20.40% | 56.20% |  |
| P2-3 | 5'mRNA | 11,027 | 93.60% | 26,348 | 1,383 | 19,760 | 4,082 |  | 290,544,007 | 93.70% | 69.10% | 97.20% | 92.50% | 96.10% | 97.50% |  |  |  |  |
| P2-3 | BCR | 140 | 98.60% | 191,915 | 22,296 | 92.60% |  | 26,868,168 | 96.30% | 95.60% | 95.10% | 91.70% | 94.60% | 95.00% |  | 95.60% | 21.00% | 40.10% | 30.70% |
| P2-3 | TCR | 5,257 | 90.20% | 6,249 | 4,902 | 92.70% |  | 32,855,043 | 93.10% | 96.60% | 95.50% | 93.50% | 96.70% | 96.10% |  | 87.90% | 20.10% | 51.40% |  |
| P3-1 | 5'mRNA | 13,365 | 89.70% | 35,227 | 1,578 | 20,872 | 4,152 |  | 470,821,213 | 91.70% | 75.00% | 96.10% | 89.90% | 92.60% | 96.10% |  |  |  |  |
| P3-1 | BCR | 567 | 98.10% | 75,580 | 37,616 | 94.80% |  | 42,854,316 | 97.60% | 94.80% | 93.80% | 89.30% | 94.10% | 94.70% |  | 98.40% | 24.30% | 32.60% | 39.70% |
| P3-1 | TCR | 8,910 | 91.00% | 6,443 | 5,326 | 94.20% |  | 57,407,503 | 94.90% | 95.70% | 93.60% | 92.40% | 89.10% | 95.40% |  | 87.70% | 22.70% | 49.90% |  |
| P3-2 | 5'mRNA | 10,390 | 88.50% | 36,547 | 967 | 18,667 | 3,238 |  | 379,723,580 | 94.60% | 77.40% | 96.00% | 91.50% | 92.10% | 95.90% |  |  |  |  |
| P3-2 | BCR | 688 | 99.70% | 58,078 | 14,381 | 92.80% |  | 39,958,086 | 96.00% | 94.70% | 92.20% | 90.60% | 89.10% | 93.40% |  | 94.90% | 20.20% | 36.30% | 32.80% |
| P3-2 | TCR | 3,544 | 96.30% | 12,262 | 9,606 | 93.00% |  | 43,457,847 | 93.00% | 94.70% | 91.30% | 90.60% | 88.80% | 93.70% |  | 87.10% | 15.50% | 58.80% |  |
| P4 | 5'mRNA | 10,149 | 86.90% | 36,477 | 1,231 | 20,196 | 3,173 |  | 370,209,420 | 92.80% | 80.00% | 94.90% | 88.80% | 92.50% | 94.60% |  |  |  |  |
| P4 | BCR | 455 | 98.90% | 103,304 | 15,560 | 93.50% |  | 47,003,662 | 96.60% | 94.80% | 93.70% | 90.00% | 92.90% | 94.70% |  | 96.60% | 23.20% | 29.60% | 40.70% |
| P4 | TCR | 6,293 | 85.70% | 9,384 | 7,823 | 93.50% |  | 59,054,260 | 95.30% | 95.60% | 93.70% | 92.40% | 93.00% | 95.30% |  | 89.20% | 20.40% | 55.40% |  |
| P5 | 5'mRNA | 15,186 | 92.30% | 25,335 | 1,453 | 20,888 | 3,925 |  | 384,738,668 | 91.40% | 68.90% | 95.00% | 89.40% | 90.70% | 94.70% |  |  |  |  |
| P5 | BCR | 421 | 98.30% | 67,481 | 22,315 | 72.60% |  | 28,409,784 | 93.80% | 95.10% | 93.70% | 90.80% | 91.20% | 95.20% |  | 86.00% | 16.40% | 40.60% | 19.50% |
| P5 | TCR | 10,116 | 84.40% | 6,239 | 5,115 | 94.70% |  | 63,121,249 | 94.40% | 95.60% | 93.60% | 92.40% | 92.60% | 95.30% |  | 86.90% | 23.80% | 48.50% |  |
| P6-1 | 5'mRNA | 13,925 | 88.40% | 28,566 | 1,363 | 20,882 | 4,016 |  | 397,790,128 | 91.20% | 67.80% | 94.80% | 89.10% | 92.70% | 94.50% |  |  |  |  |
| P6-1 | BCR | 205 | 98.00% | 254,549 | 28,603 | 84.70% |  | 52,182,605 | 97.00% | 95.40% | 94.30% | 92.60% | 90.40% | 94.90% |  | 97.10% | 22.30% | 49.50% | 22.30% |
| P6-1 | TCR | 8,603 | 94.90% | 7,981 | 5,885 | 90.30% |  | 68,665,420 | 92.10% | 95.50% | 93.50% | 92.10% | 94.20% | 95.20% |  | 84.80% | 19.90% | 49.20% |  |
| P6-2 | 5'mRNA | 7,843 | 58.40% | 39,154 | 1,043 | 19,681 | 1,797 |  | 307,091,456 | 80.00% | 64.10% | 96.00% | 87.40% | 94.30% | 95.90% |  |  |  |  |
| P6-2 | BCR | 293 | 81.90% | 117,277 | 13,389 | 35.70% |  | 34,362,375 | 88.10% | 95.50% | 94.10% | 88.30% | 93.60% | 94.60% |  | 85.40% | 12.60% | 29.40% | 35.10% |
| P6-2 | TCR | 2,527 | 84.10% | 14,215 | 4,046 | 45.40% |  | 35,922,119 | 81.10% | 95.50% | 93.70% | 89.70% | 94.60% | 95.10% |  | 70.90% | 17.00% | 34.60% |  |
| P7-1 | 5'mRNA | 17,249 | 90.80% | 21,554 | 1,252 | 20,662 | 3,738 |  | 371,788,768 | 91.90% | 66.20% | 95.00% | 89.50% | 92.70% | 94.70% |  |  |  |  |
| P7-1 | BCR | 408 | 97.60% | 118,671 | 38,050 | 63.10% |  | 48,418,047 | 92.10% | 95.20% | 93.30% | 89.10% | 93.50% | 95.00% |  | 80.90% | 18.20% | 36.00% | 10.70% |
| P7-1 | TCR | 11,055 | 91.20% | 5,951 | 4,823 | 94.50% |  | 65,789,368 | 93.70% | 95.60% | 93.50% | 91.80% | 93.10% | 95.20% |  | 88.30% | 18.80% | 53.90% |  |
| P7-2 | 5'mRNA | 12,087 | 67.60% | 24,982 | 969 | 20,441 | 1,916 |  | 301,960,393 | 80.70% | 57.30% | 96.10% | 87.90% | 93.00% | 96.00% |  |  |  |  |
| P7-2 | BCR | 835 | 75.70% | 42,884 | 8,657 | 57.40% |  | 35,808,609 | 89.60% | 95.40% | 94.20% | 88.40% | 94.20% | 94.80% |  | 85.60% | 15.80% | 39.00% | 23.00% |
| P7-2 | TCR | 4,414 | 80.90% | 34,204 | 10,439 | 53.30% |  | 150,980,611 | 80.70% | 95.70% | 93.60% | 90.50% | 92.40% | 95.10% |  | 66.00% | 13.30% | 32.70% |  |
| P8 | 5'mRNA | 9,350 | 84.70% | 44,613 | 322 | 18,938 | 3,406 |  | 417,136,234 | 93.20% | 79.30% | 95.60% | 90.80% | 92.20% | 95.50% |  |  |  |  |
| P8 | BCR | 472 | 90.70% | 131,403 | 33,998 | 89.60% |  | 62,022,584 | 96.90% | 95.30% | 94.00% | 92.00% | 92.00% | 94.90% |  | 97.60% | 20.20% | 36.70% | 38.10% |
| P8 | TCR | 2,150 | 91.60% | 27,647 | 19,222 | 83.20% |  | 59,442,392 | 92.60% | 95.50% | 93.50% | 92.00% | 93.50% | 95.10% |  | 85.70% | 18.70% | 49.80% |  |
| P9-1 | 5'mRNA | 33,506 | 93.10% | 12,682 | 957 | 21,317 | 2,478 |  | 424,932,082 | 92.10% | 60.00% | 95.50% | 90.90% | 93.10% | 95.30% |  |  |  |  |
| P9-1 | BCR | 772 | 97.00% | 68,572 | 9,851 | 92.90% |  | 52,937,751 | 96.60% | 95.20% | 93.90% | 91.50% | 92.70% | 94.90% |  | 96.50% | 21.40% | 34.80% | 36.80% |
| P9-1 | TCR | 24,754 | 83.80% | 2,543 | 2,083 | 93.40% |  | 62,967,257 | 95.40% | 95.70% | 93.50% | 92.10% | 92.00% | 95.30% |  | 87.70% | 27.90% | 44.20% |  |
| P9-2 | 5'mRNA | 16,142 | 94.20% | 18,029 | 1,225 | 20,393 | 3,233 |  | 291,037,495 | 93.30% | 69.80% | 97.30% | 93.70% | 93.20% | 97.40% |  |  |  |  |
| P9-2 | BCR | 1,475 | 99.90% | 21,768 | 13,924 | 88.20% |  | 32,109,106 | 95.20% | 96.00% | 95.30% | 91.80% | 93.70% | 95.70% |  | 92.30% | 19.20% | 58.80% | 6.90% |
| P9-2 | TCR | 10,253 | 76.50% | 3,220 | 2,492 | 92.10% |  | 33,021,320 | 92.90% | 96.50% | 95.60% | 93.40% | 93.50% | 96.00% |  | 86.90% | 18.30% | 54.20% |  |
| P10-1 | 5'mRNA | 5,870 | 71.10% | 70,032 | 1,240 | 19,449 | 2,794 |  | 411,092,116 | 92.20% | 87.70% | 95.60% | 88.60% | 92.00% | 95.30% |  |  |  |  |
| P10-1 | BCR | 378 | 96.90% | 136,874 | 16,078 | 83.90% |  | 51,738,652 | 94.50% | 95.30% | 93.80% | 90.30% | 91.20% | 95.10% |  | 91.40% | 18.20% | 30.50% | 35.20% |
| P10-1 | TCR | 1,860 | 73.70% | 35,420 | 20,348 | 68.30% |  | 65,882,557 | 93.60% | 95.80% | 93.70% | 92.00% | 85.90% | 95.50% |  | 87.60% | 21.00% | 53.60% |  |
| P10-2 | 5'mRNA | 3,883 | 72.20% | 82,576 | 1,319 | 18,829 | 3,179 |  | 320,645,249 | 79.60% | 81.90% | 96.10% | 87.60% | 94.40% | 96.00% |  |  |  |  |
| P10-2 | BCR | 223 | 92.00% | 161,117 | 23,091 | 80.10% |  | 35,929,185 | 93.50% | 95.70% | 93.80% | 89.10% | 87.40% | 95.00% |  | 92.10% | 18.60% | 42.90% | 25.70% |
| P10-2 | TCR | 1,303 | 89.60% | 29,922 | 11,413 | 57.70% |  | 38,988,505 | 82.80% | 95.60% | 93.70% | 90.20% | 92.50% | 95.10% |  | 71.80% | 13.20% | 41.20% |  |
| P11 | 5'mRNA | 30,598 |  | 10,023 |  | 81.80% |  |  | 90.80% | 96.80% |  |  | 94.60% | 96.50% |  |  |  |  |  |
| P11 | BCR | 6,907 | 49.40% | 8,046 | 4,234 | 93.10% |  | 55,577,615 | 96.50% | 96.20% | 95.30% | 92.10% | 95.30% | 95.20% |  | 96.30% | 18.10% | 39.10% | 35.10% |
| P11 | TCR | 12,086 |  | 4,728 | 2,295 | 64.50% |  | 57,145,155 | 91.30% | 96.30% | 94.40% | 91.80% | 94.00% | 95.50% |  | 77.80% | 15.60% | 43.70% |  |
| P12 | 5'mRNA | 15,536 |  | 19,159 |  | 74.70% |  |  | 93.60% | 96.80% |  |  | 93.50% | 96.60% |  |  |  |  |  |
| P12 | BCR | 2,345 | 83.90% | 20,613 | 6,383 | 79.30% |  | 48,339,382 | 95.60% | 95.60% | 94.80% | 89.90% | 95.20% | 94.70% |  | 90.90% | 15.50% | 36.70% | 31.20% |
| P12 | TCR | 5,914 | 55.10% | 9,389 | 4,477 | 61.00% |  |  | 91.80% | 96.30% | 94.60% | 91.70% | 95.00% | 95.50% |  | 80.10% | 13.40% | 51.30% |  |
| P13 | 5'mRNA | 6,811 | 87.70% | 74,123 | 1,509 | 19,426 | 4,399 |  | 504,858,502 | 92.70% | 88.20% | 97.40% | 90.60% | 91.80% | 97.30% |  |  |  |  |
| P13 | BCR | 861 | 99.80% | 56,026 | 13,945 | 93.40% |  | 48,238,736 | 96.90% | 96.40% | 95.80% | 91.90% | 92.50% | 95.70% |  | 97.20% | 21.60% | 47.50% | 25.10% |
| P13 | TCR | 5,033 | 96.70% | 10,803 | 8,923 | 91.70% |  | 54,374,405 | 95.20% | 97.00% | 95.50% | 92.70% | 95.20% | 96.10% |  | 88.50% | 21.10% | 53.00% |  |
| P14 | 5'mRNA | 6,077 | 90.40% | 90,957 | 1,700 | 19,556 | 4,499 |  | 552,746,494 | 94.10% | 88.80% | 97.30% | 91.10% | 95.40% | 97.30% |  |  |  |  |
| P14 | BCR | 1,107 | 98.50% | 48,473 | 17,417 | 98.00% |  | 53,660,234 | 97.70% | 96.40% | 95.80% | 92.40% | 95.40% | 95.60% |  | 99.00% | 24.70% | 43.30% | 29.60% |
| P14 | TCR | 4,014 | 89.40% | 12,376 | 10,152 | 91.50% |  | 49,678,048 | 95.40% | 96.70% | 95.10% | 92.40% | 94.60% | 95.70% |  | 88.10% | 19.80% | 55.20% |  |
| P15 | 5'mRNA | 7,266 |  | 50,913 |  | 89.20% |  |  | 93.80% | 96.00% |  |  | 93.10% | 96.00% |  |  |  |  |  |
| P15 | BCR | 1017 | 100.00% | 40,494 | 15,891 | 94.80% |  | 41,182,481 | 93.20% | 95.10% | 93.90% | 90.80% | 91.40% | 93.50% |  | 95.30% | 22.10% | 29.60% | 39.20% |
| P15 | TCR | 2,904 | 93.80% | 13,653 | 10,370 | 89.80% |  | 39,648,532 | 92.70% | 95.10% | 93.50% | 91.00% | 88.10% | 93.90% |  | 87.30% | 14.20% | 59.10% |  |
| P16 | 5'mRNA | 8,237 |  | 12,501 |  | 60.70% |  |  | 91.70% | 96.80% |  |  | 94.80% | 96.60% |  |  |  |  |  |
| P16 | BCR | 800 | 67.30% | 35,284 | 6,452 | 54.70% |  | 28,227,290 | 89.10% | 95.80% | 94.20% | 89.90% | 92.50% | 95.00% |  | 87.00% | 18.20% | 29.80% | 29.70% |
| P16 | TCR | 2,476 | 100.00% | 20,859 | 7,493 | 40.80% |  | 51,647,788 | 94.60% | 96.20% | 94.40% | 90.80% | 93.10% | 95.40% |  | 81.70% | 14.70% | 51.00% |  |
| NC-1 | 5'mRNA | 9,181 | 95.40% | 37,469 | 1,339 | 19,693 | 4,146 |  | 344,007,014 | 92.80% | 79.20% | 95.60% | 89.60% | 93.20% | 95.30% |  |  |  |  |
| NC-1 | BCR | 1,116 | 99.60% | 49,584 | 12,474 | 87.30% |  | 55,336,646 | 95.40% | 95.40% | 94.30% | 91.30% | 91.10% | 95.20% |  | 93.60% | 17.40% | 53.60% | 17.50% |
| NC-1 | TCR | 5,578 | 76.80% | 11,050 | 8,431 | 89.80% |  | 61,640,571 | 93.30% | 95.50% | 93.70% | 92.30% | 90.50% | 95.10% |  | 88.70% | 8.80% | 67.10% |  |
| NC-2 | 5'mRNA | 25,893 | 93.80% | 17,869 | 1,138 | 21,095 | 3,341 |  | 462,703,425 | 94.70% | 58.80% | 95.00% | 89.90% | 92.40% | 94.80% |  |  |  |  |
| NC-2 | BCR | 745 | 87.10% | 80,978 | 26,594 | 96.60% |  | 60,328,628 | 97.40% | 95.10% | 94.20% | 92.60% | 92.50% | 94.70% |  | 99.00% | 27.30% | 38.80% | 31.80% |
| NC-2 | TCR | 14,062 | 90.10% | 4,790 | 3,810 | 93.30% |  | 67,361,705 | 94.00% | 95.50% | 93.50% | 92.10% | 92.50% | 95.10% |  | 88.30% | 18.90% | 54.50% |  |
| NC-3 | 5'mRNA | 4,854 | 89.70% | 118,570 | 1,582 | 19,563 | 4,388 |  | 575,541,174 | 93.70% | 91.80% | 97.30% | 90.50% | 95.20% | 97.30% |  |  |  |  |
| NC-3 | BCR | 1,129 | 99.50% | 42,454 | 11,841 | 96.20% |  | 47,931,475 | 97.70% | 96.10% | 95.50% | 92.10% | 95.70% | 95.40% |  | 98.40% | 22.50% | 45.60% | 28.00% |
| NC-3 | TCR | 2,669 | 92.80% | 19,302 | 15,238 | 89.30% |  | 51,518,552 | 94.60% | 96.90% | 95.50% | 92.70% | 93.50% | 96.00% |  | 89.20% | 18.80% | 55.80% |  |
